# 5-Methylcytosine-Related Long Noncoding RNAs Are Potential Biomarkers to Predict Overall Survival and Regulate Tumor-Immune Environment in Patients with Bladder Cancer

**DOI:** 10.1155/2022/3117359

**Published:** 2022-03-04

**Authors:** Zhuoyuan Li, Siyu Wang, Yuxian Chen, Yaozhou Huang, Tianping Li

**Affiliations:** ^1^Pharmaceutical Department of Sichuan University West China Hospital, Chengdu, Sichuan 610041, China; ^2^China Pharmaceutical University, #639 Longmian Avenue, Jiangning District, Nanjing, Jiangsu 211198, China

## Abstract

The role of 5-methylcytosine-related long noncoding RNAs (m5C-lncRNAs) in bladder cancer (BLCA) remains unclear. Here, we aim to study the prognostic value, gene expression characteristics, and correlation between the m5C-lncRNA risk model and the tumor microenvironment, immune infiltration, and tumor mutations in BLCA. After collecting BLCA patient RNA sequence transcriptome data, clinical information and mutation data from the Cancer Genome Atlas (TCGA) database, 17 m5C-related lncRNAs independently correlated with OS were obtained by Lasso and multivariate Cox regression analysis, and a risk model was constructed. Univariate Cox, multivariate Cox regression analysis, and the C-index curve proved that the risk model was a significant independent prognostic indicator for patients with BLCA. ESTIMATE and CIBERSORT indicated that the higher the number of immune cells and stromal cells in TME, the higher the prognostic risk. We found that in the low-risk group, the expression levels of immune cells that predicted a good prognosis were higher, including plasma cells, regulatory T cells, and CD 8 T cells. There is a negative correlation between TMB and risk score. The TMB of the low-risk group is significantly higher than that of the high-risk group. In conclusion, the m5C-related risk model is crucial to predict the prognosis of patients with BLCA.

## 1. Introduction

Bladder cancer (BLCA), one of the ten most common cancer types worldwide, is a growing public health concern [1]. Approximately 3.0% of all new cancer diagnoses and 2.1% of all cancer deaths are due to urinary BLCA, imposing a significant burden on the society [2]. BLCA is a common type of malignant tumor in the urinary system, and the mortality rate of patients with BLCA above T2 increases [3, 4]. Until now, the main treatments for BLCA are radical cystectomy and chemotherapy, which will change patients' lifestyle [5, 6]. As research continues, molecular targeted therapy and immunotherapy are increasingly showing the advantages of precise treatment [7]. Despite the high recurrence rate of BLCA, the discovery and application of biomarkers can help make the best treatment decisions, which will have a positive impact on improving the prognosis of BLCA [8].

Methylation is one of the common methods for RNA posttranscription modification. It participates in the regulation of various RNA biological processes in cells, including alternative splicing [9], maintaining RNA stability [10] and normal structure [11], protein translation [12], and RNA-protein interactions [13]. Posttranscriptional modifications are installed or removed by enzymatic reactions at specific sites by methyltransferases (writers) and demethyltransferases (erasers), and methylated binding proteins (readers) can read the modified information and act as delivery messengers for the execution of downstream functions [14]. RNA methylations include N1-methyladenosine (m1A), N6-methyladenosine (m6A), and 5-methylcytosine (m5C), among which m5C that is a methyl group attaches to the fifth atom of the cytosine ring which is catalyzed by RNA methyltransferase [15], which is closely related to the activation of protooncogenes. A study has shown that the m5C methyltransferase NSUN2 and the recognition protein YBX1 are highly expressed in BLCA tissues, keeping the protooncogene HDGF at stable high expression, hence the poor prognosis of the patients, indicating that m5C methylation mediated by NSUN2 plays a facilitating role in promoting tumor occurrence and progression in BLCA [16]. In cutaneous squamous cell carcinoma, the reduced rate of protein synthesis may be caused by the low expression of NSUN2, which results in certain stem cell characteristics of tumor initiating cells [17]. In liver cancer, modified m5C H19lncRNA can promote tumor occurrence and development by recruiting the G3BP1 oncoprotein [18]. In nonsmall cell lung cancer cells, knocking down YBX1 increases the sensitivity of cells to cisplatin by inhibiting autophagy [19]. In breast cancer, TET2 knockout results in an increase in m5C of most enhancers and a significant reduction in of H3K4me1 and H3K27ac enrichment, which jointly promoted the tumorigenesis of ER*α*-positive MCF7 breast cancer cells [20].

Long noncoding RNAs (lncRNAs) are the main noncoding RNAs with transcripts longer than 200 nt, which are involved in the regulation of RNA methylation and can be used to evaluate tumor prognosis [21]. lncRNAs are closely related to the prognosis of patients with BLCA; therefore, it is crucial to identify RNA methylation-associated lncRNAs with a definitive prognostic value [22]. In addition, lncRNAs also regulate the tumor microenvironment (TME), influence tumor growth, and metastasis [23, 24]. Previous studies have demonstrated the widespread presence of modified cytosines throughout coding and noncoding sequences in a transcriptome, suggesting a broader role of this modification in the posttranscriptional control of cellular RNA function [25]. Unfortunately, there are few published data on the regulation of m5C-related lncRNAs in BLCA.

**Figure 1:**
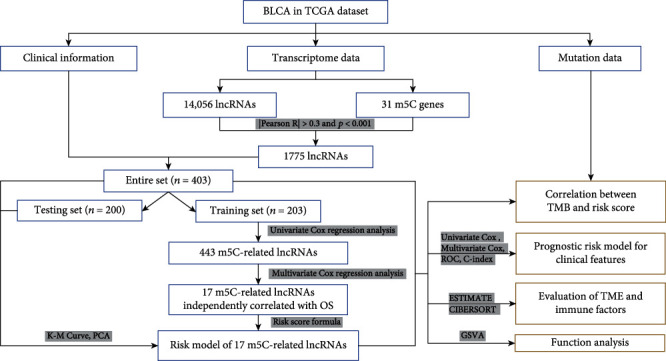
Flowchart for constructing and evaluating prognostic risk model.

In this study, the expression profiles of 14,056 lncRNAs and 31 m5C genes were abstracted from The Cancer Genome Atlas (TCGA) dataset. We identified m5C-associated lncRNAs through Pearson's correlation analysis. Using univariate Cox regression, Lasso analysis, and multivariate Cox regression, a prognosis-related risk model was constructed to predict the prognostic characteristics of patients with BLCA. The tumor microenvironment, immune infiltration, and tumor mutations of patient group based on the risk model were studied to provide a theoretical research basis for discovering BLCA biomarkers and immunotherapy targets. The workflow for the construction of 5mC-lncRNA clusters and subsequent analysis is shown in Figure 1.

## 2. Materials and Methods

### 2.1. Data Acquisition and m5C-Related lncRNAs Identification in BLCA

RNA sequence transcriptome data, clinical information, and mutation data of BLCA were downloaded from The Cancer Genome Atlas (TCGA) (https://portal.gdc.cancer.gov/) database and patients with missing OS values were excluded. lncRNA annotation file of Genome Reference Consortium Human Build 38 (GRCH38) data was acquired from Ensembl database (https://asia.ensembl.org) to annotate genome names and to screen for mRNA and lncRNA. Based on the published literature, a total of 31 m5C regulators were collected, comprising 10 writers (NSUN2, NSUN3, NSUN4, NSUN5, NSUN6, NSUN7, DNMT1, DNMT3A, DNMT3B, and TRDMT1), 4 erasers (TET1, TET2, TET3, and TDG), and 17 readers (ALYREF, YBX1, RAD52, MBD1, MBD2, MBD3, MBD4, MECP2, NEIL1, NTHL1, SMUG1, UHRF1, UHRF2, UNG, ZBTB33, ZBTB38, and ZBTB4) [26–28]. Pearson's correlation analysis was applied to obtain m5C-related lncRNA by the standard correlation coefficient (∣cor∣) > 0.3 and *p* < 0.001.

### 2.2. Construction and Verification of m5C-Related lncRNA Risk Model

In order to identify m5C-related lncRNAs with prognostic value, the entire TCGA set was randomized into two sets named training and testing. The training set is used to construct an m5C-related lncRNA risk model and the testing set and the entire set to validate it. Then, we performed a univariate Cox regression on the basis of the standard of *p* < 0.01. Lasso analysis and multivariate Cox regression were applied to establish a risk score. Calculating coefficients (coef) of m5C-related lncRNAs correlated with survival through multivariate Cox regression, each expression and coef of m5C-related lncRNAs were used to develop a formula: Risk score = coef (lncRNA1 ~ *n*) × expr (lncRNA1 ~ *n*). Subsequently, according to the median risk score, all patients with BLCA were divided into two subgroups (low-risk and high-risk groups).

### 2.3. Independence Prognostic Factor of the m5C-Related lncRNA Risk Model

Expressions that included the whole genome, m5C-related coding genes, and m5C-related lncRNAs of the risk model were carried out by principal component analysis (PCA). The Kaplan-Meier (KM) survival curve was used to compare OS of patients in the low-risk and high-risk groups. Univariate and multivariate Cox regression analyses were performed to evaluate whether prognostic pattern was independent of the other clinical variables (age, gender, grade, and TNM stage), and the receiver operating characteristic (ROC) curve and concordance index (C-index) were executed to appraise the prognostic value of clinicopathological characteristics. In light of independent prognostic factors, the predictive capacity of the nomogram was constructed and calibrated to make clinical diagnosis and treatment decisions in the risk model.

### 2.4. Function Enrichment Analysis

To further acquaint the biological pathways of gene sets in low- and high-risk groups, Gene Set Variation Analysis (GSVA) was applied to assess the significant gene sets in the BLCA cohort with ∣logFC | >0.1 and *p* value < 0.05.

### 2.5. Analysis of Immune Cell Characteristics

ESTIMATE was applied to estimate stromal and immune cells in malignant tumor tissues based on expression data and calculate the stromal score and the immune score of each sample [29]. LM22 [30], a gene signature matrix annotation for 22 immune cells, used to quantify the infiltration of immune cell components, and the CIBERSORT R script were acquired from the CIBERSORT website (http://cibersort.stanford.edu/). Subsequently, the relative proportions of 22 immune cells in patients with BLCA were calculated by 100 permutations of the default signature matrix and the root mean square error for each sample file. Finally, we screened the differentially infiltrated immune cells across the low- and high-risk groups and investigated whether there was a correlation with risk grade.

### 2.6. Calculation of Tumor Mutation Burden (TMB)

Summing the mutation data, the TMB was evaluated on the basis of tumor-specific mutated genes. The KM curve was used to map the survival of patients in the high and low tumor mutation groups.

### 2.7. Statistical Analysis

The research was done using the R package. The Kruskal-Wallis or Wilcoxon test was used for comparing the differential expression between different groups. The KM was used to estimate survival and all survival curves tested by log-rank test. Statistical significance was established at *p* < 0.05. Pearson's correlation coefficient was used to determine the correlation between lncRNAs and immune cells.

## 3. Results

### 3.1. 1775 lncRNAs Were Related to 31 m5C Genes in Patients with BLCA

Transcriptome data of 430 patients with BLCA were downloaded from the TCGA database, including 411 tumor tissues and 19 normal tissues, of which 409 cases contained complete clinical information shown in Table 1. A total of 14,056 lncRNAs and 31 m5C gene expression levels were extracted. Then, we defined 1775 lncRNAs related to each of the 31 m5C genes significantly (∣Pearson R | >0.3 and *p* < 0.001) (Table s1).

### 3.2. 17 m5C-Related lncRNAs Were Used to Construct a Risk Model

**Figure 2:**
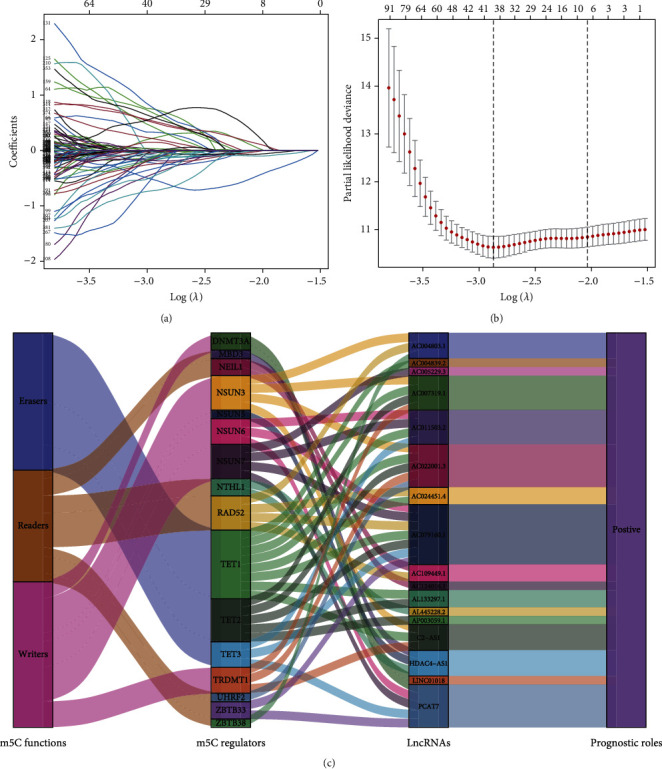
Risk model based on cancer-specific m5C-related lncRNAs in BLCA. (a) The LASSO coefficient profile of 443 OS-related lncRNAs and perpendicular imaginary line were drawn at the value chosen by 10-fold cross-validation. (b) The tuning parameters (log *λ*) of OS-related lncRNAs were selected to cross-verify the error curve. (c) The Cox regression analysis were used to screen coexpression networks of the 17 m5C-lncRNAs.

**Figure 3:**
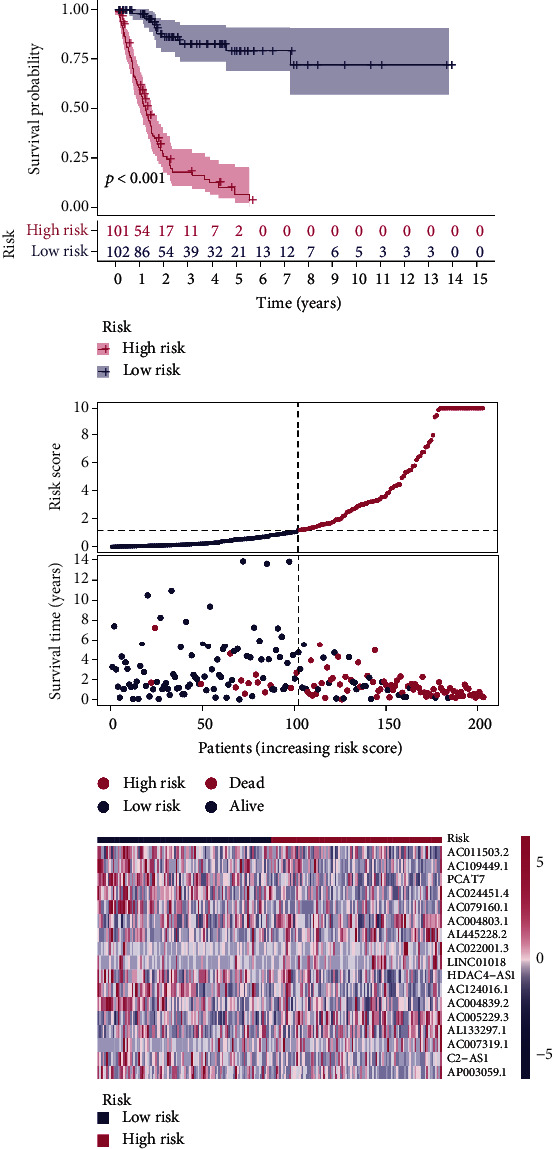
The distribution of risk scores and survival status based on the prognostic value of the risk model of 17 m5C-related lncRNAs in the training set.

**Figure 4:**
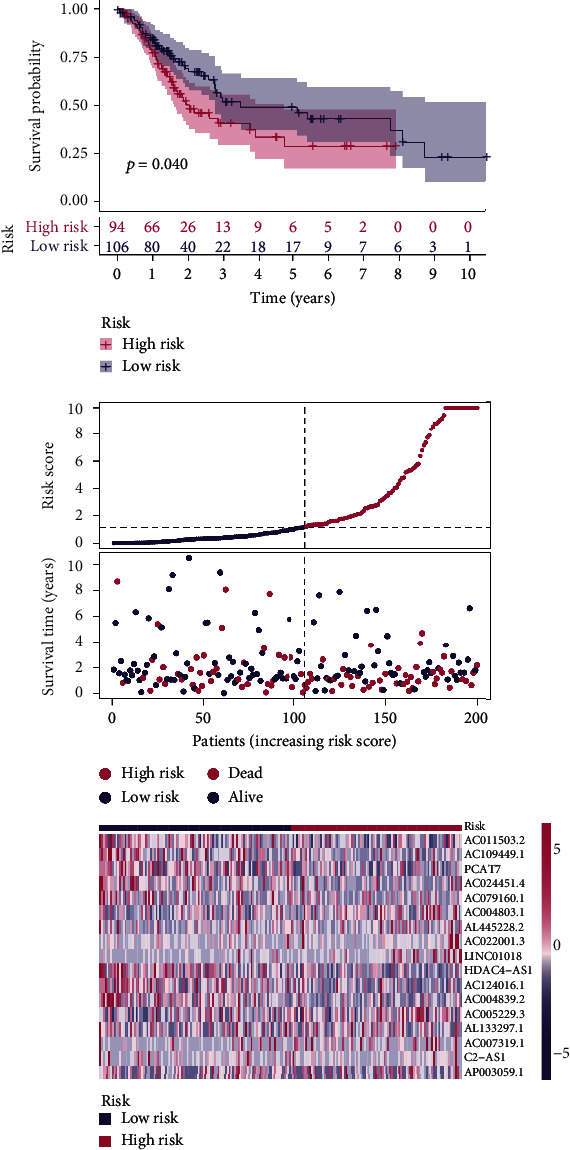
The distribution of risk scores and survival status based on the prognostic value of the risk model of 17 m5C-related lncRNAs in the testing set.

**Figure 5:**
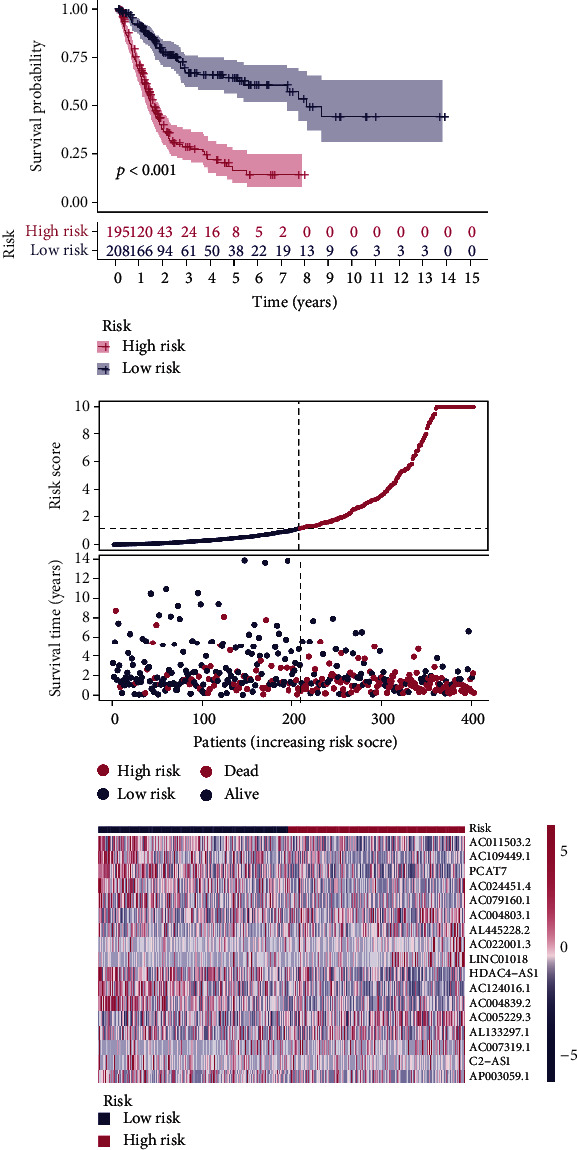
The distribution of risk scores and survival status based on the prognostic value of the risk model of 17 m5C-related lncRNAs in the entire set.

**Figure 6:**
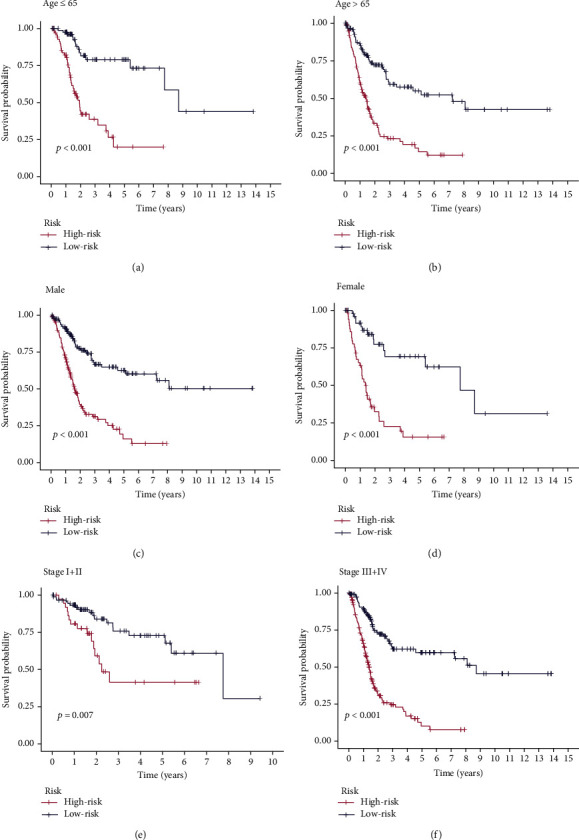
Kaplan-Meier curves of OS differences stratified by clinical characteristics between the high- and low-risk groups in the entire set. (a and b) Kaplan-Meier curve showing that the OS of the low-risk group was higher than that of the high-risk group in patients both ≤65 and >65 years. (c and d) Kaplan-Meier curve showing that the OS of the low-risk group was higher than that of the high-risk group in male and female patients. (e and f) Kaplan-Meier curve showing that the OS of the low-risk group was higher than that of the high-risk group in stage I+II and stage III+IV.

Using univariate Cox regression analysis, the correlation between 443 m5C-related lncRNAs and OS was obvious in the training set (*p* < 0.05) (Table s2). In order to discern the most available forecast markers and prognostic indicators, 17 m5C-related lncRNAs independently correlated with OS were obtained from Lasso and multivariate Cox regression analysis, and a risk model was constructed to predict clinical results (Figures 2(a)–2(c)). Based on the risk score formula and the median risk value, the training set samples were categorized into low- and high-risk groups. The KM survival curves showed that patients in the low-risk group had longer OS than in the high-risk group (*p* < 0.001), as well as the distribution of risk grades, the corresponding survival status, and the relative expression levels of the 17 m5C-related lncRNAs suggested that the high risk index was accompanied by high mortality(Figure 3). To inspect the prognostic capacity of this risk model, the risk scores of the patients in the testing set and the entire set were calculated by means of the uniform formula. Figures 4 and 5 demonstrated that there is no significance of survival rate among the training set, the testing set, and the entire set. The OS of BLCA patients with higher risk scores was shorter than that of those with lower scores. Then, we verified that there is no difference in clinicopathologic characteristics among the three sets (Table 2). According to the subgroups classified by age, gender, and tumor stage, the OS of the high-risk group continued to be worse compared to the low-risk group (Figures 6(a)–6(f)).

**Figure 7:**
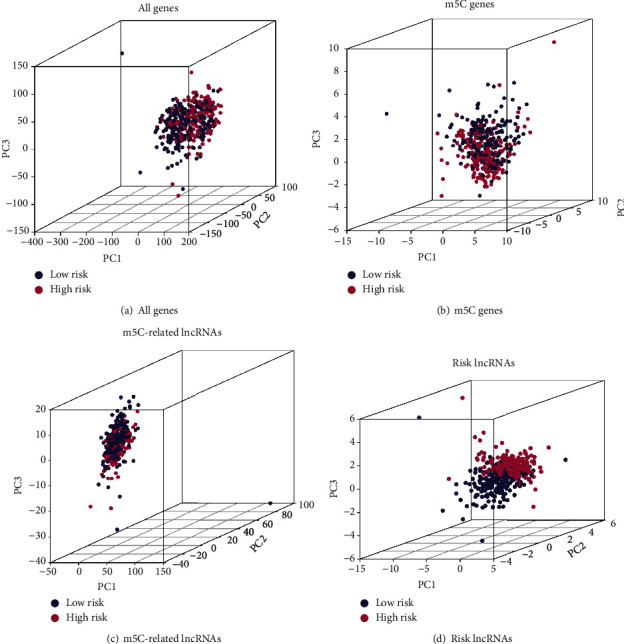
Principal component analysis (PCA) between low- and high-risk groups based on the signature of (a) all genes, (b) m5C genes, (c) m5C-related lncRNAs, and (d) risk genes.

Additionally, PCA was used to test the different distribution patterns of m5C between low- and high-risk groups on the entire gene expression profile, the 31 m5C gene expression profile, the m5C-related lncRNAs expression profile, and the m5C-related lncRNAs expression profile in the risk model (Figures 7(a)–7(d)). Patients in the two risk groups classified by 17 m5C-related lncRNAs were significantly distributed in different directions, whereas the distribution of patients grouped in the other 3 methods was relatively scattered, showing that the low- and high-risk groups can be distinguished by the prognostic signature.

### 3.3. The Risk Model Was an Independent Prognosis Compared to the Other Clinical Features

**Figure 8:**
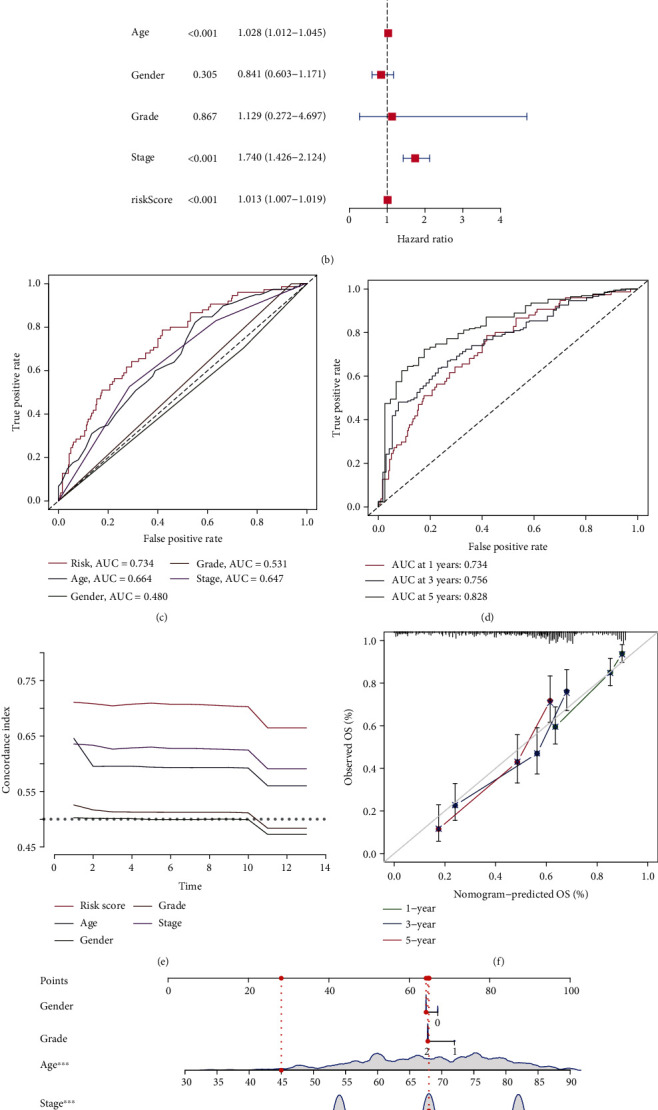
Evaluation of the prognostic risk model of the m5C-related lncRNAs and clinical characteristics in the entire set. (a and b) Univariate and multivariate Cox regression analyses of risk scores combined with clinical characteristics. (c) ROC curves of the clinical characteristics and risk score. (d) ROC curves of the risk score at 1, 3, and 5 years. (e) C-index curves of the risk score and clinical characteristics. (f and g) The nomogram predicting the probability of the 1-, 3-, and 5-year OS.

Univariate and multivariate Cox regression analysis revealed that age (HR = 1.034 and 1.028, respectively, 95% CI: 1.018–1.050 and 1.012–1.045, respectively; *p* < 0.001), pathological stage (HR = 1.760 and 1.740, respectively, 95% CI: 1.451–2.136 and 1.426–2.124, respectively; *p* < 0.001), and risk scores (HR = 1.013 and 1.013, respectively, 95% CI: 1.008–1.019 and 1.007–1.019, respectively; *p* < 0.001) were unrelated to other clinicopathological parameters, including gender and tumor grade while being independent prognostic factors (Figures 8(a) and 8(b)). To verify the specificity and sensitivity of these important prognostic factors, the area under the ROC curve (AUC) and the conformance index (C-index) of the risk score were assessed. We found that the AUC of the risk score was the highest value in all prognostic factors of this test (AUC = 0.734), and at 1, 3, and 5, years it was 0.734, 0.756, and 0.828, respectively (Figures 8(c) and 8(d)). Similarly, the C-index curve proved this outcome (Figure 8(e)). The above results indicated that the risk model of 17 m5C-related lncRNAs was a significant independent prognostic indicator for patients with BLCA. Subsequently, the nomogram comprising the risk score was fabricated to be a quantitative tool to predict the incidences of 1-, 3-, and 5-year OS incidences (Figures 8(f) and 8(g)).

### 3.4. 110 Enrichment Pathways Were Significantly Different between the Two Risk Groups

**Figure 9:**
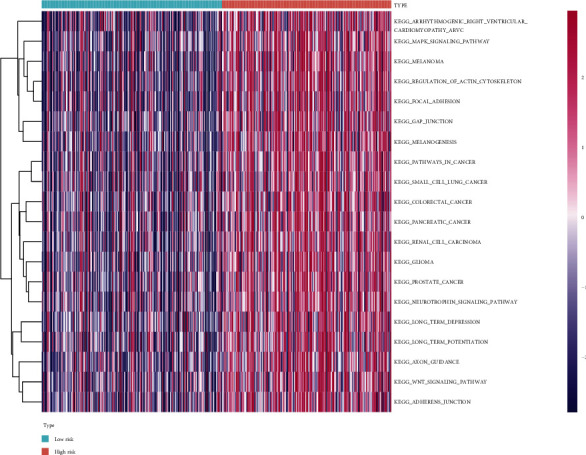
Gene set variation analysis (GSVA) of m5C-related lncRNAs.

According to the GSVA method based on the transcriptome dataset, a total of 174 pathways were obtained, including signaling pathways, metabolic pathways, immune-related pathways, cell function-related pathways, and cancer-related pathways. Then, 110 enrichment pathways with significant differences (Table s3) were analyzed in the low- and high-risk groups. The heatmap showed the results of the first 20 GSVA (Figure 9). Abnormal activation of the Wnt/*β*-catenin signaling pathway, gap junction, focal adhesion transformation, etc., may be characteristics of tumor cells in patients with high risk of BLCA.

### 3.5. The High-Risk Group Got a Higher ESTIMATE Score Compared to the Low-Risk Group

**Figure 10:**
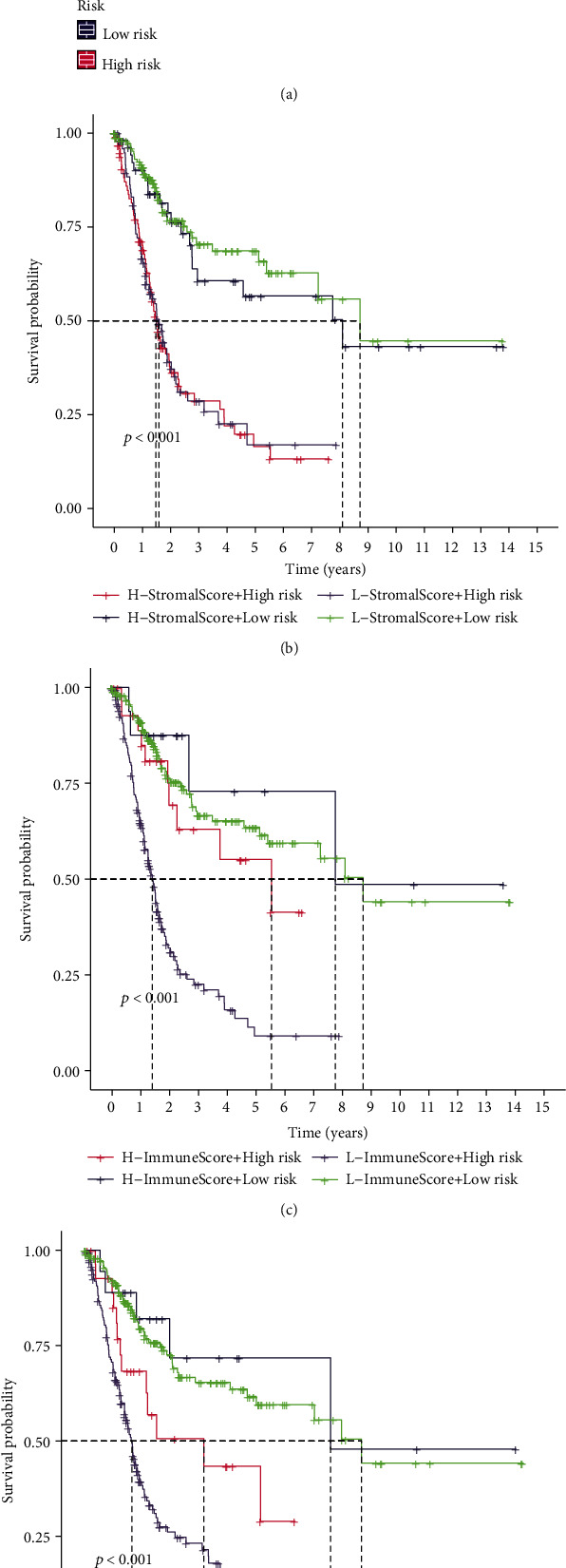
The tumor microenvironment (TME) between low- and high-risk groups in BLCA. (a) Violin plot of TME scores in low- and high-risk groups (^∗∗^*p* < 0.01 and ^∗∗∗^*p* < 0.001). (b–d) Kaplan-Meier curves of low- and high-risk groups showing survival probability with stromal score, immune score, and ESTIMATE score.

In view of the results of function enrichment, we conjectured that the tumor microenvironment (TME) of BLCA is affected by m5C-related lncRNAs. Then, the ESTIMATE algorithm was used to estimate the purity of tumor tissue in patients with BLCA. Stromal score, immune score, and ESTIMATE score were higher in the high-risk group (Figure 10(a)), indicating that the higher the number of immune cells and stromal cells in the risk groups, the lower the survival probability (Figure 10(b)).

### 3.6. Regulatory T Cells, CD8 T Cells, Plasma Cells, and Activated Dendritic Cells Showed Good Prognosis with a High Expression

**Figure 11:**
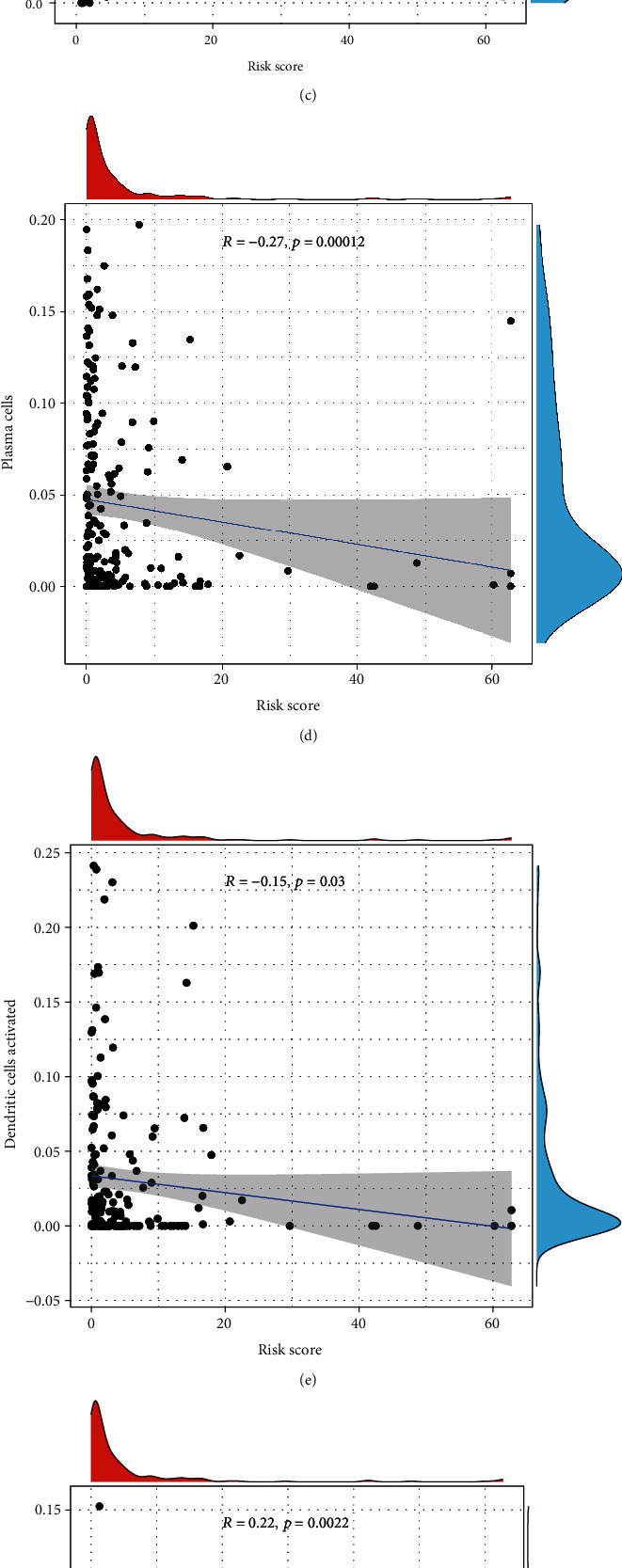
Correlation between tumor-infiltrating immune cells and risk model. (a) Immune infiltration of 22 immune cells in low- and high-risk groups (^∗^*p* < 0.05, ^∗∗^*p* < 0.01, and ^∗∗∗^*p* < 0.001). (b–h) Correlation of risk score with 7 tumor-infiltrating immune cell subtypes: regulatory T cells (b), CD 8 T cells (c), plasma cells (d), activated dendritic cells (e), neutrophils (f), M0 macrophage (g), and M2 macrophage (h).

**Figure 12:**
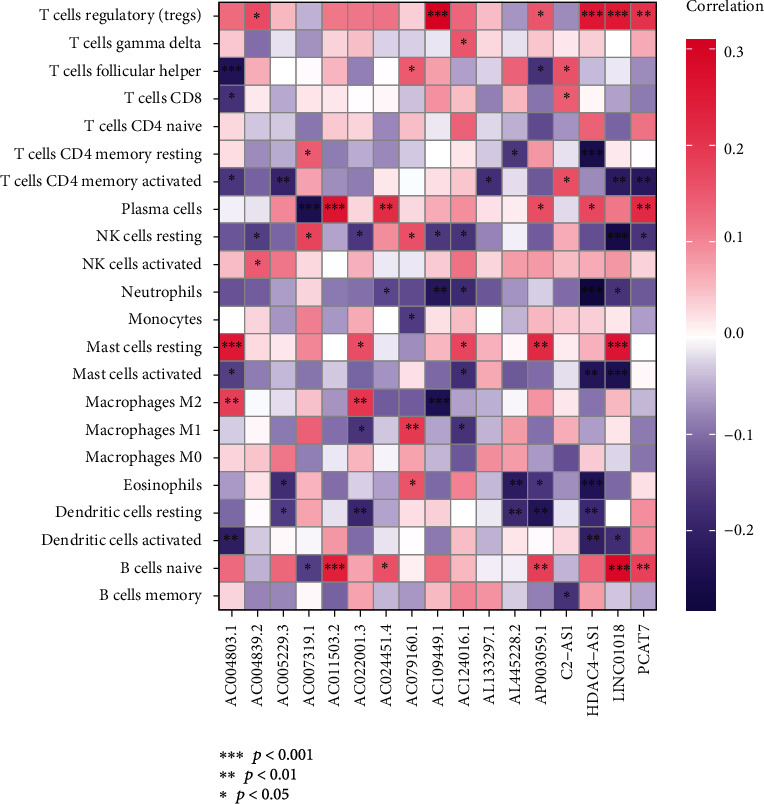
Heatmap of correlation between 17 m5C-related lncRNAs and 22 immune cells (^∗^*p* < 0.05, ^∗∗^*p* < 0.01, and ^∗∗∗^*p* < 0.001).

Using the CIBERSORT tool in the two risk groups, we found that the expression levels of immune cells were higher in the low-risk group including plasma cells, regulatory T cells, CD 4 T cells, gamma delta T cells, monocytes, CD 8 T cells, and activated dendritic cells; however, resting CD4 memory T cells, M0 macrophage, M1 macrophage, M2 macrophage, and neutrophils had lower expression levels than in the high-risk group (Figure 11(a)). Among them, regulatory T cells, CD8 T cells, plasma cells, and activated dendritic cells predicted a good prognosis in high proportion (*R* = −0.24, −0.14, −0.27, and − 0.15, respectively; *p* < 0.05), while neutrophils, macrophage M0, and macrophage M2 showed the opposite results (*R* = 0.22, 0.17, and 0.25, respectively; *p* < 0.05) (Figures 11(b)–11(h)). Simultaneously, the correlation between 17 m5C-related lncRNAs and immune cells is displayed in Figure 12.

### 3.7. The TMB Was Negatively Related to the Risk Score

**Figure 13:**
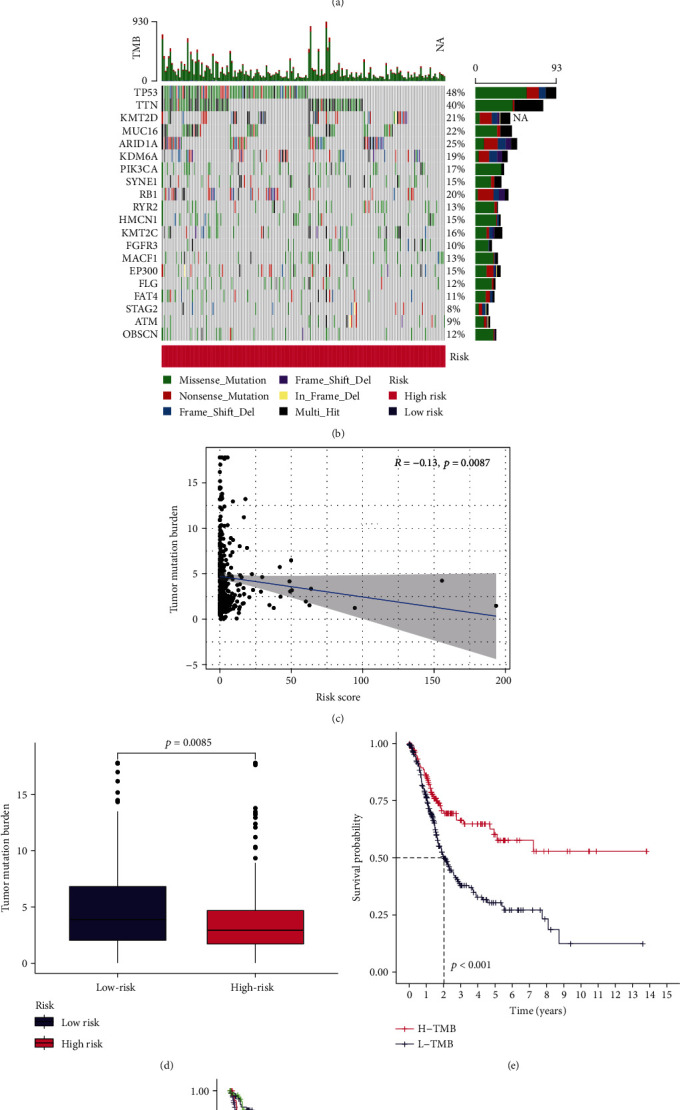
Evaluation of TMB based on tumor-specific mutated genes. (a and b) Gene mutation frequencies in the low-risk group (a) and high-risk group (b). (c and d) Correlation between risk score and TMB in risk groups. (e and f) Kaplan-Meier curves showing survival probability in low- and high-TMB patients.

Analyzing the mutation data, the top 20 driver genes with high alteration frequency between the low- and high-risk groups are shown in Figures 13(a) and 13(b). TP53 and TTN had higher mutations in tumor samples of BLCA. We also found that the correlation between TMB and risk score was negative (Figure 13(c)). The TMB in the low-risk group significantly exceeded that in the high-risk group (Figure 13(d)), as well as the higher the TMB of patients in the risk groups, the lower the mortality rate (Figures 13(e) and 13(f)).

## 4. Discussion

BLCA has attracted the public attention due to its high morbidity and mortality. In addition to traditional treatment methods such as surgery and chemotherapy, more and more attention has been paid to the precise diagnosis and treatment in recent years. Continuous clarification of the tumorigenesis and development mechanism and the discovery and investigation of therapeutic targets are conducive to the formulation of more precise treatment plans and provide a reliable basis for the precise prognosis of BLCA. There are more than 150 types of RNA modification methods reported, among which m1A, m6A, m5C, etc., are the most common and play an important role in the occurrence and development of tumors. Guo et al. has found that YTHDF1 regulates the translation of eIF3C in an m6A-dependent manner, enhances protein synthesis, and promotes tumorigenesis of ovarian cancer cells [31]. The study by Whongsiri et al. [32] found that m5C was decreased in BLCA tissues, but the expression of 8-OHdG, H3K9me3, and HP1*α* increased, indicating that the bladder tissues of BLCA patients have overall DNA hypomethylation, increased oxidative stress, and inhibitory chromatin changes.

The abnormal expression of lncRNA plays an important role in promoting tumor epithelial-mesenchymal cell transformation, tumor cell proliferation, regulating tumor-immune microenvironment, etc., to change the process of tumorigenesis and development. lncRNA RP11-390F4.3 is induced by hypoxia/HIF-1*α* and is essential for hypoxia-induced EMT and metastasis by activating multiple EMT regulators [33]. Suppressing the expression of lncRNA-H19 can reduce the invasive behavior of glioma cells [34]. GAS8-AS1 overexpression inhibits GBM cell proliferation and invasion by downregulating NEAT1 and achieves the purpose of inhibiting the proliferation of glioblastoma cells [35]. SATB2-AS1 binds directly to WDR5 and GADD45A, cis-activating SATB2 (special AT-rich binding protein 2) transcription by mediating histone H3 lysine 4 trimethylation (H3K4me3) deposition and DNA demethylation of the promoter region of SATB2, thus suppressing tumor metastasis and affects the microenvironment of colorectal cancer tumor-immune cells [36]. With the continuous deepening of lncRNA research, there have been more and more studies on lncRNA methylation modification in recent years. Li et al. [37] investigated the role and mechanism of m6A modification of lncRNA KCNQ1 overlapping transcript 1 (KCNQ1OT1) in the progression of laryngeal squamous cell carcinoma (LSCC), suggesting that ALKBH5-mediated m6A modification of KCNQ1OT1 is triggered by the upregulation of HOXA9 LSCC development. Liu et al. [38] identified the differentially expressed lncRNA in gastric cancer and found that the m6A modification of THAP7-AS1 by METTL3 enhanced its expression, and its high expression was associated with positive lymph node metastases and a poor prognosis in patients with gastric cancer. Bo et al. [39] found that ILF3-AS1 increased the level of ILF3 m6A by recruiting the N6-methyladenosine (m6A) RNA methyltransferase METL3. Yan et al. [40] observed that FOXC2-AS1 recruits the RNA methyltransferase NSUN2 to FOXC2 mRNA, increases its level of m5C, and associates with YBX1, indicating that FOXC2-AS1 acts as an oncogenic lncRNA by stabilizing FOXC2 mRNA in an m5C-dependent manner. However, there are currently few reports on the study of m5C-related lncRNA.

In this study, 430 cases of BLCA tissue data were extracted by TCGA. After univariate Cox and multivariate Cox analysis, 17 m5C-related lncRNAs independently related to OS were screened and a risk model was established for m5C-related lncRNAs. The 4 analysis, univariate Cox, multivariate Cox, C-index, and ROC curve, confirmed the risk model with good prognostic and predictive values that could be independent of other clinical characteristics. At the same time, nomograms for quantitatively predicting the prognosis of patients at 1, 3, and 5 years were also drawn. Of the 174 pathways included in the GSVA analysis, 110 were significantly different in the high-risk and low-risk groups. We believe that the abnormal activation of the Wnt/*β*-catenin signaling pathway, the transformation of focal adhesions, and gap junctions may be the characteristics of tumor cells in the high-risk group of BLCA and may affect the tumor microenvironment. It has been confirmed that Wnt/*β*-catenin signaling and TCF1 are highly activated and expressed in undifferentiated CD8+ T and memory CD8+ T cells and that TCF1 is negatively regulated when naive CD8+ T cells differentiate into effector CD8+ T cells [41]. FAK has catalytic activity in cancer cells, and its cellular localization regulates the transcription of chemokines, and these chemokines promote a favorable tumor microenvironment by inhibiting destructive host immunity. FAK activity in TME cells may also increase angiogenesis and vascular permeability [42]. After the ESTIMATE score, the high-risk group showed a poor prognosis with a higher score. The CIBERSORT score showed that the expression levels of regulatory T cells, CD8 T cells, plasma cells, and activated dendritic cells in immune cells were negatively correlated with the prognostic results. Neutrophils, M0 macrophages, and M2 macrophages were negatively correlated with the prognosis. The prognosis was positively correlated. By analyzing the mutation data of BLCA patients, TMB was negatively correlated with the mortality of BLCA patients.

17 m5C-related lncRNAs were used to construct risk models. Among them, there are some related studies on PCAT7, LINC01018, and HDAC4-AS1 in tumorigenesis and development. PCAT7 has been shown to induce malignant progression, metastasis, and poor prognosis of breast cancer [43], prostate cancer [44], nonsmall cell lung cancer [45], and nasopharyngeal carcinoma [46]. And LINC01018 shows tumor suppressor effect in acute myeloid leukemia [47] and hepatocellular carcinoma [48]. HDAC4-AS1 regulates HIF-1*α* under hypoxic conditions to inhibit the transcriptional activity of HDAC4 [49], thereby helping to reduce the toxicity of chemotherapeutics and inhibit tumor growth [50].

## 5. Conclusion

Our study provided clues for prognostic prediction in patients with BLCA, which may help furtherly elucidate the process of m5C-regulated lncRNAs. Moreover, the risk model for BLCA was translated into a nomogram, providing a quantitative and convenient prognostic prediction tool for clinicians, which possibly improves the ability to individualize treatment for patients with BLCA.

## Figures and Tables

**Table 1:** Basic clinical information of 409 cases in patients with BLCA.

Clinical features	The number of patients	Percentage (%)
Age		
>65	248	60.64
≤65	161	39.36
Gender		
Male	303	74.08
Female	106	25.92
Grade		
Low	21	5.13
High	385	94.13
Unknown	3	0.73
Pathological stage		
I	2	0.49
II	130	31.78
III	139	33.99
IV	136	33.25
Unknown	2	0.49
T stage		
T0	1	0.24
T1	3	0.73
T2	120	29.34
T3	194	47.43
T4	59	14.43
Unknown	32	7.82
M stage		
M0	194	47.43
M1	11	2.69
Unknown	204	49.88
N stage		
N0	237	57.95
N1	47	11.49
N2	76	18.58
Unknown	41	10.02

**Table 2:** Detail of clinical characteristics in different sets.

Covariates	Entire set	Testing set	Train set	P value
Age				0.7456
≤65	159 (39.45%)	81 (40.5%)	78 (38.42%)	
>65	244 (60.55%)	119 (59.5%)	125 (61.58%)	
Gender				0.3189
Female	105 (26.05%)	57 (28.5%)	48 (23.65%)	
Male	298 (73.95%)	143 (71.5%)	155 (76.35%)	
Grade				0.2809
High	380 (94.29%)	190 (95%)	190 (93.6%)	
Low	20 (4.96%)	7 (3.5%)	13 (6.4%)	
Unknown	3 (0.74%)	3 (1.5%)	0 (0%)	
Stage				0.5538
Stage I	2 (0.5%)	1 (0.5%)	1(0.49%)	
Stage II	128 (31.76%)	60 (30%)	68 (33.5%)	
Stage III	138 (34.24%)	75(37.5%)	63(31.03%)	
Stage IV	133 (33%)	62 (31%)	71 (34.98%)	
Unknown	2 (0.5%)	2 (1%)	0 (0%)	
T stage				0.2605
T0	1 (0.25%)	0 (0%)	1 (0.49%)	
T1	3 (0.74%)	1 (0.5%)	2 (0.99%)	
T2	118 (29.28%)	56 (28%)	62 (30.54%)	
T3	191 (47.39%)	92 (46%)	99 (48.77%)	
T4	58 (14.39%)	36 (18%)	22 (10.84%)	
Unknown	32 (7.94%)	15 (7.5%)	17 (8.37%)	
M stage				0.9733
M0	193 (47.89%)	95 (47.5%)	98 (48.28%)	
M1	11 (2.73%)	6 (3%)	5 (2.46%)	
Unknown	199 (49.38%)	99 (49.5%)	100 (49.26%)	
N stage				0.1891
N0	234 (58.06%)	125 (62.5%)	109 (53.69%)	
N1	46 (11.41%)	22 (11%)	24 (11.82%)	
N2	75 (18.61%)	36 (18%)	39 (19.21%)	
N3	7 (1.74%)	1 (0.5%)	6 (2.96%)	
Unknown	41 (10.17%)	16 (8%)	25 (12.32%)	

## Data Availability

All the data in this study are download from public databases as described in the passage. Data could be acquired from TCGA (https://portal.gdc.cancer.gov/) repositories.

## Supplementary Materials

Supplementary 1 Table s1: m5C-related lncRNAs.

Supplementary 2 Table s2: univariate Cox analysis in the training set.

Supplementary 3 Table s3: enrichment pathways with significant differences.
