# Supplementary material for: 5-Methylcytosine-Related Long Noncoding RNAs Are Potential Biomarkers to Predict Overall Survival and Regulate Tumor-Immune Environment in Patients with Bladder Cancer
Source: Dis Markers. 2022 Mar 4;2022:3117359. doi: 10.1155/2022/3117359 (PMC8966750; doi:10.1155/2022/3117359)
Supplement: Supplementary 1 — Table s1: m5C-related lncRNAs. [file 3117359.f1.pdf]

m5C	lncRNA	cor	pvalue	Regulation
NSUN3	AC008115.	0.471806	3.59E-24	postive
NSUN7	AC008115.	0.314575	6.83E-11	postive
TRDMT1	AC008115.	0.334761	3.21E-12	postive
RAD52	AC008115.	0.353619	1.50E-13	postive
MECP2	AC008115.	0.329726	7.04E-12	postive
UHRF2	AC008115.	0.368689	1.11E-14	postive
ZBTB33	AC008115.	0.335655	2.79E-12	postive
ZBTB38	AC008115.	0.318442	3.87E-11	postive
TET1	AC008115.	0.476685	1.05E-24	postive
TET2	AC008115.	0.603291	4.23E-42	postive
TET3	AC008115.	0.37583	3.09E-15	postive
NSUN3	YEATS2-A!	0.372233	5.92E-15	postive
NSUN6	YEATS2-A!	0.337853	1.97E-12	postive
NSUN7	YEATS2-A!	0.300733	4.88E-10	postive
DNMT3A	YEATS2-A!	0.31907	3.53E-11	postive
RAD52	YEATS2-A!	0.377942	2.11E-15	postive
NEIL1	YEATS2-A!	0.317247	4.62E-11	postive
UHRF2	YEATS2-A!	0.323436	1.84E-11	postive
ZBTB33	YEATS2-A!	0.359494	5.53E-14	postive
TET1	YEATS2-A!	0.522294	3.84E-30	postive
TET2	YEATS2-A!	0.601323	9.08E-42	postive
TET3	YEATS2-A!	0.470191	5.37E-24	postive
NSUN7	TMEM147-	0.30859	1.62E-10	postive
DNMT3A	TMEM147-	0.37264	5.50E-15	postive
NEIL1	TMEM147-	0.393936	1.04E-16	postive
NSUN6	AC139100.	0.393557	1.12E-16	postive
NSUN7	AC139100.	0.322398	2.15E-11	postive
NEIL1	AC139100.	0.353643	1.49E-13	postive
TET1	AC139100.	0.346242	5.10E-13	postive
TET2	AC139100.	0.30458	2.86E-10	postive
NSUN3	AL161729.	0.328215	8.88E-12	postive
NSUN5	AC009065.	0.30393	3.13E-10	postive
NSUN6	AC009065.	0.313447	8.05E-11	postive
MBD3	AC009065.	0.342114	9.97E-13	postive
NEIL1	AC009065.	0.514117	4.17E-29	postive
NTHL1	AC009065.	0.411297	3.29E-18	postive
SMUG1	AC009065.	0.350972	2.34E-13	postive
RAD52	AL096701.	0.344697	6.56E-13	postive
NEIL1	AL096701.	0.366375	1.68E-14	postive
NSUN2	AC026740.	0.303789	3.19E-10	postive
NSUN5	AC026740.	0.401797	2.23E-17	postive
MBD3	AC026740.	0.447288	1.30E-21	postive
ZBTB33	AC005993.	0.330439	6.30E-12	postive
TET2	AC005993.	0.353687	1.48E-13	postive
TET3	AC005993.	0.316294	5.31E-11	postive
NSUN6	LINC0210S	0.306621	2.14E-10	postive
NSUN7	LINC0210S	0.320806	2.72E-11	postive
TET1	LINC0210S	0.620441	4.31E-45	postive
TET3	LINC0210S	0.324015	1.68E-11	postive
TET2	LINC0106E	0.477134	9.35E-25	postive
NEIL1	LINC0140Z	0.361085	4.21E-14	postive
NSUN6	AC022165.	0.309726	1.38E-10	postive
UHRF2	AC022165.	0.300127	5.31E-10	postive
ZBTB33	AC022165.	0.308046	1.75E-10	postive
TET1	AC022165.	0.543853	5.18E-33	postive
TET2	AC022165.	0.574624	1.74E-37	postive
TET3	AC022165.	0.358624	6.42E-14	postive

NSUN6	AC006270.	0.33833	1.83E-12	postive
NSUN7	AC006270.	0.306326	2.23E-10	postive
TET1	AC006270.	0.663584	1.67E-53	postive
TET2	AC006270.	0.412055	2.82E-18	postive
TET3	AC006270.	0.336109	2.60E-12	postive
NEIL1	LINC0010€	0.358495	6.57E-14	postive
NSUN3	AC011461.	0.455562	1.88E-22	postive
NSUN6	AC011461.	0.348577	3.47E-13	postive
RAD52	AC011461.	0.374778	3.74E-15	postive
MBD1	AC011461.	0.317172	4.67E-11	postive
MECP2	AC011461.	0.305019	2.68E-10	postive
NEIL1	AC011461.	0.453486	3.07E-22	postive
TET2	AC011461.	0.43004	6.26E-20	postive
NSUN3	FMR1-IT1	0.35889	6.14E-14	postive
NSUN6	FMR1-IT1	0.396677	6.12E-17	postive
NSUN7	FMR1-IT1	0.356505	9.21E-14	postive
RAD52	FMR1-IT1	0.396519	6.31E-17	postive
MBD1	FMR1-IT1	0.326591	1.14E-11	postive
MECP2	FMR1-IT1	0.394637	9.09E-17	postive
NEIL1	FMR1-IT1	0.352021	1.96E-13	postive
ZBTB33	FMR1-IT1	0.386784	4.08E-16	postive
TET1	FMR1-IT1	0.455563	1.88E-22	postive
TET2	FMR1-IT1	0.553761	2.11E-34	postive
TET3	FMR1-IT1	0.439951	6.96E-21	postive
NSUN6	LINC0151ε	0.323212	1.90E-11	postive
NSUN6	PDXDC2P-	0.475323	1.48E-24	postive
NSUN7	PDXDC2P-	0.41012	4.19E-18	postive
RAD52	PDXDC2P-	0.407527	7.09E-18	postive
NEIL1	PDXDC2P-	0.460433	5.87E-23	postive
TET2	PDXDC2P-	0.367266	1.43E-14	postive
TET3	PDXDC2P-	0.340847	1.22E-12	postive
NSUN6	AP003352.	0.367225	1.44E-14	postive
NEIL1	AP003352.	0.422047	3.50E-19	postive
NSUN6	AC068768.	0.421056	4.31E-19	postive
RAD52	AC068768.	0.363041	3.00E-14	postive
NEIL1	AC068768.	0.377358	2.34E-15	postive
TET1	AC068768.	0.467399	1.07E-23	postive
TET2	AC068768.	0.468372	8.44E-24	postive
TET3	AC068768.	0.320705	2.76E-11	postive
NSUN6	AC005828.	0.376846	2.57E-15	postive
NSUN7	AC005828.	0.335863	2.70E-12	postive
TET1	AC005828.	0.603455	3.97E-42	postive
TET2	AC005828.	0.303539	3.30E-10	postive
TET3	AC005828.	0.361891	3.66E-14	postive
NSUN3	AC124283.	0.440205	6.57E-21	postive
UHRF2	AC124283.	0.34312	8.48E-13	postive
TET1	AC124283.	0.544778	3.86E-33	postive
TET2	AC124283.	0.601841	7.43E-42	postive
TET3	AC124283.	0.33792	1.95E-12	postive
NSUN3	AC137932.	0.337975	1.93E-12	postive
NSUN6	AC137932.	0.362453	3.32E-14	postive
RAD52	AC137932.	0.454918	2.19E-22	postive
NEIL1	AC137932.	0.380833	1.24E-15	postive
TET1	AC137932.	0.400096	3.13E-17	postive
TET2	AC137932.	0.546522	2.21E-33	postive
TET3	AC137932.	0.382786	8.62E-16	postive
DNMT3B	AC011453.	0.332224	4.78E-12	postive
TET1	AC011453.	0.415166	1.48E-18	postive

DNMT3A	AC021683.	0.336013	2.64E-12	postive
NSUN6	AC105137.	0.408584	5.72E-18	postive
NSUN7	AC105137.	0.384676	6.06E-16	postive
RAD52	AC105137.	0.342687	9.09E-13	postive
NEIL1	AC105137.	0.536899	4.60E-32	postive
TET1	AC105137.	0.393521	1.13E-16	postive
TET2	AC105137.	0.477006	9.66E-25	postive
TET3	AC105137.	0.320235	2.97E-11	postive
NSUN3	AC004951.	0.362484	3.30E-14	postive
RAD52	AC004951.	0.30138	4.46E-10	postive
UHRF2	AC004951.	0.312142	9.72E-11	postive
TET1	AC004951.	0.580384	2.24E-38	postive
TET2	AC004951.	0.534367	1.01E-31	postive
TET3	AC004951.	0.392465	1.38E-16	postive
RAD52	AP001160.	0.379015	1.73E-15	postive
MBD3	AP001160.	0.354212	1.36E-13	postive
NEIL1	AP001160.	0.453247	3.25E-22	postive
NSUN6	AP001107.	0.36097	4.29E-14	postive
RAD52	AP001107.	0.393725	1.08E-16	postive
NEIL1	AP001107.	0.430931	5.15E-20	postive
TET2	AP001107.	0.433172	3.15E-20	postive
TET1	AC016394.	0.362469	3.31E-14	postive
DNMT3A	Z95115.1	0.334106	3.56E-12	postive
TET1	LINC01545	0.47544	1.44E-24	postive
NSUN6	AC108134.	0.362201	3.47E-14	postive
NEIL1	AC108134.	0.493895	1.16E-26	postive
SMUG1	AC108134.	0.311647	1.04E-10	postive
TET1	LINC02315	0.373512	4.71E-15	postive
NSUN3	AC026202.	0.335932	2.67E-12	postive
UHRF2	AC026202.	0.335918	2.68E-12	postive
TET1	AC026202.	0.471935	3.48E-24	postive
TET2	AC026202.	0.504385	6.56E-28	postive
UHRF2	SCAT1	0.312497	9.24E-11	postive
NSUN6	AC106052.	0.339527	1.51E-12	postive
NSUN7	AC106052.	0.347195	4.36E-13	postive
RAD52	AC106052.	0.30612	2.30E-10	postive
NEIL1	AC106052.	0.434353	2.43E-20	postive
NSUN3	GLYCTK-A	0.450829	5.73E-22	postive
TRDMT1	GLYCTK-A	0.359552	5.48E-14	postive
TET1	GLYCTK-A	0.619942	5.29E-45	postive
TET2	GLYCTK-A	0.439637	7.47E-21	postive
TET3	GLYCTK-A	0.357383	7.94E-14	postive
NSUN3	AL031282.	0.30552	2.50E-10	postive
NSUN6	AL031282.	0.33381	3.73E-12	postive
NSUN7	AL031282.	0.441333	5.09E-21	postive
DNMT3A	AL031282.	0.466858	1.23E-23	postive
RAD52	AL031282.	0.414341	1.76E-18	postive
MBD1	AL031282.	0.328547	8.44E-12	postive
ZBTB33	AL031282.	0.393108	1.22E-16	postive
TET1	AL031282.	0.371664	6.56E-15	postive
TET2	AL031282.	0.392739	1.31E-16	postive
TET3	AL031282.	0.502376	1.15E-27	postive
NSUN5	AP001160.	0.338226	1.86E-12	postive
MBD3	AP001160.	0.35772	7.49E-14	postive
NSUN3	FENDRR	0.557127	6.95E-35	postive
TRDMT1	FENDRR	0.303386	3.38E-10	postive
ZBTB38	FENDRR	0.319174	3.47E-11	postive
NSUN3	AC110609.	0.519686	8.28E-30	postive

TRDMT1	AC110609.	0.352304	1.87E-13	postive
TET1	AC110609.	0.414111	1.84E-18	postive
NSUN6	AC092119.	0.377598	2.24E-15	postive
RAD52	AC092119.	0.400938	2.65E-17	postive
NEIL1	AC092119.	0.571711	4.84E-37	postive
NSUN6	AL117379.	0.39715	5.58E-17	postive
RAD52	AL117379.	0.340112	1.38E-12	postive
NEIL1	AL117379.	0.555972	1.02E-34	postive
NSUN7	AL121906.	0.314439	6.97E-11	postive
NSUN5	RPARP-AS	0.340786	1.23E-12	postive
NSUN6	RPARP-AS	0.331351	5.47E-12	postive
NSUN7	RPARP-AS	0.32365	1.78E-11	postive
RAD52	RPARP-AS	0.3006	4.97E-10	postive
MBD3	RPARP-AS	0.332296	4.73E-12	postive
NEIL1	RPARP-AS	0.450194	6.65E-22	postive
NTHL1	RPARP-AS	0.472857	2.76E-24	postive
SMUG1	RPARP-AS	0.341606	1.08E-12	postive
NSUN3	PART1	0.30499	2.70E-10	postive
ALYREF	AL441992.	0.376564	2.71E-15	postive
NSUN5	SNHG11	0.333592	3.86E-12	postive
MBD3	SNHG11	0.393845	1.06E-16	postive
NEIL1	SNHG11	0.383349	7.76E-16	postive
NTHL1	SNHG11	0.31468	6.73E-11	postive
SMUG1	SNHG11	0.375406	3.34E-15	postive
TET1	DSG2-AS1	0.394693	8.99E-17	postive
NSUN6	AL121583.	0.324724	1.51E-11	postive
RAD52	AL121583.	0.344198	7.12E-13	postive
NEIL1	AL121583.	0.35374	1.47E-13	postive
NSUN5	AL163051.	0.443795	2.91E-21	postive
MBD3	AL163051.	0.403352	1.64E-17	postive
NTHL1	AL163051.	0.536418	5.34E-32	postive
SMUG1	AL163051.	0.353883	1.43E-13	postive
NSUN6	ZNF32-AS	0.528195	6.60E-31	postive
NSUN7	ZNF32-AS	0.434953	2.13E-20	postive
DNMT3A	ZNF32-AS	0.491279	2.34E-26	postive
RAD52	ZNF32-AS	0.466345	1.39E-23	postive
MBD1	ZNF32-AS	0.433399	3.00E-20	postive
MECP2	ZNF32-AS	0.302892	3.62E-10	postive
NEIL1	ZNF32-AS	0.458663	8.98E-23	postive
ZBTB33	ZNF32-AS	0.351922	1.99E-13	postive
TET1	ZNF32-AS	0.30152	4.38E-10	postive
TET2	ZNF32-AS	0.435241	1.99E-20	postive
TET3	ZNF32-AS	0.461094	5.00E-23	postive
TET1	LINC01352	0.375875	3.07E-15	postive
NSUN6	AC010542.	0.300458	5.07E-10	postive
RAD52	AC010542.	0.386392	4.39E-16	postive
NEIL1	AC010542.	0.454509	2.41E-22	postive
NSUN6	C3orf35	0.321111	2.60E-11	postive
RAD52	C3orf35	0.346828	4.63E-13	postive
NEIL1	C3orf35	0.440271	6.47E-21	postive
DNMT3A	AC127024.	0.300872	4.79E-10	postive
RAD52	AC127024.	0.301574	4.34E-10	postive
MBD3	AC127024.	0.31275	8.90E-11	postive
NEIL1	AC127024.	0.412298	2.68E-18	postive
NSUN6	AC110792.	0.374427	3.99E-15	postive
NSUN7	AC110792.	0.341136	1.17E-12	postive
TET1	AC110792.	0.623577	1.17E-45	postive
TET2	AC110792.	0.438399	9.86E-21	postive

NSUN6	AL023284.:	0.320686	2.77E-11	postive
NEIL1	AL023284.:	0.503029	9.56E-28	postive
SMUG1	AL023284.:	0.331609	5.26E-12	postive
ZBTB38	AL023284.:	-0.32651	1.15E-11	negative
DNMT3A	LINC00654	0.421983	3.54E-19	postive
TET1	LINC00654	0.319826	3.15E-11	postive
NSUN6	AC067750.	0.317375	4.53E-11	postive
NSUN7	AC067750.	0.32393	1.70E-11	postive
MECP2	AC067750.	0.323531	1.81E-11	postive
TET1	AC067750.	0.595267	9.22E-41	postive
TET2	AC067750.	0.338352	1.82E-12	postive
NSUN3	AC138393.	0.568858	1.30E-36	postive
TRDMT1	AC138393.	0.357387	7.93E-14	postive
UHRF2	AC138393.	0.358312	6.77E-14	postive
ZBTB33	AC138393.	0.304912	2.72E-10	postive
ZBTB38	AC138393.	0.345592	5.67E-13	postive
TET1	AC138393.	0.595392	8.79E-41	postive
TET2	AC138393.	0.6502	9.52E-51	postive
TET3	AC138393.	0.350497	2.53E-13	postive
NSUN7	AC233280.	0.385232	5.46E-16	postive
NEIL1	AC233280.	0.455679	1.83E-22	postive
NSUN5	KMT2E-AS	0.368848	1.08E-14	postive
MBD3	KMT2E-AS	0.311786	1.02E-10	postive
NEIL1	KMT2E-AS	0.341233	1.15E-12	postive
NTHL1	KMT2E-AS	0.482469	2.37E-25	postive
SMUG1	KMT2E-AS	0.319103	3.51E-11	postive
MBD3	EXOSC10-	0.328013	9.16E-12	postive
ZBTB33	LINC02542	0.306529	2.17E-10	postive
TET1	LINC02542	0.306726	2.11E-10	postive
TET2	LINC02542	0.325875	1.27E-11	postive
TET3	LINC02542	0.316491	5.16E-11	postive
NSUN3	DIP2C-AS:	0.310944	1.16E-10	postive
UHRF2	DIP2C-AS:	0.311602	1.05E-10	postive
TET1	DIP2C-AS:	0.575498	1.28E-37	postive
TET2	DIP2C-AS:	0.564239	6.36E-36	postive
TET3	DIP2C-AS:	0.310366	1.26E-10	postive
TDG	LINC01134	0.328012	9.16E-12	postive
NSUN3	AC004466.	0.344913	6.33E-13	postive
NEIL1	AC004466.	0.318432	3.88E-11	postive
UHRF2	AC004466.	0.301708	4.26E-10	postive
TET1	AC004466.	0.414839	1.59E-18	postive
TET2	AC004466.	0.577914	5.43E-38	postive
TET3	AC004466.	0.343034	8.59E-13	postive
NSUN3	AC134407.	0.453077	3.39E-22	postive
NSUN6	AC134407.	0.333399	3.98E-12	postive
TET1	AC134407.	0.649838	1.13E-50	postive
TET2	AC134407.	0.55882	3.95E-35	postive
TET3	AC134407.	0.369116	1.03E-14	postive
RAD52	SRP14-AS:	0.328344	8.70E-12	postive
DNMT3A	AC002351.	0.30124	4.55E-10	postive
NSUN6	MIOS-DT	0.374185	4.17E-15	postive
NSUN7	MIOS-DT	0.318233	3.99E-11	postive
RAD52	MIOS-DT	0.345051	6.19E-13	postive
NEIL1	MIOS-DT	0.346945	4.54E-13	postive
TET3	MIOS-DT	0.332537	4.55E-12	postive
NEIL1	AC135507.	0.344236	7.07E-13	postive
NSUN3	AC008434.	0.512267	7.09E-29	postive
NSUN7	AC008434.	0.315109	6.32E-11	postive

TRDMT1	AC008434.	0.376438	2.77E-15	postive
MBD1	AC008434.	0.310579	1.22E-10	postive
MECP2	AC008434.	0.339544	1.51E-12	postive
UHRF2	AC008434.	0.375039	3.57E-15	postive
ZBTB33	AC008434.	0.340803	1.23E-12	postive
ZBTB38	AC008434.	0.300531	5.02E-10	postive
TET1	AC008434.	0.43734	1.25E-20	postive
TET2	AC008434.	0.496626	5.55E-27	postive
TET3	AC008434.	0.370832	7.61E-15	postive
MBD3	AL355001.	0.332286	4.73E-12	postive
NSUN6	AC008105.	0.317108	4.71E-11	postive
RAD52	AC008105.	0.348866	3.31E-13	postive
NEIL1	AC008105.	0.500608	1.87E-27	postive
NSUN7	ELOA-AS1	0.321203	2.57E-11	postive
DNMT3A	ELOA-AS1	0.419946	5.45E-19	postive
TET3	ELOA-AS1	0.353043	1.65E-13	postive
NSUN3	NPTN-IT1	0.438024	1.07E-20	postive
UHRF2	NPTN-IT1	0.355845	1.03E-13	postive
ZBTB38	NPTN-IT1	0.325782	1.29E-11	postive
TET1	NPTN-IT1	0.455241	2.03E-22	postive
TET2	NPTN-IT1	0.633979	1.37E-47	postive
TET3	NPTN-IT1	0.314017	7.41E-11	postive
TET2	CERS3-AS:	0.392253	1.44E-16	postive
NSUN3	AC093157.	0.471704	3.68E-24	postive
TET1	AC093157.	0.455077	2.11E-22	postive
TET2	AC093157.	0.350352	2.59E-13	postive
NEIL1	AC027682.	0.432171	3.93E-20	postive
TET2	AC027682.	0.311672	1.04E-10	postive
NSUN3	SSBP3-AS:	0.427502	1.09E-19	postive
NSUN7	SSBP3-AS:	0.370501	8.07E-15	postive
DNMT3A	SSBP3-AS:	0.304568	2.86E-10	postive
TRDMT1	SSBP3-AS:	0.301246	4.55E-10	postive
RAD52	SSBP3-AS:	0.398091	4.64E-17	postive
MECP2	SSBP3-AS:	0.330426	6.32E-12	postive
NEIL1	SSBP3-AS:	0.329616	7.16E-12	postive
UHRF2	SSBP3-AS:	0.318467	3.86E-11	postive
ZBTB33	SSBP3-AS:	0.331246	5.56E-12	postive
TET1	SSBP3-AS:	0.580612	2.06E-38	postive
TET2	SSBP3-AS:	0.57265	3.49E-37	postive
TET3	SSBP3-AS:	0.409591	4.66E-18	postive
NSUN3	WWTR1-IT	0.771602	2.24E-82	postive
TRDMT1	WWTR1-IT	0.458435	9.49E-23	postive
ZBTB38	WWTR1-IT	0.307035	2.02E-10	postive
TET1	WWTR1-IT	0.412503	2.57E-18	postive
TET2	WWTR1-IT	0.430577	5.57E-20	postive
NSUN6	NARF-IT1	0.301136	4.62E-10	postive
NSUN7	NARF-IT1	0.341222	1.15E-12	postive
RAD52	NARF-IT1	0.341808	1.05E-12	postive
NEIL1	NARF-IT1	0.349325	3.07E-13	postive
UHRF2	NARF-IT1	0.301132	4.62E-10	postive
ZBTB33	NARF-IT1	0.348381	3.59E-13	postive
TET1	NARF-IT1	0.452402	3.97E-22	postive
TET2	NARF-IT1	0.576234	9.86E-38	postive
TET3	NARF-IT1	0.451301	5.14E-22	postive
NSUN6	AC007785.	0.34678	4.67E-13	postive
NSUN7	AC007785.	0.304028	3.09E-10	postive
RAD52	AC007785.	0.313578	7.90E-11	postive
TET1	AC007785.	0.430778	5.33E-20	postive

TET3	AC007785.	0.357845	7.34E-14	postive
NSUN3	LINC01194	0.33316	4.13E-12	postive
DNMT3A	LINC01194	0.318929	3.60E-11	postive
TET1	LINC01194	0.547253	1.75E-33	postive
TET3	LINC01194	0.380205	1.39E-15	postive
DNMT3A	THUMPD3	0.305601	2.47E-10	postive
NEIL1	THUMPD3	0.313084	8.48E-11	postive
NSUN3	FTX	0.463798	2.60E-23	postive
TRDMT1	FTX	0.304401	2.93E-10	postive
UHRF2	FTX	0.344695	6.56E-13	postive
ZBTB38	FTX	0.30546	2.52E-10	postive
TET1	FTX	0.645018	1.02E-49	postive
TET2	FTX	0.621171	3.18E-45	postive
TET3	FTX	0.36417	2.46E-14	postive
NSUN4	AL592166.	0.328643	8.31E-12	postive
NSUN7	AL592166.	0.310836	1.17E-10	postive
RAD52	AL592166.	0.304488	2.89E-10	postive
NEIL1	AL592166.	0.312756	8.90E-11	postive
TET1	AL592166.	0.326489	1.16E-11	postive
TET2	AL592166.	0.302603	3.77E-10	postive
NEIL1	AC092809.	0.31457	6.83E-11	postive
DNMT1	AC012313.	0.306682	2.12E-10	postive
NTHL1	MINCR	0.363799	2.63E-14	postive
TET1	NECTIN4-/	0.42105	4.32E-19	postive
TET2	NECTIN4-/	0.495407	7.72E-27	postive
DNMT3A	AL591043.	0.338107	1.89E-12	postive
TET3	AL591043.	0.368382	1.18E-14	postive
NSUN3	AC058791.	0.615432	3.37E-44	postive
TRDMT1	AC058791.	0.414432	1.73E-18	postive
UHRF2	AC058791.	0.356267	9.59E-14	postive
ZBTB38	AC058791.	0.302746	3.69E-10	postive
TET1	AC058791.	0.632611	2.49E-47	postive
TET2	AC058791.	0.569185	1.16E-36	postive
NSUN6	AC126118.	0.334978	3.11E-12	postive
NSUN7	AC126118.	0.307412	1.91E-10	postive
RAD52	AC126118.	0.350514	2.52E-13	postive
NEIL1	AC126118.	0.489863	3.41E-26	postive
NSUN6	AC011503.	0.302939	3.59E-10	postive
NSUN7	AC011503.	0.342021	1.01E-12	postive
TET1	AC011503.	0.460521	5.75E-23	postive
TET3	AC011503.	0.422196	3.39E-19	postive
NSUN7	BDNF-AS	0.37162	6.61E-15	postive
NEIL1	BDNF-AS	0.315456	6.00E-11	postive
NSUN6	AL390719.	0.378905	1.77E-15	postive
RAD52	AL390719.	0.33313	4.15E-12	postive
NEIL1	AL390719.	0.542802	7.24E-33	postive
NSUN6	LINC02626	0.302819	3.65E-10	postive
RAD52	LINC02626	0.45211	4.25E-22	postive
NEIL1	LINC02626	0.430019	6.29E-20	postive
NSUN6	AC008883.	0.306505	2.18E-10	postive
TET1	AC008883.	0.578581	4.28E-38	postive
TET2	AC008883.	0.516894	1.87E-29	postive
TET3	AC008883.	0.376056	2.97E-15	postive
MBD3	AC137630.	0.389305	2.53E-16	postive
NEIL1	AC137630.	0.329113	7.73E-12	postive
MBD3	AC132872.	0.309695	1.38E-10	postive
NEIL1	AC132872.	0.390025	2.21E-16	postive
NTHL1	AC132872.	0.380921	1.22E-15	postive

DNMT3A	LINC0156(	0.45946	7.42E-23	postive
ZBTB33	LINC0156(	0.328161	8.95E-12	postive
TET1	LINC0156(	0.338025	1.92E-12	postive
TET3	LINC0156(	0.379841	1.49E-15	postive
NSUN3	POU6F2-A	0.357267	8.09E-14	postive
TET2	POU6F2-A	0.334029	3.61E-12	postive
TET3	POU6F2-A	0.327799	9.46E-12	postive
SMUG1	AC004241.	0.336621	2.40E-12	postive
NTHL1	AL138762.	0.302714	3.71E-10	postive
NSUN3	AC127024.	0.526785	1.01E-30	postive
NSUN6	AC127024.	0.318811	3.66E-11	postive
NSUN7	AC127024.	0.362853	3.10E-14	postive
TRDMT1	AC127024.	0.31086	1.17E-10	postive
RAD52	AC127024.	0.387462	3.59E-16	postive
MECP2	AC127024.	0.335683	2.78E-12	postive
NEIL1	AC127024.	0.352184	1.91E-13	postive
UHRF2	AC127024.	0.337595	2.05E-12	postive
ZBTB33	AC127024.	0.301567	4.35E-10	postive
TET1	AC127024.	0.57611	1.03E-37	postive
TET2	AC127024.	0.61213	1.28E-43	postive
TET3	AC127024.	0.416791	1.06E-18	postive
MBD4	AL589765.	0.342894	8.79E-13	postive
NSUN7	AC087742.	0.359875	5.18E-14	postive
RAD52	AC087742.	0.321896	2.31E-11	postive
NSUN3	AC024267.	0.549697	7.95E-34	postive
TRDMT1	AC024267.	0.35897	6.05E-14	postive
UHRF2	AC024267.	0.325185	1.41E-11	postive
TET1	AC024267.	0.665384	6.93E-54	postive
TET2	AC024267.	0.605228	1.98E-42	postive
TET3	AC024267.	0.37211	6.05E-15	postive
NSUN6	IQCH-AS1	0.487267	6.78E-26	postive
NSUN7	IQCH-AS1	0.509182	1.71E-28	postive
RAD52	IQCH-AS1	0.361562	3.88E-14	postive
NEIL1	IQCH-AS1	0.489795	3.47E-26	postive
SMUG1	IQCH-AS1	0.309104	1.50E-10	postive
TET2	IQCH-AS1	0.355226	1.14E-13	postive
NSUN3	TXNDC12-	0.513824	4.54E-29	postive
TRDMT1	TXNDC12-	0.352587	1.78E-13	postive
UHRF2	TXNDC12-	0.327723	9.57E-12	postive
TET1	TXNDC12-	0.684381	4.42E-58	postive
TET2	TXNDC12-	0.565636	3.95E-36	postive
TET3	TXNDC12-	0.369169	1.02E-14	postive
NSUN3	AC024075.	0.348367	3.60E-13	postive
NSUN4	AC024075.	0.330777	5.98E-12	postive
NSUN6	AC024075.	0.347252	4.32E-13	postive
NSUN7	AC024075.	0.335476	2.87E-12	postive
RAD52	AC024075.	0.359493	5.53E-14	postive
MECP2	AC024075.	0.392307	1.43E-16	postive
NEIL1	AC024075.	0.317581	4.39E-11	postive
ZBTB33	AC024075.	0.413263	2.20E-18	postive
ZBTB38	AC024075.	0.300233	5.23E-10	postive
TET1	AC024075.	0.447647	1.20E-21	postive
TET2	AC024075.	0.560382	2.34E-35	postive
TET3	AC024075.	0.452248	4.11E-22	postive
NSUN6	PAXIP1-D	0.340096	1.38E-12	postive
NSUN7	PAXIP1-D	0.30596	2.35E-10	postive
NEIL1	PAXIP1-D	0.357471	7.82E-14	postive
NSUN3	DLEU2	0.437232	1.28E-20	postive

TET1	DLEU2	0.554438	1.69E-34	postive
TET2	DLEU2	0.471157	4.22E-24	postive
NSUN6	AC011462.	0.399542	3.49E-17	postive
RAD52	AC011462.	0.476996	9.69E-25	postive
NEIL1	AC011462.	0.587394	1.75E-39	postive
NSUN6	AC005041.	0.348087	3.77E-13	postive
NSUN7	AC005041.	0.332734	4.41E-12	postive
DNMT3A	AC005041.	0.396356	6.51E-17	postive
RAD52	AC005041.	0.380634	1.28E-15	postive
NEIL1	AC005041.	0.401069	2.58E-17	postive
MECP2	ZNF710-A	0.438255	1.02E-20	postive
NSUN6	AC008655.	0.416993	1.01E-18	postive
NSUN7	AC008655.	0.316785	4.94E-11	postive
RAD52	AC008655.	0.428317	9.11E-20	postive
NEIL1	AC008655.	0.375096	3.53E-15	postive
NSUN3	AL590729.	0.465584	1.68E-23	postive
NSUN6	AL590729.	0.312806	8.83E-11	postive
NSUN7	AL590729.	0.349248	3.11E-13	postive
TRDMT1	AL590729.	0.331251	5.56E-12	postive
UHRF2	AL590729.	0.328345	8.70E-12	postive
ZBTB33	AL590729.	0.334046	3.60E-12	postive
TET1	AL590729.	0.667493	2.46E-54	postive
TET2	AL590729.	0.561629	1.54E-35	postive
TET3	AL590729.	0.440532	6.10E-21	postive
TET1	AC108704.	0.51645	2.13E-29	postive
NSUN3	AC106037.	0.480269	4.19E-25	postive
NSUN7	AC106037.	0.305008	2.69E-10	postive
TRDMT1	AC106037.	0.352759	1.73E-13	postive
UHRF2	AC106037.	0.316243	5.35E-11	postive
TET1	AC106037.	0.658717	1.74E-52	postive
TET2	AC106037.	0.476182	1.19E-24	postive
TET3	AC106037.	0.378006	2.08E-15	postive
TET1	G2E3-AS1	0.443022	3.47E-21	postive
TET2	G2E3-AS1	0.361246	4.09E-14	postive
TET3	G2E3-AS1	0.313226	8.31E-11	postive
NSUN3	AC025031.	0.30202	4.08E-10	postive
NSUN6	AC025031.	0.337084	2.23E-12	postive
NSUN7	AC025031.	0.310401	1.25E-10	postive
TET1	AC025031.	0.688248	5.64E-59	postive
TET2	AC025031.	0.398535	4.25E-17	postive
TET3	AC025031.	0.348048	3.79E-13	postive
NSUN6	C1orf220	0.46711	1.15E-23	postive
NSUN7	C1orf220	0.42017	5.20E-19	postive
RAD52	C1orf220	0.403679	1.54E-17	postive
MBD1	C1orf220	0.340352	1.32E-12	postive
NEIL1	C1orf220	0.469048	7.14E-24	postive
ZBTB33	C1orf220	0.325246	1.40E-11	postive
TET1	C1orf220	0.359374	5.65E-14	postive
TET2	C1orf220	0.437045	1.34E-20	postive
TET3	C1orf220	0.379733	1.52E-15	postive
NSUN6	AL133255.	0.473582	2.30E-24	postive
NSUN7	AL133255.	0.354795	1.23E-13	postive
RAD52	AL133255.	0.352654	1.76E-13	postive
NEIL1	AL133255.	0.400149	3.10E-17	postive
TET1	AL133255.	0.343083	8.53E-13	postive
TET1	AC022893.	0.405396	1.09E-17	postive
NEIL1	AC011498.	0.307069	2.01E-10	postive
NSUN6	AC126178.	0.432285	3.83E-20	postive

RAD52	AC126178.	0.322261	2.19E-11	postive
NEIL1	AC126178.	0.345091	6.15E-13	postive
NSUN5	IDH1-AS1	0.321249	2.55E-11	postive
NTHL1	IDH1-AS1	0.401123	2.55E-17	postive
NSUN6	AC061975.	0.313497	7.99E-11	postive
RAD52	AC061975.	0.315679	5.81E-11	postive
NEIL1	AC061975.	0.486776	7.71E-26	postive
TET2	AC061975.	0.394559	9.23E-17	postive
NSUN7	AC107419.	0.328011	9.16E-12	postive
TET1	AC107419.	0.669933	7.32E-55	postive
TET2	AC107419.	0.310287	1.27E-10	postive
TET3	AC107419.	0.375319	3.39E-15	postive
NSUN6	AC008870.	0.331807	5.10E-12	postive
NSUN7	AC008870.	0.305558	2.49E-10	postive
RAD52	AC008870.	0.377462	2.30E-15	postive
NEIL1	AC008870.	0.55976	2.89E-35	postive
TET2	AC008870.	0.428542	8.67E-20	postive
NSUN6	AL358072.	0.439851	7.12E-21	postive
NSUN7	AL358072.	0.37657	2.70E-15	postive
RAD52	AL358072.	0.37164	6.59E-15	postive
NEIL1	AL358072.	0.394868	8.69E-17	postive
TET1	AL358072.	0.369641	9.41E-15	postive
NSUN3	AC093484.	0.455867	1.75E-22	postive
TRDMT1	AC093484.	0.315816	5.70E-11	postive
UHRF2	AC093484.	0.380979	1.20E-15	postive
TET1	AC093484.	0.426841	1.25E-19	postive
TET2	AC093484.	0.499171	2.77E-27	postive
NSUN6	AC009806.	0.307168	1.98E-10	postive
NEIL1	AC009806.	0.316041	5.51E-11	postive
TET1	AC009806.	0.305625	2.46E-10	postive
NSUN6	AC080112.	0.402468	1.96E-17	postive
NEIL1	AC124068.	0.357127	8.29E-14	postive
NSUN6	AL078587.	0.384478	6.29E-16	postive
NSUN7	AL078587.	0.3709	7.52E-15	postive
NEIL1	AL078587.	0.348483	3.53E-13	postive
TET1	AL078587.	0.353424	1.55E-13	postive
TET3	AL078587.	0.362452	3.32E-14	postive
TRDMT1	BVES-AS1	0.305212	2.61E-10	postive
TET2	AC084759.	0.468681	7.82E-24	postive
TET1	AC119800.	0.514185	4.09E-29	postive
TET2	AC119800.	0.378444	1.92E-15	postive
MBD3	AC138230.	0.336428	2.47E-12	postive
NEIL1	AC138230.	0.418827	6.90E-19	postive
NSUN6	AC113139.	0.316041	5.51E-11	postive
RAD52	AC113139.	0.314004	7.42E-11	postive
NEIL1	AC113139.	0.316031	5.52E-11	postive
NSUN6	AC010618.	0.305166	2.63E-10	postive
RAD52	AC010618.	0.348911	3.29E-13	postive
NEIL1	AC010618.	0.440897	5.62E-21	postive
TET1	AC010618.	0.313282	8.24E-11	postive
NSUN6	AC103706.	0.393377	1.16E-16	postive
DNMT1	AC103706.	-0.31435	7.06E-11	negative
NEIL1	AC103706.	0.439007	8.60E-21	postive
SMUG1	AC103706.	0.37968	1.53E-15	postive
DNMT3A	AC073842.	0.458004	1.05E-22	postive
RAD52	AC073842.	0.375489	3.29E-15	postive
NEIL1	AC073842.	0.302723	3.70E-10	postive
TET2	AC073842.	0.303559	3.29E-10	postive

TET3	AC073842.	0.385326	5.36E-16	postive
DNMT1	TMPO-AS:	0.47062	4.83E-24	postive
ALYREF	TMPO-AS:	0.361894	3.66E-14	postive
MBD4	TMPO-AS:	0.432195	3.91E-20	postive
TDG	TMPO-AS:	0.338379	1.81E-12	postive
UHRF1	TMPO-AS:	0.510697	1.11E-28	postive
UNG	TMPO-AS:	0.405899	9.84E-18	postive
NSUN5	TYMSOS	0.380074	1.42E-15	postive
ALYREF	TYMSOS	0.358739	6.30E-14	postive
NTHL1	TYMSOS	0.508225	2.24E-28	postive
TET2	MIR4713H	0.431383	4.67E-20	postive
NSUN7	FLVCR1-D	0.341335	1.13E-12	postive
TET2	AC015712.	0.305203	2.62E-10	postive
NSUN3	AP001107.	0.465202	1.84E-23	postive
NSUN6	AP001107.	0.301201	4.58E-10	postive
RAD52	AP001107.	0.347141	4.40E-13	postive
MECP2	AP001107.	0.309059	1.51E-10	postive
NEIL1	AP001107.	0.33183	5.08E-12	postive
ZBTB33	AP001107.	0.316115	5.45E-11	postive
TET1	AP001107.	0.467559	1.03E-23	postive
TET2	AP001107.	0.614906	4.17E-44	postive
TET3	AP001107.	0.457553	1.17E-22	postive
TRDMT1	AL122035.	0.329007	7.86E-12	postive
MECP2	AL122035.	0.346355	5.01E-13	postive
ZBTB38	AL122035.	0.318993	3.57E-11	postive
NSUN3	NOP53-AS	0.304118	3.05E-10	postive
TET1	NOP53-AS	0.321322	2.52E-11	postive
TET2	NOP53-AS	0.474663	1.75E-24	postive
NEIL1	AL121832.	0.32009	3.03E-11	postive
ZBTB33	AL117327.	0.440019	6.85E-21	postive
NSUN6	AC010201.	0.484826	1.29E-25	postive
NSUN7	AC010201.	0.39181	1.57E-16	postive
RAD52	AC010201.	0.413667	2.02E-18	postive
NEIL1	AC010201.	0.578594	4.26E-38	postive
TET2	AC010201.	0.30876	1.58E-10	postive
NSUN6	AC003102.	0.357827	7.36E-14	postive
NSUN7	AC003102.	0.36531	2.02E-14	postive
NEIL1	AC003102.	0.502278	1.18E-27	postive
NSUN6	H3-3A-DT	0.342657	9.13E-13	postive
NSUN7	H3-3A-DT	0.38301	8.27E-16	postive
DNMT3A	H3-3A-DT	0.329306	7.51E-12	postive
RAD52	H3-3A-DT	0.317033	4.76E-11	postive
MECP2	H3-3A-DT	0.348949	3.27E-13	postive
ZBTB33	H3-3A-DT	0.30287	3.63E-10	postive
TET1	H3-3A-DT	0.644856	1.10E-49	postive
TET2	H3-3A-DT	0.451908	4.46E-22	postive
TET3	H3-3A-DT	0.409921	4.36E-18	postive
NSUN6	OSGEPL1-	0.339494	1.52E-12	postive
NSUN7	OSGEPL1-	0.329939	6.81E-12	postive
RAD52	OSGEPL1-	0.313067	8.50E-11	postive
NEIL1	OSGEPL1-	0.517571	1.53E-29	postive
TET1	AL359878.	0.335829	2.72E-12	postive
TET3	AC020661.	0.31006	1.31E-10	postive
NSUN6	MUC20-O	0.306035	2.33E-10	postive
NSUN7	MUC20-O	0.379126	1.69E-15	postive
RAD52	MUC20-O	0.379285	1.65E-15	postive
NEIL1	MUC20-O	0.423904	2.35E-19	postive
TET1	MUC20-O	0.362846	3.10E-14	postive

TET2	MUC20-O	0.403742	1.52E-17	postive
NSUN6	AL031667.	0.349616	2.93E-13	postive
NSUN7	AL031667.	0.34672	4.71E-13	postive
RAD52	AL031667.	0.447493	1.24E-21	postive
NEIL1	AL031667.	0.315675	5.81E-11	postive
SMUG1	AL031667.	0.347105	4.43E-13	postive
TET1	AL031667.	0.439252	8.14E-21	postive
TET2	AL031667.	0.451781	4.59E-22	postive
TET3	AL031667.	0.355971	1.01E-13	postive
NSUN3	RAP2C-AS	0.41063	3.77E-18	postive
TRDMT1	RAP2C-AS	0.406138	9.38E-18	postive
ZBTB33	RAP2C-AS	0.301341	4.49E-10	postive
ZBTB38	RAP2C-AS	0.446581	1.54E-21	postive
TET1	RAP2C-AS	0.492572	1.66E-26	postive
TET2	RAP2C-AS	0.473823	2.16E-24	postive
NTHL1	PPP1R21-I	0.32927	7.55E-12	postive
NSUN3	AC069549.	0.696529	6.17E-61	postive
TRDMT1	AC069549.	0.397938	4.78E-17	postive
TET1	AC069549.	0.378527	1.89E-15	postive
TET2	AC069549.	0.480236	4.23E-25	postive
TET1	TFPI2-DT	0.340145	1.37E-12	postive
NSUN6	AL135999.	0.395591	7.56E-17	postive
NSUN7	AL135999.	0.316472	5.17E-11	postive
RAD52	AL135999.	0.466129	1.47E-23	postive
MBD1	AL135999.	0.354025	1.40E-13	postive
NEIL1	AL135999.	0.486154	9.08E-26	postive
TET2	AL135999.	0.301953	4.12E-10	postive
TET3	AL135999.	0.319934	3.10E-11	postive
NSUN6	AL022328.	0.49833	3.49E-27	postive
RAD52	AL022328.	0.463012	3.14E-23	postive
MBD1	AL022328.	0.381954	1.01E-15	postive
NEIL1	AL022328.	0.642984	2.56E-49	postive
NSUN6	AC011815.	0.35661	9.05E-14	postive
NSUN7	AC011815.	0.324477	1.57E-11	postive
RAD52	AC011815.	0.366665	1.59E-14	postive
MBD1	AC011815.	0.306555	2.16E-10	postive
NEIL1	AC011815.	0.397874	4.84E-17	postive
TET1	AC011815.	0.353334	1.57E-13	postive
TET2	AC011815.	0.369399	9.82E-15	postive
TET3	AC011815.	0.31799	4.14E-11	postive
NSUN6	AL365330.	0.365548	1.94E-14	postive
NSUN7	AL365330.	0.310488	1.23E-10	postive
DNMT3A	AL365330.	0.412573	2.53E-18	postive
RAD52	AL365330.	0.488499	4.90E-26	postive
MBD1	AL365330.	0.38802	3.23E-16	postive
NEIL1	AL365330.	0.532775	1.64E-31	postive
TET3	AL365330.	0.325141	1.42E-11	postive
NEIL1	AP002812.	0.33293	4.28E-12	postive
NSUN6	AC245060.	0.364472	2.34E-14	postive
NSUN7	AC245060.	0.346572	4.83E-13	postive
RAD52	AC245060.	0.379493	1.58E-15	postive
MBD1	AC245060.	0.370043	8.76E-15	postive
MECP2	AC245060.	0.356935	8.56E-14	postive
NEIL1	AC245060.	0.412477	2.58E-18	postive
ZBTB33	AC245060.	0.339661	1.48E-12	postive
TET2	AC245060.	0.41384	1.95E-18	postive
TET3	AC245060.	0.409971	4.32E-18	postive
NSUN3	AC023825.	0.722287	1.72E-67	postive

TRDMT1	AC023825.	0.458625	9.06E-23	postive
UHRF2	AC023825.	0.358196	6.91E-14	postive
ZBTB38	AC023825.	0.332993	4.24E-12	postive
TET1	AC023825.	0.422617	3.10E-19	postive
TET2	AC023825.	0.388983	2.69E-16	postive
NSUN3	AC040169.	0.337026	2.25E-12	postive
TET1	AC040169.	0.457966	1.06E-22	postive
TET2	AC040169.	0.462524	3.54E-23	postive
TET1	AL929236.	0.487792	5.90E-26	postive
TET2	AL929236.	0.341512	1.10E-12	postive
TET3	AL929236.	0.307869	1.79E-10	postive
NSUN6	IGBP1-AS1	0.492373	1.75E-26	postive
NSUN7	IGBP1-AS1	0.314805	6.60E-11	postive
ALYREF	IGBP1-AS1	-0.30949	1.42E-10	negative
RAD52	IGBP1-AS1	0.427342	1.12E-19	postive
MBD1	IGBP1-AS1	0.316219	5.37E-11	postive
NEIL1	IGBP1-AS1	0.541115	1.23E-32	postive
TET2	IGBP1-AS1	0.330285	6.45E-12	postive
DNMT3A	AL133215.	0.348976	3.25E-13	postive
ALYREF	AL133215.	0.326072	1.23E-11	postive
NTHL1	AL133215.	0.343629	7.80E-13	postive
SMUG1	AL133215.	0.336987	2.26E-12	postive
DNMT3A	AC009237.	0.313146	8.41E-11	postive
TET3	AC009237.	0.318393	3.90E-11	postive
SMUG1	AC073508.	0.340901	1.21E-12	postive
NSUN6	LINC00910	0.447445	1.26E-21	postive
NSUN7	LINC00910	0.445569	1.94E-21	postive
RAD52	LINC00910	0.307636	1.85E-10	postive
NEIL1	LINC00910	0.32378	1.74E-11	postive
TET3	LINC00910	0.304669	2.82E-10	postive
NSUN3	BMS1P4	0.360928	4.32E-14	postive
NSUN6	BMS1P4	0.324049	1.67E-11	postive
TET1	BMS1P4	0.608188	6.17E-43	postive
TET2	BMS1P4	0.411695	3.03E-18	postive
NSUN7	AC021087.	0.351171	2.26E-13	postive
DNMT3A	AC021087.	0.304957	2.71E-10	postive
RAD52	AC021087.	0.303911	3.14E-10	postive
TET1	AC021087.	0.555255	1.29E-34	postive
TET2	AC021087.	0.312857	8.77E-11	postive
TET3	AC021087.	0.366173	1.74E-14	postive
NSUN7	AL121906.	0.330585	6.16E-12	postive
DNMT3B	AL121906.	0.341263	1.14E-12	postive
RAD52	AL121906.	0.315702	5.79E-11	postive
TET1	AL121906.	0.313384	8.12E-11	postive
TET3	AL121906.	0.309114	1.50E-10	postive
NSUN3	AC089999.	0.329643	7.13E-12	postive
NSUN6	AC089999.	0.418289	7.73E-19	postive
TET1	AC089999.	0.563576	7.97E-36	postive
TET2	AC089999.	0.425918	1.53E-19	postive
NSUN5	AP002360.	0.39001	2.21E-16	postive
MBD3	AP002360.	0.36887	1.08E-14	postive
NTHL1	AP002360.	0.498337	3.48E-27	postive
SMUG1	AP002360.	0.348838	3.33E-13	postive
NSUN7	AC109460.	0.301164	4.60E-10	postive
RAD52	AC109460.	0.39319	1.20E-16	postive
NEIL1	AC109460.	0.537053	4.39E-32	postive
NSUN2	AC099850.	0.43017	6.09E-20	postive
DNMT1	AC099850.	0.570113	8.44E-37	postive

TRDMT1	AC099850.	0.342935	8.73E-13	postive
ALYREF	AC099850.	0.346911	4.57E-13	postive
MBD4	AC099850.	0.363394	2.82E-14	postive
NEIL1	AC099850.	-0.30408	3.06E-10	negative
TDG	AC099850.	0.338196	1.87E-12	postive
UHRF1	AC099850.	0.555232	1.30E-34	postive
UHRF2	AC099850.	0.415546	1.37E-18	postive
ZBTB33	AC099850.	0.384943	5.76E-16	postive
TET3	AC099850.	0.358409	6.66E-14	postive
NSUN7	AL589765.	0.330768	5.99E-12	postive
NEIL1	AL589765.	0.39679	5.98E-17	postive
NSUN3	AC004771.	0.340548	1.28E-12	postive
NSUN6	AC004771.	0.305436	2.53E-10	postive
UHRF2	AC004771.	0.31275	8.90E-11	postive
TET1	AC004771.	0.529038	5.11E-31	postive
TET2	AC004771.	0.545197	3.38E-33	postive
TET3	AC004771.	0.347376	4.23E-13	postive
NSUN6	AC124069.	0.3356	2.82E-12	postive
NSUN7	AC124069.	0.303411	3.36E-10	postive
TET1	AC124069.	0.65271	2.97E-51	postive
TET2	AC124069.	0.414636	1.65E-18	postive
TET3	AC124069.	0.342616	9.20E-13	postive
NSUN6	AL133355.	0.417465	9.18E-19	postive
ALYREF	AL133355.	-0.31369	7.77E-11	negative
NEIL1	AL133355.	0.38215	9.70E-16	postive
DNMT3A	A2M-AS1	0.325227	1.40E-11	postive
MECP2	A2M-AS1	0.341432	1.11E-12	postive
TET1	A2M-AS1	0.341274	1.14E-12	postive
NSUN3	AL353801.	0.328726	8.21E-12	postive
NSUN6	AL353801.	0.365344	2.01E-14	postive
TET1	AL353801.	0.515787	2.58E-29	postive
NSUN3	AC025031.	0.578795	3.96E-38	postive
ZBTB38	AC025031.	0.312098	9.78E-11	postive
TET1	AC025031.	0.558583	4.28E-35	postive
TET2	AC025031.	0.604198	2.97E-42	postive
TET3	AC025031.	0.330875	5.89E-12	postive
NSUN7	PTOV1-AS	0.346787	4.66E-13	postive
DNMT3A	PTOV1-AS	0.390848	1.88E-16	postive
RAD52	PTOV1-AS	0.372493	5.65E-15	postive
NSUN6	RAB11B-A	0.314603	6.80E-11	postive
MBD3	RAB11B-A	0.325872	1.27E-11	postive
NEIL1	RAB11B-A	0.467748	9.85E-24	postive
SMUG1	RAB11B-A	0.417385	9.34E-19	postive
ZBTB38	RAB11B-A	-0.34615	5.17E-13	negative
SMUG1	FAM83C-A	0.300727	4.89E-10	postive
RAD52	AP005329.	0.360664	4.52E-14	postive
NSUN7	AC010615.	0.465417	1.75E-23	postive
DNMT3A	AC010615.	0.321453	2.47E-11	postive
NEIL1	AC010615.	0.305234	2.60E-10	postive
TET1	AC010615.	0.426192	1.44E-19	postive
TET2	AC010615.	0.330838	5.93E-12	postive
TET3	AC010615.	0.434526	2.34E-20	postive
NSUN6	TTC23L-AS	0.367283	1.43E-14	postive
NSUN7	TTC23L-AS	0.344724	6.53E-13	postive
DNMT3A	TTC23L-AS	0.310322	1.26E-10	postive
SMUG1	TTC23L-AS	0.327154	1.04E-11	postive
NSUN6	AP001160.	0.301426	4.43E-10	postive
RAD52	AP001160.	0.401596	2.33E-17	postive

NEIL1	AP001160.	0.405954	9.74E-18	postive
TET2	AP001160.	0.343208	8.35E-13	postive
NSUN6	AL024498.	0.320074	3.04E-11	postive
NSUN7	AL024498.	0.368557	1.14E-14	postive
NSUN6	AL158835.	0.348782	3.36E-13	postive
NSUN7	AL158835.	0.336722	2.36E-12	postive
DNMT3A	AL158835.	0.364959	2.15E-14	postive
RAD52	AL158835.	0.328478	8.53E-12	postive
TET1	AL158835.	0.404419	1.32E-17	postive
TET3	AL158835.	0.332785	4.38E-12	postive
NSUN6	GHRLOS	0.357468	7.82E-14	postive
RAD52	GHRLOS	0.362597	3.24E-14	postive
TET1	GHRLOS	0.47587	1.29E-24	postive
TET2	GHRLOS	0.318025	4.12E-11	postive
TET3	GHRLOS	0.353359	1.57E-13	postive
DNMT3A	AC022007.	0.311385	1.08E-10	postive
NSUN6	AC245140.	0.343651	7.78E-13	postive
RAD52	AC245140.	0.316438	5.20E-11	postive
MECP2	AC245140.	0.48234	2.45E-25	postive
ZBTB33	AC245140.	0.347147	4.40E-13	postive
TET3	AC245140.	0.316297	5.31E-11	postive
NSUN3	AC093620.	0.530356	3.43E-31	postive
TET1	AC093620.	0.429216	7.49E-20	postive
TET2	AC093620.	0.446882	1.43E-21	postive
DNMT1	IPO9-AS1	0.33301	4.23E-12	postive
DNMT3A	IPO9-AS1	0.343101	8.50E-13	postive
NTHL1	IPO9-AS1	0.36202	3.58E-14	postive
NSUN7	AC112493.	0.347524	4.13E-13	postive
TET1	AC112493.	0.573677	2.43E-37	postive
TET3	AC112493.	0.303337	3.40E-10	postive
NSUN7	ASB16-AS	0.359336	5.68E-14	postive
NEIL1	ASB16-AS	0.419442	6.06E-19	postive
NSUN7	AL161669.	0.303566	3.29E-10	postive
NEIL1	AL161669.	0.372699	5.45E-15	postive
NSUN6	AC093535.	0.437746	1.14E-20	postive
NSUN7	AC093535.	0.336778	2.34E-12	postive
TET1	AC093535.	0.618142	1.11E-44	postive
TET2	AC093535.	0.488922	4.38E-26	postive
TET3	AC093535.	0.36632	1.69E-14	postive
NSUN3	AC010186.	0.424035	2.29E-19	postive
NSUN7	AC010186.	0.327351	1.01E-11	postive
TRDMT1	AC010186.	0.321303	2.53E-11	postive
RAD52	AC010186.	0.353843	1.44E-13	postive
TET1	AC010186.	0.655271	8.93E-52	postive
TET2	AC010186.	0.46237	3.67E-23	postive
TET3	AC010186.	0.397359	5.35E-17	postive
DNMT1	AC090152.	0.380291	1.37E-15	postive
NSUN6	AL139397.	0.318018	4.12E-11	postive
NSUN7	AL139397.	0.391439	1.68E-16	postive
DNMT3A	AL139397.	0.333687	3.80E-12	postive
RAD52	AL139397.	0.317871	4.21E-11	postive
ZBTB33	AL139397.	0.311536	1.06E-10	postive
TET1	AL139397.	0.539034	2.37E-32	postive
TET3	AL139397.	0.41847	7.44E-19	postive
NSUN6	RNASEH2E	0.333196	4.11E-12	postive
NSUN7	RNASEH2E	0.312972	8.62E-11	postive
TET1	RNASEH2E	0.665976	5.19E-54	postive
TET2	RNASEH2E	0.374957	3.62E-15	postive

TET3	RNASEH2E	0.348262	3.66E-13	postive
TRDMT1	LMF1-AS1	0.396012	6.96E-17	postive
NSUN3	AGAP1-IT:	0.510373	1.22E-28	postive
TET1	AGAP1-IT:	0.504848	5.77E-28	postive
TET2	AGAP1-IT:	0.395674	7.43E-17	postive
NSUN6	AC092794.	0.384716	6.01E-16	postive
NSUN7	AC092794.	0.322091	2.25E-11	postive
RAD52	AC092794.	0.303403	3.37E-10	postive
NEIL1	AC092794.	0.360422	4.72E-14	postive
TET1	AC092794.	0.512636	6.38E-29	postive
TET2	AC092794.	0.583824	6.45E-39	postive
TET3	AC092794.	0.362971	3.04E-14	postive
NSUN3	AL109923.	0.354592	1.27E-13	postive
NSUN6	AL109923.	0.317739	4.29E-11	postive
TET1	AL109923.	0.683176	8.34E-58	postive
TET2	AL109923.	0.517654	1.50E-29	postive
TET3	AL109923.	0.33723	2.18E-12	postive
NSUN3	AC011465.	0.310595	1.22E-10	postive
UHRF2	AC011465.	0.30712	2.00E-10	postive
TET1	AC011465.	0.396214	6.69E-17	postive
TET2	AC011465.	0.566582	2.86E-36	postive
TET3	AC011465.	0.305964	2.35E-10	postive
NSUN3	AC004837.	0.405721	1.02E-17	postive
TRDMT1	AC004837.	0.34509	6.15E-13	postive
UHRF2	AC004837.	0.360049	5.03E-14	postive
ZBTB38	AC004837.	0.305795	2.41E-10	postive
TET1	AC004837.	0.712515	6.41E-65	postive
TET2	AC004837.	0.582666	9.83E-39	postive
TET3	AC004837.	0.384269	6.54E-16	postive
NSUN3	MANEA-D	0.37052	8.04E-15	postive
NSUN6	MANEA-D	0.450285	6.51E-22	postive
NSUN7	MANEA-D	0.331659	5.22E-12	postive
RAD52	MANEA-D	0.339321	1.56E-12	postive
NEIL1	MANEA-D	0.445197	2.11E-21	postive
TET1	MANEA-D	0.490198	3.12E-26	postive
TET2	MANEA-D	0.569741	9.61E-37	postive
NEIL1	AL021154.	0.355364	1.12E-13	postive
NSUN6	AC010271.	0.364697	2.25E-14	postive
DNMT3A	AC010271.	0.357027	8.43E-14	postive
RAD52	AC010271.	0.452557	3.82E-22	postive
MBD1	AC010271.	0.3425	9.37E-13	postive
TET3	AC010271.	0.339412	1.54E-12	postive
NSUN6	AC009065.	0.304672	2.82E-10	postive
RAD52	AC009065.	0.410054	4.24E-18	postive
NEIL1	AC009065.	0.49696	5.06E-27	postive
SMUG1	AC009065.	0.352782	1.73E-13	postive
ZBTB38	AC009065.	-0.30101	4.70E-10	negative
NSUN3	COL4A2-A	0.533098	1.49E-31	postive
ZBTB38	COL4A2-A	0.327106	1.05E-11	postive
TET1	COL4A2-A	0.465532	1.70E-23	postive
TET2	COL4A2-A	0.303353	3.39E-10	postive
NSUN3	AC106820.	0.305631	2.46E-10	postive
NSUN7	AC106820.	0.370412	8.20E-15	postive
DNMT1	AC106820.	0.315012	6.41E-11	postive
DNMT3A	AC106820.	0.483568	1.78E-25	postive
MECP2	AC106820.	0.307638	1.85E-10	postive
TDG	AC106820.	0.315928	5.60E-11	postive
ZBTB33	AC106820.	0.374204	4.15E-15	postive

TET1	AC106820.	0.566841	2.61E-36	postive
TET2	AC106820.	0.336242	2.55E-12	postive
TET3	AC106820.	0.42474	1.97E-19	postive
NSUN3	AP000873.	0.307765	1.82E-10	postive
NSUN6	AP000873.	0.364423	2.36E-14	postive
NSUN7	AP000873.	0.406371	8.95E-18	postive
DNMT3A	AP000873.	0.352641	1.77E-13	postive
RAD52	AP000873.	0.363188	2.92E-14	postive
TET1	AP000873.	0.516363	2.18E-29	postive
TET2	AP000873.	0.332823	4.35E-12	postive
TET3	AP000873.	0.42114	4.24E-19	postive
NSUN3	AC022154.	0.593712	1.66E-40	postive
DNMT3A	AC022154.	0.339744	1.46E-12	postive
TRDMT1	AC022154.	0.344813	6.44E-13	postive
RAD52	AC022154.	0.314772	6.64E-11	postive
MECP2	AC022154.	0.313036	8.54E-11	postive
ZBTB38	AC022154.	0.320526	2.84E-11	postive
TET1	AC022154.	0.440312	6.41E-21	postive
TET2	AC022154.	0.511522	8.77E-29	postive
TET3	AC022154.	0.39228	1.43E-16	postive
TET1	AC027307.	0.30773	1.83E-10	postive
NSUN6	AC099778.	0.368452	1.16E-14	postive
NSUN7	AC099778.	0.33758	2.06E-12	postive
NEIL1	AC099778.	0.429108	7.67E-20	postive
SMUG1	AC099778.	0.309823	1.36E-10	postive
MBD3	AC002401.	0.321582	2.43E-11	postive
NSUN3	TGFB2-AS	0.555677	1.12E-34	postive
TRDMT1	TGFB2-AS	0.30427	2.98E-10	postive
NSUN6	AC084125.	0.402881	1.80E-17	postive
RAD52	AC084125.	0.368479	1.16E-14	postive
NEIL1	AC084125.	0.368515	1.15E-14	postive
NSUN6	AC104564.	0.351439	2.16E-13	postive
NSUN7	AC104564.	0.359572	5.46E-14	postive
DNMT3A	AC104564.	0.320245	2.96E-11	postive
RAD52	AC104564.	0.382471	9.14E-16	postive
MBD1	AC104564.	0.354752	1.24E-13	postive
MECP2	AC104564.	0.312092	9.79E-11	postive
NEIL1	AC104564.	0.473493	2.35E-24	postive
TET2	AC104564.	0.34826	3.66E-13	postive
TET3	AC104564.	0.359864	5.19E-14	postive
NSUN7	AC137630.	0.427653	1.05E-19	postive
DNMT3A	AC137630.	0.312048	9.85E-11	postive
NSUN6	AC025031.	0.337239	2.17E-12	postive
NSUN7	AC025031.	0.319408	3.35E-11	postive
TET1	AC025031.	0.681924	1.61E-57	postive
TET2	AC025031.	0.404785	1.23E-17	postive
TET3	AC025031.	0.351475	2.15E-13	postive
NSUN3	AC138932.	0.461169	4.91E-23	postive
NSUN7	AC138932.	0.300307	5.18E-10	postive
RAD52	AC138932.	0.380468	1.32E-15	postive
UHRF2	AC138932.	0.300674	4.92E-10	postive
TET1	AC138932.	0.576129	1.02E-37	postive
TET2	AC138932.	0.543484	5.83E-33	postive
TET3	AC138932.	0.360639	4.54E-14	postive
TET1	AC092755.	0.433958	2.65E-20	postive
TET2	AC092755.	0.396552	6.27E-17	postive
NSUN3	AC005253.	0.333728	3.78E-12	postive
NSUN6	AC005253.	0.389925	2.25E-16	postive

NSUN7	AC005253.	0.345515	5.74E-13	postive
DNMT3A	AC005253.	0.324663	1.53E-11	postive
RAD52	AC005253.	0.399621	3.44E-17	postive
MBD1	AC005253.	0.386787	4.07E-16	postive
MECP2	AC005253.	0.337978	1.93E-12	postive
NEIL1	AC005253.	0.438418	9.82E-21	postive
UHRF2	AC005253.	0.301798	4.21E-10	postive
ZBTB33	AC005253.	0.409859	4.42E-18	postive
TET1	AC005253.	0.344412	6.87E-13	postive
TET2	AC005253.	0.538573	2.73E-32	postive
TET3	AC005253.	0.437396	1.23E-20	postive
NSUN6	LINC0129(	0.341953	1.02E-12	postive
NSUN7	LINC0129(	0.305018	2.68E-10	postive
RAD52	LINC0129(	0.350098	2.70E-13	postive
TET1	LINC0129(	0.357908	7.26E-14	postive
TET2	LINC0129(	0.315152	6.28E-11	postive
TET3	LINC0129(	0.393477	1.14E-16	postive
UHRF2	KCNQ1OT	0.302029	4.08E-10	postive
TET1	KCNQ1OT	0.345923	5.37E-13	postive
TET2	KCNQ1OT	0.550892	5.40E-34	postive
NSUN3	AL031775.	0.333308	4.04E-12	postive
NSUN6	AL031775.	0.300931	4.75E-10	postive
NSUN7	AL031775.	0.30207	4.06E-10	postive
TET1	AL031775.	0.405442	1.08E-17	postive
TET2	AL031775.	0.347357	4.25E-13	postive
TET3	AL031775.	0.391831	1.56E-16	postive
NSUN3	AC022272.	0.348245	3.67E-13	postive
NSUN6	AC022272.	0.326108	1.22E-11	postive
NSUN7	AC022272.	0.315992	5.55E-11	postive
ZBTB33	AC022272.	0.304703	2.81E-10	postive
TET1	AC022272.	0.690755	1.46E-59	postive
TET2	AC022272.	0.546716	2.08E-33	postive
TET3	AC022272.	0.401552	2.35E-17	postive
NSUN3	AP002907.	0.348778	3.36E-13	postive
TET1	AP002907.	0.463121	3.06E-23	postive
TET2	AP002907.	0.552806	2.89E-34	postive
TET3	AP002907.	0.372076	6.09E-15	postive
NTHL1	LINC01571	0.388573	2.91E-16	postive
NSUN3	LINC0225(	0.532908	1.58E-31	postive
MECP2	LINC0225(	0.300653	4.94E-10	postive
ZBTB38	LINC0225(	0.402849	1.81E-17	postive
TET2	LINC0225(	0.333097	4.17E-12	postive
TET1	AC004471.	0.444806	2.31E-21	postive
NSUN6	AC110285.	0.310713	1.19E-10	postive
NEIL1	AC110285.	0.504506	6.34E-28	postive
DNMT1	AC090587.	0.309615	1.40E-10	postive
ZBTB38	AC090587.	0.329398	7.40E-12	postive
ZBTB4	AC090587.	0.33082	5.94E-12	postive
RAD52	AC005840.	0.300395	5.12E-10	postive
NEIL1	AC005840.	0.393238	1.19E-16	postive
SMUG1	AC005840.	0.399642	3.42E-17	postive
TET1	AP002784.	0.346006	5.30E-13	postive
NSUN6	LINC0215(	0.376895	2.55E-15	postive
TET1	LINC0215(	0.53313	1.47E-31	postive
TET2	LINC0215(	0.500661	1.84E-27	postive
TET3	LINC0215(	0.336958	2.27E-12	postive
NSUN6	AC027682.	0.312598	9.10E-11	postive
RAD52	AC027682.	0.353381	1.56E-13	postive

NEIL1	AC027682.	0.534272	1.04E-31	postive
NSUN6	AC010487.	0.391138	1.78E-16	postive
NEIL1	AC010487.	0.483574	1.78E-25	postive
SMUG1	AC010487.	0.305073	2.66E-10	postive
NSUN6	Z84485.1	0.357746	7.46E-14	postive
NSUN7	Z84485.1	0.386851	4.03E-16	postive
RAD52	Z84485.1	0.338899	1.67E-12	postive
MBD1	Z84485.1	0.353578	1.51E-13	postive
NEIL1	Z84485.1	0.526518	1.09E-30	postive
NEIL1	EPB41L4A-	0.357663	7.57E-14	postive
ZBTB38	EPB41L4A-	-0.31838	3.90E-11	negative
TET1	AL355574.	0.340805	1.23E-12	postive
NSUN3	AC115618.	0.367834	1.30E-14	postive
NSUN7	AP000845.	0.366045	1.78E-14	postive
DNMT3A	AP000845.	0.383473	7.58E-16	postive
RAD52	AP000845.	0.359557	5.47E-14	postive
TET1	AP000845.	0.332236	4.77E-12	postive
TET3	AP000845.	0.317347	4.55E-11	postive
NSUN6	AC104118.	0.314989	6.43E-11	postive
RAD52	AC104118.	0.324711	1.51E-11	postive
NEIL1	AC104118.	0.402714	1.86E-17	postive
MBD1	AL512625.	0.366596	1.61E-14	postive
ZBTB33	AL512625.	0.30882	1.57E-10	postive
TET3	AL512625.	0.316957	4.82E-11	postive
ZBTB38	AC006213.	0.37918	1.68E-15	postive
TET3	AC006213.	0.327935	9.27E-12	postive
NSUN6	MCCC1-A:	0.510042	1.34E-28	postive
NSUN7	MCCC1-A:	0.424732	1.97E-19	postive
RAD52	MCCC1-A:	0.446101	1.72E-21	postive
MBD1	MCCC1-A:	0.304909	2.73E-10	postive
NEIL1	MCCC1-A:	0.49908	2.84E-27	postive
TET1	MCCC1-A:	0.313179	8.37E-11	postive
TET2	MCCC1-A:	0.470031	5.59E-24	postive
TET3	MCCC1-A:	0.376891	2.55E-15	postive
DNMT3A	AC092295.	0.331437	5.40E-12	postive
NSUN7	WAC-AS1	0.42537	1.72E-19	postive
NSUN3	AC018410.	0.385163	5.53E-16	postive
UHRF2	AC018410.	0.331665	5.21E-12	postive
TET1	AC018410.	0.575754	1.17E-37	postive
TET2	AC018410.	0.586708	2.25E-39	postive
TET3	AC018410.	0.309523	1.42E-10	postive
NSUN3	AL355073.	0.390977	1.84E-16	postive
TRDMT1	AL355073.	0.322968	1.97E-11	postive
NSUN6	AC011389.	0.398331	4.43E-17	postive
TET1	AC011389.	0.467121	1.15E-23	postive
TET2	AC011389.	0.327066	1.06E-11	postive
NSUN6	AC245884.	0.370705	7.78E-15	postive
NSUN7	AC245884.	0.336187	2.57E-12	postive
DNMT3A	AC245884.	0.303956	3.12E-10	postive
NEIL1	AC245884.	0.325264	1.39E-11	postive
TET1	AC245884.	0.514511	3.72E-29	postive
TET2	AC245884.	0.483114	2.01E-25	postive
TET3	AC245884.	0.444657	2.39E-21	postive
NSUN3	AL121989.	0.451902	4.46E-22	postive
UHRF2	AL121989.	0.318427	3.88E-11	postive
TET1	AL121989.	0.635946	5.82E-48	postive
TET2	AL121989.	0.570469	7.46E-37	postive
TET3	AL121989.	0.322126	2.24E-11	postive

NSUN5	AC012676.	0.311628	1.05E-10	postive
MBD3	AC012676.	0.312326	9.47E-11	postive
NTHL1	AC012676.	0.300888	4.78E-10	postive
NEIL1	AC006449.	0.338881	1.67E-12	postive
TET3	AC006449.	0.350617	2.48E-13	postive
NSUN6	AC005332.	0.33107	5.72E-12	postive
NSUN7	AC005332.	0.369056	1.04E-14	postive
DNMT3A	AC005332.	0.433538	2.91E-20	postive
RAD52	AC005332.	0.474943	1.63E-24	postive
NEIL1	AC005332.	0.465327	1.79E-23	postive
NSUN6	AL390067.	0.327193	1.04E-11	postive
NSUN7	AL390067.	0.354742	1.24E-13	postive
TET1	AL390067.	0.50614	4.02E-28	postive
NSUN3	AC026124.	0.312645	9.04E-11	postive
UHRF2	AC026124.	0.311275	1.10E-10	postive
TET1	AC026124.	0.666797	3.46E-54	postive
TET2	AC026124.	0.515767	2.59E-29	postive
TET3	AC026124.	0.344696	6.56E-13	postive
DNMT1	AC231981.	0.312433	9.32E-11	postive
DNMT3A	AC231981.	0.417473	9.17E-19	postive
UNG	AC231981.	0.313337	8.18E-11	postive
NSUN6	AC005387.	0.329988	6.76E-12	postive
RAD52	AC005387.	0.415582	1.36E-18	postive
NEIL1	AC005387.	0.591742	3.47E-40	postive
MBD3	AC135178.	0.406442	8.83E-18	postive
NEIL1	AC135178.	0.312038	9.87E-11	postive
MBD3	MELTF-AS	0.346761	4.68E-13	postive
TET1	AC010175.	0.38359	7.42E-16	postive
NSUN3	AC006480.	0.419481	6.01E-19	postive
NSUN6	AC006480.	0.392017	1.51E-16	postive
NSUN7	AC006480.	0.311275	1.10E-10	postive
RAD52	AC006480.	0.353694	1.48E-13	postive
MECP2	AC006480.	0.30088	4.78E-10	postive
NEIL1	AC006480.	0.325271	1.39E-11	postive
TET1	AC006480.	0.475268	1.50E-24	postive
TET2	AC006480.	0.457707	1.13E-22	postive
TET3	AC006480.	0.347525	4.13E-13	postive
RAD52	AL121782.	0.326063	1.23E-11	postive
NSUN7	AC116913.	0.313047	8.53E-11	postive
NEIL1	AC116913.	0.427926	9.91E-20	postive
TET1	AC116913.	0.385165	5.53E-16	postive
TET2	AC116913.	0.345731	5.54E-13	postive
TET3	AC100860.	0.322192	2.21E-11	postive
DNMT3B	CEBPA-DT	0.377257	2.39E-15	postive
SMUG1	CEBPA-DT	0.342834	8.88E-13	postive
TET3	CEBPA-DT	0.304528	2.88E-10	postive
NSUN3	AL356299.	0.303082	3.52E-10	postive
NSUN6	AL356299.	0.309158	1.49E-10	postive
NSUN7	AL356299.	0.307312	1.94E-10	postive
DNMT3B	AL356299.	0.300093	5.33E-10	postive
RAD52	AL356299.	0.437395	1.23E-20	postive
NEIL1	AL356299.	0.364354	2.39E-14	postive
TET1	AL356299.	0.41591	1.27E-18	postive
TET2	AL356299.	0.48454	1.39E-25	postive
TET3	AL356299.	0.329317	7.49E-12	postive
NSUN6	AC125257.	0.343239	8.31E-13	postive
NSUN7	AC125257.	0.418545	7.32E-19	postive
DNMT3A	AC125257.	0.451393	5.03E-22	postive

DNMT3B	AC125257.	0.302662	3.73E-10	postive
RAD52	AC125257.	0.384691	6.04E-16	postive
NEIL1	AC125257.	0.307263	1.96E-10	postive
TDG	AC125257.	0.454789	2.26E-22	postive
UNG	AC125257.	0.305167	2.63E-10	postive
ZBTB33	AC125257.	0.433185	3.14E-20	postive
TET1	AC125257.	0.341472	1.11E-12	postive
TET2	AC125257.	0.350567	2.50E-13	postive
TET3	AC125257.	0.444342	2.57E-21	postive
NSUN5	HYI-AS1	0.301157	4.60E-10	postive
MBD3	HYI-AS1	0.493944	1.15E-26	postive
NTHL1	HYI-AS1	0.360991	4.28E-14	postive
NSUN6	AL080317.	0.349037	3.22E-13	postive
NSUN7	AL080317.	0.338147	1.88E-12	postive
TET1	AL080317.	0.68369	6.36E-58	postive
TET2	AL080317.	0.375173	3.49E-15	postive
TET3	AL080317.	0.376965	2.52E-15	postive
NSUN3	AC103769.	0.324255	1.62E-11	postive
TET1	AC103769.	0.551082	5.07E-34	postive
TET2	AC103769.	0.51716	1.73E-29	postive
TET3	AC103769.	0.309805	1.36E-10	postive
NSUN7	AC022784.	0.341539	1.09E-12	postive
TET1	AC022784.	0.615872	2.81E-44	postive
NSUN7	DOCK8-AS	0.328221	8.87E-12	postive
DNMT3A	AC104794.	0.343065	8.55E-13	postive
DNMT1	FOXD3-AS	0.326804	1.10E-11	postive
RAD52	AC005324.	0.300973	4.72E-10	postive
NEIL1	AC005324.	0.385847	4.86E-16	postive
NSUN6	AL136221.	0.329364	7.44E-12	postive
NSUN7	AL136221.	0.365277	2.03E-14	postive
NEIL1	AL136221.	0.309915	1.34E-10	postive
TET1	AL136221.	0.531464	2.45E-31	postive
TET2	AL136221.	0.508042	2.35E-28	postive
TET3	AL136221.	0.366997	1.50E-14	postive
NSUN6	KLF3-AS1	0.373315	4.88E-15	postive
NSUN7	KLF3-AS1	0.309457	1.43E-10	postive
RAD52	KLF3-AS1	0.347252	4.32E-13	postive
NEIL1	KLF3-AS1	0.547989	1.38E-33	postive
TET2	KLF3-AS1	0.30254	3.80E-10	postive
NSUN7	AC000068.	0.321034	2.63E-11	postive
DNMT1	AC000068.	0.303313	3.41E-10	postive
DNMT3A	AC000068.	0.33942	1.54E-12	postive
TET1	AC000068.	0.365434	1.98E-14	postive
NSUN3	AL132780.	0.354951	1.20E-13	postive
NSUN7	AL132780.	0.392004	1.51E-16	postive
MBD1	AL132780.	0.347496	4.15E-13	postive
MECP2	AL132780.	0.319106	3.51E-11	postive
TET1	AL132780.	0.318206	4.01E-11	postive
TET2	AL132780.	0.380631	1.28E-15	postive
TET3	AL132780.	0.340253	1.34E-12	postive
NSUN6	AL022322.	0.43858	9.47E-21	postive
NSUN7	AL022322.	0.376416	2.78E-15	postive
RAD52	AL022322.	0.417183	9.74E-19	postive
NEIL1	AL022322.	0.464893	1.99E-23	postive
NSUN5	AC010913.	0.371155	7.18E-15	postive
NTHL1	AC010913.	0.441371	5.05E-21	postive
NSUN6	SDK1-AS1	0.326785	1.10E-11	postive
TET1	SDK1-AS1	0.683954	5.53E-58	postive

TET2	SDK1-AS1	0.335484	2.87E-12	postive
TET3	SDK1-AS1	0.360517	4.64E-14	postive
RAD52	AC109449.	0.347911	3.88E-13	postive
NEIL1	AC109449.	0.402892	1.80E-17	postive
NSUN3	AC087854.	0.595986	7.02E-41	postive
TRDMT1	AC087854.	0.456775	1.41E-22	postive
ZBTB38	AC087854.	0.323765	1.75E-11	postive
TET1	AC087854.	0.527254	8.76E-31	postive
TET2	AC087854.	0.42809	9.57E-20	postive
MBD3	AC112491.	0.316561	5.11E-11	postive
NTHL1	AC112491.	0.42826	9.22E-20	postive
SMUG1	AC112491.	0.341778	1.05E-12	postive
ZBTB38	AC112491.	-0.36776	1.31E-14	negative
NSUN6	LINC01341	0.386374	4.40E-16	postive
NEIL1	LINC01341	0.407782	6.73E-18	postive
NSUN5	AC073896.	0.31648	5.17E-11	postive
MBD3	AC073896.	0.332507	4.57E-12	postive
NTHL1	AC073896.	0.457543	1.17E-22	postive
SMUG1	AC073896.	0.541606	1.06E-32	postive
ZBTB38	AC073896.	-0.30561	2.47E-10	negative
NSUN6	AL390195.	0.398699	4.12E-17	postive
NSUN7	AL390195.	0.323759	1.75E-11	postive
RAD52	AL390195.	0.309232	1.48E-10	postive
NEIL1	AL390195.	0.419197	6.39E-19	postive
TET1	AL390195.	0.35248	1.82E-13	postive
TET2	AL390195.	0.50537	4.98E-28	postive
TET3	AL390195.	0.380972	1.21E-15	postive
NSUN6	AC092611.	0.406149	9.36E-18	postive
NSUN7	AC092611.	0.376731	2.63E-15	postive
RAD52	AC092611.	0.383311	7.82E-16	postive
ZBTB33	AC092611.	0.314674	6.73E-11	postive
TET1	AC092611.	0.54444	4.30E-33	postive
TET2	AC092611.	0.50401	7.28E-28	postive
TET3	AC092611.	0.471999	3.42E-24	postive
NSUN7	AL118511.	0.331323	5.50E-12	postive
DNMT3A	AL118511.	0.463794	2.60E-23	postive
TET1	POLH-AS1	0.418581	7.27E-19	postive
NSUN6	LINC02803	0.34075	1.24E-12	postive
NEIL1	LINC02803	0.36914	1.03E-14	postive
NSUN4	AC008764.	0.351516	2.13E-13	postive
NSUN7	AC008764.	0.303549	3.30E-10	postive
RAD52	AC008764.	0.321296	2.53E-11	postive
MBD1	AC008764.	0.39189	1.54E-16	postive
MECP2	AC008764.	0.43629	1.58E-20	postive
ZBTB33	AC008764.	0.398179	4.56E-17	postive
ZBTB38	AC008764.	0.315682	5.81E-11	postive
ZBTB4	AC008764.	0.337671	2.03E-12	postive
TET2	AC008764.	0.446625	1.52E-21	postive
TET3	AC008764.	0.415813	1.30E-18	postive
TET3	UVRAG-DT	0.317059	4.75E-11	postive
DNMT3A	LINC01918	0.360022	5.05E-14	postive
NEIL1	AP002807.	0.428387	8.97E-20	postive
TET1	AC010226.	0.312319	9.48E-11	postive
NEIL1	AC105020.	0.479178	5.55E-25	postive
NSUN6	GATA3-AS	0.503364	8.72E-28	postive
NEIL1	GATA3-AS	0.36403	2.53E-14	postive
NTHL1	GATA3-AS	0.327919	9.29E-12	postive
SMUG1	GATA3-AS	0.331749	5.14E-12	postive

NSUN6	AC063943.	0.309931	1.34E-10	postive
NSUN7	AC063943.	0.309543	1.41E-10	postive
TET1	AC063943.	0.515848	2.53E-29	postive
NSUN7	ATP2B1-A	0.346066	5.25E-13	postive
MBD3	AC114271.	0.402982	1.77E-17	postive
NSUN6	AL136304.	0.336767	2.34E-12	postive
NSUN7	AL136304.	0.357199	8.19E-14	postive
RAD52	AL136304.	0.342956	8.70E-13	postive
NEIL1	AL136304.	0.31395	7.48E-11	postive
NSUN3	AC007622.	0.416927	1.03E-18	postive
NSUN6	AC007622.	0.349616	2.93E-13	postive
NSUN7	AC007622.	0.311478	1.07E-10	postive
RAD52	AC007622.	0.301281	4.52E-10	postive
ZBTB33	AC007622.	0.345931	5.37E-13	postive
TET1	AC007622.	0.682816	1.01E-57	postive
TET2	AC007622.	0.640283	8.57E-49	postive
TET3	AC007622.	0.454108	2.65E-22	postive
NSUN3	DENND6A	0.446688	1.50E-21	postive
TRDMT1	DENND6A	0.305243	2.60E-10	postive
UHRF2	DENND6A	0.334495	3.35E-12	postive
TET1	DENND6A	0.574335	1.93E-37	postive
TET2	DENND6A	0.575312	1.37E-37	postive
NSUN3	LSAMP-AS	0.689008	3.75E-59	postive
TRDMT1	LSAMP-AS	0.477626	8.25E-25	postive
TET2	LSAMP-AS	0.351652	2.09E-13	postive
TET1	FGF12-AS	0.581782	1.35E-38	postive
NSUN3	WASHC5-,	0.415942	1.26E-18	postive
NSUN6	WASHC5-,	0.313255	8.28E-11	postive
NSUN7	WASHC5-,	0.3068	2.09E-10	postive
TRDMT1	WASHC5-,	0.31085	1.17E-10	postive
UHRF2	WASHC5-,	0.320116	3.02E-11	postive
ZBTB33	WASHC5-,	0.314205	7.21E-11	postive
TET1	WASHC5-,	0.698315	2.28E-61	postive
TET2	WASHC5-,	0.559881	2.77E-35	postive
TET3	WASHC5-,	0.415028	1.53E-18	postive
NTHL1	AL138966.	0.302639	3.75E-10	postive
SMUG1	AL138966.	0.331185	5.62E-12	postive
TET1	AC093799.	0.343815	7.57E-13	postive
TET2	AC093799.	0.461208	4.87E-23	postive
NSUN3	NCBP2-AS	0.43979	7.22E-21	postive
NSUN6	NCBP2-AS	0.422272	3.33E-19	postive
NSUN7	NCBP2-AS	0.319328	3.39E-11	postive
RAD52	NCBP2-AS	0.376637	2.67E-15	postive
MBD1	NCBP2-AS	0.333813	3.73E-12	postive
MECP2	NCBP2-AS	0.343563	7.89E-13	postive
ZBTB33	NCBP2-AS	0.387647	3.46E-16	postive
TET1	NCBP2-AS	0.519734	8.16E-30	postive
TET2	NCBP2-AS	0.556341	9.02E-35	postive
TET3	NCBP2-AS	0.48917	4.10E-26	postive
NTHL1	SLC12A9-,	0.302976	3.57E-10	postive
MBD3	CAHM	0.321755	2.36E-11	postive
DNMT3A	EIF2AK3-D	0.40224	2.05E-17	postive
RAD52	EIF2AK3-D	0.307437	1.91E-10	postive
TET3	EIF2AK3-D	0.334444	3.38E-12	postive
NSUN6	TONSL-AS	0.359001	6.02E-14	postive
NEIL1	TONSL-AS	0.355584	1.08E-13	postive
NSUN3	AC068790.	0.417535	9.05E-19	postive
NSUN6	AC068790.	0.307436	1.91E-10	postive

UHRF2	AC068790.	0.31189	1.01E-10	postive
TET1	AC068790.	0.628285	1.60E-46	postive
TET2	AC068790.	0.599646	1.73E-41	postive
TET3	AC068790.	0.376881	2.56E-15	postive
NSUN3	DLEU1	0.318376	3.91E-11	postive
TET1	DLEU1	0.699157	1.42E-61	postive
TET2	DLEU1	0.492472	1.70E-26	postive
TET3	DLEU1	0.352913	1.69E-13	postive
NSUN3	AP001619.	0.320759	2.74E-11	postive
NSUN6	AP001619.	0.392784	1.30E-16	postive
NSUN7	AP001619.	0.35064	2.47E-13	postive
RAD52	AP001619.	0.331793	5.11E-12	postive
NEIL1	AP001619.	0.332738	4.41E-12	postive
UHRF2	AP001619.	0.314546	6.86E-11	postive
ZBTB33	AP001619.	0.36601	1.79E-14	postive
TET1	AP001619.	0.571134	5.92E-37	postive
TET2	AP001619.	0.584547	4.96E-39	postive
TET3	AP001619.	0.484903	1.26E-25	postive
NSUN6	C2orf49-D	0.313417	8.08E-11	postive
DNMT3A	C2orf49-D	0.474171	1.98E-24	postive
RAD52	C2orf49-D	0.431083	4.99E-20	postive
MBD1	C2orf49-D	0.317205	4.64E-11	postive
ZBTB33	C2orf49-D	0.33745	2.10E-12	postive
TET1	C2orf49-D	0.342826	8.89E-13	postive
TET2	C2orf49-D	0.3044	2.93E-10	postive
TET3	C2orf49-D	0.453687	2.93E-22	postive
NSUN7	ADNP-AS1	0.408661	5.63E-18	postive
RAD52	ADNP-AS1	0.380267	1.37E-15	postive
NEIL1	ADNP-AS1	0.366006	1.79E-14	postive
TET1	ADNP-AS1	0.305033	2.68E-10	postive
TET2	ADNP-AS1	0.44784	1.15E-21	postive
TET3	ADNP-AS1	0.315456	6.00E-11	postive
NSUN3	AC069542.	0.422354	3.28E-19	postive
UHRF2	AC069542.	0.310307	1.27E-10	postive
TET1	AC069542.	0.466581	1.31E-23	postive
TET2	AC069542.	0.589013	9.60E-40	postive
TET1	AC073283.	0.44651	1.56E-21	postive
TET2	AC073283.	0.345824	5.46E-13	postive
TET3	AC073283.	0.339678	1.47E-12	postive
NSUN3	AC004584.	0.314058	7.36E-11	postive
NSUN6	AC004584.	0.336405	2.48E-12	postive
DNMT3A	AC004584.	0.309471	1.43E-10	postive
RAD52	AC004584.	0.411518	3.15E-18	postive
NEIL1	AC004584.	0.347282	4.30E-13	postive
TET1	AC004584.	0.364372	2.38E-14	postive
TET2	AC004584.	0.50849	2.07E-28	postive
TET3	AC004584.	0.402882	1.80E-17	postive
NSUN6	AC005785.	0.399434	3.56E-17	postive
RAD52	AC005785.	0.412897	2.37E-18	postive
NEIL1	AC005785.	0.62367	1.12E-45	postive
NSUN7	AC007546.	0.379184	1.68E-15	postive
TET1	AC007546.	0.502171	1.21E-27	postive
TET2	AC007546.	0.483752	1.70E-25	postive
TET3	AC007546.	0.32038	2.90E-11	postive
DNMT3A	AC084125.	0.329244	7.58E-12	postive
RAD52	AC084125.	0.341986	1.02E-12	postive
NSUN3	AC015849.	0.423453	2.59E-19	postive
NSUN7	AC015849.	0.328155	8.96E-12	postive

RAD52	AC015849.	0.348463	3.54E-13	postive
MECP2	AC015849.	0.340672	1.26E-12	postive
NEIL1	AC015849.	0.383328	7.79E-16	postive
UHRF2	AC015849.	0.337388	2.12E-12	postive
ZBTB33	AC015849.	0.381066	1.19E-15	postive
TET1	AC015849.	0.420918	4.44E-19	postive
TET2	AC015849.	0.63143	4.15E-47	postive
TET3	AC015849.	0.452519	3.86E-22	postive
DNMT3A	KCTD21-A	0.33841	1.81E-12	postive
NSUN3	AL031600.	0.462063	3.96E-23	postive
DNMT3A	AL031600.	0.387824	3.35E-16	postive
RAD52	AL031600.	0.375893	3.06E-15	postive
MBD1	AL031600.	0.330479	6.26E-12	postive
MECP2	AL031600.	0.353881	1.44E-13	postive
TET1	AL031600.	0.343463	8.02E-13	postive
TET2	AL031600.	0.431086	4.98E-20	postive
TET3	AL031600.	0.38226	9.50E-16	postive
NSUN3	TH2LCRR	0.433098	3.20E-20	postive
NSUN6	TH2LCRR	0.355866	1.03E-13	postive
TRDMT1	TH2LCRR	0.319217	3.45E-11	postive
UHRF2	TH2LCRR	0.307217	1.97E-10	postive
TET1	TH2LCRR	0.623664	1.12E-45	postive
TET2	TH2LCRR	0.562588	1.11E-35	postive
TET3	TH2LCRR	0.344072	7.26E-13	postive
NSUN6	U73166.1	0.32563	1.32E-11	postive
NSUN7	U73166.1	0.413484	2.10E-18	postive
DNMT3A	U73166.1	0.432957	3.30E-20	postive
RAD52	U73166.1	0.343642	7.79E-13	postive
NEIL1	U73166.1	0.476067	1.23E-24	postive
TET2	U73166.1	0.315645	5.84E-11	postive
TET3	U73166.1	0.337373	2.13E-12	postive
NSUN6	PCAT7	0.317781	4.27E-11	postive
NSUN7	PCAT7	0.304971	2.70E-10	postive
DNMT3A	PCAT7	0.479517	5.08E-25	postive
ZBTB33	PCAT7	0.403867	1.48E-17	postive
TET3	PCAT7	0.485556	1.06E-25	postive
TET1	AC079336.	0.47996	4.54E-25	postive
TET2	AC079336.	0.468516	8.14E-24	postive
NSUN3	RBMS3-AS	0.728138	4.38E-69	postive
TRDMT1	RBMS3-AS	0.453168	3.31E-22	postive
TET1	RBMS3-AS	0.335697	2.77E-12	postive
TET1	AL031666.	0.663764	1.53E-53	postive
TET2	AL031666.	0.469129	7.00E-24	postive
TET3	AL031666.	0.334897	3.15E-12	postive
NSUN5	AC048337.	0.346651	4.77E-13	postive
NTHL1	AC048337.	0.402893	1.80E-17	postive
NSUN6	AC073957.	0.484078	1.56E-25	postive
NSUN7	AC073957.	0.436111	1.64E-20	postive
RAD52	AC073957.	0.394456	9.42E-17	postive
MBD1	AC073957.	0.404045	1.43E-17	postive
NEIL1	AC073957.	0.397275	5.44E-17	postive
ZBTB33	AC073957.	0.315107	6.32E-11	postive
TET1	AC073957.	0.363914	2.58E-14	postive
TET2	AC073957.	0.434627	2.29E-20	postive
TET3	AC073957.	0.456888	1.37E-22	postive
NSUN3	C1QTNF7-	0.766192	1.45E-80	postive
TRDMT1	C1QTNF7-	0.471488	3.89E-24	postive
TET1	C1QTNF7-	0.407787	6.73E-18	postive

TET2	C1QTNF7-	0.392497	1.37E-16	postive
NSUN6	AC027243.	0.310745	1.19E-10	postive
DNMT3A	AC027243.	0.345043	6.20E-13	postive
RAD52	AC027243.	0.341523	1.10E-12	postive
NEIL1	AC027243.	0.365175	2.07E-14	postive
YBX1	AC098484.	0.654689	1.17E-51	postive
TET1	AC098484.	0.494129	1.09E-26	postive
TET2	AC098484.	0.370729	7.75E-15	postive
TET3	AC098484.	0.326642	1.13E-11	postive
NSUN6	AC142472.	0.346191	5.14E-13	postive
NSUN7	AC142472.	0.356929	8.57E-14	postive
MBD3	AC142472.	0.336424	2.47E-12	postive
NEIL1	AC142472.	0.356718	8.89E-14	postive
SMUG1	AC142472.	0.324724	1.51E-11	postive
TET1	CASC20	0.451514	4.89E-22	postive
NSUN3	JARID2-AS	0.517443	1.59E-29	postive
TET1	JARID2-AS	0.459104	8.08E-23	postive
TET2	JARID2-AS	0.45977	6.88E-23	postive
DNMT3A	AC008555.	0.416409	1.14E-18	postive
NSUN6	AC005790.	0.304649	2.83E-10	postive
NSUN3	AL133243.	0.468076	9.08E-24	postive
TET1	AL133243.	0.561566	1.57E-35	postive
TET2	AL133243.	0.53675	4.82E-32	postive
TET3	AL133243.	0.320281	2.95E-11	postive
NSUN3	AL162724.	0.715723	9.43E-66	postive
TRDMT1	AL162724.	0.448266	1.04E-21	postive
UHRF2	AL162724.	0.315573	5.90E-11	postive
ZBTB38	AL162724.	0.307291	1.95E-10	postive
TET1	AL162724.	0.42231	3.31E-19	postive
TET2	AL162724.	0.355026	1.18E-13	postive
NSUN6	AC106028.	0.343845	7.54E-13	postive
NSUN7	AC106028.	0.317356	4.54E-11	postive
RAD52	AC106028.	0.357739	7.47E-14	postive
NEIL1	AC106028.	0.45309	3.37E-22	postive
TET2	AC106028.	0.30783	1.80E-10	postive
NSUN3	AC100778.	0.319968	3.09E-11	postive
NSUN6	AC100778.	0.332814	4.36E-12	postive
RAD52	AC100778.	0.313661	7.80E-11	postive
MBD1	AC100778.	0.434857	2.17E-20	postive
NEIL1	AC100778.	0.306082	2.31E-10	postive
TET2	AC100778.	0.366411	1.66E-14	postive
TET3	AC100778.	0.320541	2.83E-11	postive
NEIL1	AC023302.	0.42731	1.13E-19	postive
SMUG1	AC023302.	0.353392	1.56E-13	postive
NSUN6	HDAC2-AS	0.514286	3.97E-29	postive
NSUN7	HDAC2-AS	0.43668	1.45E-20	postive
DNMT3A	HDAC2-AS	0.386574	4.24E-16	postive
RAD52	HDAC2-AS	0.347948	3.85E-13	postive
MBD1	HDAC2-AS	0.322452	2.13E-11	postive
NEIL1	HDAC2-AS	0.323629	1.78E-11	postive
ZBTB33	HDAC2-AS	0.378136	2.03E-15	postive
TET1	HDAC2-AS	0.357267	8.09E-14	postive
TET2	HDAC2-AS	0.381638	1.07E-15	postive
TET3	HDAC2-AS	0.433624	2.85E-20	postive
NSUN6	AL158834.	0.51704	1.79E-29	postive
NSUN7	AL158834.	0.371892	6.30E-15	postive
RAD52	AL158834.	0.411113	3.42E-18	postive
NEIL1	AL158834.	0.435928	1.71E-20	postive

TET1	AL158834.	0.503	9.64E-28	postive
TET2	AL158834.	0.406345	9.00E-18	postive
TET3	AL158834.	0.308824	1.57E-10	postive
NSUN3	RMRP	0.368294	1.19E-14	postive
TET1	RMRP	0.396616	6.19E-17	postive
TET2	RMRP	0.419476	6.02E-19	postive
DNMT3B	AP000442.	0.34112	1.17E-12	postive
RAD52	AP000442.	0.498053	3.76E-27	postive
TET2	AP000442.	0.333934	3.66E-12	postive
TET3	AP000442.	0.388143	3.15E-16	postive
NSUN3	AC011337.	0.321447	2.47E-11	postive
TET2	AC011337.	0.411853	2.94E-18	postive
TET1	AC104041.	0.488834	4.48E-26	postive
TET2	AC104041.	0.456902	1.37E-22	postive
TET3	AC104041.	0.356455	9.29E-14	postive
NSUN3	AC011466.	0.395587	7.56E-17	postive
NSUN7	AC011466.	0.310126	1.30E-10	postive
DNMT3A	AC011466.	0.315869	5.65E-11	postive
TRDMT1	AC011466.	0.314591	6.81E-11	postive
RAD52	AC011466.	0.40566	1.03E-17	postive
MECP2	AC011466.	0.313115	8.45E-11	postive
NEIL1	AC011466.	0.326362	1.18E-11	postive
UHRF2	AC011466.	0.308335	1.68E-10	postive
TET1	AC011466.	0.418366	7.60E-19	postive
TET2	AC011466.	0.438101	1.05E-20	postive
TET3	AC011466.	0.398036	4.69E-17	postive
TET1	AC073389.	0.325966	1.25E-11	postive
RAD52	AC024451.	0.345202	6.04E-13	postive
TET1	AC024451.	0.305136	2.64E-10	postive
TET3	AC005034.	0.316376	5.25E-11	postive
NSUN3	AC007390.	0.361095	4.20E-14	postive
NSUN6	AC007390.	0.347729	3.99E-13	postive
NSUN7	AC007390.	0.323623	1.78E-11	postive
RAD52	AC007390.	0.343351	8.16E-13	postive
NEIL1	AC007390.	0.339234	1.58E-12	postive
UHRF2	AC007390.	0.332243	4.76E-12	postive
ZBTB33	AC007390.	0.320333	2.92E-11	postive
TET1	AC007390.	0.548067	1.35E-33	postive
TET2	AC007390.	0.638582	1.82E-48	postive
TET3	AC007390.	0.44624	1.66E-21	postive
TET1	AC009948.	0.497502	4.37E-27	postive
TET3	AC009948.	0.321905	2.31E-11	postive
NSUN7	AC006213.	0.315494	5.97E-11	postive
DNMT3A	AC006213.	0.325767	1.29E-11	postive
TET3	AC006213.	0.338042	1.91E-12	postive
NSUN3	AC092171.	0.566842	2.61E-36	postive
TRDMT1	AC092171.	0.38418	6.65E-16	postive
UHRF2	AC092171.	0.342573	9.26E-13	postive
TET1	AC092171.	0.657653	2.89E-52	postive
TET2	AC092171.	0.593253	1.97E-40	postive
TET3	AC092171.	0.342012	1.01E-12	postive
NSUN6	AC006001.	0.381699	1.05E-15	postive
NSUN7	AC006001.	0.314717	6.69E-11	postive
TET1	AC006001.	0.511333	9.26E-29	postive
TET2	AC006001.	0.391029	1.82E-16	postive
TET3	AC006001.	0.374956	3.63E-15	postive
NSUN6	LINC02604	0.521416	4.98E-30	postive
RAD52	LINC02604	0.383986	6.89E-16	postive

NEIL1	LINC0260	0.554593	1.61E-34	postive
NSUN6	AP001462.	0.357881	7.29E-14	postive
NSUN7	AP001462.	0.32047	2.86E-11	postive
RAD52	AP001462.	0.362349	3.38E-14	postive
MBD1	AP001462.	0.348453	3.55E-13	postive
NEIL1	AP001462.	0.402236	2.05E-17	postive
NSUN3	ADAMTSL	0.534844	8.69E-32	postive
UHRF2	ADAMTSL	0.320904	2.68E-11	postive
ZBTB38	ADAMTSL	0.309279	1.47E-10	postive
TET1	ADAMTSL	0.537262	4.11E-32	postive
TET2	ADAMTSL	0.58577	3.17E-39	postive
TET3	ADAMTSL	0.304421	2.92E-10	postive
NSUN3	KLF7-IT1	0.657226	3.54E-52	postive
TRDMT1	KLF7-IT1	0.454348	2.51E-22	postive
UHRF2	KLF7-IT1	0.35606	9.94E-14	postive
ZBTB38	KLF7-IT1	0.31626	5.34E-11	postive
TET1	KLF7-IT1	0.613803	6.51E-44	postive
TET2	KLF7-IT1	0.552068	3.68E-34	postive
NSUN4	AL021878.	0.306741	2.11E-10	postive
NSUN6	AL021878.	0.423092	2.80E-19	postive
NSUN7	AL021878.	0.450918	5.62E-22	postive
DNMT3A	AL021878.	0.407853	6.64E-18	postive
RAD52	AL021878.	0.36507	2.11E-14	postive
MBD1	AL021878.	0.396321	6.56E-17	postive
MECP2	AL021878.	0.307623	1.86E-10	postive
NEIL1	AL021878.	0.30298	3.57E-10	postive
TDG	AL021878.	0.372019	6.15E-15	postive
ZBTB33	AL021878.	0.354457	1.30E-13	postive
TET1	AL021878.	0.536674	4.94E-32	postive
TET2	AL021878.	0.459431	7.47E-23	postive
TET3	AL021878.	0.472575	2.96E-24	postive
NSUN7	SEPTIN7-D	0.44463	2.40E-21	postive
DNMT3A	SEPTIN7-D	0.326194	1.21E-11	postive
MECP2	SEPTIN7-D	0.383031	8.23E-16	postive
ZBTB33	SEPTIN7-D	0.361824	3.70E-14	postive
TET1	SEPTIN7-D	0.387155	3.80E-16	postive
TET3	SEPTIN7-D	0.305978	2.34E-10	postive
MBD1	ID2-AS1	0.369737	9.25E-15	postive
NSUN7	STK24-AS	0.35675	8.84E-14	postive
NTHL1	ZFAS1	0.35045	2.55E-13	postive
ZBTB38	ZFAS1	-0.3347	3.24E-12	negative
NEIL1	EPS15-AS	0.300907	4.77E-10	postive
TET2	EPS15-AS	0.307392	1.92E-10	postive
NSUN7	AC010761.	0.353173	1.62E-13	postive
DNMT3B	AC010761.	0.312581	9.12E-11	postive
NSUN6	AC010326.	0.394011	1.03E-16	postive
NSUN7	AC010326.	0.315525	5.94E-11	postive
DNMT3A	AC010326.	0.306938	2.05E-10	postive
RAD52	AC010326.	0.461803	4.21E-23	postive
NEIL1	AC010326.	0.468349	8.49E-24	postive
SMUG1	AC010326.	0.301954	4.12E-10	postive
NSUN3	AC087286.	0.638622	1.79E-48	postive
TRDMT1	AC087286.	0.410586	3.81E-18	postive
UHRF2	AC087286.	0.353245	1.60E-13	postive
ZBTB38	AC087286.	0.317566	4.40E-11	postive
TET1	AC087286.	0.626945	2.82E-46	postive
TET2	AC087286.	0.582756	9.51E-39	postive
TET3	AC087286.	0.300422	5.10E-10	postive

UHRF2	GAS6-DT	0.300833	4.81E-10	postive
ZBTB38	GAS6-DT	0.323306	1.87E-11	postive
NSUN6	MIR4435-2	-0.34901	3.23E-13	negative
MBD1	MIR4435-2	-0.30106	4.66E-10	negative
TET3	MIR4435-2	-0.3114	1.08E-10	negative
NSUN5	AC109322.	0.306931	2.05E-10	postive
DNMT3A	AC109322.	0.342335	9.62E-13	postive
MBD3	AC109322.	0.377599	2.24E-15	postive
NTHL1	AC109322.	0.388703	2.84E-16	postive
NEIL1	SPINT1-AS	0.372748	5.40E-15	postive
SMUG1	SPINT1-AS	0.41716	9.79E-19	postive
NSUN6	AC079160.	0.349901	2.79E-13	postive
NSUN7	AC079160.	0.317783	4.27E-11	postive
RAD52	AC079160.	0.323801	1.74E-11	postive
ZBTB33	AC079160.	0.35018	2.66E-13	postive
TET1	AC079160.	0.540452	1.52E-32	postive
TET2	AC079160.	0.401499	2.37E-17	postive
TET3	AC079160.	0.448683	9.45E-22	postive
NSUN6	EIF2B5-DT	0.303424	3.36E-10	postive
NSUN3	AC010973.	0.551966	3.80E-34	postive
ZBTB38	AC010973.	0.309357	1.45E-10	postive
TET1	AC010973.	0.399988	3.20E-17	postive
TET2	AC010973.	0.541729	1.02E-32	postive
TET3	AC010973.	0.393101	1.22E-16	postive
NSUN5	SNHG17	0.383888	7.02E-16	postive
MBD3	SNHG17	0.426615	1.32E-19	postive
ZBTB4	SNHG17	-0.30401	3.09E-10	negative
NSUN6	AC008764.	0.329522	7.26E-12	postive
RAD52	AC008764.	0.337708	2.02E-12	postive
MBD3	AC008764.	0.363476	2.78E-14	postive
NEIL1	AC008764.	0.542555	7.82E-33	postive
NSUN3	AC018766.	0.300382	5.12E-10	postive
NSUN6	AC018766.	0.399638	3.42E-17	postive
NSUN7	AC018766.	0.425952	1.52E-19	postive
DNMT3A	AC018766.	0.380991	1.20E-15	postive
RAD52	AC018766.	0.382865	8.49E-16	postive
MECP2	AC018766.	0.319451	3.33E-11	postive
NEIL1	AC018766.	0.359157	5.86E-14	postive
UHRF2	AC018766.	0.329038	7.82E-12	postive
ZBTB33	AC018766.	0.347781	3.96E-13	postive
TET1	AC018766.	0.502358	1.15E-27	postive
TET2	AC018766.	0.492755	1.58E-26	postive
TET3	AC018766.	0.495233	8.09E-27	postive
NSUN6	AL162274.	0.372649	5.50E-15	postive
RAD52	AL162274.	0.377175	2.42E-15	postive
NEIL1	AL162274.	0.43452	2.34E-20	postive
SMUG1	AL162274.	0.302021	4.08E-10	postive
NEIL1	AP000553.	0.393781	1.07E-16	postive
TET1	AC009686.	0.337669	2.03E-12	postive
NSUN3	AC022973.	0.525675	1.41E-30	postive
TRDMT1	AC022973.	0.342998	8.64E-13	postive
UHRF2	AC022973.	0.312695	8.97E-11	postive
TET1	AC022973.	0.696236	7.25E-61	postive
TET2	AC022973.	0.558925	3.82E-35	postive
TET3	AC022973.	0.360286	4.83E-14	postive
NEIL1	AC073349.	0.362305	3.41E-14	postive
TET2	AC073349.	0.354868	1.22E-13	postive
ZBTB33	AL157400.	0.310137	1.30E-10	postive

TET1	AL157400.	0.541245	1.18E-32	postive
TET2	AL157400.	0.389322	2.52E-16	postive
TET3	AL157400.	0.329381	7.42E-12	postive
NSUN5	SNHG10	0.317249	4.62E-11	postive
NSUN6	SNHG10	0.386544	4.27E-16	postive
NSUN7	SNHG10	0.308475	1.65E-10	postive
NEIL1	SNHG10	0.361261	4.08E-14	postive
NTHL1	SNHG10	0.379224	1.66E-15	postive
SMUG1	SNHG10	0.315936	5.60E-11	postive
NSUN7	DNAJC3-D	0.466818	1.24E-23	postive
DNMT3A	DNAJC3-D	0.436411	1.54E-20	postive
RAD52	DNAJC3-D	0.325729	1.30E-11	postive
TDG	DNAJC3-D	0.321809	2.34E-11	postive
ZBTB33	DNAJC3-D	0.303008	3.56E-10	postive
TET1	DNAJC3-D	0.352267	1.88E-13	postive
TET3	DNAJC3-D	0.351004	2.32E-13	postive
NSUN6	AC068196.	0.41293	2.35E-18	postive
RAD52	AC068196.	0.401916	2.18E-17	postive
NEIL1	AC068196.	0.404172	1.39E-17	postive
TET1	AC068196.	0.37475	3.76E-15	postive
NSUN3	AC108727.	0.444833	2.29E-21	postive
UHRF2	AC108727.	0.312549	9.17E-11	postive
ZBTB38	AC108727.	0.308873	1.55E-10	postive
TET1	AC108727.	0.533725	1.23E-31	postive
TET2	AC108727.	0.641624	4.71E-49	postive
TET3	AC108727.	0.354557	1.28E-13	postive
NSUN3	AC020891.	0.358982	6.04E-14	postive
UHRF2	AC020891.	0.319712	3.21E-11	postive
TET1	AC020891.	0.461271	4.79E-23	postive
TET2	AC020891.	0.642505	3.17E-49	postive
TET3	AC020891.	0.344591	6.68E-13	postive
NEIL1	AL358334.	0.318964	3.58E-11	postive
TET1	AL358334.	0.334536	3.33E-12	postive
TET2	AL358334.	0.542892	7.03E-33	postive
NSUN6	AL139123.	0.45058	6.08E-22	postive
RAD52	AL139123.	0.420488	4.86E-19	postive
NEIL1	AL139123.	0.597586	3.82E-41	postive
NSUN6	CAPN10-D	0.440172	6.62E-21	postive
NSUN7	CAPN10-D	0.35412	1.38E-13	postive
DNMT3A	CAPN10-D	0.339712	1.47E-12	postive
RAD52	CAPN10-D	0.447005	1.39E-21	postive
NEIL1	CAPN10-D	0.545948	2.66E-33	postive
TET1	CAPN10-D	0.37965	1.54E-15	postive
TET2	CAPN10-D	0.366308	1.69E-14	postive
DNMT1	AC116914.	0.338966	1.65E-12	postive
MBD4	AC116914.	0.302254	3.95E-10	postive
UHRF1	AC116914.	0.332037	4.92E-12	postive
NSUN7	MAST4-AS	0.38447	6.30E-16	postive
NSUN3	AC121764.	0.44448	2.49E-21	postive
TET1	AC121764.	0.572011	4.36E-37	postive
TET2	AC121764.	0.381575	1.08E-15	postive
MBD4	C2CD4D-A	0.318479	3.85E-11	postive
NSUN6	MIR600HG	0.315909	5.62E-11	postive
NSUN7	MIR600HG	0.414032	1.87E-18	postive
DNMT3A	MIR600HG	0.441068	5.41E-21	postive
RAD52	MIR600HG	0.386655	4.18E-16	postive
MBD1	MIR600HG	0.3507	2.44E-13	postive
MECP2	MIR600HG	0.361688	3.79E-14	postive

ZBTB33	MIR600HG	0.371821	6.38E-15	postive
TET1	MIR600HG	0.410196	4.12E-18	postive
TET3	MIR600HG	0.481001	3.47E-25	postive
TET1	AL023755.	0.375443	3.32E-15	postive
TET2	AL023755.	0.511537	8.73E-29	postive
NSUN6	Z93930.3	0.318523	3.82E-11	postive
RAD52	Z93930.3	0.337551	2.07E-12	postive
TET1	Z93930.3	0.477428	8.68E-25	postive
TET2	Z93930.3	0.301013	4.70E-10	postive
TET3	Z93930.3	0.339136	1.61E-12	postive
NSUN5	AC005076.	0.344108	7.22E-13	postive
NTHL1	AC005076.	0.362032	3.57E-14	postive
NEIL1	SLBP-DT	0.352972	1.67E-13	postive
SMUG1	SLBP-DT	0.30555	2.49E-10	postive
NSUN6	AL353796.	0.320942	2.67E-11	postive
DNMT3A	AL353796.	0.355533	1.09E-13	postive
ZBTB33	AL353796.	0.313251	8.28E-11	postive
TET3	AL353796.	0.446062	1.73E-21	postive
MBD3	AL355385.	0.304464	2.90E-10	postive
NEIL1	AL355385.	0.315549	5.92E-11	postive
NSUN6	AC010491.	0.414521	1.69E-18	postive
NSUN7	AC010491.	0.333593	3.86E-12	postive
RAD52	AC010491.	0.42618	1.44E-19	postive
NEIL1	AC010491.	0.39255	1.36E-16	postive
NSUN6	AL513218.	0.445354	2.04E-21	postive
RAD52	AL513218.	0.427994	9.77E-20	postive
NEIL1	AL513218.	0.526417	1.13E-30	postive
NSUN7	AC011005.	0.305707	2.44E-10	postive
DNMT3A	AC011005.	0.451537	4.86E-22	postive
RAD52	AC011005.	0.300961	4.73E-10	postive
TET1	AC011005.	0.347053	4.46E-13	postive
TET3	AC011005.	0.336121	2.59E-12	postive
DNMT3A	LOH12CR2	0.320184	2.99E-11	postive
DNMT3A	AC040160.	0.31818	4.02E-11	postive
NSUN3	RNF216-IT	0.578605	4.24E-38	postive
TRDMT1	RNF216-IT	0.405256	1.12E-17	postive
UHRF2	RNF216-IT	0.358759	6.27E-14	postive
ZBTB38	RNF216-IT	0.307908	1.78E-10	postive
TET1	RNF216-IT	0.61624	2.42E-44	postive
TET2	RNF216-IT	0.549741	7.84E-34	postive
NSUN3	AC096992.	0.503738	7.85E-28	postive
NSUN7	AC096992.	0.318013	4.12E-11	postive
TRDMT1	AC096992.	0.307914	1.78E-10	postive
RAD52	AC096992.	0.306944	2.05E-10	postive
MBD4	AC096992.	0.341045	1.18E-12	postive
MECP2	AC096992.	0.350405	2.57E-13	postive
ZBTB33	AC096992.	0.30754	1.88E-10	postive
TET1	AC096992.	0.391619	1.63E-16	postive
TET2	AC096992.	0.364961	2.15E-14	postive
TET3	AC096992.	0.311744	1.03E-10	postive
YBX1	AL603839.	0.791511	1.71E-89	postive
NEIL1	AL358472.	0.311744	1.03E-10	postive
NEIL1	AC012173.	0.315339	6.11E-11	postive
TET2	MMADHC	0.319086	3.52E-11	postive
NSUN6	AC090044.	0.300558	5.00E-10	postive
MBD1	AC090044.	0.358224	6.88E-14	postive
NSUN6	AC135050.	0.336466	2.46E-12	postive
DNMT3A	AC135050.	0.309772	1.37E-10	postive

RAD52	AC135050.	0.39421	9.87E-17	postive
NEIL1	AC135050.	0.426808	1.26E-19	postive
ZBTB33	AC135050.	0.306927	2.05E-10	postive
TET2	AC135050.	0.41192	2.90E-18	postive
TET3	AC135050.	0.363641	2.70E-14	postive
ZBTB4	AC036108.	0.306851	2.07E-10	postive
NSUN3	LINC01915	0.56586	3.66E-36	postive
NSUN7	AL021391.	0.363188	2.92E-14	postive
NSUN7	AL451085.	0.410407	3.95E-18	postive
DNMT3A	AL451085.	0.347055	4.46E-13	postive
TET3	AL451085.	0.320699	2.77E-11	postive
NSUN6	AC006566.	0.334253	3.48E-12	postive
NSUN7	AC006566.	0.337431	2.11E-12	postive
TET1	AC006566.	0.637468	2.98E-48	postive
TET2	AC006566.	0.393916	1.05E-16	postive
TET3	AC006566.	0.345816	5.47E-13	postive
NSUN6	AC137630.	0.366792	1.56E-14	postive
NSUN7	AC137630.	0.477192	9.21E-25	postive
RAD52	AC137630.	0.317494	4.45E-11	postive
NEIL1	AC137630.	0.395601	7.54E-17	postive
TET1	AC137630.	0.432046	4.04E-20	postive
TET2	AC137630.	0.373093	5.07E-15	postive
TET3	AC137630.	0.303	3.56E-10	postive
ZBTB33	AL360270.	0.306169	2.28E-10	postive
TET2	AL360270.	0.357143	8.27E-14	postive
TET3	AL360270.	0.342461	9.43E-13	postive
NSUN7	AP000255.	0.345932	5.36E-13	postive
DNMT3A	AP000255.	0.343736	7.67E-13	postive
NSUN3	AL512770.	0.355247	1.14E-13	postive
NSUN6	AL512770.	0.456506	1.50E-22	postive
DNMT3A	AL512770.	0.352082	1.94E-13	postive
RAD52	AL512770.	0.422317	3.30E-19	postive
MBD1	AL512770.	0.341304	1.14E-12	postive
NEIL1	AL512770.	0.452055	4.30E-22	postive
TET2	AL512770.	0.347507	4.14E-13	postive
TET3	AL512770.	0.333182	4.12E-12	postive
RAD52	AC026333.	0.309327	1.46E-10	postive
NEIL1	AC026333.	0.319852	3.14E-11	postive
NSUN3	AC145423.	0.3408	1.23E-12	postive
NSUN6	AC145423.	0.351209	2.25E-13	postive
RAD52	AC145423.	0.364797	2.21E-14	postive
NEIL1	AC145423.	0.353877	1.44E-13	postive
TET1	AC145423.	0.479769	4.77E-25	postive
TET2	AC145423.	0.465662	1.65E-23	postive
TET3	AC145423.	0.316691	5.01E-11	postive
NSUN6	C21orf62-.	0.391906	1.54E-16	postive
NSUN7	C21orf62-.	0.456681	1.44E-22	postive
ZBTB33	C21orf62-.	0.332026	4.93E-12	postive
TET1	C21orf62-.	0.506376	3.76E-28	postive
TET2	C21orf62-.	0.461229	4.84E-23	postive
TET3	C21orf62-.	0.40953	4.72E-18	postive
NSUN6	AC034236.	0.365347	2.01E-14	postive
NEIL1	AC034236.	0.557235	6.70E-35	postive
NSUN3	AC004803.	0.311	1.15E-10	postive
RAD52	AC004803.	0.367271	1.43E-14	postive
ZBTB38	AC004803.	0.308727	1.59E-10	postive
NSUN7	AC024560.	0.352382	1.85E-13	postive
TET1	AC024560.	0.372305	5.85E-15	postive

DNMT3A	AL645608.	0.324532	1.56E-11	postive
DNMT3A	AL592424.	0.361819	3.71E-14	postive
NEIL1	AC010331.	0.339172	1.60E-12	postive
NTHL1	AC010331.	0.302488	3.83E-10	postive
NSUN6	AC245884.	0.459071	8.15E-23	postive
NSUN7	AC245884.	0.306496	2.18E-10	postive
RAD52	AC245884.	0.476246	1.17E-24	postive
NEIL1	AC245884.	0.526261	1.18E-30	postive
TET1	AC245884.	0.370174	8.56E-15	postive
TET2	AC245884.	0.354414	1.31E-13	postive
NSUN7	AC008781.	0.32127	2.54E-11	postive
TET2	AC008781.	0.308887	1.55E-10	postive
NEIL1	AC009061.	0.308087	1.74E-10	postive
SMUG1	AC009061.	0.349078	3.20E-13	postive
RAD52	AL390066.	0.337593	2.06E-12	postive
NEIL1	AL390066.	0.446002	1.75E-21	postive
ZBTB33	LINC01001	0.304794	2.77E-10	postive
TET2	LINC01001	0.341139	1.17E-12	postive
NSUN3	AL513327.	0.512025	7.60E-29	postive
NSUN6	AL513327.	0.300081	5.34E-10	postive
TRDMT1	AL513327.	0.338969	1.65E-12	postive
MECP2	AL513327.	0.328522	8.47E-12	postive
UHRF2	AL513327.	0.346571	4.83E-13	postive
TET1	AL513327.	0.563332	8.65E-36	postive
TET2	AL513327.	0.6012	9.52E-42	postive
TET3	AL513327.	0.379558	1.57E-15	postive
NSUN7	AL137060.	0.353243	1.60E-13	postive
DNMT3A	AL137060.	0.31774	4.29E-11	postive
NSUN7	AC109460.	0.307641	1.85E-10	postive
DNMT3A	AC109460.	0.324957	1.46E-11	postive
RAD52	AC109460.	0.332222	4.78E-12	postive
NEIL1	AC109460.	0.330505	6.24E-12	postive
TET2	AC109460.	0.305098	2.65E-10	postive
NSUN6	AL359878.	0.387654	3.46E-16	postive
NSUN7	AL359878.	0.428524	8.71E-20	postive
RAD52	AL359878.	0.322713	2.05E-11	postive
TET1	AL359878.	0.680199	3.95E-57	postive
TET2	AL359878.	0.402085	2.11E-17	postive
TET3	AL359878.	0.428498	8.76E-20	postive
TET1	AC072039.	0.348913	3.29E-13	postive
NSUN5	ZNF232-A	0.323976	1.69E-11	postive
DNMT1	ZNF232-A	0.317079	4.73E-11	postive
ALYREF	ZNF232-A	0.39015	2.15E-16	postive
MBD3	ZNF232-A	0.330244	6.50E-12	postive
NSUN6	AC131235.	0.342512	9.35E-13	postive
NSUN7	AC131235.	0.304913	2.72E-10	postive
SMUG1	AC131235.	0.300786	4.85E-10	postive
TET1	TMEM202-	0.315886	5.64E-11	postive
TET2	TMEM202-	0.53775	3.53E-32	postive
NSUN6	AC016888.	-0.30609	2.31E-10	negative
MBD2	AC016888.	0.356793	8.77E-14	postive
MBD4	AC016888.	0.300702	4.90E-10	postive
NSUN3	AC100778.	0.410907	3.56E-18	postive
NSUN6	AC100778.	0.365331	2.01E-14	postive
DNMT3A	AC100778.	0.377736	2.19E-15	postive
RAD52	AC100778.	0.427856	1.01E-19	postive
MBD1	AC100778.	0.472259	3.21E-24	postive
MECP2	AC100778.	0.302467	3.84E-10	postive

NEIL1	AC100778.	0.315009	6.41E-11	postive
ZBTB33	AC100778.	0.353405	1.56E-13	postive
TET1	AC100778.	0.335964	2.66E-12	postive
TET2	AC100778.	0.484462	1.41E-25	postive
TET3	AC100778.	0.425471	1.68E-19	postive
NEIL1	AL359636.	0.401516	2.36E-17	postive
NSUN6	SUV39H2-	0.306089	2.31E-10	postive
NSUN6	AC027796.	0.309835	1.35E-10	postive
RAD52	AC027796.	0.319912	3.11E-11	postive
MBD1	AC027796.	0.308136	1.73E-10	postive
NEIL1	AC027796.	0.323953	1.70E-11	postive
NSUN3	AC068790.	0.382244	9.53E-16	postive
UHRF2	AC068790.	0.344228	7.08E-13	postive
TET1	AC068790.	0.62733	2.39E-46	postive
TET2	AC068790.	0.595051	1.00E-40	postive
TET3	AC068790.	0.34933	3.07E-13	postive
NSUN6	AC002059.	0.36682	1.55E-14	postive
NSUN3	LINC02595	0.515656	2.68E-29	postive
TRDMT1	LINC02595	0.350746	2.43E-13	postive
UHRF2	LINC02595	0.300413	5.10E-10	postive
NSUN5	LINC01952	0.377636	2.23E-15	postive
ALYREF	LINC01952	0.308461	1.65E-10	postive
NTHL1	LINC01952	0.346285	5.06E-13	postive
NTHL1	AL445228.	0.332036	4.92E-12	postive
TRDMT1	AC092614.	0.30427	2.98E-10	postive
ZBTB33	AC092614.	0.305332	2.57E-10	postive
TET1	AC092614.	0.3964	6.46E-17	postive
DNMT1	AC012073.	0.329215	7.61E-12	postive
DNMT3A	AC012073.	0.552248	3.47E-34	postive
DNMT3B	AC012073.	0.360067	5.01E-14	postive
UNG	AC012073.	0.312554	9.16E-11	postive
TET1	AC012073.	0.343873	7.50E-13	postive
TET3	AC012073.	0.414426	1.73E-18	postive
NSUN6	AC068724.	0.347252	4.32E-13	postive
RAD52	AC068724.	0.354139	1.37E-13	postive
NEIL1	AC068724.	0.453383	3.15E-22	postive
NSUN3	LINC-PINT	0.551335	4.67E-34	postive
TRDMT1	LINC-PINT	0.358933	6.09E-14	postive
UHRF2	LINC-PINT	0.383498	7.55E-16	postive
ZBTB38	LINC-PINT	0.340567	1.28E-12	postive
TET1	LINC-PINT	0.535493	7.11E-32	postive
TET2	LINC-PINT	0.516069	2.37E-29	postive
NSUN6	AL731569.	0.433467	2.95E-20	postive
NSUN7	AL731569.	0.394813	8.79E-17	postive
RAD52	AL731569.	0.390237	2.12E-16	postive
NEIL1	AL731569.	0.454607	2.36E-22	postive
TET1	AL731569.	0.403531	1.58E-17	postive
NSUN3	AC020978.	0.40711	7.71E-18	postive
NSUN6	AC020978.	0.363723	2.66E-14	postive
RAD52	AC020978.	0.372155	6.01E-15	postive
NEIL1	AC020978.	0.442926	3.55E-21	postive
TET1	AC020978.	0.407102	7.73E-18	postive
TET2	AC020978.	0.473777	2.19E-24	postive
NSUN3	AC099811.	0.567737	1.92E-36	postive
TRDMT1	AC099811.	0.35811	7.01E-14	postive
UHRF2	AC099811.	0.36515	2.08E-14	postive
ZBTB38	AC099811.	0.330993	5.79E-12	postive
TET1	AC099811.	0.592825	2.32E-40	postive

TET2	AC099811.	0.620566	4.09E-45	postive
TET3	AC099811.	0.311626	1.05E-10	postive
NSUN7	AC008763.	0.349822	2.83E-13	postive
NSUN6	AL008633.	0.34533	5.92E-13	postive
NSUN7	AL008633.	0.33048	6.26E-12	postive
ZBTB33	AL008633.	0.335275	2.96E-12	postive
TET1	AL008633.	0.529596	4.32E-31	postive
TET2	AL008633.	0.362658	3.21E-14	postive
TET3	AL008633.	0.378456	1.92E-15	postive
NSUN3	AC078852.	0.399789	3.32E-17	postive
NSUN6	AC078852.	0.307194	1.97E-10	postive
TET1	AC078852.	0.675394	4.67E-56	postive
TET2	AC078852.	0.461809	4.21E-23	postive
TET3	AC078852.	0.313761	7.69E-11	postive
NSUN6	AC104825.	0.402548	1.92E-17	postive
NSUN7	AC104825.	0.367057	1.49E-14	postive
NEIL1	AC104825.	0.351463	2.15E-13	postive
TET1	LINC0246€	0.385162	5.53E-16	postive
TET2	LINC0246€	0.379946	1.46E-15	postive
NSUN3	AL162727.	0.329564	7.21E-12	postive
NSUN6	AL162727.	0.313538	7.94E-11	postive
NSUN7	AL162727.	0.34548	5.77E-13	postive
UHRF2	AL162727.	0.315002	6.42E-11	postive
ZBTB33	AL162727.	0.30934	1.45E-10	postive
TET1	AL162727.	0.582944	8.88E-39	postive
TET2	AL162727.	0.562846	1.02E-35	postive
TET3	AL162727.	0.375965	3.02E-15	postive
TET1	AC104117.	0.30632	2.23E-10	postive
NSUN2	AC034231.	0.337998	1.93E-12	postive
NSUN7	AC034231.	0.33092	5.85E-12	postive
DNMT3A	AC034231.	0.3429	8.78E-13	postive
RAD52	AC034231.	0.303029	3.55E-10	postive
TET3	AC034231.	0.335208	3.00E-12	postive
NSUN3	AL133243.	0.524046	2.29E-30	postive
TRDMT1	AL133243.	0.337403	2.12E-12	postive
UHRF2	AL133243.	0.345183	6.06E-13	postive
ZBTB38	AL133243.	0.301261	4.54E-10	postive
TET1	AL133243.	0.611218	1.84E-43	postive
TET2	AL133243.	0.619661	5.95E-45	postive
TET3	AL133243.	0.380274	1.37E-15	postive
NSUN3	AC010998.	0.368404	1.17E-14	postive
NSUN6	AC010998.	0.321542	2.44E-11	postive
RAD52	AC010998.	0.371687	6.53E-15	postive
ZBTB33	AC010998.	0.310448	1.24E-10	postive
TET1	AC010998.	0.352223	1.90E-13	postive
TET2	AC010998.	0.504993	5.54E-28	postive
TET3	AC010998.	0.391763	1.58E-16	postive
NSUN3	AC022001.	0.471337	4.04E-24	postive
TRDMT1	AC022001.	0.327006	1.07E-11	postive
UHRF2	AC022001.	0.350166	2.67E-13	postive
TET1	AC022001.	0.563336	8.64E-36	postive
TET2	AC022001.	0.566348	3.09E-36	postive
NSUN7	NR2F2-AS	0.308315	1.68E-10	postive
TET1	NR2F2-AS	0.591867	3.32E-40	postive
TET2	NR2F2-AS	0.505269	5.13E-28	postive
TET3	NR2F2-AS	0.346916	4.57E-13	postive
NEIL1	AC010997.	0.329517	7.27E-12	postive
NSUN6	AC010319.	0.304456	2.91E-10	postive

NSUN7	AC010319.	0.357616	7.63E-14	postive
RAD52	AC010319.	0.32071	2.76E-11	postive
MBD1	AC010319.	0.305218	2.61E-10	postive
NEIL1	AC010319.	0.434091	2.57E-20	postive
TET2	AC010319.	0.352001	1.97E-13	postive
NSUN6	AC145285.	0.302202	3.98E-10	postive
RAD52	AC145285.	0.361745	3.75E-14	postive
NEIL1	AC145285.	0.436922	1.37E-20	postive
NSUN7	AC067852.	0.327092	1.05E-11	postive
ZBTB33	AC023024.	0.346601	4.81E-13	postive
TET1	AC023024.	0.440275	6.47E-21	postive
TET2	AC023024.	0.35162	2.10E-13	postive
TET3	AC023024.	0.526795	1.01E-30	postive
NSUN6	AC092828.	0.321326	2.52E-11	postive
NSUN7	AC092828.	0.303797	3.19E-10	postive
TET1	AC092828.	0.679299	6.29E-57	postive
TET2	AC092828.	0.459884	6.70E-23	postive
TET3	AC092828.	0.348791	3.35E-13	postive
DNMT3A	AC013400.	0.365015	2.13E-14	postive
NSUN3	AC087294.	0.340256	1.34E-12	postive
NSUN6	AC087294.	0.328686	8.26E-12	postive
NSUN7	AC087294.	0.343374	8.13E-13	postive
UHRF2	AC087294.	0.305912	2.37E-10	postive
ZBTB33	AC087294.	0.326041	1.24E-11	postive
TET1	AC087294.	0.661956	3.68E-53	postive
TET2	AC087294.	0.536186	5.74E-32	postive
TET3	AC087294.	0.401337	2.45E-17	postive
MBD3	AC048341.	0.318081	4.08E-11	postive
NEIL1	AC048341.	0.306421	2.20E-10	postive
NSUN6	AL139021.	0.340009	1.40E-12	postive
NSUN7	AL139021.	0.408086	6.33E-18	postive
MECP2	AL139021.	0.31582	5.69E-11	postive
ZBTB33	AL139021.	0.332417	4.64E-12	postive
TET1	AL139021.	0.545332	3.24E-33	postive
TET2	AL139021.	0.495833	6.88E-27	postive
TET3	AL139021.	0.352486	1.81E-13	postive
NSUN6	AC034102.	0.366379	1.67E-14	postive
NSUN7	AC034102.	0.340646	1.26E-12	postive
TET1	AC034102.	0.645027	1.02E-49	postive
TET2	AC034102.	0.366093	1.76E-14	postive
TET3	AC034102.	0.368778	1.10E-14	postive
NEIL1	GAS5	0.408229	6.15E-18	postive
ZBTB38	GAS5	-0.30162	4.32E-10	negative
NSUN6	AC016027.	0.417945	8.30E-19	postive
NEIL1	AC016027.	0.318907	3.61E-11	postive
NSUN6	ARHGEF2-	0.340423	1.31E-12	postive
NSUN7	ARHGEF2-	0.405484	1.07E-17	postive
RAD52	ARHGEF2-	0.345001	6.24E-13	postive
NEIL1	ARHGEF2-	0.330444	6.30E-12	postive
TET1	ARHGEF2-	0.52189	4.33E-30	postive
NSUN3	AC139887.	0.331507	5.34E-12	postive
NSUN6	AC139887.	0.414531	1.69E-18	postive
NSUN7	AC139887.	0.382689	8.77E-16	postive
DNMT3A	AC139887.	0.322271	2.19E-11	postive
RAD52	AC139887.	0.374314	4.07E-15	postive
MECP2	AC139887.	0.313192	8.35E-11	postive
NEIL1	AC139887.	0.373348	4.85E-15	postive
ZBTB33	AC139887.	0.32136	2.51E-11	postive

TET1	AC139887.	0.527462	8.23E-31	postive
TET2	AC139887.	0.593275	1.96E-40	postive
TET3	AC139887.	0.477358	8.83E-25	postive
NSUN6	LINC01126	0.306975	2.04E-10	postive
NSUN7	LINC01126	0.361734	3.76E-14	postive
DNMT3A	LINC01126	0.347943	3.86E-13	postive
RAD52	LINC01126	0.307732	1.83E-10	postive
MECP2	LINC01126	0.33729	2.16E-12	postive
ZBTB33	LINC01126	0.325486	1.35E-11	postive
TET1	LINC01126	0.582039	1.23E-38	postive
TET2	LINC01126	0.332426	4.63E-12	postive
TET3	LINC01126	0.421615	3.83E-19	postive
NSUN6	LINC02416	0.308558	1.63E-10	postive
NEIL1	LINC02416	0.323883	1.72E-11	postive
NSUN3	LINC00641	0.445831	1.83E-21	postive
NSUN7	LINC00641	0.3179	4.19E-11	postive
TRDMT1	LINC00641	0.423234	2.72E-19	postive
MECP2	LINC00641	0.411203	3.36E-18	postive
TDG	LINC00641	0.337521	2.08E-12	postive
UHRF2	LINC00641	0.302423	3.86E-10	postive
TET1	LINC00641	0.562956	9.83E-36	postive
TET2	LINC00641	0.478737	6.21E-25	postive
TET3	LINC00641	0.376562	2.71E-15	postive
NSUN6	AC087289.	0.30619	2.28E-10	postive
RAD52	AC087289.	0.434907	2.15E-20	postive
NEIL1	AC087289.	0.587227	1.86E-39	postive
DNMT3A	SREBF2-AS	0.416729	1.07E-18	postive
MECP2	SREBF2-AS	0.324203	1.64E-11	postive
TDG	SREBF2-AS	0.301226	4.56E-10	postive
TET1	SREBF2-AS	0.318823	3.66E-11	postive
TET3	SREBF2-AS	0.324226	1.63E-11	postive
NSUN7	TAF1A-AS	0.300768	4.86E-10	postive
NSUN6	AL158163.	0.340871	1.22E-12	postive
RAD52	AL158163.	0.310378	1.25E-10	postive
NEIL1	AL158163.	0.388472	2.96E-16	postive
TET1	AL158163.	0.444525	2.46E-21	postive
TET2	AL158163.	0.459498	7.35E-23	postive
NEIL1	AC012435.	0.308462	1.65E-10	postive
TET1	AC012435.	0.384982	5.72E-16	postive
TET2	AC012435.	0.559233	3.44E-35	postive
TET3	AC012435.	0.300092	5.33E-10	postive
NSUN6	AC020558.	0.458191	1.01E-22	postive
RAD52	AC020558.	0.409162	5.09E-18	postive
MBD1	AC020558.	0.393277	1.18E-16	postive
NEIL1	AC020558.	0.514863	3.36E-29	postive
NSUN7	CEP83-DT	0.309956	1.33E-10	postive
DNMT1	CEP83-DT	0.352693	1.75E-13	postive
DNMT3A	CEP83-DT	0.41809	8.06E-19	postive
UNG	CEP83-DT	0.345768	5.51E-13	postive
NSUN6	LINC00663	0.4389	8.81E-21	postive
NSUN7	LINC00663	0.395681	7.42E-17	postive
NEIL1	LINC00663	0.389725	2.34E-16	postive
NSUN5	AC008610.	0.342062	1.01E-12	postive
MBD3	AC008610.	0.392864	1.28E-16	postive
NEIL1	AC008610.	0.48101	3.46E-25	postive
NTHL1	AC008610.	0.402678	1.88E-17	postive
DNMT3A	AC002550.	0.375331	3.39E-15	postive
RAD52	AC002550.	0.310713	1.19E-10	postive

TET1	AC002550.	0.30835	1.68E-10	postive
NSUN7	TDRKH-AS	0.302295	3.93E-10	postive
MBD4	TDRKH-AS	0.368219	1.21E-14	postive
NSUN7	AC009506.	0.301178	4.59E-10	postive
DNMT3A	AC009506.	0.329971	6.78E-12	postive
NSUN6	AL139287.	0.315658	5.83E-11	postive
NSUN7	AL139287.	0.339004	1.64E-12	postive
RAD52	AL139287.	0.330726	6.03E-12	postive
NEIL1	AL139287.	0.413121	2.26E-18	postive
TET3	AL139287.	0.332076	4.89E-12	postive
TET1	AC051619.	0.321165	2.58E-11	postive
NEIL1	AC009283.	0.361135	4.17E-14	postive
DNMT3A	BX649601.	0.37885	1.78E-15	postive
NSUN3	AC019080.	0.46244	3.61E-23	postive
TRDMT1	AC019080.	0.349351	3.06E-13	postive
UHRF2	AC019080.	0.350589	2.49E-13	postive
TET1	AC019080.	0.498759	3.10E-27	postive
TET2	AC019080.	0.388261	3.08E-16	postive
RAD52	AC021851.	0.391257	1.74E-16	postive
NEIL1	AC021851.	0.317269	4.60E-11	postive
NSUN6	MAP3K14-	0.412691	2.47E-18	postive
NSUN7	MAP3K14-	0.333648	3.83E-12	postive
DNMT3A	MAP3K14-	0.452836	3.58E-22	postive
RAD52	MAP3K14-	0.316982	4.80E-11	postive
MBD1	MAP3K14-	0.371796	6.40E-15	postive
ZBTB33	MAP3K14-	0.373834	4.44E-15	postive
TET2	MAP3K14-	0.347003	4.50E-13	postive
TET3	MAP3K14-	0.431505	4.55E-20	postive
NSUN6	AC009318.	0.34315	8.43E-13	postive
NSUN7	AC009318.	0.315496	5.97E-11	postive
TET1	AC009318.	0.603162	4.45E-42	postive
TET2	AC009318.	0.321202	2.57E-11	postive
TET3	AC009318.	0.328945	7.94E-12	postive
NEIL1	AC092143.	0.357259	8.11E-14	postive
TET2	AC092143.	0.345315	5.93E-13	postive
NSUN3	AC006160.	0.639307	1.32E-48	postive
TRDMT1	AC006160.	0.456001	1.70E-22	postive
UHRF2	AC006160.	0.372603	5.54E-15	postive
ZBTB38	AC006160.	0.313378	8.13E-11	postive
TET1	AC006160.	0.544411	4.34E-33	postive
TET2	AC006160.	0.444786	2.32E-21	postive
DNMT3B	AC131009.	0.313885	7.55E-11	postive
TET1	AC131009.	0.390699	1.94E-16	postive
NSUN3	ANKRD44-	0.737993	7.27E-72	postive
TRDMT1	ANKRD44-	0.46947	6.43E-24	postive
TET1	ANKRD44-	0.417214	9.68E-19	postive
TET2	ANKRD44-	0.422708	3.04E-19	postive
TET1	TTC3-AS1	0.533469	1.33E-31	postive
TET2	TTC3-AS1	0.569671	9.84E-37	postive
TET3	TTC3-AS1	0.337847	1.97E-12	postive
SMUG1	LINC0229E	0.307593	1.87E-10	postive
NSUN6	NDUFA6-[	0.367662	1.34E-14	postive
NSUN7	NDUFA6-[	0.391412	1.69E-16	postive
DNMT3A	NDUFA6-[	0.348348	3.61E-13	postive
RAD52	NDUFA6-[	0.381908	1.01E-15	postive
MECP2	NDUFA6-[	0.362983	3.03E-14	postive
ZBTB33	NDUFA6-[	0.341032	1.19E-12	postive
TET1	NDUFA6-[	0.388313	3.05E-16	postive

TET2	NDUFA6-[	0.401691	2.28E-17	postive
TET3	NDUFA6-[	0.366777	1.56E-14	postive
NSUN3	AC130324.	0.309381	1.45E-10	postive
TET1	AC130324.	0.363303	2.87E-14	postive
DNMT3A	GHET1	0.415834	1.29E-18	postive
ZBTB33	GHET1	0.342828	8.89E-13	postive
TET3	GHET1	0.41674	1.07E-18	postive
NSUN6	AL590652.	0.551657	4.20E-34	postive
NSUN7	AL590652.	0.433227	3.11E-20	postive
RAD52	AL590652.	0.454434	2.46E-22	postive
MBD1	AL590652.	0.346356	5.00E-13	postive
NEIL1	AL590652.	0.489255	4.01E-26	postive
ZBTB33	AL590652.	0.309689	1.38E-10	postive
TET2	AL590652.	0.466877	1.22E-23	postive
TET3	AL590652.	0.368811	1.09E-14	postive
MBD3	AC008915.	0.342405	9.51E-13	postive
SMUG1	AC008915.	0.30506	2.67E-10	postive
NSUN3	AC015849.	0.467772	9.79E-24	postive
UHRF2	AC015849.	0.300973	4.72E-10	postive
ZBTB38	AC015849.	0.306327	2.23E-10	postive
TET1	AC015849.	0.555141	1.34E-34	postive
TET2	AC015849.	0.605898	1.52E-42	postive
TET3	AC015849.	0.37461	3.86E-15	postive
NSUN6	DICER1-AS	0.306265	2.25E-10	postive
NSUN7	DICER1-AS	0.382577	8.96E-16	postive
NEIL1	DICER1-AS	0.380578	1.30E-15	postive
NTHL1	DICER1-AS	0.360343	4.78E-14	postive
NSUN3	AC009095.	0.488028	5.55E-26	postive
NSUN6	AC009095.	0.315068	6.36E-11	postive
NSUN7	AC009095.	0.315487	5.98E-11	postive
RAD52	AC009095.	0.346435	4.94E-13	postive
UHRF2	AC009095.	0.313815	7.63E-11	postive
ZBTB33	AC009095.	0.332942	4.27E-12	postive
TET1	AC009095.	0.605993	1.47E-42	postive
TET2	AC009095.	0.659593	1.15E-52	postive
TET3	AC009095.	0.453531	3.04E-22	postive
NSUN3	AL117329.	0.319536	3.29E-11	postive
ZBTB33	AL117329.	0.324555	1.55E-11	postive
TET1	AL117329.	0.676436	2.74E-56	postive
TET2	AL117329.	0.568714	1.37E-36	postive
TET3	AL117329.	0.427094	1.19E-19	postive
NSUN6	AC021016.	0.313537	7.94E-11	postive
MBD1	AC021016.	0.30986	1.35E-10	postive
NEIL1	AC021016.	0.328989	7.88E-12	postive
NSUN3	AC078846.	0.33576	2.75E-12	postive
NSUN6	AC078846.	0.344114	7.21E-13	postive
NSUN7	AC078846.	0.354834	1.22E-13	postive
DNMT3A	AC078846.	0.326862	1.09E-11	postive
RAD52	AC078846.	0.302673	3.73E-10	postive
NEIL1	AC078846.	0.310914	1.16E-10	postive
TET1	AC078846.	0.606011	1.46E-42	postive
TET2	AC078846.	0.528997	5.18E-31	postive
TET3	AC078846.	0.392074	1.49E-16	postive
TET1	RNF216P1	0.37102	7.36E-15	postive
NTHL1	BTF3-DT	0.394939	8.57E-17	postive
DNMT3B	AL161772.	0.482652	2.26E-25	postive
TET3	AL161772.	0.367055	1.49E-14	postive
NSUN3	AL158166.	0.394689	9.00E-17	postive

UHRF2	AL158166.	0.37602	2.99E-15	postive
ZBTB38	AL158166.	0.322848	2.01E-11	postive
TET1	AL158166.	0.346629	4.79E-13	postive
TET2	AL158166.	0.476199	1.19E-24	postive
NEIL1	U47924.3	0.314946	6.47E-11	postive
NSUN3	AC004846.	0.521774	4.48E-30	postive
TRDMT1	AC004846.	0.309902	1.34E-10	postive
TET1	AC004846.	0.310877	1.17E-10	postive
NSUN5	AC026979.	0.351468	2.15E-13	postive
MBD3	AC026979.	0.408261	6.11E-18	postive
NTHL1	AC026979.	0.578891	3.83E-38	postive
SMUG1	AC026979.	0.373044	5.12E-15	postive
ZBTB38	AC026979.	-0.3239	1.71E-11	negative
ZBTB33	AL590226.	-0.32594	1.26E-11	negative
NSUN6	AL121832.	0.351868	2.01E-13	postive
NSUN7	AL121832.	0.457905	1.08E-22	postive
DNMT3A	AL121832.	0.324357	1.60E-11	postive
RAD52	AL121832.	0.361393	3.99E-14	postive
MBD1	AL121832.	0.375358	3.37E-15	postive
NEIL1	AL121832.	0.429553	6.96E-20	postive
TET1	AL121832.	0.335286	2.96E-12	postive
TET2	AL121832.	0.319607	3.26E-11	postive
TET3	AL121832.	0.425117	1.82E-19	postive
NEIL1	AP003059.	0.303314	3.41E-10	postive
TET1	LINC0234(	0.316981	4.80E-11	postive
TET3	LINC0234(	0.352871	1.70E-13	postive
NSUN6	NDUFB2-/	0.43702	1.34E-20	postive
NSUN7	NDUFB2-/	0.364073	2.51E-14	postive
TET1	NDUFB2-/	0.346377	4.99E-13	postive
TET3	NDUFB2-/	0.374709	3.79E-15	postive
NSUN3	AL450998.	0.571045	6.11E-37	postive
TRDMT1	AL450998.	0.364585	2.29E-14	postive
MECP2	AL450998.	0.322493	2.12E-11	postive
UHRF2	AL450998.	0.362885	3.08E-14	postive
ZBTB33	AL450998.	0.304869	2.74E-10	postive
ZBTB38	AL450998.	0.31853	3.82E-11	postive
TET1	AL450998.	0.545734	2.85E-33	postive
TET2	AL450998.	0.648474	2.11E-50	postive
TET3	AL450998.	0.384267	6.54E-16	postive
NSUN6	AL161729.	0.387176	3.79E-16	postive
RAD52	AL161729.	0.300139	5.30E-10	postive
NEIL1	AL161729.	0.356962	8.53E-14	postive
TET1	AL161729.	0.35068	2.45E-13	postive
NSUN3	AC009041.	0.428139	9.46E-20	postive
TRDMT1	AC009041.	0.390852	1.88E-16	postive
TET1	AC009041.	0.505855	4.35E-28	postive
TET2	AC009041.	0.394599	9.16E-17	postive
NSUN5	AP003392.	0.32802	9.15E-12	postive
MBD3	AP003392.	0.393039	1.24E-16	postive
NTHL1	AP003392.	0.355462	1.10E-13	postive
TET2	AP003392.	-0.30288	3.62E-10	negative
TET2	AC087392.	0.334038	3.60E-12	postive
NSUN6	AL451050.	0.351439	2.16E-13	postive
NEIL1	AL451050.	0.366777	1.56E-14	postive
NSUN3	FKBP14-A!	0.428337	9.07E-20	postive
TRDMT1	FKBP14-A!	0.336695	2.37E-12	postive
UHRF2	FKBP14-A!	0.359854	5.20E-14	postive
ZBTB38	FKBP14-A!	0.311986	9.94E-11	postive

TET1	FKBP14-A!	0.460342	6.00E-23	postive
TET2	FKBP14-A!	0.520469	6.58E-30	postive
NEIL1	AC005332.	0.461485	4.55E-23	postive
NSUN3	AP001469.	0.425974	1.51E-19	postive
TRDMT1	AP001469.	0.301608	4.32E-10	postive
UHRF2	AP001469.	0.337787	1.99E-12	postive
ZBTB33	AP001469.	0.346715	4.72E-13	postive
ZBTB38	AP001469.	0.331045	5.74E-12	postive
TET1	AP001469.	0.546748	2.06E-33	postive
TET2	AP001469.	0.635868	6.02E-48	postive
TET3	AP001469.	0.445536	1.95E-21	postive
TET1	AL162734.	0.509935	1.38E-28	postive
TET2	AL162734.	0.526608	1.06E-30	postive
TET3	AL162734.	0.309466	1.43E-10	postive
NSUN6	AL928654.	0.373236	4.95E-15	postive
NSUN7	AL928654.	0.376114	2.94E-15	postive
RAD52	AL928654.	0.424819	1.94E-19	postive
MBD1	AL928654.	0.391699	1.60E-16	postive
MECP2	AL928654.	0.328908	7.98E-12	postive
NEIL1	AL928654.	0.535467	7.17E-32	postive
TET2	AL928654.	0.386126	4.61E-16	postive
NSUN5	AC015917.	0.323584	1.80E-11	postive
MBD3	AC015917.	0.384932	5.77E-16	postive
NTHL1	AC015917.	0.495643	7.24E-27	postive
NSUN3	Z94721.1	0.382492	9.10E-16	postive
NSUN7	Z94721.1	0.326727	1.11E-11	postive
DNMT3A	Z94721.1	0.33751	2.08E-12	postive
RAD52	Z94721.1	0.332456	4.61E-12	postive
MECP2	Z94721.1	0.31049	1.23E-10	postive
TET1	Z94721.1	0.535471	7.16E-32	postive
TET2	Z94721.1	0.389258	2.55E-16	postive
TET3	Z94721.1	0.420339	5.02E-19	postive
NEIL1	SZT2-AS1	0.30078	4.85E-10	postive
NTHL1	CH17-340	0.320039	3.05E-11	postive
SMUG1	CH17-340	0.312249	9.57E-11	postive
NSUN3	AL139353.	0.341025	1.19E-12	postive
NSUN7	AL139353.	0.344069	7.27E-13	postive
UHRF2	AL139353.	0.308461	1.65E-10	postive
ZBTB33	AL139353.	0.331649	5.23E-12	postive
TET1	AL139353.	0.575695	1.19E-37	postive
TET2	AL139353.	0.583443	7.41E-39	postive
TET3	AL139353.	0.378813	1.80E-15	postive
NEIL1	MIR762HG	0.319072	3.52E-11	postive
NSUN6	AC021016.	0.413465	2.11E-18	postive
DNMT1	AC021016.	-0.36418	2.46E-14	negative
ALYREF	AC021016.	-0.30284	3.64E-10	negative
UHRF1	AC021016.	-0.31847	3.85E-11	negative
NSUN3	AC104984.	0.586056	2.85E-39	postive
TRDMT1	AC104984.	0.371222	7.10E-15	postive
UHRF2	AC104984.	0.359396	5.63E-14	postive
ZBTB38	AC104984.	0.306053	2.32E-10	postive
TET1	AC104984.	0.600027	1.50E-41	postive
TET2	AC104984.	0.60143	8.71E-42	postive
TET3	AC104984.	0.308028	1.75E-10	postive
DNMT3A	CTB-178M	0.340707	1.25E-12	postive
TET1	AC007014.	0.34159	1.09E-12	postive
NSUN3	AL158212.	0.333147	4.14E-12	postive
DNMT3A	AL158212.	0.345285	5.96E-13	postive

TRDMT1	AL158212.	0.317045	4.76E-11	postive
RAD52	AL158212.	0.342024	1.01E-12	postive
MECP2	AL158212.	0.468714	7.75E-24	postive
UHRF2	AL158212.	0.371539	6.71E-15	postive
ZBTB33	AL158212.	0.342129	9.95E-13	postive
TET1	AL158212.	0.501063	1.65E-27	postive
TET2	AL158212.	0.443427	3.16E-21	postive
TET3	AL158212.	0.385742	4.96E-16	postive
NSUN6	AC084781.	0.404355	1.34E-17	postive
TET1	AC084781.	0.468934	7.34E-24	postive
DNMT3A	AL354892.	0.426444	1.37E-19	postive
NSUN3	AP001432.	0.438213	1.03E-20	postive
ZBTB33	AP001432.	0.334329	3.44E-12	postive
ZBTB38	AP001432.	0.331995	4.95E-12	postive
TET1	AP001432.	0.563257	8.88E-36	postive
TET2	AP001432.	0.612124	1.28E-43	postive
TET3	AP001432.	0.425154	1.80E-19	postive
NSUN6	AL138756.	0.394828	8.76E-17	postive
NSUN7	AL138756.	0.409476	4.77E-18	postive
TET1	AL138756.	0.513247	5.36E-29	postive
TET3	AL138756.	0.364235	2.44E-14	postive
NSUN7	AC079145.	0.309045	1.52E-10	postive
DNMT3A	AC079145.	0.347647	4.05E-13	postive
NSUN6	AC006435.	0.526075	1.25E-30	postive
NSUN7	AC006435.	0.387821	3.35E-16	postive
RAD52	AC006435.	0.481848	2.79E-25	postive
MBD1	AC006435.	0.403715	1.53E-17	postive
NEIL1	AC006435.	0.510561	1.15E-28	postive
TET2	AC006435.	0.31863	3.76E-11	postive
TET3	AC006435.	0.335351	2.93E-12	postive
NSUN6	AC008781.	0.326732	1.11E-11	postive
TET1	AC008781.	0.633139	1.98E-47	postive
TET2	AC008781.	0.336276	2.53E-12	postive
NSUN7	AL162377.	0.305515	2.50E-10	postive
SMUG1	AL132712.	0.320777	2.74E-11	postive
SMUG1	AC023043.	0.306111	2.30E-10	postive
NSUN6	AL033384.	0.419893	5.52E-19	postive
RAD52	AL033384.	0.465292	1.80E-23	postive
NEIL1	AL033384.	0.516161	2.31E-29	postive
NSUN7	AC091271.	0.332941	4.27E-12	postive
DNMT3A	AC091271.	0.320014	3.06E-11	postive
NSUN3	AL021368.	0.327975	9.21E-12	postive
NSUN6	AL021368.	0.391823	1.56E-16	postive
NSUN7	AL021368.	0.439856	7.11E-21	postive
RAD52	AL021368.	0.363339	2.85E-14	postive
MECP2	AL021368.	0.3835	7.54E-16	postive
NEIL1	AL021368.	0.363684	2.68E-14	postive
UHRF2	AL021368.	0.348307	3.63E-13	postive
ZBTB33	AL021368.	0.316477	5.17E-11	postive
TET1	AL021368.	0.524128	2.23E-30	postive
TET2	AL021368.	0.552825	2.87E-34	postive
TET3	AL021368.	0.34574	5.53E-13	postive
NSUN3	AL513327.	0.444729	2.35E-21	postive
NSUN6	AL513327.	0.329272	7.55E-12	postive
NSUN7	AL513327.	0.382887	8.46E-16	postive
DNMT3A	AL513327.	0.316963	4.81E-11	postive
RAD52	AL513327.	0.378715	1.83E-15	postive
MECP2	AL513327.	0.301907	4.15E-10	postive

UHRF2	AL513327.	0.304342	2.95E-10	postive
ZBTB33	AL513327.	0.343904	7.46E-13	postive
TET1	AL513327.	0.607104	9.47E-43	postive
TET2	AL513327.	0.595749	7.68E-41	postive
TET3	AL513327.	0.455809	1.77E-22	postive
NSUN6	AC245884.	0.381575	1.08E-15	postive
DNMT3A	AC245884.	0.312845	8.78E-11	postive
RAD52	AC245884.	0.309946	1.33E-10	postive
NEIL1	AC245884.	0.380541	1.31E-15	postive
ZBTB33	AC245884.	0.329591	7.18E-12	postive
TET2	AC245884.	0.339236	1.58E-12	postive
TET3	AC245884.	0.435377	1.94E-20	postive
NSUN3	AC073130.	0.42418	2.22E-19	postive
TRDMT1	AC073130.	0.327175	1.04E-11	postive
UHRF2	AC073130.	0.360265	4.85E-14	postive
ZBTB38	AC073130.	0.305704	2.44E-10	postive
TET1	AC073130.	0.626242	3.80E-46	postive
TET2	AC073130.	0.567922	1.80E-36	postive
TET3	AC073130.	0.345749	5.53E-13	postive
NSUN6	AL160314.	0.381518	1.09E-15	postive
NSUN7	AL160314.	0.445674	1.89E-21	postive
TET1	AL160314.	0.536634	5.00E-32	postive
NSUN7	AC128687.	0.352254	1.89E-13	postive
DNMT3A	AC128687.	0.32326	1.89E-11	postive
NSUN5	AC021028.	0.310823	1.18E-10	postive
TET1	AC009754.	0.589316	8.58E-40	postive
TET2	AC009754.	0.561398	1.66E-35	postive
TET3	AC009754.	0.348107	3.75E-13	postive
NSUN3	AL117336.	0.391254	1.74E-16	postive
TRDMT1	AL117336.	0.363746	2.65E-14	postive
UHRF2	AL117336.	0.305326	2.57E-10	postive
TET1	AL117336.	0.384906	5.80E-16	postive
NTHL1	AC009097.	0.304521	2.88E-10	postive
SMUG1	AC009097.	0.30546	2.52E-10	postive
NSUN6	AL583810.	0.41363	2.04E-18	postive
RAD52	AL583810.	0.365751	1.87E-14	postive
NEIL1	AL583810.	0.506553	3.58E-28	postive
NSUN3	AL354733.	0.318733	3.71E-11	postive
NSUN6	AL354733.	0.312823	8.81E-11	postive
DNMT3A	AL354733.	0.313705	7.75E-11	postive
RAD52	AL354733.	0.408145	6.26E-18	postive
MBD1	AL354733.	0.328446	8.57E-12	postive
MECP2	AL354733.	0.330648	6.10E-12	postive
UHRF2	AL354733.	0.300293	5.19E-10	postive
ZBTB33	AL354733.	0.392042	1.50E-16	postive
TET1	AL354733.	0.302726	3.70E-10	postive
TET2	AL354733.	0.472376	3.11E-24	postive
TET3	AL354733.	0.416029	1.24E-18	postive
NSUN3	AC010525.	0.47602	1.24E-24	postive
TET1	AC010525.	0.379305	1.64E-15	postive
TET2	AC010525.	0.391539	1.65E-16	postive
NSUN7	AC084782.	0.393204	1.20E-16	postive
NEIL1	AC084782.	0.491773	2.05E-26	postive
NSUN3	AP001528.	0.38931	2.53E-16	postive
TET1	AP001528.	0.409016	5.24E-18	postive
NSUN3	AC091057.	0.394496	9.34E-17	postive
DNMT1	AC091057.	0.469785	5.94E-24	postive
DNMT3A	AC091057.	0.374448	3.97E-15	postive

TRDMT1	AC091057.	0.307594	1.87E-10	postive
MBD1	AC091057.	0.345325	5.92E-13	postive
MBD4	AC091057.	0.352588	1.78E-13	postive
MECP2	AC091057.	0.324703	1.52E-11	postive
TDG	AC091057.	0.372662	5.48E-15	postive
UHRF1	AC091057.	0.351151	2.27E-13	postive
UHRF2	AC091057.	0.302329	3.91E-10	postive
ZBTB33	AC091057.	0.354723	1.25E-13	postive
TET1	AC091057.	0.418681	7.12E-19	postive
TET2	AC091057.	0.403638	1.55E-17	postive
TET3	AC091057.	0.463813	2.59E-23	postive
NSUN6	SNHG20	0.34353	7.93E-13	postive
NSUN7	SNHG20	0.306727	2.11E-10	postive
DNMT3A	SNHG20	0.411132	3.40E-18	postive
RAD52	SNHG20	0.365049	2.11E-14	postive
NEIL1	SNHG20	0.529798	4.06E-31	postive
DNMT3A	AL391152.	0.39493	8.59E-17	postive
TET1	AL391152.	0.352976	1.67E-13	postive
NSUN3	AC016405.	0.310808	1.18E-10	postive
TET1	AC016405.	0.66295	2.27E-53	postive
TET2	AC016405.	0.532718	1.67E-31	postive
TET3	AC016405.	0.355638	1.07E-13	postive
NSUN6	AL157838.	0.309047	1.52E-10	postive
NSUN7	AL157838.	0.32211	2.24E-11	postive
ZBTB33	AL157838.	0.325261	1.39E-11	postive
TET1	AL157838.	0.57704	7.41E-38	postive
TET2	AL157838.	0.635983	5.73E-48	postive
TET3	AL157838.	0.423329	2.66E-19	postive
TET1	XXYLT1-AS	0.301457	4.42E-10	postive
TET2	XXYLT1-AS	0.326166	1.21E-11	postive
NSUN5	DNAJC9-A	0.39004	2.20E-16	postive
ALYREF	DNAJC9-A	0.316562	5.11E-11	postive
NTHL1	DNAJC9-A	0.303632	3.26E-10	postive
NSUN6	AC021321.	0.460848	5.31E-23	postive
NSUN7	AC021321.	0.425369	1.72E-19	postive
DNMT3A	AC021321.	0.375413	3.34E-15	postive
RAD52	AC021321.	0.375025	3.58E-15	postive
SMUG1	AC021321.	0.302574	3.78E-10	postive
TET1	AC021321.	0.495092	8.40E-27	postive
TET3	AC021321.	0.341936	1.03E-12	postive
NSUN3	AC005332.	0.327772	9.50E-12	postive
NSUN6	AC005332.	0.310508	1.23E-10	postive
RAD52	AC005332.	0.350672	2.45E-13	postive
NEIL1	AC005332.	0.37944	1.60E-15	postive
RAD52	AL139423.	0.346316	5.04E-13	postive
NEIL1	AL139423.	0.355757	1.05E-13	postive
TET2	AL139423.	0.418457	7.46E-19	postive
TET3	AL139423.	0.379921	1.46E-15	postive
NSUN6	AC024361.	0.421881	3.62E-19	postive
NSUN7	AC024361.	0.450938	5.59E-22	postive
DNMT3A	AC024361.	0.321606	2.42E-11	postive
RAD52	AC024361.	0.37994	1.46E-15	postive
NEIL1	AC024361.	0.354799	1.23E-13	postive
TET1	AC024361.	0.54443	4.31E-33	postive
TET2	AC024361.	0.329803	6.95E-12	postive
TET3	AC024361.	0.395453	7.76E-17	postive
NSUN5	SNHG5	0.321564	2.43E-11	postive
MBD3	SNHG5	0.334196	3.51E-12	postive

NTHL1	SNHG5	0.460521	5.75E-23	postive
NSUN7	AC022079.	0.333719	3.78E-12	postive
TET1	AC022079.	0.432335	3.79E-20	postive
NSUN6	STX18-AS1	0.336739	2.35E-12	postive
NSUN7	STX18-AS1	0.474299	1.92E-24	postive
DNMT3A	STX18-AS1	0.357178	8.22E-14	postive
RAD52	STX18-AS1	0.427252	1.15E-19	postive
MECP2	STX18-AS1	0.352986	1.67E-13	postive
NEIL1	STX18-AS1	0.367121	1.47E-14	postive
ZBTB33	STX18-AS1	0.421791	3.69E-19	postive
TET1	STX18-AS1	0.438227	1.02E-20	postive
TET2	STX18-AS1	0.524382	2.07E-30	postive
TET3	STX18-AS1	0.375709	3.16E-15	postive
NSUN7	AC103746.	0.314851	6.56E-11	postive
TET1	AC103746.	0.704312	7.66E-63	postive
TET3	AC103746.	0.322	2.28E-11	postive
DNMT3A	LINC01018	0.331699	5.19E-12	postive
NSUN3	RC3H1-IT1	0.366946	1.52E-14	postive
UHRF2	RC3H1-IT1	0.324265	1.62E-11	postive
TET1	RC3H1-IT1	0.517136	1.74E-29	postive
TET2	RC3H1-IT1	0.590791	4.96E-40	postive
TET3	RC3H1-IT1	0.307743	1.83E-10	postive
YBX1	AC100793.	0.380792	1.25E-15	postive
NSUN7	LINC00865	0.325657	1.31E-11	postive
NEIL1	LINC00865	0.377957	2.10E-15	postive
NSUN3	AC104534.	0.329895	6.86E-12	postive
NSUN7	AC104534.	0.309644	1.39E-10	postive
TET1	AC104534.	0.467966	9.33E-24	postive
TET2	AC104534.	0.440441	6.23E-21	postive
TET3	AC104534.	0.382543	9.02E-16	postive
NEIL1	AC010359.	0.318008	4.13E-11	postive
SMUG1	AC010359.	0.313334	8.18E-11	postive
NSUN3	AC012568.	0.547238	1.76E-33	postive
TRDMT1	AC012568.	0.338642	1.74E-12	postive
UHRF2	AC012568.	0.353016	1.66E-13	postive
TET1	AC012568.	0.495074	8.44E-27	postive
TET2	AC012568.	0.580488	2.16E-38	postive
NSUN3	AC019183.	0.59411	1.43E-40	postive
TRDMT1	AC019183.	0.440987	5.51E-21	postive
TET1	AC019183.	0.690762	1.46E-59	postive
TET2	AC019183.	0.390088	2.18E-16	postive
TET3	AC019183.	0.3192	3.46E-11	postive
NSUN6	TMED2-DT	0.368457	1.16E-14	postive
NSUN7	TMED2-DT	0.406555	8.63E-18	postive
DNMT3A	TMED2-DT	0.362721	3.17E-14	postive
RAD52	TMED2-DT	0.492824	1.55E-26	postive
NEIL1	TMED2-DT	0.387	3.91E-16	postive
SMUG1	TMED2-DT	0.309682	1.38E-10	postive
TET1	TMED2-DT	0.333662	3.82E-12	postive
ZBTB38	AC136475.	0.34368	7.74E-13	postive
NSUN6	STAM-AS1	0.460228	6.17E-23	postive
NSUN7	STAM-AS1	0.378424	1.93E-15	postive
DNMT3A	STAM-AS1	0.416028	1.24E-18	postive
RAD52	STAM-AS1	0.409882	4.39E-18	postive
MBD1	STAM-AS1	0.33736	2.13E-12	postive
MECP2	STAM-AS1	0.322361	2.16E-11	postive
NEIL1	STAM-AS1	0.319744	3.19E-11	postive
ZBTB33	STAM-AS1	0.326979	1.07E-11	postive

TET1	STAM-AS1	0.417854	8.47E-19	postive
TET3	STAM-AS1	0.348212	3.69E-13	postive
NTHL1	HOXC-AS:	0.39638	6.48E-17	postive
NSUN3	AC104170.	0.597486	3.96E-41	postive
TRDMT1	AC104170.	0.33068	6.07E-12	postive
UHRF2	AC104170.	0.326685	1.12E-11	postive
ZBTB38	AC104170.	0.304117	3.05E-10	postive
TET1	AC104170.	0.558538	4.34E-35	postive
TET2	AC104170.	0.57409	2.10E-37	postive
ZBTB33	AL592043.	0.301802	4.21E-10	postive
TET1	AL592043.	0.459237	7.83E-23	postive
TET2	AL592043.	0.318331	3.93E-11	postive
TET3	AL592043.	0.351262	2.23E-13	postive
NSUN6	AC096642.	0.311137	1.12E-10	postive
RAD52	AC096642.	0.460965	5.16E-23	postive
NEIL1	AC096642.	0.429703	6.74E-20	postive
TET1	AC096642.	0.301404	4.45E-10	postive
TET2	AC096642.	0.35897	6.05E-14	postive
NSUN3	DHDDS-A:	0.523091	3.03E-30	postive
NSUN7	DHDDS-A:	0.309483	1.42E-10	postive
TRDMT1	DHDDS-A:	0.342515	9.35E-13	postive
RAD52	DHDDS-A:	0.300573	4.99E-10	postive
MECP2	DHDDS-A:	0.322872	2.00E-11	postive
UHRF2	DHDDS-A:	0.345911	5.38E-13	postive
TET1	DHDDS-A:	0.592505	2.61E-40	postive
TET2	DHDDS-A:	0.595482	8.50E-41	postive
TET3	DHDDS-A:	0.369056	1.04E-14	postive
TET1	AC063919.	0.478512	6.58E-25	postive
NSUN3	AC090559.	0.524516	1.99E-30	postive
TRDMT1	AC090559.	0.312654	9.03E-11	postive
ZBTB38	AC090559.	0.421865	3.63E-19	postive
NSUN3	AP000766.	0.309733	1.37E-10	postive
NSUN6	AP000766.	0.32577	1.29E-11	postive
NSUN7	AP000766.	0.391732	1.59E-16	postive
ZBTB33	AP000766.	0.305501	2.51E-10	postive
TET1	AP000766.	0.593833	1.58E-40	postive
TET2	AP000766.	0.480663	3.79E-25	postive
TET3	AP000766.	0.394919	8.61E-17	postive
NSUN3	AC078778.	0.523785	2.47E-30	postive
NSUN7	AC078778.	0.302206	3.98E-10	postive
TRDMT1	AC078778.	0.34909	3.19E-13	postive
TET1	AC078778.	0.68146	2.05E-57	postive
TET2	AC078778.	0.508456	2.09E-28	postive
TET3	AC078778.	0.386241	4.52E-16	postive
NSUN3	AC008966.	0.458895	8.50E-23	postive
TRDMT1	AC008966.	0.347052	4.46E-13	postive
UHRF2	AC008966.	0.357579	7.68E-14	postive
ZBTB38	AC008966.	0.305793	2.41E-10	postive
TET1	AC008966.	0.392084	1.49E-16	postive
TET2	AC008966.	0.480944	3.52E-25	postive
ZBTB33	AC099487.	0.316844	4.90E-11	postive
TET2	AC099487.	0.371166	7.17E-15	postive
TET1	AL135905.	0.364903	2.17E-14	postive
NEIL1	AC020891.	0.436441	1.53E-20	postive
DNMT1	TSPOAP1-	0.321499	2.46E-11	postive
MBD4	TSPOAP1-	0.301927	4.14E-10	postive
NEIL1	AC073655.	0.428819	8.17E-20	postive
NSUN7	AL451085.	0.349981	2.75E-13	postive

MBD3	AL451085.	0.325064	1.44E-11	postive
TET3	AL360181.	0.318312	3.94E-11	postive
NSUN6	AC092140.	0.319011	3.56E-11	postive
TET1	AC092140.	0.35536	1.12E-13	postive
TET2	AC092140.	0.355676	1.06E-13	postive
NSUN6	LINC0261[illegible]	0.414491	1.70E-18	postive
NSUN7	LINC0261[illegible]	0.392068	1.49E-16	postive
RAD52	LINC0261[illegible]	0.378599	1.87E-15	postive
NEIL1	LINC0261[illegible]	0.33241	4.64E-12	postive
TET1	LINC0261[illegible]	0.420967	4.40E-19	postive
TET3	LINC0261[illegible]	0.324851	1.48E-11	postive
NSUN6	AL358472.	0.354711	1.25E-13	postive
NSUN7	AL358472.	0.371	7.38E-15	postive
RAD52	AL358472.	0.365445	1.97E-14	postive
NEIL1	AL358472.	0.495294	7.96E-27	postive
NSUN6	LINC0108[illegible]	0.443899	2.84E-21	postive
RAD52	LINC0108[illegible]	0.367241	1.44E-14	postive
NEIL1	LINC0108[illegible]	0.534411	9.93E-32	postive
NSUN5	AP006621.	0.365877	1.83E-14	postive
MBD3	AP006621.	0.429634	6.84E-20	postive
NTHL1	AP006621.	0.34491	6.34E-13	postive
NSUN6	AL032819.	0.32681	1.10E-11	postive
DNMT3A	AL032819.	0.332246	4.76E-12	postive
TET1	AL032819.	0.428682	8.41E-20	postive
TET3	AL032819.	0.394159	9.97E-17	postive
NSUN6	AC010300.	0.429158	7.59E-20	postive
NSUN7	AC010300.	0.343065	8.55E-13	postive
TET1	AC010300.	0.555494	1.19E-34	postive
TET2	AC010300.	0.317319	4.57E-11	postive
TET3	AC010300.	0.305695	2.44E-10	postive
TET1	AC121247.	0.578454	4.47E-38	postive
SMUG1	AC004801.	0.359948	5.12E-14	postive
TET1	AC024581.	0.363372	2.83E-14	postive
TET2	AC024581.	0.351069	2.30E-13	postive
NSUN7	AC073529.	0.346142	5.18E-13	postive
TDG	AC073529.	0.30018	5.27E-10	postive
UHRF2	AC073529.	0.350063	2.72E-13	postive
ZBTB33	AC073529.	0.458525	9.28E-23	postive
TET1	AC073529.	0.555188	1.32E-34	postive
TET2	AC073529.	0.508639	1.99E-28	postive
TET3	AC073529.	0.433926	2.67E-20	postive
NSUN6	AC010618.	0.41802	8.18E-19	postive
RAD52	AC010618.	0.431619	4.43E-20	postive
NEIL1	AC010618.	0.67062	5.19E-55	postive
NSUN6	AC105339.	0.316867	4.88E-11	postive
NEIL1	AC105339.	0.425707	1.60E-19	postive
UHRF2	AC105339.	0.314787	6.62E-11	postive
ZBTB33	AC105339.	0.371748	6.46E-15	postive
TET1	AC105339.	0.380371	1.35E-15	postive
TET2	AC105339.	0.643607	1.93E-49	postive
TET3	AC105339.	0.36643	1.66E-14	postive
DNMT3A	AL645608.	0.314557	6.85E-11	postive
TET1	AL645608.	0.472068	3.36E-24	postive
TET3	AL645608.	0.392212	1.45E-16	postive
NSUN7	LINC0256[illegible]	0.385634	5.06E-16	postive
ZBTB33	LINC0256[illegible]	0.320544	2.83E-11	postive
TET1	LINC0256[illegible]	0.488902	4.40E-26	postive
TET2	LINC0256[illegible]	0.318476	3.85E-11	postive

TET3	LINC0256(	0.364453	2.35E-14	postive
NSUN3	AP006621.	0.309012	1.52E-10	postive
NSUN6	AP006621.	0.316092	5.47E-11	postive
NSUN7	AP006621.	0.366389	1.67E-14	postive
TRDMT1	AP006621.	0.312035	9.87E-11	postive
MECP2	AP006621.	0.301341	4.49E-10	postive
NEIL1	AP006621.	0.305689	2.44E-10	postive
UHRF2	AP006621.	0.328279	8.79E-12	postive
TET1	AP006621.	0.46178	4.24E-23	postive
TET2	AP006621.	0.345291	5.96E-13	postive
TET3	AP006621.	0.329922	6.83E-12	postive
ALYREF	AC132938.	0.333184	4.11E-12	postive
NSUN6	AC104785.	0.419063	6.57E-19	postive
NSUN7	AC104785.	0.317271	4.60E-11	postive
NEIL1	AC104785.	0.442548	3.87E-21	postive
DNMT3B	AC079907.	0.351723	2.06E-13	postive
RAD52	AC079907.	0.390123	2.16E-16	postive
NEIL1	AC079907.	0.34631	5.04E-13	postive
TET2	AC079907.	0.333754	3.76E-12	postive
NSUN6	MIR29B2C	0.488876	4.43E-26	postive
NSUN7	MIR29B2C	0.353719	1.48E-13	postive
RAD52	MIR29B2C	0.310982	1.15E-10	postive
NEIL1	MIR29B2C	0.342521	9.34E-13	postive
TET1	MIR29B2C	0.432619	3.56E-20	postive
TET2	MIR29B2C	0.494674	9.41E-27	postive
NSUN6	AC120053.	0.349434	3.01E-13	postive
NSUN7	AC120053.	0.319119	3.50E-11	postive
RAD52	AC120053.	0.39349	1.13E-16	postive
NEIL1	AC120053.	0.512886	5.94E-29	postive
NSUN6	HMGA1P4	0.368703	1.11E-14	postive
NSUN3	MSC-AS1	0.375132	3.51E-15	postive
TRDMT1	MSC-AS1	0.306564	2.16E-10	postive
NSUN3	AL022311.	0.419531	5.95E-19	postive
NSUN6	AL022311.	0.42393	2.34E-19	postive
RAD52	AL022311.	0.393334	1.17E-16	postive
NEIL1	AL022311.	0.414969	1.54E-18	postive
TET1	AL022311.	0.539314	2.17E-32	postive
TET2	AL022311.	0.505905	4.29E-28	postive
TET3	AL022311.	0.354726	1.25E-13	postive
TET1	GCC2-AS1	0.365919	1.81E-14	postive
NSUN6	LINC01311	0.305965	2.35E-10	postive
NEIL1	LINC01311	0.322628	2.07E-11	postive
NSUN6	AC008870.	0.319837	3.15E-11	postive
NEIL1	AC008870.	0.421309	4.09E-19	postive
TET2	AC008870.	0.300803	4.83E-10	postive
NSUN6	AC024940.	0.342993	8.65E-13	postive
NSUN7	AC024940.	0.310474	1.24E-10	postive
TET1	AC024940.	0.602712	5.30E-42	postive
TET2	AC024940.	0.367553	1.36E-14	postive
TET3	AC024940.	0.314341	7.07E-11	postive
TET1	AP002336.	0.369142	1.03E-14	postive
NSUN6	MYCL-AS1	0.328918	7.97E-12	postive
NSUN6	AC002347.	0.330569	6.18E-12	postive
TET1	AC002347.	0.566172	3.29E-36	postive
TET1	RPL37A-D	0.484984	1.23E-25	postive
TET1	AC092127.	0.492937	1.50E-26	postive
TET1	AP000223.	0.404329	1.35E-17	postive
TET2	AP000223.	0.309956	1.33E-10	postive

DNMT3A	AC007292.	0.36634	1.69E-14	postive
DNMT3B	AC007292.	0.368008	1.26E-14	postive
RAD52	AC007292.	0.468722	7.74E-24	postive
NEIL1	AC007292.	0.362031	3.57E-14	postive
MBD3	AC022098.	0.302007	4.09E-10	postive
YBX1	AL050341.	0.422316	3.30E-19	postive
RAD52	AC006111.	0.365916	1.82E-14	postive
DNMT3A	AL031186.	0.416894	1.03E-18	postive
DNMT3B	AL031186.	0.332506	4.57E-12	postive
RAD52	AL031186.	0.384357	6.43E-16	postive
NEIL1	AL031186.	0.308402	1.66E-10	postive
NSUN7	UBL7-AS1	0.337414	2.11E-12	postive
MBD1	UBL7-AS1	0.300981	4.72E-10	postive
TDG	UBL7-AS1	0.314928	6.49E-11	postive
ZBTB33	UBL7-AS1	0.416017	1.24E-18	postive
TET1	UBL7-AS1	0.385505	5.19E-16	postive
TET2	UBL7-AS1	0.481867	2.77E-25	postive
TET3	UBL7-AS1	0.480432	4.02E-25	postive
MBD3	AC011444.	0.346631	4.78E-13	postive
NEIL1	AC011444.	0.371375	6.91E-15	postive
NSUN7	AL121574.	0.37291	5.24E-15	postive
DNMT3A	AL121574.	0.30605	2.32E-10	postive
TET1	AL121574.	0.457759	1.12E-22	postive
TET3	AL121574.	0.330846	5.92E-12	postive
NSUN3	C1orf147	0.300997	4.71E-10	postive
TET2	C1orf147	0.349931	2.78E-13	postive
NTHL1	LINC00853	0.340156	1.37E-12	postive
NSUN3	AP005057.	0.422079	3.47E-19	postive
TRDMT1	AP005057.	0.337231	2.18E-12	postive
TET1	AP005057.	0.660525	7.33E-53	postive
TET2	AP005057.	0.301221	4.56E-10	postive
TET3	AP005057.	0.393105	1.22E-16	postive
NSUN6	AC114730.	0.450674	5.94E-22	postive
RAD52	AC114730.	0.429606	6.88E-20	postive
MBD1	AC114730.	0.328142	8.98E-12	postive
NEIL1	AC114730.	0.423654	2.48E-19	postive
NSUN7	PHC2-AS1	0.328185	8.92E-12	postive
TET1	PHC2-AS1	0.554823	1.49E-34	postive
TET2	PHC2-AS1	0.477202	9.19E-25	postive
TET3	PHC2-AS1	0.41471	1.63E-18	postive
NSUN6	MORC2-A	0.319365	3.38E-11	postive
NSUN7	MORC2-A	0.329325	7.49E-12	postive
ZBTB33	MORC2-A	0.304907	2.73E-10	postive
TET1	MORC2-A	0.61913	7.40E-45	postive
TET2	MORC2-A	0.312006	9.92E-11	postive
TET3	MORC2-A	0.388727	2.82E-16	postive
RAD52	AC010168.	0.381568	1.08E-15	postive
NEIL1	AC010168.	0.333225	4.09E-12	postive
TET2	AC010168.	0.333849	3.71E-12	postive
UHRF1	AC027228.	0.3545	1.29E-13	postive
RAD52	AP000864.	0.305261	2.59E-10	postive
NSUN7	LINC02163	0.353142	1.63E-13	postive
DNMT3A	LINC02163	0.372169	5.99E-15	postive
ZBTB33	LINC02163	0.371348	6.94E-15	postive
TET1	LINC02163	0.587668	1.58E-39	postive
TET2	LINC02163	0.450732	5.87E-22	postive
TET3	LINC02163	0.430817	5.28E-20	postive
NSUN6	CYTOR	-0.30286	3.63E-10	negative

TET3	CYTOR	-0.31976	3.18E-11	negative
UHRF2	PTPRK-AS	0.304786	2.77E-10	postive
TET1	PTPRK-AS	0.449774	7.33E-22	postive
TET2	PTPRK-AS	0.573088	2.99E-37	postive
NSUN3	AC127024.	0.442822	3.63E-21	postive
UHRF2	AC127024.	0.333564	3.88E-12	postive
TET1	AC127024.	0.487776	5.93E-26	postive
TET2	AC127024.	0.601075	9.99E-42	postive
TET3	AC127024.	0.315415	6.04E-11	postive
NSUN5	AL121832.	0.345263	5.98E-13	postive
MBD3	AL121832.	0.353019	1.66E-13	postive
NTHL1	AL121832.	0.502176	1.21E-27	postive
SMUG1	AL121832.	0.321932	2.30E-11	postive
NSUN6	AC006557.	0.320468	2.86E-11	postive
NSUN7	AC006557.	0.323483	1.82E-11	postive
DNMT3A	AC006557.	0.391533	1.65E-16	postive
RAD52	AC006557.	0.413612	2.04E-18	postive
MBD1	AC006557.	0.348635	3.44E-13	postive
NEIL1	AC006557.	0.347297	4.29E-13	postive
TET3	AC006557.	0.39949	3.52E-17	postive
NSUN6	AC019131.	0.38509	5.60E-16	postive
NEIL1	AC019131.	0.416267	1.18E-18	postive
SMUG1	AC019131.	0.307415	1.91E-10	postive
NSUN7	STPG3-AS	0.345112	6.13E-13	postive
TET1	AC009226.	0.32015	3.00E-11	postive
NSUN3	EGOT	0.308754	1.58E-10	postive
TRDMT1	EGOT	0.318003	4.13E-11	postive
TET1	EGOT	0.461733	4.29E-23	postive
NSUN3	AL445649.	0.586903	2.09E-39	postive
TRDMT1	AL445649.	0.367293	1.43E-14	postive
TET1	AL445649.	0.303165	3.48E-10	postive
NSUN6	AC020911.	0.34509	6.15E-13	postive
NSUN7	AC020911.	0.357236	8.14E-14	postive
DNMT3A	AC020911.	0.311517	1.06E-10	postive
RAD52	AC020911.	0.369152	1.03E-14	postive
MBD1	AC020911.	0.391345	1.71E-16	postive
NEIL1	AC020911.	0.458616	9.08E-23	postive
ZBTB33	AC020911.	0.377326	2.36E-15	postive
TET1	AC020911.	0.300452	5.08E-10	postive
TET2	AC020911.	0.47483	1.68E-24	postive
TET3	AC020911.	0.407728	6.81E-18	postive
ZBTB33	LINC0064[illegible]	0.344214	7.10E-13	postive
TET2	LINC0064[illegible]	0.528594	5.85E-31	postive
TET3	LINC0064[illegible]	0.39518	8.18E-17	postive
NSUN6	AC010503.	0.386122	4.62E-16	postive
NSUN7	AC010503.	0.384529	6.23E-16	postive
NEIL1	AC010503.	0.500823	1.76E-27	postive
SMUG1	AC010503.	0.35994	5.12E-14	postive
NSUN6	AC022150.	0.327817	9.44E-12	postive
NSUN7	AC022150.	0.354066	1.39E-13	postive
DNMT3A	AC022150.	0.394541	9.26E-17	postive
RAD52	AC022150.	0.33545	2.88E-12	postive
MBD1	AC022150.	0.349302	3.08E-13	postive
NEIL1	AC022150.	0.30758	1.87E-10	postive
ZBTB33	AC022150.	0.347003	4.50E-13	postive
TET3	AC022150.	0.488731	4.60E-26	postive
RAD52	AC010809.	0.366772	1.56E-14	postive
NEIL1	AC010809.	0.393517	1.13E-16	postive

YBX1	LINC02384	0.304405	2.93E-10	postive
NSUN6	LIPC-AS1	0.330074	6.67E-12	postive
NSUN7	LIPC-AS1	0.301382	4.46E-10	postive
TET1	LIPC-AS1	0.635066	8.56E-48	postive
TET2	LIPC-AS1	0.324915	1.47E-11	postive
TET3	LIPC-AS1	0.31393	7.50E-11	postive
NSUN3	MIRLET7A:	0.391401	1.70E-16	postive
NSUN6	MIRLET7A:	0.342213	9.81E-13	postive
NSUN7	MIRLET7A:	0.324461	1.57E-11	postive
TRDMT1	MIRLET7A:	0.307506	1.89E-10	postive
UHRF2	MIRLET7A:	0.301327	4.50E-10	postive
TET1	MIRLET7A:	0.702675	1.95E-62	postive
TET2	MIRLET7A:	0.527977	7.04E-31	postive
TET3	MIRLET7A:	0.372368	5.78E-15	postive
NEIL1	AC007938.	0.343838	7.54E-13	postive
TET2	AC007938.	0.358244	6.85E-14	postive
RAD52	RCCD1-AS	0.369617	9.45E-15	postive
NEIL1	RCCD1-AS	0.542262	8.58E-33	postive
NSUN6	AP001628.	0.375465	3.31E-15	postive
NSUN7	AP001628.	0.334769	3.21E-12	postive
TET1	AP001628.	0.621426	2.86E-45	postive
TET2	AP001628.	0.483138	2.00E-25	postive
TET3	AP001628.	0.382597	8.93E-16	postive
NSUN3	AC005519.	0.355431	1.11E-13	postive
NSUN6	AC005519.	0.412482	2.58E-18	postive
NSUN7	AC005519.	0.398342	4.42E-17	postive
DNMT3A	AC005519.	0.329409	7.39E-12	postive
RAD52	AC005519.	0.427262	1.14E-19	postive
MBD1	AC005519.	0.343376	8.13E-13	postive
MECP2	AC005519.	0.386803	4.06E-16	postive
NEIL1	AC005519.	0.426625	1.31E-19	postive
ZBTB33	AC005519.	0.349606	2.93E-13	postive
TET1	AC005519.	0.452083	4.28E-22	postive
TET2	AC005519.	0.584175	5.68E-39	postive
TET3	AC005519.	0.39276	1.31E-16	postive
NSUN3	TRAF3IP2-	0.439496	7.71E-21	postive
NSUN7	TRAF3IP2-	0.304871	2.74E-10	postive
DNMT3A	TRAF3IP2-	0.306881	2.06E-10	postive
TRDMT1	TRAF3IP2-	0.433047	3.24E-20	postive
MECP2	TRAF3IP2-	0.345903	5.39E-13	postive
UHRF2	TRAF3IP2-	0.35387	1.44E-13	postive
TET1	TRAF3IP2-	0.744941	6.68E-74	postive
TET2	TRAF3IP2-	0.42641	1.38E-19	postive
NSUN6	AC087289.	0.309898	1.34E-10	postive
NSUN7	AC087289.	0.337766	2.00E-12	postive
DNMT3A	AC087289.	0.312443	9.31E-11	postive
RAD52	AC087289.	0.380124	1.41E-15	postive
TET1	AC087289.	0.575653	1.21E-37	postive
TET2	AC087289.	0.473525	2.33E-24	postive
TET3	AC087289.	0.446699	1.50E-21	postive
DNMT3A	AC007541.	0.308418	1.66E-10	postive
NSUN6	AC245052.	0.328183	8.92E-12	postive
NSUN7	AC245052.	0.330015	6.73E-12	postive
DNMT3A	AC245052.	0.421054	4.32E-19	postive
DNMT3B	AC245052.	0.300848	4.80E-10	postive
RAD52	AC245052.	0.50001	2.20E-27	postive
NEIL1	AC245052.	0.392829	1.29E-16	postive
TET3	AC245052.	0.402329	2.01E-17	postive

NSUN6	AC013356.	0.434595	2.30E-20	postive
RAD52	AC013356.	0.375961	3.02E-15	postive
NEIL1	AC013356.	0.443673	2.99E-21	postive
TET1	AC013356.	0.333182	4.12E-12	postive
TET2	AC013356.	0.471118	4.26E-24	postive
TET3	AC013356.	0.42692	1.23E-19	postive
TET1	AP006545.	0.40403	1.43E-17	postive
TET1	AC023034.	0.520467	6.58E-30	postive
TET2	AC023034.	0.532898	1.58E-31	postive
TET3	AC023034.	0.323112	1.93E-11	postive
NSUN6	AC090579.	0.402121	2.10E-17	postive
NSUN7	AC090579.	0.30954	1.41E-10	postive
TET1	AC090579.	0.548031	1.36E-33	postive
TET2	AC090579.	0.408724	5.56E-18	postive
TET3	AC090579.	0.371825	6.37E-15	postive
MECP2	DM1-AS	0.324462	1.57E-11	postive
NSUN3	AL138995.	0.395967	7.02E-17	postive
NSUN6	AC092794.	0.305472	2.52E-10	postive
UHRF2	AC092794.	0.30592	2.36E-10	postive
TET1	AC092794.	0.571283	5.62E-37	postive
TET2	AC092794.	0.56486	5.15E-36	postive
TET3	AC092794.	0.339277	1.57E-12	postive
DNMT3A	AC100812.	0.314133	7.28E-11	postive
MBD3	AC100812.	0.301673	4.28E-10	postive
NEIL1	DLGAP1-A	0.358274	6.82E-14	postive
NSUN6	Z98200.1	0.345062	6.18E-13	postive
RAD52	Z98200.1	0.340283	1.34E-12	postive
TET1	Z98200.1	0.418925	6.76E-19	postive
UHRF1	MIR22HG	-0.3244	1.59E-11	negative
NSUN3	AC138956.	0.440425	6.25E-21	postive
NSUN6	AC138956.	0.373011	5.15E-15	postive
NSUN7	AC138956.	0.351668	2.08E-13	postive
TRDMT1	AC138956.	0.321533	2.44E-11	postive
RAD52	AC138956.	0.348641	3.44E-13	postive
MECP2	AC138956.	0.390761	1.92E-16	postive
NEIL1	AC138956.	0.303864	3.16E-10	postive
UHRF2	AC138956.	0.345423	5.83E-13	postive
ZBTB33	AC138956.	0.389122	2.62E-16	postive
TET1	AC138956.	0.557242	6.69E-35	postive
TET2	AC138956.	0.632689	2.41E-47	postive
TET3	AC138956.	0.445513	1.96E-21	postive
NTHL1	AC131238.	0.343439	8.05E-13	postive
SMUG1	AC131238.	0.359609	5.42E-14	postive
TET1	AC118755.	0.393376	1.16E-16	postive
TET1	AC016550.	0.370579	7.96E-15	postive
TET3	AC016550.	0.30248	3.83E-10	postive
TET1	RABGAP1L	0.392904	1.27E-16	postive
TET2	RABGAP1L	0.510448	1.19E-28	postive
NSUN3	AL132642.	0.366506	1.64E-14	postive
TRDMT1	AL132642.	0.302386	3.88E-10	postive
NSUN6	AC092171.	0.360402	4.73E-14	postive
NSUN7	AC092171.	0.307681	1.84E-10	postive
RAD52	AC092171.	0.369712	9.29E-15	postive
NEIL1	AC092171.	0.496412	5.88E-27	postive
SMUG1	AC092171.	0.342211	9.82E-13	postive
RAD52	AC005840.	0.306109	2.30E-10	postive
NSUN7	LINC01003	0.329922	6.83E-12	postive
NEIL1	AC016526.	0.414638	1.65E-18	postive

NSUN7	CSRP3-AS	0.389772	2.31E-16	postive
RAD52	CSRP3-AS	0.309264	1.47E-10	postive
MBD1	CSRP3-AS	0.40771	6.83E-18	postive
NEIL1	CSRP3-AS	0.361484	3.93E-14	postive
DNMT3A	LINC01992	0.347556	4.11E-13	postive
RAD52	JPX	0.412466	2.59E-18	postive
NSUN3	AC087286.	0.539775	1.88E-32	postive
TRDMT1	AC087286.	0.35638	9.41E-14	postive
UHRF2	AC087286.	0.36264	3.22E-14	postive
ZBTB38	AC087286.	0.305095	2.66E-10	postive
TET1	AC087286.	0.600361	1.32E-41	postive
TET2	AC087286.	0.603072	4.60E-42	postive
TET3	AC087286.	0.300959	4.73E-10	postive
RAD52	AP001033.	0.31948	3.32E-11	postive
TET1	AP001033.	0.495663	7.20E-27	postive
TET2	AP001033.	0.383409	7.67E-16	postive
NSUN7	KIF9-AS1	0.319437	3.34E-11	postive
NEIL1	KIF9-AS1	0.320917	2.68E-11	postive
MECP2	LINC00092	0.397828	4.88E-17	postive
NSUN7	LINC01012	0.331327	5.49E-12	postive
DNMT3A	LINC01012	0.30482	2.76E-10	postive
NSUN6	LENG8-AS	0.408517	5.80E-18	postive
NSUN7	LENG8-AS	0.307884	1.79E-10	postive
RAD52	LENG8-AS	0.502497	1.11E-27	postive
NEIL1	LENG8-AS	0.464113	2.40E-23	postive
TET1	AC008537.	0.343127	8.47E-13	postive
NSUN3	SBF2-AS1	0.566155	3.31E-36	postive
TRDMT1	SBF2-AS1	0.343657	7.77E-13	postive
TET1	SBF2-AS1	0.349866	2.81E-13	postive
NSUN6	AC020663.	0.385533	5.16E-16	postive
DNMT1	AC020663.	-0.31772	4.31E-11	negative
NEIL1	AC020663.	0.355298	1.13E-13	postive
NTHL1	AC020663.	0.328852	8.05E-12	postive
SMUG1	AC020663.	0.445373	2.03E-21	postive
NSUN6	AL121652.	0.321013	2.64E-11	postive
TET1	AL121652.	0.681078	2.50E-57	postive
TET2	AL121652.	0.369025	1.05E-14	postive
TET3	AL121652.	0.328034	9.13E-12	postive
NSUN6	AC020663.	0.370367	8.27E-15	postive
RAD52	AC020663.	0.347631	4.06E-13	postive
NEIL1	AC020663.	0.343009	8.63E-13	postive
TET1	AC020663.	0.491736	2.07E-26	postive
TET2	AC020663.	0.323911	1.71E-11	postive
TET3	AC020663.	0.323494	1.82E-11	postive
DNMT3A	LINC01144	0.371469	6.79E-15	postive
NTHL1	LINC01144	0.322467	2.12E-11	postive
TET1	MAGEA10-	0.419606	5.86E-19	postive
TET2	CALML3-A	0.332354	4.68E-12	postive
TRDMT1	AC108868.	0.328848	8.05E-12	postive
NSUN3	AC104984.	0.521143	5.39E-30	postive
TRDMT1	AC104984.	0.351463	2.15E-13	postive
UHRF2	AC104984.	0.335651	2.79E-12	postive
TET1	AC104984.	0.605681	1.66E-42	postive
TET2	AC104984.	0.578918	3.79E-38	postive
NSUN3	AC073896.	0.377122	2.45E-15	postive
NSUN7	AC073896.	0.304286	2.98E-10	postive
DNMT3A	AC073896.	0.404181	1.39E-17	postive
RAD52	AC073896.	0.334512	3.34E-12	postive

MECP2	AC073896.	0.315505	5.96E-11	postive
TDG	AC073896.	0.308892	1.55E-10	postive
ZBTB33	AC073896.	0.366313	1.69E-14	postive
TET1	AC073896.	0.428306	9.13E-20	postive
TET2	AC073896.	0.385689	5.01E-16	postive
TET3	AC073896.	0.442588	3.83E-21	postive
NSUN6	AC097641.	0.404287	1.36E-17	postive
NSUN7	AC097641.	0.373964	4.34E-15	postive
RAD52	AC097641.	0.347949	3.85E-13	postive
NEIL1	AC097641.	0.319778	3.17E-11	postive
TET1	AC097641.	0.558372	4.59E-35	postive
TET2	AC097641.	0.424402	2.12E-19	postive
TET3	AC097641.	0.39306	1.23E-16	postive
NEIL1	AC092375.	0.352948	1.68E-13	postive
NSUN7	AC080013.	0.365563	1.93E-14	postive
NEIL1	AC080013.	0.376973	2.51E-15	postive
NSUN6	AP005432.	0.333812	3.73E-12	postive
NSUN7	AP005432.	0.306478	2.18E-10	postive
TET1	AP005432.	0.646505	5.19E-50	postive
TET2	AP005432.	0.363295	2.87E-14	postive
TET3	AP005432.	0.338393	1.81E-12	postive
NSUN6	AC104532.	0.512636	6.38E-29	postive
NSUN7	AC104532.	0.377071	2.47E-15	postive
RAD52	AC104532.	0.380803	1.24E-15	postive
MBD1	AC104532.	0.326359	1.18E-11	postive
NEIL1	AC104532.	0.471225	4.15E-24	postive
TET2	AC104532.	0.339416	1.54E-12	postive
TET3	AC104532.	0.400351	2.97E-17	postive
NSUN6	DANCR	0.322359	2.16E-11	postive
DNMT3A	DANCR	0.302651	3.74E-10	postive
NSUN3	LAMC1-AS	0.560222	2.47E-35	postive
TRDMT1	LAMC1-AS	0.401703	2.28E-17	postive
TET1	LAMC1-AS	0.541383	1.13E-32	postive
TET2	LAMC1-AS	0.465253	1.82E-23	postive
TET3	LAMC1-AS	0.309143	1.50E-10	postive
NSUN3	AC093423.	0.706791	1.84E-63	postive
TRDMT1	AC093423.	0.483789	1.68E-25	postive
UHRF2	AC093423.	0.343379	8.13E-13	postive
ZBTB38	AC093423.	0.306609	2.15E-10	postive
TET1	AC093423.	0.56052	2.24E-35	postive
TET2	AC093423.	0.48764	6.14E-26	postive
RAD52	FLJ12825	0.36757	1.36E-14	postive
NEIL1	FLJ12825	0.39664	6.16E-17	postive
SMUG1	FLJ12825	0.309096	1.51E-10	postive
NSUN3	AC104051.	0.440472	6.19E-21	postive
NSUN6	AC245060.	0.361862	3.68E-14	postive
RAD52	AC245060.	0.381625	1.07E-15	postive
NEIL1	AC245060.	0.416478	1.13E-18	postive
TET1	AC084880.	0.40742	7.25E-18	postive
TET2	AC084880.	0.541954	9.46E-33	postive
NSUN3	AC024075.	0.566163	3.30E-36	postive
MECP2	AC024075.	0.411889	2.91E-18	postive
ZBTB33	AC024075.	0.394523	9.29E-17	postive
ZBTB38	AC024075.	0.416793	1.06E-18	postive
TET1	AC024075.	0.396265	6.63E-17	postive
TET2	AC024075.	0.580305	2.30E-38	postive
TET3	AC024075.	0.473876	2.13E-24	postive
DNMT3A	ADGRD1-/	0.307613	1.86E-10	postive

NSUN6	CD2BP2-D	0.303624	3.26E-10	postive
NEIL1	CD2BP2-D	0.309978	1.33E-10	postive
NSUN6	CRTC3-AS	0.356289	9.56E-14	postive
NSUN7	CRTC3-AS	0.511101	9.89E-29	postive
DNMT3A	CRTC3-AS	0.349837	2.82E-13	postive
ZBTB33	CRTC3-AS	0.357365	7.96E-14	postive
TET1	CRTC3-AS	0.593869	1.56E-40	postive
TET2	CRTC3-AS	0.386995	3.92E-16	postive
TET3	CRTC3-AS	0.473283	2.48E-24	postive
NSUN6	PTOV1-AS	0.422805	2.98E-19	postive
NSUN7	PTOV1-AS	0.301394	4.45E-10	postive
RAD52	PTOV1-AS	0.563917	7.10E-36	postive
MBD1	PTOV1-AS	0.365634	1.91E-14	postive
NEIL1	PTOV1-AS	0.622622	1.74E-45	postive
NSUN3	LINC00630	0.464014	2.46E-23	postive
NSUN7	LINC00630	0.363508	2.77E-14	postive
DNMT3A	LINC00630	0.311802	1.02E-10	postive
TRDMT1	LINC00630	0.358079	7.05E-14	postive
UHRF2	LINC00630	0.385219	5.47E-16	postive
ZBTB33	LINC00630	0.508816	1.89E-28	postive
ZBTB38	LINC00630	0.405055	1.17E-17	postive
TET1	LINC00630	0.693229	3.80E-60	postive
TET2	LINC00630	0.66048	7.49E-53	postive
TET3	LINC00630	0.499416	2.59E-27	postive
NSUN7	AC011447.	0.343586	7.86E-13	postive
TET1	AC011447.	0.464327	2.28E-23	postive
TET2	AC011447.	0.440349	6.36E-21	postive
TET3	AC011447.	0.425501	1.67E-19	postive
DNMT3A	AC007743.	0.314321	7.09E-11	postive
TET1	AC016571.	0.464315	2.29E-23	postive
NSUN3	AC244093.	0.461225	4.85E-23	postive
TRDMT1	AC244093.	0.31415	7.27E-11	postive
UHRF2	AC244093.	0.332253	4.76E-12	postive
TET1	AC244093.	0.620388	4.40E-45	postive
TET2	AC244093.	0.548565	1.15E-33	postive
TET3	AC244093.	0.341942	1.03E-12	postive
NSUN6	AL662884.	0.319366	3.37E-11	postive
RAD52	AL662884.	0.338475	1.79E-12	postive
NEIL1	AL662884.	0.419601	5.87E-19	postive
NSUN6	AC007038.	0.415841	1.29E-18	postive
NSUN7	AC007038.	0.310803	1.18E-10	postive
RAD52	AC007038.	0.475155	1.55E-24	postive
NEIL1	AC007038.	0.517695	1.48E-29	postive
TET1	AC007038.	0.30412	3.05E-10	postive
TET2	AC007038.	0.306179	2.28E-10	postive
NSUN6	AC011978.	0.302027	4.08E-10	postive
TET1	AC011978.	0.407903	6.57E-18	postive
NSUN6	SLC25A25-	0.383097	8.13E-16	postive
NSUN7	SLC25A25-	0.402155	2.08E-17	postive
RAD52	SLC25A25-	0.362146	3.50E-14	postive
MBD1	SLC25A25-	0.304717	2.80E-10	postive
NEIL1	SLC25A25-	0.39018	2.14E-16	postive
TET3	SLC25A25-	0.352166	1.91E-13	postive
ZBTB38	AC097359.	0.33051	6.23E-12	postive
TET1	AC097359.	0.453871	2.81E-22	postive
TET2	AC097359.	0.361969	3.61E-14	postive
NSUN3	AC080023.	0.431813	4.25E-20	postive
ZBTB38	AC243772.	0.336147	2.58E-12	postive

TET2	AC243772.	0.495149	8.27E-27	postive
TET3	AC243772.	0.384545	6.21E-16	postive
NSUN3	LINC0045€	0.327899	9.32E-12	postive
NSUN7	LINC0045€	0.316862	4.89E-11	postive
TET1	LINC0045€	0.664897	8.80E-54	postive
TET2	LINC0045€	0.301567	4.35E-10	postive
TET3	LINC0045€	0.441083	5.39E-21	postive
NSUN6	AC002128.	0.452225	4.14E-22	postive
NSUN7	AC002128.	0.488342	5.10E-26	postive
DNMT3A	AC002128.	0.353547	1.52E-13	postive
RAD52	AC002128.	0.421718	3.75E-19	postive
MECP2	AC002128.	0.314256	7.15E-11	postive
NEIL1	AC002128.	0.419692	5.75E-19	postive
ZBTB33	AC002128.	0.339484	1.52E-12	postive
TET1	AC002128.	0.554625	1.59E-34	postive
TET2	AC002128.	0.500677	1.83E-27	postive
TET3	AC002128.	0.474211	1.96E-24	postive
DNMT3B	SNHG1	0.30517	2.63E-10	postive
RAD52	SNHG1	0.35659	9.08E-14	postive
NSUN3	AL353804.	0.569198	1.16E-36	postive
TRDMT1	AL353804.	0.386478	4.32E-16	postive
UHRF2	AL353804.	0.337943	1.94E-12	postive
TET1	AL353804.	0.663821	1.49E-53	postive
TET2	AL353804.	0.56862	1.42E-36	postive
TET3	AL353804.	0.332572	4.53E-12	postive
NSUN6	AP006621.	0.31279	8.85E-11	postive
NSUN7	AP006621.	0.329167	7.67E-12	postive
MBD1	AP006621.	0.447932	1.12E-21	postive
NEIL1	AP006621.	0.453044	3.41E-22	postive
NSUN6	AC007255.	0.362981	3.03E-14	postive
NSUN7	AC007255.	0.398787	4.05E-17	postive
TET1	AC007255.	0.346198	5.14E-13	postive
TET2	AC007255.	0.306465	2.19E-10	postive
TET3	AC007255.	0.331248	5.56E-12	postive
NEIL1	AL132655.	0.318282	3.96E-11	postive
TET1	AC007285.	0.515032	3.20E-29	postive
TET2	AC007285.	0.545908	2.69E-33	postive
TET3	AC007285.	0.349037	3.22E-13	postive
NSUN3	ARAP1-AS	0.604118	3.06E-42	postive
TRDMT1	ARAP1-AS	0.340905	1.21E-12	postive
UHRF2	ARAP1-AS	0.346815	4.64E-13	postive
ZBTB38	ARAP1-AS	0.339397	1.54E-12	postive
TET1	ARAP1-AS	0.539169	2.27E-32	postive
TET2	ARAP1-AS	0.624356	8.41E-46	postive
TET3	ARAP1-AS	0.321621	2.41E-11	postive
NSUN7	LINC01414	0.350866	2.38E-13	postive
TET1	LINC01414	0.589418	8.26E-40	postive
TET3	LINC01414	0.350148	2.68E-13	postive
NSUN4	DHRS4-AS	0.317771	4.27E-11	postive
NSUN7	DHRS4-AS	0.315859	5.66E-11	postive
MBD1	DHRS4-AS	0.311276	1.10E-10	postive
NSUN6	AC254562.	0.396251	6.64E-17	postive
NSUN7	AC254562.	0.325217	1.40E-11	postive
RAD52	AC254562.	0.345524	5.73E-13	postive
NEIL1	AC254562.	0.430638	5.49E-20	postive
NSUN7	LINC00664	0.342395	9.53E-13	postive
TET1	LINC00664	0.415567	1.36E-18	postive
TET3	LINC00664	0.305825	2.40E-10	postive

NSUN3	AP003171.	0.676501	2.65E-56	postive
TRDMT1	AP003171.	0.414136	1.83E-18	postive
TET1	AP003171.	0.354841	1.22E-13	postive
TET2	AP003171.	0.405071	1.16E-17	postive
NSUN5	HDAC4-AS	0.32193	2.30E-11	postive
MBD3	HDAC4-AS	0.312729	8.93E-11	postive
NTHL1	HDAC4-AS	0.396428	6.42E-17	postive
NSUN6	AC092171.	0.415789	1.30E-18	postive
DNMT1	AC092171.	-0.30497	2.70E-10	negative
NEIL1	AC092171.	0.379105	1.70E-15	postive
SMUG1	AC092171.	0.429936	6.40E-20	postive
NTHL1	SNHG30	0.307242	1.96E-10	postive
NSUN3	FO680682.	0.568075	1.71E-36	postive
TRDMT1	FO680682.	0.396813	5.96E-17	postive
UHRF2	FO680682.	0.339061	1.63E-12	postive
TET1	FO680682.	0.686375	1.53E-58	postive
TET2	FO680682.	0.535579	6.93E-32	postive
TET3	FO680682.	0.315524	5.94E-11	postive
NSUN3	AC008870.	0.458809	8.67E-23	postive
TRDMT1	AC008870.	0.314802	6.61E-11	postive
RAD52	AC008870.	0.307014	2.03E-10	postive
UHRF2	AC008870.	0.323676	1.77E-11	postive
ZBTB33	AC008870.	0.338499	1.78E-12	postive
ZBTB38	AC008870.	0.320493	2.85E-11	postive
TET1	AC008870.	0.552245	3.47E-34	postive
TET2	AC008870.	0.612565	1.07E-43	postive
TET3	AC008870.	0.408803	5.47E-18	postive
NSUN7	AC107032.	0.393011	1.24E-16	postive
DNMT3A	AC107032.	0.302959	3.58E-10	postive
TET1	AC107032.	0.35036	2.59E-13	postive
TET3	AC107032.	0.305385	2.55E-10	postive
NSUN6	EXTL3-AS1	0.473682	2.24E-24	postive
RAD52	EXTL3-AS1	0.406567	8.61E-18	postive
MBD1	EXTL3-AS1	0.321126	2.60E-11	postive
NEIL1	EXTL3-AS1	0.534486	9.70E-32	postive
TET2	EXTL3-AS1	0.310441	1.24E-10	postive
TET1	MIR181A2	0.407917	6.55E-18	postive
RAD52	DTX2P1-U	0.302804	3.66E-10	postive
TET1	DTX2P1-U	0.345771	5.51E-13	postive
TET2	DTX2P1-U	0.404369	1.34E-17	postive
TET3	DTX2P1-U	0.315749	5.75E-11	postive
NSUN3	AC024270.	0.345601	5.66E-13	postive
UHRF2	AC024270.	0.337745	2.01E-12	postive
TET1	AC024270.	0.568863	1.30E-36	postive
TET2	AC024270.	0.616712	2.00E-44	postive
TET3	AC024270.	0.363526	2.76E-14	postive
NSUN6	AL031666.	0.338679	1.73E-12	postive
NSUN7	AL031666.	0.312953	8.64E-11	postive
TET1	AL031666.	0.682673	1.09E-57	postive
TET2	AL031666.	0.482793	2.18E-25	postive
TET3	AL031666.	0.364222	2.44E-14	postive
NSUN6	AL161452.	0.399008	3.87E-17	postive
RAD52	AL161452.	0.440308	6.42E-21	postive
NEIL1	AL161452.	0.498174	3.64E-27	postive
NSUN3	SGMS1-AS	0.589437	8.20E-40	postive
NSUN7	SGMS1-AS	0.301833	4.19E-10	postive
TRDMT1	SGMS1-AS	0.386656	4.18E-16	postive
TET1	SGMS1-AS	0.583552	7.12E-39	postive

TET2	SGMS1-AS	0.431758	4.30E-20	postive
NSUN6	AL049795.	0.328548	8.43E-12	postive
NSUN7	AL049795.	0.349654	2.91E-13	postive
RAD52	AL049795.	0.325603	1.32E-11	postive
MBD1	AL049795.	0.300919	4.76E-10	postive
NEIL1	AL049795.	0.487012	7.25E-26	postive
NEIL1	AC126773.	0.304794	2.77E-10	postive
NSUN6	AC093462.	0.442831	3.62E-21	postive
MBD1	AC093462.	0.311757	1.03E-10	postive
NEIL1	AC093462.	0.419325	6.22E-19	postive
NSUN3	AL117350.	0.358452	6.61E-14	postive
RAD52	AL117350.	0.345042	6.20E-13	postive
TET1	AL117350.	0.381613	1.07E-15	postive
TET2	AL117350.	0.361547	3.89E-14	postive
TET3	AL117350.	0.301546	4.36E-10	postive
NEIL1	AL355472.	0.367753	1.31E-14	postive
NSUN7	LINC01424	0.320277	2.95E-11	postive
DNMT3A	LINC01424	0.364729	2.24E-14	postive
RAD52	LINC01424	0.34705	4.47E-13	postive
NEIL1	LINC01424	0.349835	2.82E-13	postive
TET1	LINC01424	0.353324	1.58E-13	postive
NSUN3	AC073487.	0.540865	1.33E-32	postive
NSUN6	AC073487.	0.364684	2.25E-14	postive
NSUN7	AC073487.	0.318609	3.78E-11	postive
DNMT3A	AC073487.	0.300903	4.77E-10	postive
TRDMT1	AC073487.	0.31336	8.15E-11	postive
RAD52	AC073487.	0.392674	1.33E-16	postive
MECP2	AC073487.	0.325974	1.25E-11	postive
NEIL1	AC073487.	0.304802	2.77E-10	postive
UHRF2	AC073487.	0.309332	1.46E-10	postive
TET1	AC073487.	0.590443	5.64E-40	postive
TET2	AC073487.	0.610991	2.02E-43	postive
TET3	AC073487.	0.414559	1.68E-18	postive
NSUN3	AL157871.	0.450513	6.17E-22	postive
UHRF2	AL157871.	0.347044	4.47E-13	postive
ZBTB33	AL157871.	0.311481	1.07E-10	postive
ZBTB38	AL157871.	0.317957	4.16E-11	postive
TET1	AL157871.	0.541579	1.06E-32	postive
TET2	AL157871.	0.661877	3.82E-53	postive
TET3	AL157871.	0.387487	3.57E-16	postive
YBX1	AL512353.	0.597095	4.60E-41	postive
NSUN7	C15orf56	0.310406	1.25E-10	postive
NSUN3	AC015853.	0.40633	9.03E-18	postive
TET1	AC015853.	0.461533	4.50E-23	postive
TET2	AC015853.	0.506111	4.05E-28	postive
NSUN3	AL354726.	0.514139	4.14E-29	postive
TRDMT1	AL354726.	0.37307	5.09E-15	postive
UHRF2	AL354726.	0.396674	6.12E-17	postive
ZBTB33	AL354726.	0.301039	4.68E-10	postive
TET1	AL354726.	0.648583	2.01E-50	postive
TET2	AL354726.	0.608855	4.73E-43	postive
TET3	AL354726.	0.360393	4.74E-14	postive
TET1	AC012085.	0.496038	6.51E-27	postive
TET2	AC012085.	0.350889	2.37E-13	postive
DNMT3A	LINC01342	0.301986	4.10E-10	postive
NSUN6	AC008124.	0.461768	4.25E-23	postive
NSUN7	AC008124.	0.451961	4.40E-22	postive
DNMT3A	AC008124.	0.375973	3.01E-15	postive

RAD52	AC008124.	0.391713	1.60E-16	postive
MBD1	AC008124.	0.367418	1.39E-14	postive
NEIL1	AC008124.	0.389489	2.44E-16	postive
TDG	AC008124.	0.309448	1.43E-10	postive
ZBTB33	AC008124.	0.310307	1.27E-10	postive
TET1	AC008124.	0.317414	4.50E-11	postive
TET2	AC008124.	0.379642	1.54E-15	postive
TET3	AC008124.	0.40092	2.66E-17	postive
NSUN7	AC006252.	0.334022	3.61E-12	postive
NSUN6	AC009090.	0.401149	2.54E-17	postive
RAD52	AC009090.	0.411953	2.88E-18	postive
NEIL1	AC009090.	0.467565	1.03E-23	postive
TET2	AC009090.	0.374903	3.66E-15	postive
NSUN3	AC018653.	0.30199	4.10E-10	postive
RAD52	AC018653.	0.410203	4.12E-18	postive
MECP2	AC018653.	0.306436	2.20E-10	postive
ZBTB33	AC018653.	0.403024	1.75E-17	postive
TET2	AC018653.	0.447357	1.28E-21	postive
TET3	AC018653.	0.303752	3.21E-10	postive
NSUN7	AC008771.	0.341593	1.08E-12	postive
NSUN3	AC107308.	0.419339	6.20E-19	postive
TRDMT1	AC107308.	0.376539	2.72E-15	postive
UHRF2	AC107308.	0.403383	1.63E-17	postive
NSUN6	AC020765.	0.33737	2.13E-12	postive
NEIL1	AC020765.	0.341406	1.12E-12	postive
NTHL1	AC020765.	0.350386	2.57E-13	postive
SMUG1	AC020765.	0.309076	1.51E-10	postive
NSUN6	AC011330.	0.430479	5.69E-20	postive
ALYREF	AC011330.	-0.31452	6.89E-11	negative
RAD52	AC011330.	0.381065	1.19E-15	postive
NEIL1	AC011330.	0.587683	1.57E-39	postive
TET2	AC011330.	0.338835	1.69E-12	postive
SMUG1	LINC02601	0.32712	1.05E-11	postive
NSUN6	AL359220.	0.449884	7.15E-22	postive
NSUN7	AL359220.	0.35013	2.69E-13	postive
RAD52	AL359220.	0.303645	3.25E-10	postive
ZBTB33	AL359220.	0.359235	5.78E-14	postive
TET1	AL359220.	0.461295	4.77E-23	postive
TET2	AL359220.	0.594681	1.15E-40	postive
TET3	AL359220.	0.520389	6.73E-30	postive
NSUN7	CCDC183-	0.313617	7.85E-11	postive
MBD1	CCDC183-	0.403459	1.61E-17	postive
TET3	CCDC183-	0.327842	9.40E-12	postive
NEIL1	VASH1-AS	0.339865	1.43E-12	postive
SMUG1	VASH1-AS	0.30759	1.87E-10	postive
NSUN3	AC108449.	0.581702	1.39E-38	postive
TRDMT1	AC108449.	0.403712	1.53E-17	postive
MECP2	AC108449.	0.349063	3.21E-13	postive
UHRF2	AC108449.	0.338046	1.91E-12	postive
ZBTB33	AC108449.	0.370051	8.74E-15	postive
ZBTB38	AC108449.	0.381534	1.09E-15	postive
TET1	AC108449.	0.684901	3.36E-58	postive
TET2	AC108449.	0.691334	1.07E-59	postive
TET3	AC108449.	0.458917	8.45E-23	postive
NSUN7	CENPN-AS	0.321126	2.60E-11	postive
TET1	CENPN-AS	0.305842	2.39E-10	postive
NSUN6	AC127024.	0.314432	6.97E-11	postive
NSUN7	AC127024.	0.425171	1.79E-19	postive

DNMT3A	AC127024.	0.370429	8.18E-15	postive
RAD52	AC127024.	0.441156	5.30E-21	postive
NEIL1	AC127024.	0.392072	1.49E-16	postive
TET1	AC127024.	0.508821	1.89E-28	postive
TET2	AC127024.	0.358116	7.00E-14	postive
TET3	AC127024.	0.333047	4.20E-12	postive
NSUN5	AL355353.	0.347862	3.91E-13	postive
MBD3	AL355353.	0.318538	3.82E-11	postive
NTHL1	AL355353.	0.491258	2.35E-26	postive
SMUG1	AL355353.	0.414476	1.71E-18	postive
NSUN3	AC004884.	0.444219	2.64E-21	postive
TRDMT1	AC004884.	0.315497	5.97E-11	postive
UHRF2	AC004884.	0.332505	4.57E-12	postive
TET1	AC004884.	0.656471	5.07E-52	postive
TET2	AC004884.	0.577381	6.56E-38	postive
TET3	AC004884.	0.344147	7.18E-13	postive
NSUN7	LINC0282(	0.312792	8.85E-11	postive
DNMT3A	LINC0282(	0.393048	1.24E-16	postive
TDG	LINC0282(	0.367261	1.43E-14	postive
ZBTB33	LINC0282(	0.374897	3.66E-15	postive
TET1	LINC0282(	0.498221	3.59E-27	postive
TET2	LINC0282(	0.342319	9.65E-13	postive
TET3	LINC0282(	0.475835	1.30E-24	postive
NSUN7	AC016355.	0.349516	2.97E-13	postive
DNMT3A	AC016355.	0.326315	1.19E-11	postive
NSUN3	AC009088.	0.35399	1.41E-13	postive
TET1	AC009088.	0.471326	4.05E-24	postive
MECP2	AC105206.	0.324416	1.58E-11	postive
TET1	AC105206.	0.420801	4.55E-19	postive
TET2	AC105206.	0.366547	1.63E-14	postive
MBD3	FAM225A	0.346243	5.10E-13	postive
NSUN6	AC245884.	0.34985	2.81E-13	postive
NSUN7	AC245884.	0.342177	9.87E-13	postive
TET1	AC245884.	0.614326	5.27E-44	postive
TET2	AC245884.	0.505586	4.69E-28	postive
TET3	AC245884.	0.397488	5.22E-17	postive
NSUN7	AC068888.	0.331826	5.08E-12	postive
RAD52	AC068888.	0.31538	6.07E-11	postive
TET3	DGUOK-A	0.33857	1.76E-12	postive
NSUN5	AC046143.	0.323013	1.96E-11	postive
MBD3	AC046143.	0.366727	1.57E-14	postive
NTHL1	AC046143.	0.368982	1.06E-14	postive
SMUG1	AC046143.	0.389895	2.26E-16	postive
NSUN5	ELFN1-AS:	0.305884	2.38E-10	postive
NSUN6	AL353622.	0.300608	4.97E-10	postive
NEIL1	AL353622.	0.342179	9.87E-13	postive
NSUN6	AC010422.	0.309558	1.41E-10	postive
MBD3	AC010422.	0.341173	1.16E-12	postive
NEIL1	AC010422.	0.52816	6.67E-31	postive
RAD52	FAM66C	0.469723	6.04E-24	postive
TET2	FAM66C	0.306994	2.03E-10	postive
RAD52	AC013731.	0.351747	2.05E-13	postive
NEIL1	AC013731.	0.468474	8.23E-24	postive
NSUN3	AL512506.	0.370194	8.52E-15	postive
NSUN6	AL512506.	0.340102	1.38E-12	postive
NSUN7	AL512506.	0.322802	2.02E-11	postive
TET1	AL512506.	0.697107	4.47E-61	postive
TET2	AL512506.	0.437059	1.33E-20	postive

TET3	AL512506.	0.35165	2.09E-13	postive
DNMT3A	AL031985.	0.316736	4.98E-11	postive
YBX1	AL031985.	0.62999	7.70E-47	postive
NSUN6	AP001630.	0.342663	9.12E-13	postive
NSUN7	AP001630.	0.315493	5.97E-11	postive
TET1	AP001630.	0.642451	3.25E-49	postive
TET2	AP001630.	0.317647	4.35E-11	postive
TET3	AP001630.	0.348301	3.64E-13	postive
NSUN5	AP003068.	0.41279	2.42E-18	postive
MBD3	AP003068.	0.389514	2.43E-16	postive
NTHL1	AP003068.	0.363143	2.95E-14	postive
NSUN3	ZNF8-ERV	0.356661	8.97E-14	postive
TRDMT1	ZNF8-ERV	0.315644	5.84E-11	postive
TDG	ZNF8-ERV	0.32369	1.77E-11	postive
UHRF2	ZNF8-ERV	0.33478	3.20E-12	postive
ZBTB33	ZNF8-ERV	0.366209	1.72E-14	postive
TET1	ZNF8-ERV	0.417088	9.94E-19	postive
TET2	ZNF8-ERV	0.559371	3.29E-35	postive
TET3	ZNF8-ERV	0.358954	6.07E-14	postive
NSUN7	AL109936.	0.399278	3.67E-17	postive
NSUN6	SEC24B-A!	0.364791	2.21E-14	postive
NSUN7	SEC24B-A!	0.443879	2.85E-21	postive
RAD52	SEC24B-A!	0.37219	5.97E-15	postive
NEIL1	SEC24B-A!	0.490656	2.76E-26	postive
TET2	SEC24B-A!	0.307627	1.86E-10	postive
NSUN6	AC116914.	0.312529	9.19E-11	postive
RAD52	AC116914.	0.36239	3.36E-14	postive
MBD1	AC116914.	0.320949	2.67E-11	postive
NEIL1	AC116914.	0.493695	1.22E-26	postive
NSUN6	U47924.1	0.460945	5.19E-23	postive
NSUN7	U47924.1	0.378593	1.87E-15	postive
RAD52	U47924.1	0.404671	1.26E-17	postive
NEIL1	U47924.1	0.346696	4.73E-13	postive
SMUG1	U47924.1	0.355071	1.17E-13	postive
TET1	U47924.1	0.470606	4.85E-24	postive
NSUN7	AC008669.	0.335521	2.85E-12	postive
NSUN6	DPP9-AS1	0.352671	1.76E-13	postive
RAD52	DPP9-AS1	0.399589	3.46E-17	postive
NEIL1	DPP9-AS1	0.401406	2.41E-17	postive
TET2	DPP9-AS1	0.315524	5.95E-11	postive
NSUN6	AC018616.	0.317001	4.79E-11	postive
NSUN7	AC018616.	0.313861	7.58E-11	postive
TET1	AC018616.	0.623749	1.09E-45	postive
TET2	AC018616.	0.326877	1.09E-11	postive
TET3	AC018616.	0.364848	2.19E-14	postive
NSUN6	AC112722.	0.413925	1.92E-18	postive
NSUN7	AC112722.	0.355801	1.04E-13	postive
TET1	AC112722.	0.581789	1.35E-38	postive
TET2	AC112722.	0.446504	1.56E-21	postive
NSUN6	ZNF775-A	0.302267	3.95E-10	postive
TET1	ZNF775-A	0.528762	5.56E-31	postive
TET2	ZNF775-A	0.433616	2.86E-20	postive
TET3	ZNF775-A	0.374329	4.06E-15	postive
NSUN3	AC008735.	0.391846	1.56E-16	postive
NSUN6	AC008735.	0.362656	3.21E-14	postive
DNMT3A	AC008735.	0.369911	8.97E-15	postive
RAD52	AC008735.	0.420693	4.66E-19	postive
MBD1	AC008735.	0.352806	1.72E-13	postive

MECP2	AC008735.	0.316526	5.13E-11	postive
NEIL1	AC008735.	0.379134	1.69E-15	postive
ZBTB33	AC008735.	0.304559	2.86E-10	postive
TET1	AC008735.	0.308109	1.73E-10	postive
TET2	AC008735.	0.461313	4.75E-23	postive
TET3	AC008735.	0.436617	1.47E-20	postive
NSUN7	PRKAR2A-	0.380484	1.32E-15	postive
NEIL1	AC034236.	0.403843	1.49E-17	postive
TET1	AC025031.	0.54064	1.43E-32	postive
TET2	AC025031.	0.505436	4.89E-28	postive
TET3	AC025031.	0.306121	2.30E-10	postive
ZBTB33	OGFRP1	0.315263	6.18E-11	postive
NSUN3	AC100823.	0.349268	3.10E-13	postive
TET1	AC100823.	0.628766	1.30E-46	postive
TET2	AC100823.	0.310002	1.32E-10	postive
TET3	AC100823.	0.311258	1.10E-10	postive
NEIL1	ASMTL-AS	0.483383	1.87E-25	postive
TET1	AP003559.	0.323041	1.95E-11	postive
TET2	AP003559.	0.407126	7.69E-18	postive
TET3	AP003559.	0.342347	9.60E-13	postive
NSUN3	DCUN1D2	0.397117	5.61E-17	postive
NSUN7	DCUN1D2	0.348802	3.35E-13	postive
MECP2	DCUN1D2	0.318024	4.12E-11	postive
UHRF2	DCUN1D2	0.352048	1.95E-13	postive
ZBTB33	DCUN1D2	0.371401	6.87E-15	postive
TET1	DCUN1D2	0.472232	3.23E-24	postive
TET2	DCUN1D2	0.598413	2.78E-41	postive
TET3	DCUN1D2	0.420311	5.05E-19	postive
NSUN6	SPAG5-AS	0.43613	1.64E-20	postive
NSUN7	SPAG5-AS	0.419709	5.73E-19	postive
DNMT3A	SPAG5-AS	0.31874	3.70E-11	postive
RAD52	SPAG5-AS	0.395903	7.11E-17	postive
NEIL1	SPAG5-AS	0.32901	7.86E-12	postive
ZBTB33	SPAG5-AS	0.334953	3.12E-12	postive
TET1	SPAG5-AS	0.602196	6.47E-42	postive
TET2	SPAG5-AS	0.490348	3.00E-26	postive
TET3	SPAG5-AS	0.492354	1.76E-26	postive
NSUN6	AF131215.	0.391695	1.60E-16	postive
MBD1	AF131215.	0.355993	1.01E-13	postive
NEIL1	AF131215.	0.363635	2.71E-14	postive
ZBTB33	AF131215.	0.329513	7.27E-12	postive
TET2	AF131215.	0.330289	6.45E-12	postive
NSUN6	AC008543.	0.418782	6.97E-19	postive
NSUN7	AC008543.	0.362037	3.57E-14	postive
RAD52	AC008543.	0.310624	1.21E-10	postive
TET1	AC008543.	0.594931	1.05E-40	postive
TET2	AC008543.	0.52348	2.70E-30	postive
TET3	AC008543.	0.445	2.21E-21	postive
NSUN6	AC092910.	0.334588	3.30E-12	postive
NSUN7	AC092910.	0.468413	8.35E-24	postive
DNMT3A	AC092910.	0.432901	3.35E-20	postive
RAD52	AC092910.	0.329303	7.51E-12	postive
MBD1	AC092910.	0.350864	2.38E-13	postive
MBD4	AC092910.	0.363274	2.88E-14	postive
MECP2	AC092910.	0.38979	2.31E-16	postive
ZBTB33	AC092910.	0.462259	3.77E-23	postive
TET1	AC092910.	0.468128	8.96E-24	postive
TET2	AC092910.	0.431608	4.44E-20	postive

TET3	AC092910.	0.488821	4.50E-26	postive
DNMT3A	LRRC8D-D	0.357268	8.09E-14	postive
NSUN6	THAP9-AS	0.347614	4.07E-13	postive
NSUN7	THAP9-AS	0.375084	3.54E-15	postive
RAD52	THAP9-AS	0.379572	1.56E-15	postive
MBD1	THAP9-AS	0.306733	2.11E-10	postive
NEIL1	THAP9-AS	0.438513	9.61E-21	postive
NSUN6	AP002840.	0.326588	1.14E-11	postive
DNMT1	AP002840.	-0.31835	3.92E-11	negative
NEIL1	AP002840.	0.416159	1.21E-18	postive
UHRF1	AP002840.	-0.33111	5.68E-12	negative
NSUN4	AL604028.	0.336909	2.29E-12	postive
NSUN6	AL604028.	0.353151	1.62E-13	postive
NSUN7	AL604028.	0.341678	1.07E-12	postive
NEIL1	AL604028.	0.400597	2.83E-17	postive
SMUG1	AL604028.	0.328754	8.17E-12	postive
RAD52	AC004918.	0.323399	1.85E-11	postive
NEIL1	AC004918.	0.400273	3.02E-17	postive
NSUN7	AC015802.	0.311605	1.05E-10	postive
RAD52	AC015802.	0.301536	4.37E-10	postive
NEIL1	AC015802.	0.419268	6.29E-19	postive
DNMT3A	LINC02696	0.316074	5.48E-11	postive
NSUN3	AL138831.	0.354625	1.27E-13	postive
TET1	AL138831.	0.348999	3.24E-13	postive
TET2	AL138831.	0.419988	5.41E-19	postive
ZBTB33	AC006206.	0.343851	7.53E-13	postive
TET1	AC006206.	0.354098	1.38E-13	postive
TET3	AC006206.	0.317235	4.62E-11	postive
NSUN6	AC006042.	0.558451	4.47E-35	postive
NSUN7	AC006042.	0.404047	1.43E-17	postive
RAD52	AC006042.	0.410286	4.05E-18	postive
MBD1	AC006042.	0.35451	1.29E-13	postive
NEIL1	AC006042.	0.506855	3.29E-28	postive
TET3	AC006042.	0.345175	6.07E-13	postive
NSUN6	AC087741.	0.472813	2.79E-24	postive
RAD52	AC087741.	0.40564	1.04E-17	postive
NEIL1	AC087741.	0.609455	3.73E-43	postive
MBD3	LINC01357	0.316476	5.17E-11	postive
NSUN3	AC006059.	0.473429	2.39E-24	postive
UHRF2	AC006059.	0.329656	7.11E-12	postive
ZBTB38	AC006059.	0.328365	8.68E-12	postive
TET1	AC006059.	0.581434	1.53E-38	postive
TET2	AC006059.	0.613029	8.90E-44	postive
TET3	AC006059.	0.345835	5.45E-13	postive
NSUN6	AC087392.	0.346578	4.83E-13	postive
RAD52	AC087392.	0.393672	1.10E-16	postive
NEIL1	AC087392.	0.44914	8.50E-22	postive
TET1	AC087392.	0.310919	1.16E-10	postive
TET2	AC087392.	0.397063	5.67E-17	postive
NSUN6	AC004908.	0.383017	8.26E-16	postive
NSUN7	AC004908.	0.322623	2.07E-11	postive
RAD52	AC004908.	0.403384	1.63E-17	postive
NEIL1	AC004908.	0.405654	1.03E-17	postive
TET1	AC004908.	0.4222	3.38E-19	postive
TET2	AC004908.	0.494337	1.03E-26	postive
SMUG1	HAGLROS	0.330201	6.54E-12	postive
MECP2	AP003486.	0.417181	9.74E-19	postive
ZBTB38	AP003486.	0.306664	2.13E-10	postive

TET2	AP003486.	0.348745	3.38E-13	postive
NSUN3	AC019254.	0.332987	4.24E-12	postive
NEIL1	AC005261.	0.356305	9.53E-14	postive
NSUN7	AL445309.	0.307246	1.96E-10	postive
TET1	AL445309.	0.654756	1.14E-51	postive
TET2	AL445309.	0.551419	4.54E-34	postive
TET3	AL445309.	0.381894	1.02E-15	postive
NSUN6	AC008738.	0.311758	1.03E-10	postive
NSUN7	AC008738.	0.313744	7.71E-11	postive
DNMT3A	AC008738.	0.319534	3.29E-11	postive
DNMT3B	AC008738.	0.332566	4.53E-12	postive
SMUG1	AC008738.	0.348978	3.25E-13	postive
TET3	AC008738.	0.342905	8.78E-13	postive
NEIL1	AC106028.	0.329447	7.35E-12	postive
TET1	AC106028.	0.334047	3.59E-12	postive
TET2	AC106028.	0.394024	1.02E-16	postive
NSUN3	AC234775.	0.328568	8.41E-12	postive
ZBTB33	AC234775.	0.370332	8.32E-15	postive
ZBTB38	AC234775.	0.353725	1.47E-13	postive
TET1	AC234775.	0.526333	1.15E-30	postive
TET2	AC234775.	0.619502	6.35E-45	postive
TET3	AC234775.	0.43446	2.37E-20	postive
NSUN3	MIR302CH	0.365298	2.02E-14	postive
NSUN6	MIR302CH	0.403071	1.73E-17	postive
NSUN7	MIR302CH	0.337578	2.06E-12	postive
RAD52	MIR302CH	0.390944	1.85E-16	postive
NEIL1	MIR302CH	0.378339	1.96E-15	postive
TET1	MIR302CH	0.532516	1.78E-31	postive
TET2	MIR302CH	0.588388	1.21E-39	postive
TET3	MIR302CH	0.369928	8.94E-15	postive
RAD52	AL354836.	0.309056	1.51E-10	postive
NEIL1	AL354836.	0.494736	9.25E-27	postive
NSUN6	AC026782.	0.347321	4.27E-13	postive
TET1	AC026782.	0.544644	4.03E-33	postive
TET2	AC026782.	0.462803	3.31E-23	postive
NSUN5	AL023803.	0.300395	5.12E-10	postive
MBD3	AL023803.	0.389807	2.30E-16	postive
NTHL1	AL023803.	0.340233	1.35E-12	postive
MBD1	AL118506.	0.323252	1.89E-11	postive
ZBTB33	AL118506.	0.307801	1.81E-10	postive
TET3	AL118506.	0.323231	1.89E-11	postive
NSUN7	Z98884.2	0.328	9.17E-12	postive
DNMT3A	Z98884.2	0.304248	2.99E-10	postive
RAD52	Z98884.2	0.417114	9.88E-19	postive
MECP2	Z98884.2	0.324805	1.49E-11	postive
UHRF2	Z98884.2	0.336002	2.64E-12	postive
ZBTB33	Z98884.2	0.331046	5.74E-12	postive
TET1	Z98884.2	0.477227	9.13E-25	postive
TET2	Z98884.2	0.538923	2.45E-32	postive
TET3	Z98884.2	0.370614	7.91E-15	postive
RAD52	MZF1-AS1	0.333993	3.63E-12	postive
NEIL1	MZF1-AS1	0.314953	6.46E-11	postive
RAD52	AC145423.	0.329023	7.84E-12	postive
MBD3	AC145423.	0.318246	3.98E-11	postive
NEIL1	AC145423.	0.354703	1.25E-13	postive
SMUG1	AC145423.	0.306051	2.32E-10	postive
TET1	AC008676.	0.477427	8.68E-25	postive
NSUN3	AC011933.	0.521568	4.76E-30	postive

TRDMT1	AC011933.	0.401623	2.31E-17	postive
TET1	AC011933.	0.691871	7.97E-60	postive
TET2	AC011933.	0.47325	2.50E-24	postive
TET3	AC011933.	0.308457	1.65E-10	postive
NSUN6	AC011921.	0.346037	5.27E-13	postive
NSUN7	AC011921.	0.445435	2.00E-21	postive
DNMT3A	AC011921.	0.414687	1.64E-18	postive
RAD52	AC011921.	0.336438	2.47E-12	postive
TET3	AC011921.	0.375191	3.47E-15	postive
NEIL1	AC244034.	0.340006	1.40E-12	postive
NSUN5	AC027644.	0.328754	8.17E-12	postive
MBD3	AC027644.	0.362785	3.14E-14	postive
NTHL1	AC027644.	0.429531	6.99E-20	postive
SMUG1	AC027644.	0.341601	1.08E-12	postive
TET1	AC009054.	0.38352	7.52E-16	postive
TET2	AC009054.	0.368178	1.22E-14	postive
NTHL1	ILRUN-AS1	0.410533	3.85E-18	postive
SMUG1	ILRUN-AS1	0.403499	1.59E-17	postive
NSUN3	AC078852.	0.350544	2.51E-13	postive
NSUN6	AC078852.	0.321873	2.32E-11	postive
NSUN7	AC078852.	0.3034	3.37E-10	postive
TET1	AC078852.	0.624536	7.80E-46	postive
TET2	AC078852.	0.471088	4.30E-24	postive
TET3	AC078852.	0.31827	3.97E-11	postive
NSUN6	AC124242.	0.304211	3.01E-10	postive
NSUN7	AC124242.	0.327071	1.06E-11	postive
NSUN6	AL021707.	0.334387	3.41E-12	postive
NSUN7	AL021707.	0.333592	3.86E-12	postive
DNMT3A	AL021707.	0.450178	6.67E-22	postive
RAD52	AL021707.	0.419543	5.94E-19	postive
MBD1	AL021707.	0.322267	2.19E-11	postive
NEIL1	AL021707.	0.351961	1.98E-13	postive
TET2	AL021707.	0.324868	1.48E-11	postive
TET3	AL021707.	0.331058	5.73E-12	postive
NSUN7	AC106820.	0.364546	2.31E-14	postive
DNMT3A	AC106820.	0.394965	8.53E-17	postive
NTHL1	AC106820.	0.360031	5.05E-14	postive
TET1	AC106820.	0.400413	2.94E-17	postive
NSUN6	AL671710.	0.39289	1.27E-16	postive
RAD52	AL671710.	0.369566	9.53E-15	postive
NEIL1	AL671710.	0.495274	8.00E-27	postive
SMUG1	AL671710.	0.311058	1.14E-10	postive
NSUN6	MIR34AHG	0.455584	1.87E-22	postive
RAD52	MIR34AHG	0.303981	3.11E-10	postive
NEIL1	MIR34AHG	0.372648	5.50E-15	postive
TET2	MIR34AHG	0.408907	5.36E-18	postive
TET3	MIR34AHG	0.336666	2.38E-12	postive
NSUN6	AC092171.	0.329763	7.00E-12	postive
NSUN6	AC135803.	0.413483	2.10E-18	postive
NSUN3	AC004918.	0.447009	1.39E-21	postive
UHRF2	AC004918.	0.315779	5.73E-11	postive
ZBTB33	AC004918.	0.361796	3.72E-14	postive
ZBTB38	AC004918.	0.305347	2.56E-10	postive
TET1	AC004918.	0.539488	2.05E-32	postive
TET2	AC004918.	0.642743	2.85E-49	postive
TET3	AC004918.	0.400616	2.82E-17	postive
NSUN6	AC023908.	0.461315	4.74E-23	postive
NSUN7	AC023908.	0.339833	1.44E-12	postive

DNMT3A	AC023908.	0.306222	2.27E-10	postive
RAD52	AC023908.	0.400271	3.02E-17	postive
MBD1	AC023908.	0.36118	4.14E-14	postive
NEIL1	AC023908.	0.511943	7.78E-29	postive
NSUN3	FAM13A-A	0.423794	2.41E-19	postive
NSUN6	FAM13A-A	0.33743	2.11E-12	postive
NSUN7	FAM13A-A	0.324931	1.46E-11	postive
RAD52	FAM13A-A	0.339062	1.63E-12	postive
NEIL1	FAM13A-A	0.313809	7.63E-11	postive
TET1	FAM13A-A	0.553624	2.21E-34	postive
TET2	FAM13A-A	0.516345	2.19E-29	postive
TET3	FAM13A-A	0.321129	2.60E-11	postive
NSUN3	AC073046.	0.342058	1.01E-12	postive
NSUN6	AC073046.	0.454681	2.32E-22	postive
TET1	AC073046.	0.373956	4.34E-15	postive
TET2	AC073046.	0.440206	6.57E-21	postive
TET3	AC073046.	0.586624	2.32E-39	postive
MBD3	AC016876.	0.316809	4.92E-11	postive
NSUN3	AL606534.	0.571471	5.26E-37	postive
TRDMT1	AL606534.	0.420788	4.57E-19	postive
UHRF2	AL606534.	0.349047	3.21E-13	postive
ZBTB38	AL606534.	0.311462	1.07E-10	postive
TET1	AL606534.	0.427112	1.18E-19	postive
TET2	AL606534.	0.485855	9.82E-26	postive
NSUN6	ZFHX2-AS	0.511066	9.99E-29	postive
NSUN7	ZFHX2-AS	0.369761	9.21E-15	postive
RAD52	ZFHX2-AS	0.493746	1.21E-26	postive
MBD1	ZFHX2-AS	0.315075	6.35E-11	postive
NEIL1	ZFHX2-AS	0.571559	5.10E-37	postive
SMUG1	ZFHX2-AS	0.315921	5.61E-11	postive
NSUN7	DNAH10O	0.306295	2.24E-10	postive
DNMT3A	DNAH10O	0.432923	3.33E-20	postive
TET1	DNAH10O	0.33741	2.12E-12	postive
DNMT3A	TIMMDC1-	0.318639	3.76E-11	postive
RAD52	TIMMDC1-	0.313415	8.09E-11	postive
NEIL1	TIMMDC1-	0.353903	1.43E-13	postive
NSUN6	AC090515.	0.312573	9.13E-11	postive
NEIL1	AC090515.	0.448356	1.02E-21	postive
NSUN6	AC105020.	0.332457	4.61E-12	postive
NEIL1	AC105020.	0.507806	2.52E-28	postive
NSUN6	AC068620.	0.411185	3.37E-18	postive
NSUN7	AC068620.	0.339418	1.54E-12	postive
RAD52	AC068620.	0.420063	5.32E-19	postive
NEIL1	AC068620.	0.593458	1.82E-40	postive
NSUN6	AC055855.	0.441796	4.58E-21	postive
NSUN7	AC055855.	0.329483	7.31E-12	postive
RAD52	AC055855.	0.511656	8.44E-29	postive
MBD1	AC055855.	0.31763	4.36E-11	postive
NEIL1	AC055855.	0.624805	6.96E-46	postive
TET2	AC055855.	0.409012	5.25E-18	postive
DNMT1	AC025176.	0.3241	1.66E-11	postive
NEIL1	LINC01011	0.332399	4.65E-12	postive
NSUN5	AC139530.	0.319718	3.20E-11	postive
NSUN7	AC139530.	0.342333	9.63E-13	postive
ALYREF	AC139530.	0.345098	6.15E-13	postive
MBD3	AC139530.	0.362832	3.11E-14	postive
NEIL1	AC139530.	0.337493	2.09E-12	postive
NTHL1	AC139530.	0.421925	3.59E-19	postive

SMUG1	AC139530.	0.377241	2.39E-15	postive
NSUN6	AL353708.	0.392406	1.40E-16	postive
NSUN7	AL353708.	0.315066	6.36E-11	postive
RAD52	AL353708.	0.309727	1.38E-10	postive
NEIL1	AL353708.	0.441228	5.21E-21	postive
NSUN6	AC004076.	0.398128	4.61E-17	postive
NSUN7	AC004076.	0.366089	1.76E-14	postive
DNMT3A	AC004076.	0.300962	4.73E-10	postive
RAD52	AC004076.	0.408333	6.02E-18	postive
MECP2	AC004076.	0.310853	1.17E-10	postive
NEIL1	AC004076.	0.449041	8.70E-22	postive
ZBTB33	AC004076.	0.368415	1.17E-14	postive
TET1	AC004076.	0.346153	5.17E-13	postive
TET2	AC004076.	0.492928	1.50E-26	postive
TET3	AC004076.	0.459704	7.00E-23	postive
NSUN6	AC108471.	0.378706	1.83E-15	postive
NSUN7	AC108471.	0.397999	4.72E-17	postive
DNMT3A	AC108471.	0.323696	1.77E-11	postive
NEIL1	AC108471.	0.33826	1.85E-12	postive
DNMT3A	AC009955.	0.302551	3.79E-10	postive
TET1	AC009955.	0.553285	2.47E-34	postive
DNMT3A	LINC02571	0.316977	4.80E-11	postive
TET3	LINC02571	0.303205	3.46E-10	postive
NSUN6	AL512652.	0.316483	5.17E-11	postive
RAD52	AL512652.	0.331189	5.61E-12	postive
NEIL1	AL512652.	0.443068	3.43E-21	postive
NSUN5	AL035461.	0.328643	8.31E-12	postive
MBD3	AL035461.	0.386696	4.14E-16	postive
NTHL1	AL035461.	0.305155	2.63E-10	postive
NSUN6	AL117336.	0.33687	2.30E-12	postive
NSUN7	AL117336.	0.333342	4.01E-12	postive
DNMT3A	AL117336.	0.38014	1.41E-15	postive
RAD52	AL117336.	0.382422	9.22E-16	postive
NEIL1	AL117336.	0.340203	1.36E-12	postive
NEIL1	TRPM2-AS	0.321895	2.31E-11	postive
NSUN3	AL590133.	0.421322	4.08E-19	postive
TET1	AL590133.	0.434632	2.28E-20	postive
TET2	AL590133.	0.407976	6.47E-18	postive
TET3	AL590133.	0.330218	6.52E-12	postive
NSUN6	PIK3IP1-D	0.318416	3.88E-11	postive
TET1	PIK3IP1-D	0.300502	5.04E-10	postive
NEIL1	AL122125.	0.392356	1.41E-16	postive
NSUN6	ZNF213-A	0.384018	6.85E-16	postive
NSUN7	ZNF213-A	0.334121	3.55E-12	postive
DNMT3A	ZNF213-A	0.309782	1.37E-10	postive
RAD52	ZNF213-A	0.336773	2.34E-12	postive
NEIL1	ZNF213-A	0.466441	1.36E-23	postive
SMUG1	AC016065.	0.302199	3.98E-10	postive
NSUN6	TBILA	0.434099	2.57E-20	postive
ALYREF	TBILA	-0.30557	2.48E-10	negative
RAD52	TBILA	0.365335	2.01E-14	postive
NEIL1	TBILA	0.31978	3.17E-11	postive
UHRF1	TBILA	-0.30144	4.42E-10	negative
NSUN3	AC103858.	0.34909	3.19E-13	postive
ZBTB38	AC103858.	0.315402	6.05E-11	postive
TET2	AC103858.	0.31172	1.03E-10	postive
NSUN6	SRI-AS1	0.373255	4.93E-15	postive
NSUN7	SRI-AS1	0.348963	3.26E-13	postive

TET1	SRI-AS1	0.660429	7.67E-53	postive
TET2	SRI-AS1	0.4299	6.45E-20	postive
TET3	SRI-AS1	0.371263	7.04E-15	postive
NSUN3	AC004943.	0.347906	3.88E-13	postive
UHRF2	AC004943.	0.345244	6.00E-13	postive
TET1	AC004943.	0.553112	2.61E-34	postive
TET2	AC004943.	0.580393	2.23E-38	postive
TET3	AC004943.	0.322149	2.23E-11	postive
NEIL1	AL033527.	0.327908	9.31E-12	postive
RAD52	LINC0287[illegible]	0.498309	3.51E-27	postive
NSUN7	AC074044.	0.338979	1.65E-12	postive
NEIL1	AC074044.	0.321472	2.47E-11	postive
RAD52	MRPL20-[illegible]	0.311015	1.14E-10	postive
MBD3	MRPL20-[illegible]	0.304288	2.97E-10	postive
MECP2	MRPL20-[illegible]	0.300684	4.91E-10	postive
NEIL1	MRPL20-[illegible]	0.419114	6.50E-19	postive
NSUN3	AL356124.	0.67069	5.02E-55	postive
TRDMT1	AL356124.	0.394689	9.00E-17	postive
ZBTB38	AL356124.	0.32971	7.05E-12	postive
TET1	AL356124.	0.647242	3.71E-50	postive
TET2	AL356124.	0.520323	6.87E-30	postive
TET3	AL356124.	0.310913	1.16E-10	postive
NSUN7	LMNTD2-[illegible]	0.302552	3.79E-10	postive
NEIL1	LMNTD2-[illegible]	0.483668	1.74E-25	postive
NSUN7	SRD5A3-A	0.350523	2.52E-13	postive
NSUN6	AC080129.	0.426093	1.47E-19	postive
NSUN7	AC080129.	0.343707	7.71E-13	postive
MBD1	AC080129.	0.325698	1.30E-11	postive
NEIL1	AC080129.	0.474597	1.78E-24	postive
NSUN7	MIR2052H	0.343361	8.15E-13	postive
TET1	MIR2052H	0.662793	2.45E-53	postive
TET2	MIR2052H	0.379082	1.71E-15	postive
TET3	MIR2052H	0.423067	2.81E-19	postive
NSUN3	AC087276.	0.436939	1.37E-20	postive
NEIL1	AC087276.	0.301423	4.44E-10	postive
UHRF2	AC087276.	0.336415	2.48E-12	postive
ZBTB33	AC087276.	0.330491	6.25E-12	postive
ZBTB38	AC087276.	0.301463	4.41E-10	postive
TET1	AC087276.	0.506505	3.63E-28	postive
TET2	AC087276.	0.648925	1.71E-50	postive
TET3	AC087276.	0.390736	1.93E-16	postive
MECP2	AC093627.	0.316777	4.95E-11	postive
NTHL1	AC026304.	0.390704	1.94E-16	postive
SMUG1	AC026304.	0.362185	3.48E-14	postive
NSUN6	SNHG14	0.398311	4.44E-17	postive
TET1	SNHG14	0.494555	9.71E-27	postive
TET2	SNHG14	0.355801	1.04E-13	postive
TET3	SNHG14	0.353494	1.53E-13	postive
NSUN3	AL356019.	0.388023	3.23E-16	postive
RAD52	AL356019.	0.355754	1.05E-13	postive
MECP2	AL356019.	0.368226	1.21E-14	postive
ZBTB33	AL356019.	0.338301	1.84E-12	postive
ZBTB38	AL356019.	0.316296	5.31E-11	postive
TET1	AL356019.	0.334021	3.61E-12	postive
TET2	AL356019.	0.546896	1.96E-33	postive
NSUN3	AC012557.	0.425211	1.78E-19	postive
NSUN6	AC012557.	0.326598	1.14E-11	postive
NSUN7	AC012557.	0.347022	4.49E-13	postive

TRDMT1	AC012557.	0.366402	1.67E-14	postive
TET1	AC012557.	0.665872	5.46E-54	postive
TET2	AC012557.	0.517094	1.76E-29	postive
TET3	AC012557.	0.367779	1.31E-14	postive
NTHL1	SNHG9	0.346563	4.84E-13	postive
SMUG1	SNHG9	0.30071	4.90E-10	postive
ZBTB38	SNHG9	-0.34138	1.12E-12	negative
NSUN3	AC008114.	0.3627	3.18E-14	postive
TET1	AC008114.	0.576264	9.75E-38	postive
TET2	AC008114.	0.544099	4.80E-33	postive
TET3	AC008114.	0.368044	1.25E-14	postive
NSUN6	WASIR2	0.360826	4.40E-14	postive
NSUN6	AC025287.	0.344181	7.14E-13	postive
NSUN7	AC025287.	0.327584	9.78E-12	postive
RAD52	AC025287.	0.325773	1.29E-11	postive
NEIL1	AC025287.	0.403892	1.47E-17	postive
ZBTB33	AC025287.	0.315937	5.60E-11	postive
TET1	AC025287.	0.47847	6.65E-25	postive
TET2	AC025287.	0.579038	3.63E-38	postive
TET3	AC025287.	0.380647	1.28E-15	postive
NSUN6	AL590096.	0.302352	3.90E-10	postive
RAD52	AL590096.	0.310035	1.32E-10	postive
NEIL1	AL590096.	0.387622	3.48E-16	postive
RAD52	AC073288.	0.318264	3.97E-11	postive
NEIL1	AC073288.	0.339455	1.53E-12	postive
NTHL1	AC083973.	0.320485	2.86E-11	postive
RAD52	AL603832.	0.316634	5.05E-11	postive
RAD52	AC067930.	0.324606	1.54E-11	postive
TET3	AC067930.	0.351637	2.09E-13	postive
SMUG1	TIPARP-AS	0.377106	2.45E-15	postive
NSUN3	AC011815.	0.3748	3.73E-15	postive
TRDMT1	AC011815.	0.323703	1.76E-11	postive
UHRF2	AC011815.	0.331957	4.98E-12	postive
TET1	AC011815.	0.491573	2.16E-26	postive
TET2	AC011815.	0.379231	1.66E-15	postive
NSUN6	AC023794.	0.327267	1.03E-11	postive
NSUN7	AC023794.	0.322029	2.27E-11	postive
TET1	AC023794.	0.655785	7.01E-52	postive
TET2	AC023794.	0.500287	2.04E-27	postive
TET3	AC023794.	0.377344	2.35E-15	postive
NSUN3	AC139887.	0.407377	7.31E-18	postive
NSUN6	AC139887.	0.349434	3.01E-13	postive
NSUN7	AC139887.	0.432106	3.98E-20	postive
DNMT3A	AC139887.	0.41498	1.54E-18	postive
TRDMT1	AC139887.	0.309883	1.35E-10	postive
RAD52	AC139887.	0.348474	3.53E-13	postive
MECP2	AC139887.	0.368322	1.19E-14	postive
UHRF2	AC139887.	0.312619	9.07E-11	postive
ZBTB33	AC139887.	0.430892	5.20E-20	postive
TET1	AC139887.	0.655277	8.90E-52	postive
TET2	AC139887.	0.591097	4.42E-40	postive
TET3	AC139887.	0.541545	1.08E-32	postive
NSUN3	AL031710.	0.344574	6.69E-13	postive
TET1	AL031710.	0.611017	2.00E-43	postive
TET2	AL031710.	0.377515	2.28E-15	postive
TET1	AC093382.	0.519753	8.12E-30	postive
NEIL1	HOXB-AS3	0.402733	1.86E-17	postive
DNMT3A	AP000880.	0.345118	6.13E-13	postive

NSUN6	LINC01675	0.345143	6.10E-13	postive
TET1	LINC01675	0.60797	6.73E-43	postive
TET3	LINC01675	0.321333	2.52E-11	postive
NSUN6	AC017083.	0.364375	2.38E-14	postive
DNMT3A	AC017083.	0.401674	2.29E-17	postive
RAD52	AC017083.	0.364828	2.20E-14	postive
NEIL1	AC017083.	0.390973	1.84E-16	postive
NSUN3	AP005899.	0.498116	3.70E-27	postive
NSUN7	AP005899.	0.337622	2.05E-12	postive
TRDMT1	AP005899.	0.390929	1.86E-16	postive
UHRF2	AP005899.	0.4148	1.60E-18	postive
ZBTB33	AP005899.	0.353083	1.64E-13	postive
ZBTB38	AP005899.	0.325877	1.27E-11	postive
TET1	AP005899.	0.553654	2.19E-34	postive
TET2	AP005899.	0.566552	2.89E-36	postive
TET3	AP005899.	0.4153	1.44E-18	postive
NSUN3	AL731566.	0.397217	5.50E-17	postive
NSUN6	AL731566.	0.36864	1.12E-14	postive
NSUN7	AL731566.	0.328308	8.75E-12	postive
TRDMT1	AL731566.	0.301066	4.66E-10	postive
RAD52	AL731566.	0.367774	1.31E-14	postive
MECP2	AL731566.	0.310653	1.20E-10	postive
UHRF2	AL731566.	0.326613	1.13E-11	postive
ZBTB33	AL731566.	0.360093	4.99E-14	postive
TET1	AL731566.	0.647949	2.68E-50	postive
TET2	AL731566.	0.613631	6.98E-44	postive
TET3	AL731566.	0.478697	6.27E-25	postive
NEIL1	LINC01176	0.514435	3.81E-29	postive
TRDMT1	VIM-AS1	0.386192	4.56E-16	postive
NSUN3	AL355102.	0.767062	7.48E-81	postive
TRDMT1	AL355102.	0.468735	7.71E-24	postive
TET1	AL355102.	0.375223	3.45E-15	postive
TET2	AL355102.	0.388374	3.02E-16	postive
NSUN6	AL354892.	0.447536	1.23E-21	postive
RAD52	AL354892.	0.444926	2.25E-21	postive
MBD1	AL354892.	0.300366	5.14E-10	postive
NEIL1	AL354892.	0.473916	2.11E-24	postive
TET2	AL354892.	0.400159	3.09E-17	postive
DNMT3A	CACNA1C	0.312483	9.25E-11	postive
NSUN6	AC100821.	0.402226	2.05E-17	postive
NSUN7	AC100821.	0.306662	2.13E-10	postive
RAD52	AC100821.	0.320502	2.85E-11	postive
TET1	AC100821.	0.515269	2.99E-29	postive
TET2	AC100821.	0.420188	5.18E-19	postive
TET3	AC100821.	0.384561	6.19E-16	postive
NSUN3	AC007314.	0.423491	2.57E-19	postive
NSUN6	AC007314.	0.303708	3.23E-10	postive
UHRF2	AC007314.	0.328262	8.81E-12	postive
TET1	AC007314.	0.56868	1.39E-36	postive
TET2	AC007314.	0.571502	5.21E-37	postive
TET3	AC007314.	0.35667	8.96E-14	postive
NSUN3	AC007684.	0.340935	1.21E-12	postive
NSUN6	AC007684.	0.344312	6.98E-13	postive
NSUN7	AC007684.	0.30304	3.54E-10	postive
TET1	AC007684.	0.646876	4.38E-50	postive
TET2	AC007684.	0.541656	1.04E-32	postive
TET3	AC007684.	0.348185	3.71E-13	postive
NEIL1	AC124016.	0.40586	9.92E-18	postive

NSUN3	AC068790.	0.36734	1.41E-14	postive
NSUN7	AC068790.	0.305514	2.50E-10	postive
UHRF2	AC068790.	0.333608	3.85E-12	postive
ZBTB33	AC068790.	0.308994	1.53E-10	postive
TET1	AC068790.	0.678552	9.26E-57	postive
TET2	AC068790.	0.595447	8.61E-41	postive
TET3	AC068790.	0.374681	3.81E-15	postive
NSUN6	AC018695.	0.411126	3.41E-18	postive
RAD52	AC018695.	0.333795	3.74E-12	postive
NEIL1	AC018695.	0.477191	9.22E-25	postive
NSUN3	AC138207.	0.773531	4.94E-83	postive
TRDMT1	AC138207.	0.444373	2.55E-21	postive
TET1	AC138207.	0.505518	4.78E-28	postive
TET2	AC138207.	0.407481	7.16E-18	postive
NSUN7	AL513477.	0.311425	1.08E-10	postive
RAD52	AL513477.	0.393834	1.06E-16	postive
NEIL1	AL513477.	0.438248	1.02E-20	postive
TET1	AC124312.	0.577228	6.93E-38	postive
TET2	AC124312.	0.308809	1.57E-10	postive
TET3	AC124312.	0.335459	2.88E-12	postive
NSUN6	LINC00894	0.444835	2.29E-21	postive
NSUN7	LINC00894	0.350509	2.52E-13	postive
RAD52	LINC00894	0.443035	3.46E-21	postive
MECP2	LINC00894	0.322173	2.22E-11	postive
NEIL1	LINC00894	0.412132	2.77E-18	postive
TET1	LINC00894	0.449773	7.33E-22	postive
TET2	LINC00894	0.448649	9.53E-22	postive
TET3	LINC00894	0.343745	7.66E-13	postive
TET2	AP001205.	0.434602	2.30E-20	postive
TET3	AP001205.	0.343915	7.45E-13	postive
RAD52	AL162741.	0.36173	3.76E-14	postive
NEIL1	AL162741.	0.390569	1.99E-16	postive
TET1	AL162741.	0.367396	1.40E-14	postive
NSUN6	AC027243.	0.34374	7.67E-13	postive
NSUN7	AC027243.	0.314807	6.60E-11	postive
DNMT3A	AC027243.	0.330053	6.69E-12	postive
RAD52	AC027243.	0.311112	1.13E-10	postive
TET1	AC027243.	0.626372	3.59E-46	postive
TET2	AC027243.	0.354728	1.24E-13	postive
TET3	AC027243.	0.388064	3.20E-16	postive
NSUN6	AL021707.	0.368788	1.09E-14	postive
RAD52	AL021707.	0.351734	2.06E-13	postive
NEIL1	AL021707.	0.515404	2.88E-29	postive
NSUN7	HCG15	0.321615	2.41E-11	postive
DNMT1	HCG15	0.310125	1.30E-10	postive
NSUN5	SNHG25	0.344494	6.78E-13	postive
MBD3	SNHG25	0.434195	2.51E-20	postive
NTHL1	SNHG25	0.492668	1.61E-26	postive
SMUG1	SNHG25	0.316626	5.06E-11	postive
NSUN3	SLFNL1-A[illegible]	0.310217	1.28E-10	postive
RAD52	SLFNL1-A[illegible]	0.308481	1.64E-10	postive
TET1	SLFNL1-A[illegible]	0.409641	4.62E-18	postive
TET2	SLFNL1-A[illegible]	0.314361	7.05E-11	postive
TET3	SLFNL1-A[illegible]	0.337684	2.03E-12	postive
NSUN6	AL109659.	0.430998	5.08E-20	postive
RAD52	AL109659.	0.319327	3.39E-11	postive
NEIL1	AL109659.	0.528968	5.22E-31	postive
NSUN3	AC110769.	0.466836	1.23E-23	postive

NSUN6	AC110769.	0.32053	2.84E-11	postive
NSUN7	AC110769.	0.311678	1.04E-10	postive
DNMT3A	AC110769.	0.308454	1.65E-10	postive
TRDMT1	AC110769.	0.35495	1.20E-13	postive
RAD52	AC110769.	0.341542	1.09E-12	postive
MECP2	AC110769.	0.309262	1.47E-10	postive
UHRF2	AC110769.	0.3186	3.78E-11	postive
ZBTB33	AC110769.	0.326165	1.21E-11	postive
TET1	AC110769.	0.654966	1.03E-51	postive
TET2	AC110769.	0.512126	7.38E-29	postive
TET3	AC110769.	0.357943	7.21E-14	postive
NSUN3	AL391095.	0.31449	6.91E-11	postive
RAD52	AL391095.	0.332485	4.59E-12	postive
TET1	AL391095.	0.437257	1.27E-20	postive
TET2	AL391095.	0.350546	2.51E-13	postive
TET3	AL391095.	0.318736	3.70E-11	postive
NSUN6	LINC0141(	0.355423	1.11E-13	postive
NEIL1	LINC0141(	0.350572	2.50E-13	postive
RAD52	AL360181.	0.394415	9.49E-17	postive
MBD1	AL360181.	0.333964	3.64E-12	postive
NEIL1	AL360181.	0.345047	6.20E-13	postive
TET2	AL360181.	0.386661	4.17E-16	postive
RAD52	AC005841.	0.40667	8.43E-18	postive
NEIL1	AC005841.	0.335868	2.70E-12	postive
TET2	AC005841.	0.514292	3.97E-29	postive
TET3	AC005841.	0.364825	2.20E-14	postive
NSUN3	LINC0257	0.457236	1.26E-22	postive
TRDMT1	LINC0257	0.325144	1.42E-11	postive
NSUN3	AC020978.	0.66447	1.08E-53	postive
TRDMT1	AC020978.	0.32645	1.16E-11	postive
ZBTB38	AC020978.	0.307955	1.77E-10	postive
TET1	AC020978.	0.53722	4.17E-32	postive
TET2	AC020978.	0.500774	1.78E-27	postive
TET3	AC020978.	0.343834	7.55E-13	postive
TET1	AC004839.	0.317161	4.67E-11	postive
NSUN3	AL035530.	0.453803	2.85E-22	postive
TRDMT1	AL035530.	0.311185	1.12E-10	postive
TET1	AL035530.	0.38615	4.59E-16	postive
TET2	AL035530.	0.387827	3.35E-16	postive
NTHL1	AL023803.	0.412715	2.46E-18	postive
TET2	AC022364.	0.330228	6.51E-12	postive
TDG	AC132807.	0.32946	7.33E-12	postive
TET1	AC132807.	0.467124	1.15E-23	postive
TET2	AC132807.	0.406873	8.09E-18	postive
TET3	AC132807.	0.40971	4.55E-18	postive
NEIL1	AC010719.	0.426907	1.24E-19	postive
NSUN3	UBOX5-AS	0.346729	4.71E-13	postive
NSUN6	UBOX5-AS	0.376949	2.52E-15	postive
NSUN7	UBOX5-AS	0.302256	3.95E-10	postive
RAD52	UBOX5-AS	0.41826	7.77E-19	postive
NEIL1	UBOX5-AS	0.390835	1.89E-16	postive
TET1	UBOX5-AS	0.517275	1.67E-29	postive
TET2	UBOX5-AS	0.50865	1.98E-28	postive
TET3	UBOX5-AS	0.321663	2.40E-11	postive
UNG	AL391834.	0.377219	2.40E-15	postive
NSUN6	AL021707.	0.458655	9.00E-23	postive
NSUN7	AL021707.	0.384021	6.85E-16	postive
DNMT3A	AL021707.	0.333766	3.76E-12	postive

RAD52	AL021707.	0.429351	7.28E-20	postive
MBD1	AL021707.	0.333177	4.12E-12	postive
MECP2	AL021707.	0.330778	5.98E-12	postive
NEIL1	AL021707.	0.393665	1.10E-16	postive
ZBTB33	AL021707.	0.356589	9.08E-14	postive
TET1	AL021707.	0.502335	1.16E-27	postive
TET2	AL021707.	0.551143	4.97E-34	postive
TET3	AL021707.	0.455581	1.87E-22	postive
NSUN5	GS1-124K!	0.374971	3.62E-15	postive
NTHL1	GS1-124K!	0.39997	3.21E-17	postive
NSUN3	LAMTOR5-	0.382384	9.29E-16	postive
NSUN7	LAMTOR5-	0.400174	3.08E-17	postive
DNMT3A	LAMTOR5-	0.300862	4.80E-10	postive
TRDMT1	LAMTOR5-	0.307364	1.93E-10	postive
ZBTB33	LAMTOR5-	0.326386	1.17E-11	postive
TET1	LAMTOR5-	0.741874	5.40E-73	postive
TET2	LAMTOR5-	0.462272	3.76E-23	postive
TET3	LAMTOR5-	0.40952	4.73E-18	postive
NSUN3	AC005070.	0.423431	2.60E-19	postive
UHRF2	AC005070.	0.332434	4.63E-12	postive
TET1	AC005070.	0.596104	6.71E-41	postive
TET2	AC005070.	0.633452	1.73E-47	postive
TET3	AC005070.	0.372782	5.37E-15	postive
NEIL1	AL158151.	0.315885	5.64E-11	postive
DNMT3A	SOX1-OT	0.317779	4.27E-11	postive
TET1	AL139260.	0.491451	2.23E-26	postive
NSUN6	AL390728.	0.418999	6.66E-19	postive
RAD52	AL390728.	0.488651	4.70E-26	postive
NEIL1	AL390728.	0.582265	1.14E-38	postive
NSUN3	AL139383.	0.698669	1.87E-61	postive
TRDMT1	AL139383.	0.406385	8.93E-18	postive
TET1	AL139383.	0.321381	2.50E-11	postive
TET2	AL139383.	0.336514	2.44E-12	postive
NSUN6	MIS18A-A	0.318813	3.66E-11	postive
NSUN7	MIS18A-A	0.433432	2.98E-20	postive
DNMT3A	MIS18A-A	0.414992	1.54E-18	postive
RAD52	MIS18A-A	0.355123	1.16E-13	postive
NEIL1	MIS18A-A	0.330853	5.91E-12	postive
TET1	MIS18A-A	0.471706	3.68E-24	postive
TET2	MIS18A-A	0.425526	1.66E-19	postive
TET3	MIS18A-A	0.372488	5.66E-15	postive
NSUN6	AC005828.	0.317141	4.69E-11	postive
NSUN7	AC005828.	0.304398	2.93E-10	postive
TET1	AC005828.	0.663982	1.38E-53	postive
TET2	AC005828.	0.467112	1.15E-23	postive
TET3	AC005828.	0.352401	1.84E-13	postive
NSUN7	AC002451.	0.334705	3.24E-12	postive
TET1	AC002451.	0.49552	7.49E-27	postive
DNMT1	AL513165.	0.413925	1.92E-18	postive
MBD3	AL513165.	0.337303	2.15E-12	postive
DNMT3A	AC093249.	0.370478	8.10E-15	postive
RAD52	AC093249.	0.347205	4.35E-13	postive
NEIL1	AC093249.	0.327071	1.06E-11	postive
NSUN6	AC079174.	0.338663	1.73E-12	postive
DNMT3A	AC079174.	0.324985	1.45E-11	postive
RAD52	AC079174.	0.377263	2.38E-15	postive
NEIL1	AC079174.	0.425457	1.69E-19	postive
NSUN6	AC010132.	0.391326	1.72E-16	postive

NSUN7	AC010132.	0.361405	3.98E-14	postive
ZBTB33	AC010132.	0.305376	2.55E-10	postive
TET1	AC010132.	0.60905	4.38E-43	postive
TET2	AC010132.	0.496888	5.16E-27	postive
TET3	AC010132.	0.450145	6.73E-22	postive
NTHL1	AC025162.	0.316705	5.00E-11	postive
SMUG1	AC025162.	0.353911	1.43E-13	postive
RAD52	H1-10-AS	0.307781	1.82E-10	postive
MBD3	H1-10-AS	0.410458	3.91E-18	postive
NEIL1	H1-10-AS	0.470327	5.19E-24	postive
NEIL1	AC009133.	0.340162	1.36E-12	postive
DNMT3A	GRASLND	0.306405	2.21E-10	postive
NSUN3	MKNK1-AS	0.697904	2.87E-61	postive
TRDMT1	MKNK1-AS	0.44261	3.81E-21	postive
UHRF2	MKNK1-AS	0.36892	1.07E-14	postive
ZBTB38	MKNK1-AS	0.338749	1.71E-12	postive
TET1	MKNK1-AS	0.521075	5.50E-30	postive
TET2	MKNK1-AS	0.561377	1.68E-35	postive
TET3	MKNK1-AS	0.307602	1.86E-10	postive
NSUN6	ZNF436-A	0.570818	6.61E-37	postive
NSUN7	ZNF436-A	0.459571	7.22E-23	postive
DNMT3A	ZNF436-A	0.384972	5.73E-16	postive
RAD52	ZNF436-A	0.426243	1.43E-19	postive
NEIL1	ZNF436-A	0.479782	4.75E-25	postive
TET1	ZNF436-A	0.369941	8.92E-15	postive
TET2	ZNF436-A	0.402575	1.91E-17	postive
TET3	ZNF436-A	0.516331	2.20E-29	postive
NSUN3	AC005104.	0.339936	1.41E-12	postive
NSUN6	AC005104.	0.344443	6.84E-13	postive
NSUN7	AC005104.	0.307636	1.85E-10	postive
RAD52	AC005104.	0.342804	8.92E-13	postive
NEIL1	AC005104.	0.309264	1.47E-10	postive
TET1	AC005104.	0.528853	5.41E-31	postive
TET2	AC005104.	0.630127	7.26E-47	postive
TET3	AC005104.	0.379129	1.69E-15	postive
NSUN3	AL359921.	0.404958	1.19E-17	postive
NSUN6	AL359921.	0.306445	2.20E-10	postive
RAD52	AL359921.	0.353702	1.48E-13	postive
NEIL1	AL359921.	0.371151	7.19E-15	postive
TET1	AL359921.	0.38903	2.67E-16	postive
TET2	AL359921.	0.574092	2.10E-37	postive
NSUN3	HM13-IT1	0.358135	6.98E-14	postive
DNMT3A	HM13-IT1	0.341819	1.05E-12	postive
RAD52	HM13-IT1	0.334289	3.46E-12	postive
MECP2	HM13-IT1	0.369243	1.01E-14	postive
ZBTB33	HM13-IT1	0.349536	2.96E-13	postive
TET2	HM13-IT1	0.426625	1.31E-19	postive
TET3	HM13-IT1	0.398343	4.42E-17	postive
NSUN3	AC068533.	0.380355	1.35E-15	postive
TET1	AC068533.	0.612742	1.00E-43	postive
TET2	AC068533.	0.558277	4.74E-35	postive
TET3	AC068533.	0.350738	2.43E-13	postive
NSUN3	ATP1B3-A	0.455117	2.09E-22	postive
UHRF2	ATP1B3-A	0.345348	5.90E-13	postive
TET1	ATP1B3-A	0.379801	1.50E-15	postive
TET2	ATP1B3-A	0.577306	6.74E-38	postive
NSUN3	LINC0140	0.415506	1.38E-18	postive
NSUN6	LINC0140	0.360123	4.97E-14	postive

NSUN7	LINC0140(	0.393457	1.14E-16	postive
DNMT3A	LINC0140(	0.32629	1.19E-11	postive
RAD52	LINC0140(	0.377611	2.24E-15	postive
MECP2	LINC0140(	0.322685	2.06E-11	postive
ZBTB33	LINC0140(	0.341734	1.06E-12	postive
TET1	LINC0140(	0.638094	2.26E-48	postive
TET2	LINC0140(	0.52335	2.81E-30	postive
TET3	LINC0140(	0.482148	2.58E-25	postive
NSUN3	AL596223.	0.777583	1.95E-84	postive
NSUN6	AC105219.	0.324094	1.66E-11	postive
NEIL1	AC105219.	0.399525	3.50E-17	postive
NSUN3	AC004637.	0.44845	9.97E-22	postive
UHRF2	AC004637.	0.354494	1.29E-13	postive
TET1	AC004637.	0.554024	1.94E-34	postive
TET2	AC004637.	0.556633	8.19E-35	postive
SMUG1	MAPKAPK!	0.354459	1.30E-13	postive
UNG	MAPKAPK!	0.319549	3.28E-11	postive
NSUN3	AC104564.	0.496971	5.05E-27	postive
TRDMT1	AC104564.	0.314529	6.87E-11	postive
UHRF2	AC104564.	0.320961	2.66E-11	postive
TET1	AC104564.	0.530024	3.79E-31	postive
TET2	AC104564.	0.517716	1.47E-29	postive
NSUN6	AC084876.	0.335429	2.89E-12	postive
RAD52	AC084876.	0.34483	6.42E-13	postive
NEIL1	AC084876.	0.465444	1.74E-23	postive
TET1	AC084876.	0.379342	1.63E-15	postive
TET2	AC084876.	0.370476	8.11E-15	postive
NSUN6	NBR2	0.390727	1.93E-16	postive
NSUN7	NBR2	0.426467	1.36E-19	postive
DNMT3A	NBR2	0.322233	2.20E-11	postive
NEIL1	NBR2	0.418306	7.70E-19	postive
TET3	NBR2	0.317121	4.70E-11	postive
NTHL1	AC005363.	0.373731	4.52E-15	postive
SMUG1	AC005363.	0.301231	4.56E-10	postive
NSUN3	ANKRD10-	0.40073	2.76E-17	postive
NSUN6	ANKRD10-	0.337105	2.22E-12	postive
NSUN7	ANKRD10-	0.359525	5.50E-14	postive
DNMT3A	ANKRD10-	0.324347	1.60E-11	postive
TRDMT1	ANKRD10-	0.316405	5.22E-11	postive
RAD52	ANKRD10-	0.401111	2.56E-17	postive
MBD1	ANKRD10-	0.334009	3.62E-12	postive
MECP2	ANKRD10-	0.3735	4.72E-15	postive
NEIL1	ANKRD10-	0.344069	7.27E-13	postive
UHRF2	ANKRD10-	0.35731	8.03E-14	postive
ZBTB33	ANKRD10-	0.357606	7.64E-14	postive
TET1	ANKRD10-	0.411979	2.86E-18	postive
TET2	ANKRD10-	0.564047	6.79E-36	postive
TET3	ANKRD10-	0.442041	4.34E-21	postive
ALYREF	MAFG-DT	0.38371	7.26E-16	postive
MBD3	MAFG-DT	0.376718	2.63E-15	postive
NTHL1	MAFG-DT	0.421839	3.65E-19	postive
NSUN3	AP003900.	0.790838	3.06E-89	postive
TRDMT1	AP003900.	0.455309	2.00E-22	postive
TET1	AP003900.	0.300908	4.77E-10	postive
TET2	AP003900.	0.307488	1.89E-10	postive
NSUN6	AC010761.	0.359416	5.61E-14	postive
DNMT3A	AC010761.	0.336458	2.46E-12	postive
RAD52	AC010761.	0.330558	6.19E-12	postive

MBD1	AC010761.	0.412427	2.61E-18	postive
NEIL1	AC010761.	0.385753	4.95E-16	postive
TET3	AC010761.	0.417378	9.35E-19	postive
NSUN6	AL355488.	0.366903	1.53E-14	postive
NSUN7	AL355488.	0.409609	4.65E-18	postive
DNMT3A	AL355488.	0.318272	3.97E-11	postive
RAD52	AL355488.	0.511646	8.47E-29	postive
NEIL1	AL355488.	0.495529	7.47E-27	postive
TET1	AL355488.	0.361467	3.94E-14	postive
TET2	AL355488.	0.362486	3.30E-14	postive
NSUN3	AC107027.	0.318997	3.56E-11	postive
TDG	AC107027.	0.300808	4.83E-10	postive
ZBTB33	AC107027.	0.338952	1.66E-12	postive
TET1	AC107027.	0.410849	3.61E-18	postive
TET2	AC107027.	0.386101	4.64E-16	postive
TET3	AC107027.	0.348374	3.59E-13	postive
NSUN5	ARRDC1-A	0.365576	1.93E-14	postive
MBD3	ARRDC1-A	0.390612	1.97E-16	postive
NEIL1	AC124319.	0.3181	4.07E-11	postive
TET2	AC124319.	0.392981	1.25E-16	postive
NSUN7	AP005136.	0.311507	1.07E-10	postive
TET1	AP005136.	0.327982	9.20E-12	postive
TET3	AP005136.	0.316107	5.46E-11	postive
NSUN3	AC004832.	0.578606	4.24E-38	postive
TRDMT1	AC004832.	0.396655	6.14E-17	postive
UHRF2	AC004832.	0.329778	6.98E-12	postive
TET1	AC004832.	0.657578	3.00E-52	postive
TET2	AC004832.	0.499388	2.61E-27	postive
MBD3	CHKB-DT	0.330995	5.78E-12	postive
NTHL1	CHKB-DT	0.399092	3.81E-17	postive
SMUG1	CHKB-DT	0.367062	1.48E-14	postive
NSUN6	AL121992.	0.363969	2.55E-14	postive
NEIL1	AL121992.	0.379745	1.51E-15	postive
NSUN7	PXN-AS1	0.308311	1.68E-10	postive
ALYREF	PXN-AS1	0.307161	1.98E-10	postive
MBD3	PXN-AS1	0.315578	5.90E-11	postive
NTHL1	PXN-AS1	0.321741	2.37E-11	postive
SMUG1	PXN-AS1	0.32467	1.52E-11	postive
NTHL1	AC083880.	0.313036	8.54E-11	postive
NSUN3	AF230666.	0.355679	1.06E-13	postive
NSUN6	AF230666.	0.353344	1.57E-13	postive
NSUN7	AF230666.	0.314846	6.56E-11	postive
RAD52	AF230666.	0.314951	6.46E-11	postive
TET1	AF230666.	0.591872	3.31E-40	postive
TET2	AF230666.	0.50913	1.73E-28	postive
TET3	AF230666.	0.348407	3.57E-13	postive
MBD3	LINC02166	0.38971	2.34E-16	postive
DNMT3A	AC022211.	0.353173	1.62E-13	postive
RAD52	AC022211.	0.321857	2.33E-11	postive
TDG	AC022211.	0.311375	1.09E-10	postive
TET2	AC022211.	0.382045	9.89E-16	postive
TET3	AC022211.	0.427248	1.15E-19	postive
NSUN3	GRK5-IT1	0.707913	9.58E-64	postive
TRDMT1	GRK5-IT1	0.457546	1.17E-22	postive
UHRF2	GRK5-IT1	0.349166	3.15E-13	postive
ZBTB38	GRK5-IT1	0.316049	5.50E-11	postive
TET1	GRK5-IT1	0.592406	2.71E-40	postive
TET2	GRK5-IT1	0.531045	2.78E-31	postive

NSUN6	AGBL5-IT1	0.36097	4.29E-14	postive
RAD52	AGBL5-IT1	0.325961	1.25E-11	postive
NEIL1	AGBL5-IT1	0.415817	1.29E-18	postive
NSUN6	AL133410.	0.44956	7.71E-22	postive
NSUN7	AL133410.	0.372503	5.64E-15	postive
RAD52	AL133410.	0.357396	7.92E-14	postive
NEIL1	AL133410.	0.627237	2.49E-46	postive
DNMT3A	AL691432.	0.396201	6.71E-17	postive
NSUN5	ZSCAN16-	0.455641	1.85E-22	postive
ALYREF	ZSCAN16-	0.310648	1.21E-10	postive
MBD3	ZSCAN16-	0.521303	5.15E-30	postive
NTHL1	ZSCAN16-	0.516238	2.26E-29	postive
TET3	ZSCAN16-	-0.31546	6.00E-11	negative
NSUN6	AP001412.	0.422389	3.25E-19	postive
NSUN7	AP001412.	0.323472	1.83E-11	postive
RAD52	AP001412.	0.32705	1.06E-11	postive
NEIL1	AP001412.	0.491156	2.42E-26	postive
NEIL1	PDC-AS1	0.442057	4.32E-21	postive
TET2	PDC-AS1	0.367739	1.32E-14	postive
MBD3	AC006538.	0.331124	5.67E-12	postive
NSUN6	AL138921.	0.362996	3.02E-14	postive
NSUN7	AL138921.	0.336133	2.59E-12	postive
RAD52	AL138921.	0.450801	5.77E-22	postive
NEIL1	AL138921.	0.351794	2.04E-13	postive
TET1	AL138921.	0.518432	1.19E-29	postive
TET2	AL138921.	0.380682	1.27E-15	postive
TET3	AL138921.	0.321875	2.32E-11	postive
NSUN3	AC008443.	0.537648	3.65E-32	postive
DNMT3A	AC008443.	0.365166	2.07E-14	postive
TET3	AC008443.	0.314813	6.60E-11	postive
DNMT3A	AC025165.	0.328653	8.30E-12	postive
DNMT3A	AC046134.	0.302402	3.87E-10	postive
TET3	AC046134.	0.365214	2.05E-14	postive
NSUN3	AC087301.	0.413177	2.24E-18	postive
NSUN7	AC087301.	0.338776	1.70E-12	postive
DNMT3A	AC087301.	0.32523	1.40E-11	postive
TRDMT1	AC087301.	0.308479	1.64E-10	postive
RAD52	AC087301.	0.371561	6.68E-15	postive
MECP2	AC087301.	0.309152	1.49E-10	postive
NEIL1	AC087301.	0.337205	2.19E-12	postive
ZBTB33	AC087301.	0.318652	3.75E-11	postive
TET1	AC087301.	0.482264	2.50E-25	postive
TET2	AC087301.	0.573024	3.06E-37	postive
TET3	AC087301.	0.387925	3.29E-16	postive
DNMT3A	AL358075.	0.326456	1.16E-11	postive
NSUN6	AC092296.	0.315314	6.13E-11	postive
NSUN7	AC092296.	0.370472	8.11E-15	postive
NEIL1	AC092296.	0.339389	1.54E-12	postive
TET1	AC092296.	0.364769	2.22E-14	postive
NSUN6	AC008735.	0.430721	5.40E-20	postive
DNMT3A	AC008735.	0.325618	1.32E-11	postive
RAD52	AC008735.	0.503974	7.36E-28	postive
MBD1	AC008735.	0.375555	3.25E-15	postive
MECP2	AC008735.	0.313681	7.78E-11	postive
NEIL1	AC008735.	0.454035	2.70E-22	postive
TET2	AC008735.	0.40051	2.88E-17	postive
TET3	AC008735.	0.372155	6.01E-15	postive
DNMT3B	AL133520.	0.300563	5.00E-10	postive

NSUN7	AC005225.	0.316777	4.95E-11	postive
TET1	AC005225.	0.573493	2.59E-37	postive
DNMT3A	AL662797.	0.392627	1.34E-16	postive
NSUN7	AP001178.	0.350378	2.58E-13	postive
TET1	AP001178.	0.618304	1.04E-44	postive
TET2	AP001178.	0.401539	2.35E-17	postive
TET3	AP001178.	0.364018	2.53E-14	postive
TET1	AC004477.	0.545149	3.43E-33	postive
NSUN3	FBXO30-D	0.392322	1.42E-16	postive
NSUN7	FBXO30-D	0.353573	1.51E-13	postive
TRDMT1	FBXO30-D	0.350303	2.61E-13	postive
MECP2	FBXO30-D	0.383368	7.73E-16	postive
ZBTB38	FBXO30-D	0.341721	1.06E-12	postive
TET1	FBXO30-D	0.510446	1.19E-28	postive
TET2	FBXO30-D	0.469003	7.22E-24	postive
ZBTB38	AL357033.	0.306286	2.25E-10	postive
TET3	AC124283.	0.323011	1.96E-11	postive
NSUN3	AC005899.	0.343431	8.06E-13	postive
NSUN6	AC005899.	0.330692	6.06E-12	postive
NEIL1	AC005899.	0.308515	1.64E-10	postive
TET1	AC005899.	0.517857	1.41E-29	postive
TET2	AC005899.	0.319307	3.40E-11	postive
NSUN6	AC124312.	0.359745	5.30E-14	postive
NSUN7	AC124312.	0.305724	2.43E-10	postive
TET1	AC124312.	0.610563	2.40E-43	postive
TET2	AC124312.	0.401379	2.43E-17	postive
TET3	AC124312.	0.404631	1.27E-17	postive
NSUN6	AC024267.	0.467456	1.06E-23	postive
NSUN7	AC024267.	0.335344	2.93E-12	postive
RAD52	AC024267.	0.395686	7.42E-17	postive
NEIL1	AC024267.	0.348336	3.61E-13	postive
ZBTB33	AC024267.	0.328063	9.09E-12	postive
TET1	AC024267.	0.468294	8.60E-24	postive
TET2	AC024267.	0.499896	2.27E-27	postive
TET3	AC024267.	0.454078	2.67E-22	postive
NSUN7	AL136295.	0.310121	1.30E-10	postive
NEIL1	AL136295.	0.344291	7.01E-13	postive
DNMT3A	NIFK-AS1	0.341499	1.10E-12	postive
MBD3	AC004449.	0.318232	3.99E-11	postive
NSUN3	AC129510.	0.388404	3.00E-16	postive
NSUN6	AC129510.	0.358163	6.95E-14	postive
NSUN7	AC129510.	0.396564	6.25E-17	postive
DNMT3A	AC129510.	0.337181	2.19E-12	postive
RAD52	AC129510.	0.437526	1.20E-20	postive
MECP2	AC129510.	0.358892	6.13E-14	postive
NEIL1	AC129510.	0.384861	5.85E-16	postive
TET1	AC129510.	0.458207	1.00E-22	postive
TET2	AC129510.	0.473888	2.13E-24	postive
TET3	AC129510.	0.35675	8.84E-14	postive
NSUN3	MAP3K20-	0.648626	1.97E-50	postive
TRDMT1	MAP3K20-	0.420456	4.90E-19	postive
TET1	MAP3K20-	0.416999	1.01E-18	postive
TET2	MAP3K20-	0.329403	7.40E-12	postive
NSUN7	AL359644.	0.304929	2.72E-10	postive
TET1	AL359644.	0.642216	3.61E-49	postive
TET3	AL359644.	0.353555	1.52E-13	postive
NSUN3	AC009704.	0.418295	7.72E-19	postive
NSUN7	AC009704.	0.314983	6.43E-11	postive

NEIL1	AC009704.	0.306749	2.10E-10	postive
UHRF2	AC009704.	0.303341	3.40E-10	postive
TET1	AC009704.	0.586726	2.23E-39	postive
TET2	AC009704.	0.553741	2.13E-34	postive
TET3	AC009704.	0.345444	5.81E-13	postive
NSUN3	AC079921.	0.682338	1.29E-57	postive
TRDMT1	AC079921.	0.508906	1.84E-28	postive
ZBTB38	AC079921.	0.351342	2.20E-13	postive
TET1	AC079921.	0.303432	3.35E-10	postive
TET2	AL596325.	0.502312	1.17E-27	postive
NSUN3	Z82243.1	0.610978	2.03E-43	postive
TRDMT1	Z82243.1	0.373254	4.93E-15	postive
UHRF2	Z82243.1	0.34461	6.65E-13	postive
ZBTB38	Z82243.1	0.318676	3.74E-11	postive
TET1	Z82243.1	0.573752	2.37E-37	postive
TET2	Z82243.1	0.51548	2.81E-29	postive
NSUN3	MAP3K5-A	0.335095	3.05E-12	postive
NSUN6	MAP3K5-A	0.321129	2.60E-11	postive
TET1	MAP3K5-A	0.608477	5.50E-43	postive
TET2	MAP3K5-A	0.435743	1.78E-20	postive
TET1	AP001189.	0.305539	2.49E-10	postive
TET2	AC018695.	0.456844	1.39E-22	postive
NSUN6	AC011477.	0.430465	5.71E-20	postive
NSUN7	AC011477.	0.410971	3.52E-18	postive
DNMT3A	AC011477.	0.452628	3.76E-22	postive
MBD1	AC011477.	0.383008	8.27E-16	postive
NEIL1	AC011477.	0.312661	9.02E-11	postive
TDG	AC011477.	0.372621	5.52E-15	postive
ZBTB33	AC011477.	0.436095	1.65E-20	postive
TET2	AC011477.	0.379509	1.58E-15	postive
TET3	AC011477.	0.583185	8.14E-39	postive
TET2	AC067817.	0.34181	1.05E-12	postive
NSUN6	AC107068.	0.459968	6.57E-23	postive
NSUN7	AC107068.	0.383254	7.90E-16	postive
DNMT3A	AC107068.	0.377625	2.23E-15	postive
DNMT3B	AC107068.	0.324616	1.54E-11	postive
RAD52	AC107068.	0.379322	1.64E-15	postive
NEIL1	AC107068.	0.33096	5.81E-12	postive
TDG	AC107068.	0.426461	1.36E-19	postive
ZBTB33	AC107068.	0.38859	2.90E-16	postive
TET1	AC107068.	0.351815	2.03E-13	postive
TET2	AC107068.	0.404965	1.19E-17	postive
TET3	AC107068.	0.49542	7.69E-27	postive
NSUN3	N4BP2L2-I	0.356997	8.47E-14	postive
NSUN6	N4BP2L2-I	0.383292	7.84E-16	postive
NSUN7	N4BP2L2-I	0.356205	9.69E-14	postive
RAD52	N4BP2L2-I	0.316526	5.13E-11	postive
TET1	N4BP2L2-I	0.660465	7.54E-53	postive
TET2	N4BP2L2-I	0.481035	3.44E-25	postive
TET3	N4BP2L2-I	0.370102	8.67E-15	postive
ZBTB33	AL355472.	0.337041	2.24E-12	postive
NSUN3	EIPR1-IT1	0.457631	1.15E-22	postive
DNMT3A	EIPR1-IT1	0.501053	1.65E-27	postive
TRDMT1	EIPR1-IT1	0.364286	2.42E-14	postive
UHRF2	EIPR1-IT1	0.307483	1.89E-10	postive
ZBTB33	EIPR1-IT1	0.317671	4.34E-11	postive
ZBTB38	EIPR1-IT1	0.304776	2.78E-10	postive
TET1	EIPR1-IT1	0.484527	1.39E-25	postive

TET2	EIPR1-IT1	0.446138	1.70E-21	postive
TET3	EIPR1-IT1	0.421759	3.72E-19	postive
RAD52	AC123768.	0.30431	2.97E-10	postive
NSUN3	AP001469.	0.32675	1.11E-11	postive
UHRF2	AP001469.	0.332996	4.24E-12	postive
TET1	AP001469.	0.464449	2.21E-23	postive
TET2	AP001469.	0.609958	3.05E-43	postive
TET3	AP001469.	0.320392	2.90E-11	postive
NSUN6	AC012186.	0.493819	1.18E-26	postive
RAD52	AC012186.	0.370708	7.78E-15	postive
NEIL1	AC012186.	0.462407	3.64E-23	postive
TET2	AC012186.	0.32771	9.59E-12	postive
ZBTB38	LINC0268ε	0.353868	1.44E-13	postive
DNMT3B	LINC01671	0.301219	4.56E-10	postive
RAD52	AC009090.	0.312688	8.98E-11	postive
NEIL1	AC009090.	0.334122	3.55E-12	postive
TET2	AC009090.	0.366971	1.51E-14	postive
NSUN3	LINC0177ξ	0.407226	7.54E-18	postive
DNMT1	LINC0177ξ	0.448345	1.02E-21	postive
TRDMT1	LINC0177ξ	0.309921	1.34E-10	postive
UHRF1	LINC0177ξ	0.393476	1.14E-16	postive
RAD52	AC079684.	0.311422	1.08E-10	postive
TET1	AC079684.	0.363444	2.80E-14	postive
TET3	AC079684.	0.303508	3.32E-10	postive
NSUN3	LINC0035ξ	0.325297	1.39E-11	postive
TET1	LINC0035ξ	0.423801	2.41E-19	postive
NSUN3	AC009121.	0.307457	1.90E-10	postive
NSUN7	AC009121.	0.408992	5.27E-18	postive
DNMT1	AC009121.	0.302316	3.92E-10	postive
DNMT3A	AC009121.	0.357516	7.76E-14	postive
RAD52	AC009121.	0.378352	1.95E-15	postive
MBD1	AC009121.	0.300025	5.38E-10	postive
UHRF2	AC009121.	0.356088	9.89E-14	postive
ZBTB33	AC009121.	0.365098	2.10E-14	postive
TET1	AC009121.	0.517153	1.73E-29	postive
TET2	AC009121.	0.380569	1.30E-15	postive
TET3	AC009121.	0.447073	1.37E-21	postive
NSUN6	UBE2Q1-A	0.38813	3.16E-16	postive
NSUN7	UBE2Q1-A	0.326512	1.15E-11	postive
RAD52	UBE2Q1-A	0.388182	3.13E-16	postive
NEIL1	UBE2Q1-A	0.394402	9.51E-17	postive
ZBTB33	UBE2Q1-A	0.37847	1.91E-15	postive
TET1	UBE2Q1-A	0.351264	2.22E-13	postive
TET2	UBE2Q1-A	0.550835	5.50E-34	postive
TET3	UBE2Q1-A	0.426728	1.28E-19	postive
NSUN6	AC004263.	0.41421	1.81E-18	postive
NSUN7	AC004263.	0.313652	7.81E-11	postive
RAD52	AC004263.	0.398763	4.07E-17	postive
TET1	AC004263.	0.479254	5.44E-25	postive
TET2	AC004263.	0.33714	2.21E-12	postive
TET3	AC004263.	0.319694	3.21E-11	postive
NSUN6	AC011479.	0.370873	7.55E-15	postive
DNMT3A	AC011479.	0.341672	1.07E-12	postive
RAD52	AC011479.	0.389751	2.32E-16	postive
NEIL1	AC011479.	0.302262	3.95E-10	postive
ZBTB33	AC011479.	0.355416	1.11E-13	postive
TET1	AC011479.	0.340981	1.20E-12	postive
TET2	AC011479.	0.476752	1.03E-24	postive

TET3	AC011479.	0.442555	3.86E-21	postive
DNMT1	CDKN2A-[	0.302128	4.02E-10	postive
UHRF1	CDKN2A-[	0.332386	4.66E-12	postive
DNMT3A	RAMP2-AS	0.396767	6.01E-17	postive
YBX1	RAMP2-AS	0.401163	2.53E-17	postive
TET1	HID1-AS1	0.430115	6.16E-20	postive
NSUN7	AC018521.	0.351191	2.25E-13	postive
TET1	AC018521.	0.60586	1.55E-42	postive
TET2	AC018521.	0.358247	6.85E-14	postive
TET3	AC018521.	0.412085	2.80E-18	postive
NSUN6	AL354696.	0.329347	7.46E-12	postive
NEIL1	AL354696.	0.39777	4.94E-17	postive
NSUN6	BX284668.	0.387158	3.80E-16	postive
NSUN7	BX284668.	0.325742	1.29E-11	postive
NEIL1	BX284668.	0.354362	1.32E-13	postive
TET1	BX284668.	0.338674	1.73E-12	postive
NSUN6	AP000866.	0.39722	5.50E-17	postive
NSUN7	AP000866.	0.410803	3.64E-18	postive
NEIL1	AP000866.	0.347514	4.14E-13	postive
SMUG1	AP000866.	0.333214	4.10E-12	postive
NSUN7	AL512408.	0.312362	9.42E-11	postive
NSUN6	AC011468.	0.36592	1.81E-14	postive
RAD52	AC011468.	0.328157	8.96E-12	postive
NEIL1	AC011468.	0.334492	3.35E-12	postive
NEIL1	AC019257.	0.328631	8.33E-12	postive
NSUN3	AL031670.	0.476831	1.01E-24	postive
TRDMT1	AL031670.	0.404787	1.23E-17	postive
RAD52	AL031670.	0.336282	2.53E-12	postive
MECP2	AL031670.	0.351541	2.12E-13	postive
UHRF2	AL031670.	0.421207	4.18E-19	postive
ZBTB33	AL031670.	0.342484	9.39E-13	postive
ZBTB38	AL031670.	0.329544	7.24E-12	postive
TET1	AL031670.	0.519658	8.35E-30	postive
TET2	AL031670.	0.582132	1.19E-38	postive
TET3	AL031670.	0.355898	1.02E-13	postive
NSUN6	AC026367.	0.424432	2.10E-19	postive
NSUN7	AC026367.	0.302062	4.06E-10	postive
NEIL1	AC026367.	0.307382	1.92E-10	postive
SMUG1	AC026367.	0.318073	4.09E-11	postive
NSUN3	MCM3AP-	0.342744	9.01E-13	postive
NSUN6	MCM3AP-	0.310089	1.31E-10	postive
NSUN7	MCM3AP-	0.352954	1.68E-13	postive
RAD52	MCM3AP-	0.339796	1.45E-12	postive
NEIL1	MCM3AP-	0.335654	2.79E-12	postive
UHRF2	MCM3AP-	0.340523	1.29E-12	postive
ZBTB33	MCM3AP-	0.357387	7.93E-14	postive
TET1	MCM3AP-	0.53488	8.59E-32	postive
TET2	MCM3AP-	0.651898	4.33E-51	postive
TET3	MCM3AP-	0.459458	7.42E-23	postive
SMUG1	AC005911.	0.446437	1.59E-21	postive
MBD3	MHENCR	0.32387	1.72E-11	postive
NEIL1	MHENCR	0.458482	9.38E-23	postive
NTHL1	MHENCR	0.371693	6.52E-15	postive
ZBTB38	MHENCR	-0.35475	1.24E-13	negative
NSUN6	PCAT1	0.314366	7.04E-11	postive
TET1	PCAT1	0.429844	6.53E-20	postive
DNMT3A	PPP1R26-/	0.322089	2.25E-11	postive
ZBTB33	PPP1R26-/	0.404838	1.22E-17	postive

TET3	PPP1R26-/	0.359865	5.19E-14	postive
NSUN3	FRMD6-AS	0.313805	7.64E-11	postive
TET1	FRMD6-AS	0.395812	7.24E-17	postive
TET2	FRMD6-AS	0.521198	5.31E-30	postive
NSUN5	LINC01315	0.301916	4.14E-10	postive
NSUN7	AL137802.	0.32955	7.23E-12	postive
DNMT3A	AL137802.	0.345044	6.20E-13	postive
NSUN6	AL592211.	0.353868	1.44E-13	postive
RAD52	AL592211.	0.363872	2.60E-14	postive
NEIL1	AL592211.	0.461101	4.99E-23	postive
NSUN3	LINC00954	0.376129	2.93E-15	postive
DNMT3A	LINC00954	0.393051	1.23E-16	postive
RAD52	LINC00954	0.312452	9.30E-11	postive
MBD1	LINC00954	0.345735	5.54E-13	postive
NSUN7	AL132639.	0.322146	2.23E-11	postive
ZBTB4	AC005180.	0.304459	2.90E-10	postive
NSUN3	SH3RF3-A	0.532324	1.88E-31	postive
ZBTB38	SH3RF3-A	0.336812	2.33E-12	postive
NSUN7	AC141002.	0.319632	3.24E-11	postive
NEIL1	AC141002.	0.377302	2.37E-15	postive
TET1	AC141002.	0.382311	9.41E-16	postive
TET2	AC141002.	0.314809	6.60E-11	postive
NSUN6	EHMT2-AS	0.415711	1.32E-18	postive
NSUN7	EHMT2-AS	0.454008	2.72E-22	postive
RAD52	EHMT2-AS	0.356981	8.50E-14	postive
NEIL1	EHMT2-AS	0.445979	1.76E-21	postive
DNMT3A	AC090517.	0.306123	2.30E-10	postive
RAD52	AC090517.	0.32471	1.51E-11	postive
SMUG1	AC090517.	0.358769	6.26E-14	postive
TET1	AC090517.	0.419502	5.99E-19	postive
TET2	AC090517.	0.30228	3.94E-10	postive
NSUN5	LINC02413	0.314163	7.25E-11	postive
NTHL1	LINC02413	0.404012	1.44E-17	postive
MBD3	MYG1-AS1	0.313727	7.73E-11	postive
NSUN6	AC010655.	0.343844	7.54E-13	postive
NSUN7	AL445231.	0.369566	9.53E-15	postive
TET1	AL445231.	0.379654	1.54E-15	postive
TET3	AL445231.	0.326828	1.10E-11	postive
DNMT3A	ZNF528-A	0.317544	4.42E-11	postive
NSUN6	AC040162.	0.361311	4.05E-14	postive
RAD52	AC040162.	0.359493	5.53E-14	postive
NEIL1	AC040162.	0.484551	1.38E-25	postive
NEIL1	AC012645.	0.355408	1.11E-13	postive
NSUN6	AC073575.	0.384218	6.60E-16	postive
NSUN7	AC073575.	0.324525	1.56E-11	postive
RAD52	AC073575.	0.483261	1.93E-25	postive
NEIL1	AC073575.	0.37655	2.71E-15	postive
TET3	AC073575.	0.306833	2.08E-10	postive
NSUN6	AL390294.	0.574636	1.73E-37	postive
NEIL1	AL390294.	0.416003	1.25E-18	postive
TET2	FRMD6-AS	0.438712	9.19E-21	postive
TET3	AC114488.	0.323918	1.71E-11	postive
TRDMT1	AC027097.	0.323397	1.85E-11	postive
MECP2	AC027097.	0.314519	6.88E-11	postive
UHRF2	AC027097.	0.351431	2.16E-13	postive
TET1	AC027097.	0.498494	3.33E-27	postive
NSUN6	AC008687.	0.318808	3.67E-11	postive
RAD52	AC008687.	0.336623	2.40E-12	postive

TET1	AC008687.	0.308426	1.66E-10	postive
TET2	AC008687.	0.376783	2.60E-15	postive
TET1	AL365436.	0.484954	1.24E-25	postive
TET2	AL365436.	0.57344	2.64E-37	postive
TET3	AL365436.	0.360593	4.58E-14	postive
NEIL1	MIR210HG	0.317398	4.51E-11	postive
NSUN3	AP001001.	0.465957	1.53E-23	postive
NSUN6	AP001001.	0.358092	7.03E-14	postive
NSUN7	AP001001.	0.30255	3.79E-10	postive
RAD52	AP001001.	0.3362	2.56E-12	postive
TET1	AP001001.	0.567023	2.45E-36	postive
TET2	AP001001.	0.597444	4.03E-41	postive
TET3	AP001001.	0.3637	2.67E-14	postive
NSUN6	AC063948.	0.35496	1.20E-13	postive
RAD52	AC063948.	0.323291	1.88E-11	postive
NEIL1	AC063948.	0.402952	1.78E-17	postive
SMUG1	AC063948.	0.311192	1.12E-10	postive
TET2	AL031848.	0.300107	5.32E-10	postive
NSUN5	AC008608.	0.3359	2.69E-12	postive
MBD3	AC008608.	0.359476	5.55E-14	postive
NTHL1	AC008608.	0.421707	3.76E-19	postive
SMUG1	AC008608.	0.331294	5.52E-12	postive
NSUN3	AC069277.	0.650731	7.45E-51	postive
TRDMT1	AC069277.	0.413616	2.04E-18	postive
NSUN3	AC004832.	0.473408	2.40E-24	postive
TRDMT1	AC004832.	0.393985	1.03E-16	postive
UHRF2	AC004832.	0.329474	7.32E-12	postive
TET1	AC004832.	0.659148	1.42E-52	postive
TET2	AC004832.	0.419456	6.05E-19	postive
SMUG1	DDX39B-A	0.341716	1.06E-12	postive
NSUN6	AC253576.	0.420298	5.06E-19	postive
NSUN7	AC253576.	0.406455	8.80E-18	postive
NEIL1	AC253576.	0.326549	1.15E-11	postive
TET1	AC253576.	0.510067	1.33E-28	postive
TET2	AC253576.	0.425847	1.55E-19	postive
TET3	AC253576.	0.31664	5.05E-11	postive
TET2	LINC00491	0.368066	1.24E-14	postive
TET1	AC011472.	0.434296	2.46E-20	postive
ZBTB38	LINC01058	0.326152	1.22E-11	postive
NSUN2	AC092171.	0.327292	1.02E-11	postive
NSUN5	AC092171.	0.332521	4.56E-12	postive
DNMT1	AC092171.	0.358307	6.78E-14	postive
MBD3	AC092171.	0.30934	1.45E-10	postive
MBD3	AC124798.	0.335449	2.88E-12	postive
NEIL1	AL162431.	0.379052	1.72E-15	postive
NSUN3	AL442125.	0.437167	1.30E-20	postive
DNMT1	AL442125.	0.302508	3.82E-10	postive
TRDMT1	AL442125.	0.472665	2.90E-24	postive
UHRF2	AL442125.	0.424132	2.24E-19	postive
ZBTB38	AL442125.	0.31429	7.12E-11	postive
TET1	AL442125.	0.464325	2.28E-23	postive
TET2	AL442125.	0.423969	2.32E-19	postive
NSUN3	AC009716.	0.670359	5.92E-55	postive
TRDMT1	AC009716.	0.438263	1.02E-20	postive
UHRF2	AC009716.	0.35364	1.49E-13	postive
ZBTB38	AC009716.	0.32414	1.65E-11	postive
TET1	AC009716.	0.492047	1.91E-26	postive
TET2	AC009716.	0.552895	2.81E-34	postive

NSUN7	AL356481.	0.364502	2.33E-14	postive
TET1	AL356481.	0.384895	5.81E-16	postive
NEIL1	HOXA-AS:	0.390059	2.19E-16	postive
TET2	HOXA-AS:	0.490503	2.88E-26	postive
NSUN2	AC012640.	0.428257	9.23E-20	postive
NSUN5	AC012640.	0.30144	4.43E-10	postive
DNMT3A	AC012640.	0.347677	4.03E-13	postive
DNMT1	AC244153.	-0.33214	4.84E-12	negative
SMUG1	AC244153.	0.35483	1.22E-13	postive
NSUN6	AC005537.	0.388671	2.85E-16	postive
NSUN7	AC005537.	0.318137	4.05E-11	postive
DNMT3A	AC005537.	0.325542	1.34E-11	postive
TET1	AC005537.	0.313889	7.55E-11	postive
TET3	AC005537.	0.361108	4.19E-14	postive
NSUN6	PHKA2-AS	0.312884	8.73E-11	postive
NSUN7	WDFY3-AS	0.351366	2.19E-13	postive
TET1	WDFY3-AS	0.314077	7.34E-11	postive
TET2	WDFY3-AS	0.312621	9.07E-11	postive
NSUN3	AC105036.	0.318213	4.00E-11	postive
TET1	AC105036.	0.504711	5.99E-28	postive
TET2	AC105036.	0.568054	1.72E-36	postive
TET3	AC105036.	0.311223	1.11E-10	postive
NTHL1	MNX1-AS:	0.316599	5.08E-11	postive
NSUN6	AL139286.	0.307646	1.85E-10	postive
NSUN7	AL139286.	0.313434	8.06E-11	postive
DNMT3A	AL139286.	0.399723	3.37E-17	postive
RAD52	AL139286.	0.370247	8.44E-15	postive
NEIL1	AL139286.	0.330837	5.93E-12	postive
TET1	AL139286.	0.310424	1.25E-10	postive
MBD3	AC138696.	0.36424	2.43E-14	postive
NTHL1	AC138696.	0.339778	1.45E-12	postive
NSUN6	AC018809.	0.33647	2.46E-12	postive
RAD52	AC018809.	0.425235	1.77E-19	postive
NEIL1	AC018809.	0.565864	3.65E-36	postive
DNMT3A	CR786580.	0.34766	4.04E-13	postive
NSUN6	VIPR1-AS1	0.433638	2.84E-20	postive
RAD52	VIPR1-AS1	0.353077	1.64E-13	postive
MBD1	VIPR1-AS1	0.321331	2.52E-11	postive
NEIL1	VIPR1-AS1	0.427998	9.76E-20	postive
TET2	VIPR1-AS1	0.484359	1.45E-25	postive
TET3	VIPR1-AS1	0.362475	3.31E-14	postive
NSUN6	AL592430.	0.390085	2.18E-16	postive
NSUN7	AL592430.	0.336008	2.64E-12	postive
TET1	AL592430.	0.660425	7.69E-53	postive
TET2	AL592430.	0.459907	6.66E-23	postive
TET3	AL592430.	0.361242	4.10E-14	postive
RAD52	LINC02367	0.344616	6.65E-13	postive
NSUN3	AC092802.	0.37791	2.12E-15	postive
NSUN6	AC092802.	0.417265	9.57E-19	postive
NSUN7	AC092802.	0.37698	2.51E-15	postive
RAD52	AC092802.	0.330175	6.57E-12	postive
MECP2	AC092802.	0.35804	7.09E-14	postive
ZBTB33	AC092802.	0.371063	7.30E-15	postive
TET1	AC092802.	0.644952	1.05E-49	postive
TET2	AC092802.	0.555592	1.16E-34	postive
TET3	AC092802.	0.441697	4.69E-21	postive
NSUN6	AC021491.	0.45881	8.67E-23	postive
NSUN7	AC021491.	0.362715	3.17E-14	postive

RAD52	AC021491.	0.333946	3.65E-12	postive
TET1	AC021491.	0.525127	1.66E-30	postive
TET2	AC021491.	0.40603	9.59E-18	postive
TET3	AC021491.	0.438491	9.66E-21	postive
RAD52	AP000569.	0.315533	5.94E-11	postive
NEIL1	AP000569.	0.356184	9.73E-14	postive
SMUG1	ATXN2-AS	0.485525	1.07E-25	postive
NSUN3	AL133371.	0.5139	4.44E-29	postive
TRDMT1	AL133371.	0.315209	6.22E-11	postive
NSUN6	AL158196.	0.33066	6.09E-12	postive
NEIL1	AL158196.	0.353215	1.61E-13	postive
NSUN6	AP001625.	0.414659	1.65E-18	postive
NSUN7	AP001625.	0.377705	2.20E-15	postive
RAD52	AP001625.	0.330555	6.19E-12	postive
NEIL1	AP001625.	0.336398	2.48E-12	postive
TET1	AP001625.	0.471552	3.83E-24	postive
TET2	AP001625.	0.525635	1.42E-30	postive
TET3	AP001625.	0.490153	3.16E-26	postive
MBD3	AC069281.	0.372128	6.03E-15	postive
NEIL1	AC069281.	0.428971	7.90E-20	postive
NSUN6	AC132872.	0.338783	1.70E-12	postive
RAD52	AC132872.	0.446276	1.65E-21	postive
NEIL1	AC132872.	0.429385	7.22E-20	postive
NSUN6	AC012615.	0.35183	2.02E-13	postive
NSUN7	AC012615.	0.314629	6.78E-11	postive
DNMT3A	AC012615.	0.34453	6.74E-13	postive
NEIL1	AC012615.	0.448778	9.24E-22	postive
MBD3	AP003419.	0.345421	5.83E-13	postive
NEIL1	AP003419.	0.483048	2.04E-25	postive
DNMT1	CDKN2B-A	0.446354	1.62E-21	postive
MBD4	CDKN2B-A	0.404964	1.19E-17	postive
UHRF1	CDKN2B-A	0.413842	1.95E-18	postive
UHRF2	CDKN2B-A	0.375575	3.24E-15	postive
NSUN7	AF129075.	0.318675	3.74E-11	postive
TET1	AF129075.	0.301888	4.16E-10	postive
NSUN3	AC092301.	0.542283	8.53E-33	postive
NSUN6	AC092301.	0.38729	3.71E-16	postive
NSUN7	AC092301.	0.306777	2.09E-10	postive
DNMT3A	AC092301.	0.362876	3.09E-14	postive
RAD52	AC092301.	0.449778	7.33E-22	postive
MBD1	AC092301.	0.335617	2.81E-12	postive
MECP2	AC092301.	0.312547	9.17E-11	postive
NEIL1	AC092301.	0.355009	1.19E-13	postive
TET1	AC092301.	0.435402	1.92E-20	postive
TET2	AC092301.	0.54717	1.80E-33	postive
TET3	AC092301.	0.439785	7.22E-21	postive
NSUN3	AC024230.	0.346202	5.13E-13	postive
NSUN7	AC018647.	0.337523	2.08E-12	postive
ZBTB33	AC018647.	0.310627	1.21E-10	postive
TET3	AC018647.	0.433035	3.25E-20	postive
TET1	AC105001.	0.302011	4.09E-10	postive
TET2	AC105001.	0.393096	1.22E-16	postive
NSUN6	AC092123.	0.302594	3.77E-10	postive
NSUN7	AC092123.	0.310968	1.15E-10	postive
RAD52	AC092123.	0.325721	1.30E-11	postive
NEIL1	AC092123.	0.305785	2.41E-10	postive
NSUN3	AL133445.	0.674095	9.04E-56	postive
TRDMT1	AL133445.	0.465518	1.70E-23	postive

UHRF2	AL133445.	0.339856	1.43E-12	postive
ZBTB38	AL133445.	0.30294	3.59E-10	postive
TET1	AL133445.	0.568336	1.56E-36	postive
TET2	AL133445.	0.49129	2.33E-26	postive
NSUN3	AC019118.	0.310563	1.22E-10	postive
NSUN6	AC019118.	0.323183	1.91E-11	postive
RAD52	AC019118.	0.411647	3.06E-18	postive
TET1	AC019118.	0.372545	5.60E-15	postive
NTHL1	AL445524.	0.352021	1.96E-13	postive
NSUN5	AC027319.	0.312882	8.74E-11	postive
MBD3	AC027319.	0.372907	5.25E-15	postive
NTHL1	AC027319.	0.379203	1.67E-15	postive
NSUN6	AL133551.	0.326626	1.13E-11	postive
DNMT3A	AL133551.	0.307979	1.77E-10	postive
NTHL1	AL133551.	0.330575	6.17E-12	postive
SMUG1	AL133551.	0.316522	5.14E-11	postive
NTHL1	LINC01456	0.31033	1.26E-10	postive
DNMT3A	AC007663.	0.303562	3.29E-10	postive
NSUN7	AC005229.	0.340607	1.27E-12	postive
NSUN3	AL161719.	0.559989	2.67E-35	postive
TRDMT1	AL161719.	0.346681	4.74E-13	postive
NSUN3	RASA3-IT1	0.647955	2.68E-50	postive
TRDMT1	RASA3-IT1	0.408025	6.41E-18	postive
UHRF2	RASA3-IT1	0.311753	1.03E-10	postive
TET1	RASA3-IT1	0.354425	1.31E-13	postive
TET2	RASA3-IT1	0.443147	3.37E-21	postive
RAD52	AC107081.	0.309818	1.36E-10	postive
NEIL1	AC107081.	0.380107	1.42E-15	postive
TET1	AC107081.	0.372111	6.05E-15	postive
TET2	AC107081.	0.435103	2.06E-20	postive
NSUN7	LINC01535	0.353836	1.45E-13	postive
TET1	LINC01535	0.350769	2.42E-13	postive
TET3	LINC01535	0.303607	3.27E-10	postive
TET1	AC106771.	0.42321	2.73E-19	postive
TET3	AC106771.	0.321513	2.45E-11	postive
NSUN6	AL080317.	0.39292	1.27E-16	postive
NSUN7	AL080317.	0.377579	2.25E-15	postive
RAD52	AL080317.	0.320678	2.78E-11	postive
TET1	AL080317.	0.694231	2.19E-60	postive
TET2	AL080317.	0.40514	1.15E-17	postive
TET3	AL080317.	0.435632	1.83E-20	postive
RAD52	AC010463.	0.341517	1.10E-12	postive
NEIL1	AC010463.	0.456402	1.54E-22	postive
TET1	AC010463.	0.323526	1.81E-11	postive
TET2	AC010463.	0.447097	1.36E-21	postive
NSUN6	AC073534.	0.385747	4.95E-16	postive
NSUN7	AC073534.	0.327335	1.02E-11	postive
TET1	AC073534.	0.583053	8.54E-39	postive
TET2	AL391335.	0.336628	2.39E-12	postive
NSUN7	AC000068.	0.327169	1.04E-11	postive
NEIL1	AC000068.	0.360928	4.32E-14	postive
NSUN6	AC005387.	0.443135	3.38E-21	postive
NSUN7	AC005387.	0.318466	3.86E-11	postive
RAD52	AC005387.	0.416051	1.23E-18	postive
NEIL1	AC005387.	0.551944	3.83E-34	postive
TET1	AC005387.	0.350104	2.70E-13	postive
TET2	AC005387.	0.475771	1.32E-24	postive
NSUN6	AL645940.	0.38012	1.41E-15	postive

NSUN7	AL645940.	0.318229	3.99E-11	postive
NEIL1	AL645940.	0.557907	5.36E-35	postive
NSUN6	LINC00893	0.476294	1.16E-24	postive
RAD52	LINC00893	0.425238	1.77E-19	postive
NEIL1	LINC00893	0.56294	9.88E-36	postive
NSUN3	CARMN	0.41688	1.04E-18	postive
TRDMT1	CARMN	0.319434	3.34E-11	postive
UHRF2	CARMN	0.309567	1.41E-10	postive
TET1	CARMN	0.675542	4.33E-56	postive
TET2	CARMN	0.51808	1.32E-29	postive
TET3	CARMN	0.309075	1.51E-10	postive
NSUN3	AC091982.	0.343266	8.28E-13	postive
MECP2	AC091982.	0.300168	5.28E-10	postive
UHRF2	AC091982.	0.303946	3.12E-10	postive
TET1	AC091982.	0.474525	1.81E-24	postive
TET2	AC091982.	0.512891	5.93E-29	postive
TET3	AC091982.	0.321652	2.40E-11	postive
NSUN3	AP000829.	0.536112	5.87E-32	postive
TRDMT1	AP000829.	0.385	5.70E-16	postive
TET1	AP000829.	0.537101	4.32E-32	postive
NSUN6	AC092279.	0.340758	1.24E-12	postive
NSUN7	AC092279.	0.351702	2.07E-13	postive
TET1	AC092279.	0.710761	1.81E-64	postive
TET2	AC092279.	0.402854	1.81E-17	postive
TET3	AC092279.	0.445593	1.93E-21	postive
NTHL1	AC004080.	0.384247	6.56E-16	postive
NSUN6	TRAPPC12	0.4137	2.01E-18	postive
RAD52	TRAPPC12	0.383229	7.94E-16	postive
MBD1	TRAPPC12	0.367418	1.39E-14	postive
NEIL1	TRAPPC12	0.424874	1.91E-19	postive
TET2	TRAPPC12	0.30643	2.20E-10	postive
NSUN6	MORF4L2-	0.414207	1.81E-18	postive
NSUN7	MORF4L2-	0.472693	2.87E-24	postive
DNMT3A	MORF4L2-	0.371871	6.32E-15	postive
RAD52	MORF4L2-	0.416574	1.11E-18	postive
MBD1	MORF4L2-	0.331152	5.64E-12	postive
MECP2	MORF4L2-	0.316955	4.82E-11	postive
NEIL1	MORF4L2-	0.396084	6.87E-17	postive
ZBTB33	MORF4L2-	0.370785	7.67E-15	postive
TET1	MORF4L2-	0.456747	1.42E-22	postive
TET2	MORF4L2-	0.340426	1.31E-12	postive
TET3	MORF4L2-	0.444374	2.55E-21	postive
NSUN3	AL137779.	0.560817	2.02E-35	postive
TRDMT1	AL137779.	0.412464	2.59E-18	postive
UHRF2	AL137779.	0.3626	3.24E-14	postive
ZBTB38	AL137779.	0.309742	1.37E-10	postive
TET1	AL137779.	0.591527	3.77E-40	postive
TET2	AL137779.	0.53879	2.55E-32	postive
NSUN2	AC022211.	0.333457	3.94E-12	postive
NSUN5	AC108047.	0.334083	3.57E-12	postive
NTHL1	AC108047.	0.356356	9.45E-14	postive
DNMT3A	AC016705.	0.39866	4.15E-17	postive
NSUN7	AC062037.	0.344001	7.35E-13	postive
RAD52	AC062037.	0.31673	4.98E-11	postive
NEIL1	AC062037.	0.353255	1.59E-13	postive
TET2	AC062037.	0.310135	1.30E-10	postive
TET3	AC062037.	0.336673	2.38E-12	postive
NSUN6	AL139041.	0.364384	2.37E-14	postive

TET1	AL139041.	0.520033	7.48E-30	postive
TET2	AL139041.	0.311202	1.11E-10	postive
TET3	AL139041.	0.338847	1.68E-12	postive
NSUN3	AC005021.	0.324046	1.67E-11	postive
UHRF2	AC005021.	0.300381	5.13E-10	postive
TET1	AC005021.	0.579715	2.85E-38	postive
TET2	AC005021.	0.560228	2.47E-35	postive
TET3	AC005021.	0.331716	5.17E-12	postive
NSUN7	AC120114.	0.388923	2.72E-16	postive
DNMT1	AC120114.	0.368808	1.09E-14	postive
DNMT3A	AC120114.	0.333286	4.05E-12	postive
TDG	AC120114.	0.308711	1.59E-10	postive
UHRF1	AC120114.	0.313904	7.53E-11	postive
UNG	AC120114.	0.316569	5.10E-11	postive
ZBTB33	AC120114.	0.368122	1.23E-14	postive
TET1	AC120114.	0.444993	2.21E-21	postive
TET2	AC120114.	0.315169	6.26E-11	postive
TET3	AC120114.	0.314224	7.19E-11	postive
DNMT3A	AC009237.	0.3291	7.75E-12	postive
NSUN3	AC244093.	0.566801	2.65E-36	postive
TRDMT1	AC244093.	0.330822	5.94E-12	postive
UHRF2	AC244093.	0.322964	1.97E-11	postive
ZBTB33	AC244093.	0.312482	9.26E-11	postive
ZBTB38	AC244093.	0.353233	1.60E-13	postive
TET1	AC244093.	0.623294	1.31E-45	postive
TET2	AC244093.	0.622202	2.07E-45	postive
TET3	AC244093.	0.414018	1.88E-18	postive
NSUN7	AL442663.	0.309256	1.47E-10	postive
TET1	AL442663.	0.314137	7.28E-11	postive
NSUN7	AL136531.	0.367617	1.35E-14	postive
DNMT3A	AL136531.	0.323807	1.74E-11	postive
RAD52	AL136531.	0.345694	5.58E-13	postive
TET1	AL136531.	0.536914	4.58E-32	postive
TET2	AL136531.	0.331737	5.15E-12	postive
NSUN6	AC233728.	0.385378	5.31E-16	postive
RAD52	AC233728.	0.3466	4.81E-13	postive
NEIL1	AC233728.	0.41973	5.71E-19	postive
SMUG1	LINC01767	0.418186	7.90E-19	postive
NSUN6	LINC01876	0.368625	1.13E-14	postive
NSUN7	LINC01876	0.342384	9.55E-13	postive
TET1	LINC01876	0.602336	6.13E-42	postive
TET2	LINC01876	0.518621	1.13E-29	postive
TET3	LINC01876	0.442808	3.64E-21	postive
TET3	PINK1-AS	0.376254	2.86E-15	postive
NSUN6	AC109347.	0.312118	9.76E-11	postive
NSUN6	AC232271.	0.430059	6.24E-20	postive
NSUN7	AC232271.	0.338194	1.87E-12	postive
RAD52	AC232271.	0.336527	2.43E-12	postive
NEIL1	AC232271.	0.582587	1.01E-38	postive
YBX1	AC104126.	0.541635	1.05E-32	postive
TET1	AC104126.	0.353223	1.60E-13	postive
TET2	AC104126.	0.32267	2.06E-11	postive
NSUN3	PABPC4-A	0.406613	8.53E-18	postive
NSUN6	PABPC4-A	0.33056	6.19E-12	postive
NSUN7	PABPC4-A	0.305381	2.55E-10	postive
RAD52	PABPC4-A	0.31101	1.14E-10	postive
ZBTB33	PABPC4-A	0.323763	1.75E-11	postive
TET1	PABPC4-A	0.629115	1.12E-46	postive

TET2	PABPC4-A	0.557053	7.12E-35	postive
TET3	PABPC4-A	0.446155	1.69E-21	postive
RAD52	ZNF133-A	0.305956	2.35E-10	postive
MBD3	AC009005.	0.352426	1.83E-13	postive
NTHL1	AC009005.	0.326964	1.08E-11	postive
NSUN3	AC093515.	0.521009	5.61E-30	postive
TRDMT1	AC093515.	0.317254	4.61E-11	postive
NSUN3	PAXIP1-AS	0.300747	4.87E-10	postive
NSUN7	PAXIP1-AS	0.31686	4.89E-11	postive
RAD52	PAXIP1-AS	0.315164	6.27E-11	postive
MECP2	PAXIP1-AS	0.308727	1.59E-10	postive
ZBTB33	PAXIP1-AS	0.35329	1.59E-13	postive
ZBTB38	PAXIP1-AS	0.414716	1.63E-18	postive
ZBTB4	PAXIP1-AS	0.363457	2.79E-14	postive
TET1	PAXIP1-AS	0.305511	2.50E-10	postive
TET2	PAXIP1-AS	0.479828	4.69E-25	postive
TET3	PAXIP1-AS	0.331875	5.05E-12	postive
NSUN6	AC005306.	0.379668	1.53E-15	postive
RAD52	AC005306.	0.448295	1.03E-21	postive
NEIL1	AC005306.	0.505371	4.98E-28	postive
NSUN3	AL122035.	0.324568	1.55E-11	postive
TRDMT1	AL122035.	0.314592	6.81E-11	postive
MECP2	AL122035.	0.421891	3.61E-19	postive
TET1	AL122035.	0.359685	5.35E-14	postive
TET2	AL122035.	0.388951	2.71E-16	postive
NTHL1	AC022098.	0.325082	1.43E-11	postive
NSUN6	LINC01572	0.318299	3.95E-11	postive
NSUN7	LINC01572	0.32391	1.71E-11	postive
RAD52	LINC01572	0.324641	1.53E-11	postive
ZBTB33	LINC01572	0.310769	1.18E-10	postive
TET1	LINC01572	0.634016	1.35E-47	postive
TET2	LINC01572	0.548458	1.19E-33	postive
TET3	LINC01572	0.395288	8.01E-17	postive
NSUN6	LINC01355	0.469461	6.44E-24	postive
NSUN7	LINC01355	0.442102	4.28E-21	postive
RAD52	LINC01355	0.479842	4.68E-25	postive
NEIL1	LINC01355	0.462516	3.55E-23	postive
TET1	LINC01355	0.459432	7.47E-23	postive
TET2	LINC01355	0.413798	1.97E-18	postive
TET3	LINC01355	0.388407	3.00E-16	postive
NSUN3	AC129507.	0.466201	1.44E-23	postive
TRDMT1	AC129507.	0.303218	3.46E-10	postive
TET1	AC129507.	0.552047	3.70E-34	postive
TET2	AC129507.	0.326466	1.16E-11	postive
NEIL1	AC074212.	0.305984	2.34E-10	postive
NSUN3	COX10-AS	0.440128	6.69E-21	postive
DNMT1	COX10-AS	0.342982	8.67E-13	postive
DNMT3A	COX10-AS	0.309095	1.51E-10	postive
TRDMT1	COX10-AS	0.341876	1.04E-12	postive
UHRF2	COX10-AS	0.302926	3.60E-10	postive
ZBTB33	COX10-AS	0.404634	1.27E-17	postive
TET1	COX10-AS	0.321445	2.48E-11	postive
TET2	COX10-AS	0.426876	1.24E-19	postive
NSUN6	AC007881.	0.30918	1.49E-10	postive
NSUN7	AC007881.	0.397793	4.92E-17	postive
DNMT3A	AC007881.	0.384271	6.53E-16	postive
TET1	AC007881.	0.458436	9.48E-23	postive
TET3	AC007881.	0.330126	6.62E-12	postive

NSUN6	ZKSCAN2-	0.476154	1.20E-24	postive
NSUN7	ZKSCAN2-	0.353257	1.59E-13	postive
DNMT3A	ZKSCAN2-	0.314683	6.72E-11	postive
RAD52	ZKSCAN2-	0.430778	5.33E-20	postive
NEIL1	ZKSCAN2-	0.592347	2.77E-40	postive
TET1	ZKSCAN2-	0.323904	1.71E-11	postive
TET2	ZKSCAN2-	0.366873	1.53E-14	postive
NSUN5	SNHG15	0.502098	1.24E-27	postive
MBD3	SNHG15	0.383605	7.40E-16	postive
ZBTB38	SNHG15	-0.31239	9.38E-11	negative
ZBTB4	SNHG15	-0.30449	2.89E-10	negative
MBD3	AC040169.	0.31381	7.63E-11	postive
NTHL1	AC040169.	0.391394	1.70E-16	postive
NSUN3	AC005730.	0.538713	2.62E-32	postive
TRDMT1	AC005730.	0.363668	2.69E-14	postive
UHRF2	AC005730.	0.336082	2.61E-12	postive
TET1	AC005730.	0.575278	1.38E-37	postive
TET2	AC005730.	0.520033	7.48E-30	postive
NSUN6	CCDC28A-	0.440034	6.83E-21	postive
NSUN7	CCDC28A-	0.375119	3.52E-15	postive
RAD52	CCDC28A-	0.50604	4.13E-28	postive
SMUG1	CCDC28A-	0.301053	4.67E-10	postive
TET1	CCDC28A-	0.370481	8.10E-15	postive
SMUG1	U47924.2	0.381498	1.09E-15	postive
TET2	WARS2-IT	0.313202	8.34E-11	postive
NSUN6	AC008764.	0.385751	4.95E-16	postive
NSUN7	AC008764.	0.335824	2.72E-12	postive
RAD52	AC008764.	0.448116	1.08E-21	postive
MBD1	AC008764.	0.304255	2.99E-10	postive
MECP2	AC008764.	0.334511	3.34E-12	postive
NEIL1	AC008764.	0.576623	8.59E-38	postive
TET2	AC008764.	0.397703	5.00E-17	postive
TET3	AC008764.	0.303931	3.13E-10	postive
NSUN7	SRRM2-AS	0.406247	9.18E-18	postive
RAD52	SRRM2-AS	0.352061	1.95E-13	postive
TET1	SRRM2-AS	0.349636	2.92E-13	postive
NSUN5	AC002398.	0.31872	3.71E-11	postive
MBD3	AC002398.	0.429927	6.42E-20	postive
NEIL1	AC002398.	0.351371	2.19E-13	postive
NTHL1	AC002398.	0.359926	5.14E-14	postive
SMUG1	AC002398.	0.378711	1.83E-15	postive
ZBTB38	AC002398.	-0.31214	9.72E-11	negative
NSUN3	AC066613.	0.541672	1.03E-32	postive
NSUN6	AC066613.	0.381732	1.05E-15	postive
TRDMT1	AC066613.	0.302568	3.78E-10	postive
RAD52	AC066613.	0.329472	7.32E-12	postive
NEIL1	AC066613.	0.373639	4.60E-15	postive
TET1	AC066613.	0.484894	1.26E-25	postive
TET2	AC066613.	0.645855	6.98E-50	postive
TET3	AC066613.	0.339189	1.59E-12	postive
NSUN6	AP000692.	0.349641	2.91E-13	postive
NSUN7	AP000692.	0.319134	3.49E-11	postive
RAD52	AP000692.	0.35571	1.05E-13	postive
NEIL1	AP000692.	0.32011	3.02E-11	postive
ZBTB33	AP000692.	0.353689	1.48E-13	postive
TET1	AP000692.	0.459019	8.25E-23	postive
TET2	AP000692.	0.627254	2.47E-46	postive
TET3	AP000692.	0.442753	3.69E-21	postive

NSUN7	ADCY6-DT	0.386979	3.93E-16	postive
TET2	AC078820.	0.393456	1.14E-16	postive
NSUN7	ZNF529-A	0.404905	1.20E-17	postive
DNMT3A	ZNF529-A	0.303093	3.52E-10	postive
NSUN3	C1RL-AS1	0.373128	5.04E-15	postive
RAD52	C1RL-AS1	0.42472	1.98E-19	postive
MECP2	C1RL-AS1	0.354625	1.27E-13	postive
UHRF2	C1RL-AS1	0.363968	2.55E-14	postive
ZBTB33	C1RL-AS1	0.38183	1.03E-15	postive
TET1	C1RL-AS1	0.302646	3.74E-10	postive
TET2	C1RL-AS1	0.524802	1.82E-30	postive
DNMT3A	AC079807.	0.492672	1.61E-26	postive
MBD1	AC079807.	0.383757	7.19E-16	postive
ZBTB33	AC079807.	0.300693	4.91E-10	postive
TET3	AC079807.	0.409325	4.92E-18	postive
NSUN7	AP001469.	0.300868	4.79E-10	postive
DNMT3A	AP001469.	0.333856	3.70E-12	postive
TET1	AP001469.	0.354601	1.27E-13	postive
TET3	AP001469.	0.320652	2.79E-11	postive
NSUN3	TBL1XR1-A	0.343529	7.93E-13	postive
UHRF2	TBL1XR1-A	0.310038	1.32E-10	postive
TET1	TBL1XR1-A	0.650892	6.91E-51	postive
TET2	TBL1XR1-A	0.575959	1.09E-37	postive
TET3	TBL1XR1-A	0.347775	3.96E-13	postive
NSUN3	AC109460.	0.322407	2.14E-11	postive
NSUN6	AC109460.	0.321488	2.46E-11	postive
NSUN7	AC109460.	0.36764	1.34E-14	postive
DNMT3A	AC109460.	0.310184	1.29E-10	postive
RAD52	AC109460.	0.430433	5.75E-20	postive
MBD1	AC109460.	0.318368	3.91E-11	postive
MECP2	AC109460.	0.326617	1.13E-11	postive
NEIL1	AC109460.	0.409083	5.17E-18	postive
UHRF2	AC109460.	0.301584	4.34E-10	postive
ZBTB33	AC109460.	0.394994	8.48E-17	postive
TET1	AC109460.	0.343153	8.43E-13	postive
TET2	AC109460.	0.5567	8.01E-35	postive
TET3	AC109460.	0.426817	1.26E-19	postive
NSUN6	AC021491.	0.306567	2.16E-10	postive
NSUN7	AC021491.	0.372831	5.32E-15	postive
TET1	AC021491.	0.522837	3.27E-30	postive
TET3	AC021491.	0.366025	1.78E-14	postive
MECP2	AC093010.	0.339297	1.57E-12	postive
NSUN7	AL589765.	0.320101	3.03E-11	postive
MBD4	AL589765.	0.336698	2.37E-12	postive
NSUN3	PRR7-AS1	0.540089	1.70E-32	postive
TRDMT1	PRR7-AS1	0.348492	3.52E-13	postive
UHRF2	PRR7-AS1	0.344947	6.30E-13	postive
TET1	PRR7-AS1	0.520679	6.18E-30	postive
TET2	PRR7-AS1	0.526995	9.47E-31	postive
NSUN6	AL450384.	0.448279	1.04E-21	postive
NSUN7	AL450384.	0.333045	4.20E-12	postive
TET1	AL450384.	0.651623	4.92E-51	postive
TET2	AL450384.	0.388267	3.08E-16	postive
TET3	AL450384.	0.362948	3.05E-14	postive
NSUN6	FSIP2-AS2	0.363817	2.62E-14	postive
RAD52	FSIP2-AS2	0.343283	8.25E-13	postive
NEIL1	FSIP2-AS2	0.397647	5.06E-17	postive
TET2	FSIP2-AS2	0.301323	4.50E-10	postive

NTHL1	AL590617.	0.326068	1.23E-11	postive
NSUN3	FAM230C	0.435038	2.09E-20	postive
RAD52	AL110115.	0.32918	7.65E-12	postive
NEIL1	AL110115.	0.35762	7.62E-14	postive
TET1	AL110115.	0.300254	5.22E-10	postive
NTHL1	AP002748.	0.30905	1.52E-10	postive
NSUN6	AC011472.	0.300799	4.84E-10	postive
RAD52	AC011472.	0.336237	2.55E-12	postive
NEIL1	AC011472.	0.35391	1.43E-13	postive
NSUN7	AC004233.	0.346658	4.76E-13	postive
TET1	AC004233.	0.399973	3.21E-17	postive
NSUN5	AC130343.	0.316386	5.24E-11	postive
MBD3	AC130343.	0.320675	2.78E-11	postive
NTHL1	AC130343.	0.328579	8.40E-12	postive
TET1	AC010789.	0.474737	1.72E-24	postive
TET2	AC010789.	0.310559	1.22E-10	postive
TET3	AC010789.	0.400992	2.62E-17	postive
NSUN3	AC011466.	0.462344	3.70E-23	postive
NSUN7	AC011466.	0.31278	8.86E-11	postive
TRDMT1	AC011466.	0.360237	4.87E-14	postive
UHRF2	AC011466.	0.337319	2.15E-12	postive
ZBTB38	AC011466.	0.30868	1.60E-10	postive
TET1	AC011466.	0.590042	6.55E-40	postive
TET2	AC011466.	0.47187	3.53E-24	postive
TET3	AC011466.	0.367846	1.29E-14	postive
NSUN6	AC024060.	0.382574	8.96E-16	postive
RAD52	AC024060.	0.390296	2.09E-16	postive
NEIL1	AC024060.	0.557023	7.19E-35	postive
DNMT3A	AL606760.	0.328819	8.09E-12	postive
NSUN6	AC005332.	0.376005	3.00E-15	postive
NSUN7	AC005332.	0.451778	4.59E-22	postive
RAD52	AC005332.	0.367645	1.34E-14	postive
NEIL1	AC005332.	0.466441	1.36E-23	postive
TET1	AC005332.	0.386529	4.28E-16	postive
TET2	AC005332.	0.42767	1.05E-19	postive
NSUN6	ATP2C2-A	0.368924	1.07E-14	postive
RAD52	ATP2C2-A	0.31118	1.12E-10	postive
NEIL1	ATP2C2-A	0.328685	8.26E-12	postive
TET2	ATP2C2-A	0.314141	7.28E-11	postive
NSUN3	AC104938.	0.407367	7.32E-18	postive
ZBTB38	AC104938.	0.305609	2.47E-10	postive
NSUN3	AC011510.	0.744695	7.91E-74	postive
TRDMT1	AC011510.	0.486167	9.05E-26	postive
UHRF2	AC011510.	0.336301	2.52E-12	postive
ZBTB38	AC011510.	0.367269	1.43E-14	postive
TET1	AC011510.	0.485893	9.72E-26	postive
TET2	AC011510.	0.444954	2.23E-21	postive
NSUN7	AP005482.	0.328415	8.61E-12	postive
DNMT3A	AP005482.	0.303377	3.38E-10	postive
DNMT3A	AC138904.	0.395096	8.32E-17	postive
NSUN6	AC007128.	0.305704	2.44E-10	postive
DNMT3A	AC007128.	0.33169	5.19E-12	postive
TDG	AC007128.	0.331318	5.50E-12	postive
ZBTB33	AC007128.	0.428998	7.85E-20	postive
TET1	AC007128.	0.522231	3.91E-30	postive
TET2	AC007128.	0.47463	1.76E-24	postive
TET3	AC007128.	0.532379	1.85E-31	postive
NSUN6	RUSC1-AS	0.329241	7.58E-12	postive

NSUN7	RUSC1-AS	0.381196	1.16E-15	postive
RAD52	RUSC1-AS	0.445469	1.98E-21	postive
NEIL1	RUSC1-AS	0.506066	4.10E-28	postive
TET2	RUSC1-AS	0.410932	3.55E-18	postive
TET3	RUSC1-AS	0.338917	1.66E-12	postive
NSUN3	AC073517.	0.316998	4.79E-11	postive
NSUN7	AC073517.	0.312262	9.56E-11	postive
UHRF2	AC073517.	0.303753	3.21E-10	postive
ZBTB33	AC073517.	0.303924	3.13E-10	postive
TET1	AC073517.	0.602983	4.76E-42	postive
TET2	AC073517.	0.55836	4.61E-35	postive
TET3	AC073517.	0.413805	1.96E-18	postive
NSUN6	AC015802.	0.360826	4.40E-14	postive
NSUN7	AC015802.	0.354747	1.24E-13	postive
DNMT3A	AC015802.	0.327568	9.80E-12	postive
RAD52	AC015802.	0.45232	4.04E-22	postive
NEIL1	AC015802.	0.504177	6.95E-28	postive
ZBTB33	AL162595.	0.359078	5.94E-14	postive
TET2	AL162595.	0.311545	1.06E-10	postive
TET3	AL162595.	0.325408	1.36E-11	postive
NSUN3	AC010422.	0.419061	6.57E-19	postive
NSUN6	AC010422.	0.375015	3.59E-15	postive
RAD52	AC010422.	0.355249	1.14E-13	postive
NEIL1	AC010422.	0.339908	1.42E-12	postive
TET1	AC010422.	0.562737	1.06E-35	postive
TET2	AC010422.	0.535105	8.02E-32	postive
TET3	AC010422.	0.324216	1.63E-11	postive
ZBTB4	AC005180.	0.30611	2.30E-10	postive
TET1	LRP4-AS1	0.561622	1.54E-35	postive
TET2	LRP4-AS1	0.363037	3.00E-14	postive
NSUN3	NUTM2B-/	0.347945	3.85E-13	postive
NSUN6	NUTM2B-/	0.347858	3.91E-13	postive
NSUN7	NUTM2B-/	0.318651	3.75E-11	postive
TET1	NUTM2B-/	0.706266	2.49E-63	postive
TET2	NUTM2B-/	0.465653	1.65E-23	postive
TET3	NUTM2B-/	0.368312	1.19E-14	postive
NSUN6	AL442128.	0.321663	2.40E-11	postive
NSUN7	AL442128.	0.325394	1.37E-11	postive
DNMT3A	AL442128.	0.388431	2.99E-16	postive
RAD52	AL442128.	0.378451	1.92E-15	postive
MECP2	AL442128.	0.332652	4.47E-12	postive
NEIL1	AL442128.	0.393298	1.18E-16	postive
NSUN3	AL133415.	0.584198	5.63E-39	postive
ZBTB38	AL133415.	0.327176	1.04E-11	postive
NSUN3	AC012358.	0.353038	1.65E-13	postive
NSUN6	AC012358.	0.366355	1.68E-14	postive
RAD52	AC012358.	0.305344	2.56E-10	postive
NEIL1	AC012358.	0.363185	2.93E-14	postive
TET1	AC012358.	0.447642	1.20E-21	postive
TET2	AC012358.	0.612831	9.65E-44	postive
TET3	AC012358.	0.377808	2.16E-15	postive
NSUN3	AC068790.	0.432148	3.95E-20	postive
TRDMT1	AC068790.	0.31028	1.27E-10	postive
UHRF2	AC068790.	0.339887	1.43E-12	postive
TET1	AC068790.	0.686864	1.18E-58	postive
TET2	AC068790.	0.59393	1.53E-40	postive
TET3	AC068790.	0.359682	5.36E-14	postive
NSUN3	AC005522.	0.387694	3.43E-16	postive

TET1	AC005522.	0.647598	3.15E-50	postive
TET2	AC005522.	0.533727	1.23E-31	postive
TET3	AC005522.	0.362638	3.22E-14	postive
DNMT3A	MRPL20-A	0.337907	1.96E-12	postive
TET3	ACTN1-AS	-0.312	9.92E-11	negative
NSUN6	AC015923.	0.320728	2.76E-11	postive
NSUN7	AC015923.	0.302402	3.87E-10	postive
TET1	AC015923.	0.674773	6.41E-56	postive
TET2	AC015923.	0.435577	1.85E-20	postive
TET3	AC015923.	0.345532	5.73E-13	postive
NSUN3	RGMB-AS1	0.355318	1.13E-13	postive
NEIL1	RGMB-AS1	0.329839	6.91E-12	postive
NSUN6	AL139099.	0.447676	1.19E-21	postive
NSUN7	AL139099.	0.342044	1.01E-12	postive
DNMT3A	AL139099.	0.308469	1.65E-10	postive
RAD52	AL139099.	0.366379	1.67E-14	postive
MBD1	AL139099.	0.309719	1.38E-10	postive
MECP2	AL139099.	0.311957	9.99E-11	postive
NEIL1	AL139099.	0.34599	5.31E-13	postive
ZBTB33	AL139099.	0.387451	3.59E-16	postive
TET1	AL139099.	0.432261	3.85E-20	postive
TET2	AL139099.	0.580377	2.25E-38	postive
TET3	AL139099.	0.47643	1.12E-24	postive
TET1	AL162171.	0.338731	1.71E-12	postive
TET2	AL162171.	0.334414	3.39E-12	postive
ZBTB33	AC012313.	0.303956	3.12E-10	postive
UNG	FAM222A-	0.303411	3.36E-10	postive
NSUN3	SUGT1P4-	0.414138	1.83E-18	postive
NSUN7	SUGT1P4-	0.344231	7.08E-13	postive
TRDMT1	SUGT1P4-	0.392101	1.48E-16	postive
MECP2	SUGT1P4-	0.371011	7.37E-15	postive
TET1	SUGT1P4-	0.483088	2.02E-25	postive
TET2	SUGT1P4-	0.346435	4.94E-13	postive
NSUN7	AL050309.	0.333927	3.66E-12	postive
TET3	AL050309.	0.300797	4.84E-10	postive
NSUN3	AC020978.	0.38597	4.75E-16	postive
UHRF2	AC020978.	0.318179	4.02E-11	postive
TET1	AC020978.	0.579377	3.22E-38	postive
TET2	AC020978.	0.607255	8.92E-43	postive
TET3	AC020978.	0.358836	6.19E-14	postive
NSUN3	RORA-AS1	0.48809	5.46E-26	postive
TRDMT1	RORA-AS1	0.313215	8.32E-11	postive
UHRF2	RORA-AS1	0.336557	2.42E-12	postive
ZBTB33	RORA-AS1	0.323871	1.72E-11	postive
TET1	RORA-AS1	0.61985	5.50E-45	postive
TET2	RORA-AS1	0.549911	7.42E-34	postive
TET3	RORA-AS1	0.38557	5.12E-16	postive
NSUN6	LINC02481	0.553996	1.96E-34	postive
NEIL1	LINC02481	0.344487	6.79E-13	postive
DNMT3A	LINC0190	0.312977	8.62E-11	postive
TET1	LINC0190	0.303027	3.55E-10	postive
DNMT1	PTPRG-AS	0.335657	2.79E-12	postive
DNMT3A	PTPRG-AS	0.373342	4.85E-15	postive
TRDMT1	PTPRG-AS	0.405481	1.07E-17	postive
UNG	PTPRG-AS	0.350717	2.44E-13	postive
NSUN3	AC007681.	0.322326	2.17E-11	postive
NSUN7	AC007681.	0.305322	2.57E-10	postive
DNMT3A	AC007681.	0.30583	2.39E-10	postive

TET1	AC007681.	0.616482	2.19E-44	postive
TET2	AC007681.	0.375724	3.15E-15	postive
TET3	AC007681.	0.417295	9.52E-19	postive
NSUN3	AL358216.	0.313492	7.99E-11	postive
NSUN6	AL358216.	0.343174	8.40E-13	postive
NSUN7	AL358216.	0.301138	4.62E-10	postive
TET1	AL358216.	0.58097	1.81E-38	postive
TET2	AL358216.	0.549658	8.05E-34	postive
NSUN3	LINC0264¢	0.486754	7.76E-26	postive
TRDMT1	LINC0264¢	0.31851	3.83E-11	postive
UHRF2	LINC0264¢	0.312386	9.38E-11	postive
TET1	LINC0264¢	0.447634	1.20E-21	postive
TET2	LINC0264¢	0.541461	1.10E-32	postive
NSUN3	AC022893.	0.446732	1.48E-21	postive
TRDMT1	AC022893.	0.357641	7.59E-14	postive
TET1	AC022893.	0.677874	1.31E-56	postive
TET2	AC022893.	0.469511	6.36E-24	postive
TET3	AC022893.	0.333022	4.22E-12	postive
NSUN5	AP001160.	0.313353	8.16E-11	postive
MBD3	AP001160.	0.347892	3.89E-13	postive
NSUN5	LINC0102з	0.423058	2.82E-19	postive
MBD3	LINC0102з	0.494622	9.54E-27	postive
NTHL1	LINC0102з	0.560469	2.28E-35	postive
SMUG1	LINC0102з	0.378565	1.88E-15	postive
DNMT3A	AC009902.	0.317124	4.70E-11	postive
NEIL1	KIF1C-AS1	0.358706	6.33E-14	postive
NTHL1	AC084809.	0.410298	4.04E-18	postive
NSUN3	AC067852.	0.609297	3.97E-43	postive
TRDMT1	AC067852.	0.366724	1.58E-14	postive
MECP2	AC067852.	0.334842	3.17E-12	postive
UHRF2	AC067852.	0.358819	6.21E-14	postive
ZBTB38	AC067852.	0.391698	1.60E-16	postive
TET1	AC067852.	0.552056	3.69E-34	postive
TET2	AC067852.	0.658705	1.75E-52	postive
TET3	AC067852.	0.36643	1.66E-14	postive
NSUN6	AC020931.	0.330798	5.96E-12	postive
RAD52	AC020931.	0.345468	5.79E-13	postive
MBD1	AC020931.	0.301012	4.70E-10	postive
NEIL1	AC020931.	0.392067	1.49E-16	postive
TET2	AC020931.	0.385681	5.02E-16	postive
NSUN6	GATA2-AS	0.325261	1.39E-11	postive
NSUN7	GATA2-AS	0.352601	1.78E-13	postive
MBD1	GATA2-AS	0.372174	5.98E-15	postive
NSUN6	AC010201.	0.549068	9.75E-34	postive
NSUN7	AC010201.	0.468272	8.65E-24	postive
DNMT3A	AC010201.	0.344016	7.33E-13	postive
RAD52	AC010201.	0.473356	2.43E-24	postive
NEIL1	AC010201.	0.46351	2.78E-23	postive
TET1	AC010201.	0.479069	5.70E-25	postive
TET2	AC010201.	0.389353	2.51E-16	postive
TET3	AC010201.	0.373612	4.62E-15	postive
NSUN3	AC090589.	0.347662	4.04E-13	postive
NSUN6	AC090589.	0.338107	1.89E-12	postive
NSUN7	AC090589.	0.347356	4.25E-13	postive
DNMT3A	AC090589.	0.300289	5.19E-10	postive
RAD52	AC090589.	0.4189	6.80E-19	postive
NEIL1	AC090589.	0.419823	5.60E-19	postive
TET1	AC090589.	0.41829	7.73E-19	postive

TET2	AC090589.	0.525152	1.64E-30	postive
TET3	AC090589.	0.43137	4.68E-20	postive
NSUN6	AC012645.	0.319103	3.51E-11	postive
NSUN7	AC012645.	0.30368	3.24E-10	postive
TET1	AC012645.	0.455192	2.05E-22	postive
TET2	AC012645.	0.396648	6.15E-17	postive
TET3	AC012645.	0.382277	9.47E-16	postive
NSUN6	LNX1-AS2	0.32384	1.73E-11	postive
NSUN7	LNX1-AS2	0.372659	5.49E-15	postive
TET1	LNX1-AS2	0.418779	6.97E-19	postive
NSUN7	AC015908.	0.363271	2.88E-14	postive
NSUN6	AC004596.	0.35596	1.01E-13	postive
NSUN7	AC004596.	0.306297	2.24E-10	postive
ZBTB33	AC004596.	0.369373	9.87E-15	postive
TET1	AC004596.	0.331007	5.77E-12	postive
TET2	AC004596.	0.444711	2.36E-21	postive
TET3	AC004596.	0.383002	8.28E-16	postive
NSUN5	AC010997.	0.311167	1.12E-10	postive
MBD3	AC010997.	0.375113	3.52E-15	postive
NEIL1	AC010997.	0.302957	3.58E-10	postive
NTHL1	AC010997.	0.428001	9.75E-20	postive
NSUN6	HCG25	0.340777	1.24E-12	postive
NSUN7	HCG25	0.386043	4.69E-16	postive
RAD52	HCG25	0.376731	2.63E-15	postive
NEIL1	HCG25	0.442876	3.59E-21	postive
TET2	HCG25	0.333501	3.92E-12	postive
NSUN5	PPP4R3B-I	0.313915	7.52E-11	postive
DNMT3A	PPP4R3B-I	0.311592	1.05E-10	postive
MBD3	PPP4R3B-I	0.304123	3.04E-10	postive
NTHL1	PPP4R3B-I	0.313982	7.45E-11	postive
NSUN7	LINC01224	0.313817	7.63E-11	postive
DNMT3B	LINC01224	0.304357	2.95E-10	postive
RAD52	AC004943.	0.338637	1.74E-12	postive
NSUN7	AC006213.	0.349142	3.16E-13	postive
DNMT3A	AC006213.	0.302362	3.89E-10	postive
TET1	AC016924.	0.367267	1.43E-14	postive
NSUN6	AC074117.	0.394149	9.99E-17	postive
DNMT3A	AC074117.	0.530993	2.83E-31	postive
RAD52	AC074117.	0.359603	5.43E-14	postive
MBD1	AC074117.	0.350054	2.72E-13	postive
NEIL1	AC074117.	0.338614	1.75E-12	postive
TET3	AC074117.	0.472898	2.73E-24	postive
NEIL1	AC069307.	0.324896	1.47E-11	postive
TET2	AF127577.	0.42273	3.02E-19	postive
TET1	AL133297.	0.339979	1.41E-12	postive
TET2	AL133297.	0.453729	2.90E-22	postive
MBD4	NCK1-DT	0.44892	8.94E-22	postive
NSUN6	AL031432.	0.306571	2.16E-10	postive
NEIL1	AL031432.	0.345048	6.20E-13	postive
NSUN6	AL031716.	0.345925	5.37E-13	postive
TET1	AL031716.	0.434397	2.40E-20	postive
TET2	AL031716.	0.346705	4.73E-13	postive
TET1	DDN-AS1	0.377253	2.39E-15	postive
NSUN7	AC010538.	0.332297	4.72E-12	postive
NSUN6	AC090246.	0.380811	1.24E-15	postive
NSUN7	AC090246.	0.365508	1.95E-14	postive
MBD1	AC090246.	0.416722	1.07E-18	postive
NEIL1	AC090246.	0.320035	3.05E-11	postive

TET3	AC090246.	0.317595	4.39E-11	postive
NSUN3	GARS1-DT	0.371576	6.66E-15	postive
NSUN6	GARS1-DT	0.405171	1.14E-17	postive
NSUN7	GARS1-DT	0.351557	2.12E-13	postive
RAD52	GARS1-DT	0.429722	6.71E-20	postive
NEIL1	GARS1-DT	0.386907	3.98E-16	postive
TET1	GARS1-DT	0.548361	1.22E-33	postive
TET2	GARS1-DT	0.451911	4.45E-22	postive
TET3	GARS1-DT	0.354566	1.28E-13	postive
TET2	LINC0133	0.318187	4.02E-11	postive
NSUN4	GRHL3-AS	0.308276	1.69E-10	postive
NSUN6	GRHL3-AS	0.420398	4.96E-19	postive
NSUN7	GRHL3-AS	0.35827	6.82E-14	postive
MBD1	GRHL3-AS	0.300153	5.29E-10	postive
NEIL1	GRHL3-AS	0.311121	1.13E-10	postive
ZBTB33	GRHL3-AS	0.338774	1.70E-12	postive
TET2	GRHL3-AS	0.459157	7.98E-23	postive
TET3	GRHL3-AS	0.440085	6.75E-21	postive
NSUN6	SBNO1-AS	0.432423	3.72E-20	postive
RAD52	SBNO1-AS	0.319131	3.49E-11	postive
NEIL1	SBNO1-AS	0.416848	1.04E-18	postive
RAD52	STAG3L5P	0.356181	9.73E-14	postive
NEIL1	STAG3L5P	0.515342	2.93E-29	postive
NSUN6	AP001065.	0.350371	2.58E-13	postive
NSUN3	MIR222HG	0.428852	8.11E-20	postive
TRDMT1	MIR222HG	0.32457	1.55E-11	postive
UHRF2	MIR222HG	0.332219	4.78E-12	postive
YBX1	AL031289.	0.467454	1.06E-23	postive
RAD52	AL031289.	0.307632	1.86E-10	postive
DNMT3A	AC016727.	0.345738	5.54E-13	postive
TET1	AC016727.	0.329475	7.31E-12	postive
TET2	AC016727.	0.347582	4.09E-13	postive
TET3	AC016727.	0.4083	6.06E-18	postive
DNMT3A	INSIG1-DT	0.332847	4.34E-12	postive
TET3	LINC0062	0.37099	7.40E-15	postive
NEIL1	LINC0256	0.326332	1.18E-11	postive
NSUN6	AC003070.	0.439674	7.40E-21	postive
NSUN7	AC003070.	0.32029	2.94E-11	postive
ALYREF	AC003070.	-0.30838	1.67E-10	negative
RAD52	AC003070.	0.376735	2.62E-15	postive
MBD1	AC003070.	0.454568	2.38E-22	postive
NEIL1	AC003070.	0.549492	8.50E-34	postive
TET2	AC003070.	0.347305	4.28E-13	postive
TET3	AC003070.	0.31708	4.73E-11	postive
NSUN3	AC108463.	0.538402	2.88E-32	postive
TRDMT1	AC108463.	0.392841	1.29E-16	postive
ZBTB38	AC108463.	0.330824	5.94E-12	postive
TET1	AC108463.	0.525828	1.34E-30	postive
TET2	AC108463.	0.300375	5.13E-10	postive
NSUN7	AC095055.	0.319472	3.32E-11	postive
TET1	AC095055.	0.445363	2.03E-21	postive
TET2	AC095055.	0.402571	1.92E-17	postive
NSUN6	AL136295.	0.3327	4.44E-12	postive
RAD52	AL136295.	0.382924	8.40E-16	postive
NEIL1	AL136295.	0.42732	1.13E-19	postive
TET1	AC013287.	0.30342	3.36E-10	postive
NSUN3	AC090772.	0.352203	1.90E-13	postive
UHRF2	AC090772.	0.309842	1.35E-10	postive

TET2	AC090772.	0.479571	5.01E-25	postive
NSUN3	AL731577.	0.391862	1.55E-16	postive
DNMT1	AL731577.	0.419394	6.13E-19	postive
TRDMT1	AL731577.	0.318712	3.72E-11	postive
UHRF1	AL731577.	0.317987	4.14E-11	postive
ZBTB33	AL731577.	0.400535	2.87E-17	postive
ZBTB38	AL731577.	0.455992	1.70E-22	postive
TET1	AL731577.	0.426913	1.23E-19	postive
TET2	AL731577.	0.453972	2.74E-22	postive
TET3	AL731577.	0.417313	9.48E-19	postive
NEIL1	Z97653.1	0.304982	2.70E-10	postive
NSUN6	AC011481.	0.431161	4.90E-20	postive
NSUN7	AC011481.	0.377576	2.25E-15	postive
RAD52	AC011481.	0.429407	7.19E-20	postive
NEIL1	AC011481.	0.525088	1.68E-30	postive
NSUN3	AP4B1-AS	0.325166	1.41E-11	postive
NSUN6	AP4B1-AS	0.332049	4.91E-12	postive
NSUN7	AP4B1-AS	0.313143	8.41E-11	postive
DNMT3A	AP4B1-AS	0.314981	6.44E-11	postive
RAD52	AP4B1-AS	0.424841	1.93E-19	postive
MBD1	AP4B1-AS	0.300429	5.09E-10	postive
MECP2	AP4B1-AS	0.330915	5.86E-12	postive
NEIL1	AP4B1-AS	0.456879	1.38E-22	postive
UHRF2	AP4B1-AS	0.332991	4.24E-12	postive
ZBTB33	AP4B1-AS	0.320144	3.01E-11	postive
TET2	AP4B1-AS	0.54412	4.76E-33	postive
TET3	AP4B1-AS	0.39324	1.19E-16	postive
NSUN3	AP001020.	0.403805	1.50E-17	postive
NSUN6	AP001020.	0.34848	3.53E-13	postive
NSUN7	AP001020.	0.319394	3.36E-11	postive
TET1	AP001020.	0.701221	4.45E-62	postive
TET2	AP001020.	0.514663	3.56E-29	postive
TET3	AP001020.	0.408122	6.29E-18	postive
NSUN7	AC026704.	0.417312	9.48E-19	postive
TET1	AC026704.	0.356087	9.89E-14	postive
TET2	AC026704.	0.374692	3.80E-15	postive
NSUN7	HCG14	0.310054	1.31E-10	postive
DNMT3A	HCG14	0.301382	4.46E-10	postive
NSUN5	AC099791.	0.309277	1.47E-10	postive
MBD3	AC099791.	0.397023	5.72E-17	postive
NEIL1	AC099791.	0.334952	3.12E-12	postive
NTHL1	AC099791.	0.515724	2.62E-29	postive
TET2	AC083906.	0.437134	1.31E-20	postive
NSUN3	AC008635.	0.438719	9.18E-21	postive
UHRF2	AC008635.	0.354726	1.25E-13	postive
ZBTB33	AC008635.	0.313366	8.14E-11	postive
TET1	AC008635.	0.49827	3.54E-27	postive
TET2	AC008635.	0.613058	8.80E-44	postive
TET3	AC008635.	0.372533	5.61E-15	postive
DNMT3A	AP005262.	0.33833	1.83E-12	postive
TET1	AP005262.	0.319531	3.29E-11	postive
TET3	AP005262.	0.35876	6.27E-14	postive
NSUN3	DST-AS1	0.404219	1.38E-17	postive
TRDMT1	DST-AS1	0.345989	5.31E-13	postive
TET1	DST-AS1	0.313356	8.15E-11	postive
SMUG1	FARP1-AS	0.369908	8.97E-15	postive
NSUN3	OSMR-AS:	0.491198	2.39E-26	postive
TRDMT1	OSMR-AS:	0.35965	5.39E-14	postive

NSUN5	HNRNPD-	0.377493	2.29E-15	postive
MBD3	HNRNPD-	0.396725	6.06E-17	postive
NTHL1	HNRNPD-	0.452663	3.73E-22	postive
NSUN6	ZDHHC20-	0.449452	7.90E-22	postive
NSUN7	ZDHHC20-	0.334372	3.42E-12	postive
RAD52	ZDHHC20-	0.355797	1.04E-13	postive
ZBTB33	ZDHHC20-	0.305828	2.40E-10	postive
TET1	ZDHHC20-	0.558129	4.98E-35	postive
TET2	ZDHHC20-	0.498377	3.44E-27	postive
TET3	ZDHHC20-	0.445024	2.20E-21	postive
NSUN6	AL031709.	0.332695	4.44E-12	postive
NSUN7	AL031709.	0.31514	6.29E-11	postive
DNMT3A	AL031709.	0.310398	1.25E-10	postive
RAD52	AL031709.	0.373182	4.99E-15	postive
MBD1	AL031709.	0.409695	4.57E-18	postive
NEIL1	AL031709.	0.331071	5.72E-12	postive
TET2	AL031709.	0.398388	4.38E-17	postive
TET3	AL031709.	0.352956	1.68E-13	postive
NSUN3	AC078795.	0.469127	7.00E-24	postive
NSUN6	AC078795.	0.364903	2.17E-14	postive
NSUN7	AC078795.	0.337435	2.11E-12	postive
TRDMT1	AC078795.	0.3142	7.21E-11	postive
RAD52	AC078795.	0.323509	1.82E-11	postive
MECP2	AC078795.	0.331972	4.97E-12	postive
UHRF2	AC078795.	0.372378	5.77E-15	postive
ZBTB33	AC078795.	0.382714	8.73E-16	postive
ZBTB38	AC078795.	0.314481	6.92E-11	postive
TET1	AC078795.	0.625799	4.58E-46	postive
TET2	AC078795.	0.654928	1.05E-51	postive
TET3	AC078795.	0.433679	2.82E-20	postive
NEIL1	AC132938.	0.389226	2.57E-16	postive
NSUN6	AC010973.	0.337435	2.11E-12	postive
RAD52	AC010973.	0.394591	9.17E-17	postive
NEIL1	AC010973.	0.507628	2.64E-28	postive
NSUN6	AL731567.	0.507488	2.75E-28	postive
NSUN7	AL731567.	0.413682	2.02E-18	postive
RAD52	AL731567.	0.373828	4.45E-15	postive
NEIL1	AL731567.	0.464686	2.09E-23	postive
TET2	AL731567.	0.398088	4.64E-17	postive
TET3	AL731567.	0.3228	2.02E-11	postive
MECP2	AC007637.	0.398762	4.07E-17	postive
ZBTB38	AC007637.	0.319394	3.36E-11	postive
ZBTB4	AC007637.	0.303566	3.29E-10	postive
TET2	AC007637.	0.352624	1.77E-13	postive
NSUN3	AC099811.	0.498293	3.52E-27	postive
TRDMT1	AC099811.	0.311749	1.03E-10	postive
UHRF2	AC099811.	0.360937	4.32E-14	postive
ZBTB38	AC099811.	0.305081	2.66E-10	postive
TET1	AC099811.	0.555297	1.27E-34	postive
TET2	AC099811.	0.606987	9.92E-43	postive
TET3	AC099811.	0.300402	5.11E-10	postive
TET1	LINC01967	0.34398	7.37E-13	postive
TET2	LINC01967	0.370286	8.39E-15	postive
NSUN7	AL137244.	0.359109	5.91E-14	postive
RAD52	AL137244.	0.373719	4.53E-15	postive
NEIL1	AL137244.	0.333011	4.23E-12	postive
TET1	AL137244.	0.309979	1.33E-10	postive
NSUN3	AC010976.	0.34918	3.14E-13	postive

NSUN6	AC010976.	0.344679	6.58E-13	postive
NSUN7	AC010976.	0.327145	1.05E-11	postive
ZBTB33	AC010976.	0.301473	4.41E-10	postive
TET1	AC010976.	0.67614	3.19E-56	postive
TET2	AC010976.	0.570647	7.01E-37	postive
TET3	AC010976.	0.410486	3.88E-18	postive
NSUN7	SNRK-AS1	0.32368	1.77E-11	postive
TET3	SNRK-AS1	0.342248	9.76E-13	postive
NSUN3	Z98885.3	0.554897	1.45E-34	postive
TRDMT1	Z98885.3	0.383498	7.55E-16	postive
MECP2	Z98885.3	0.300827	4.82E-10	postive
UHRF2	Z98885.3	0.319091	3.52E-11	postive
TET1	Z98885.3	0.704696	6.15E-63	postive
TET2	Z98885.3	0.567528	2.06E-36	postive
TET3	Z98885.3	0.394652	9.06E-17	postive
NEIL1	AP001453.	0.487671	6.09E-26	postive
NSUN7	AC011498.	0.32585	1.27E-11	postive
RAD52	AC011498.	0.333335	4.02E-12	postive
NEIL1	AC011498.	0.400553	2.86E-17	postive
TET2	AC011498.	0.31847	3.85E-11	postive
NSUN6	Z93403.1	0.357811	7.38E-14	postive
TET1	Z93403.1	0.44136	5.06E-21	postive
TET3	Z93403.1	0.318977	3.57E-11	postive
NSUN3	LINC01697	0.552952	2.75E-34	postive
TET1	AC105429.	0.474279	1.93E-24	postive
TET2	AC105429.	0.544382	4.38E-33	postive
MBD1	AC093582.	0.301962	4.12E-10	postive
TET3	AC093582.	0.346498	4.89E-13	postive
TET1	AP003086.	0.460618	5.61E-23	postive
TET2	AP003086.	0.311093	1.13E-10	postive
RAD52	SCAT2	0.471108	4.27E-24	postive
NEIL1	SCAT2	0.456123	1.65E-22	postive
SMUG1	SCAT2	0.443593	3.05E-21	postive
DNMT3A	SLC30A6-I	0.354676	1.26E-13	postive
NSUN3	LRRC8C-D	0.439938	6.98E-21	postive
TRDMT1	LRRC8C-D	0.316301	5.31E-11	postive
TET1	LRRC8C-D	0.459146	8.00E-23	postive
NSUN3	AC012615.	0.362353	3.38E-14	postive
NSUN6	AC012615.	0.431472	4.58E-20	postive
NSUN7	AC012615.	0.32107	2.62E-11	postive
DNMT3A	AC012615.	0.304571	2.86E-10	postive
RAD52	AC012615.	0.456801	1.40E-22	postive
MBD1	AC012615.	0.41184	2.94E-18	postive
NEIL1	AC012615.	0.523107	3.02E-30	postive
TET2	AC012615.	0.516871	1.88E-29	postive
TET3	AC012615.	0.418985	6.68E-19	postive
NSUN3	AC025280.	0.495367	7.80E-27	postive
UHRF2	AC025280.	0.327152	1.04E-11	postive
ZBTB38	AC025280.	0.307653	1.85E-10	postive
TET1	AC025280.	0.360648	4.54E-14	postive
TET2	AC025280.	0.520189	7.14E-30	postive
NSUN2	PP7080	0.449757	7.36E-22	postive
NSUN6	AL096870.	0.351307	2.21E-13	postive
NSUN7	AL096870.	0.351578	2.11E-13	postive
RAD52	AL096870.	0.331588	5.28E-12	postive
MBD1	AL096870.	0.393634	1.10E-16	postive
NEIL1	AL096870.	0.466709	1.27E-23	postive
NSUN3	AC084018.	0.337371	2.13E-12	postive

NSUN6	AC084018.	0.400822	2.71E-17	postive
NSUN7	AC084018.	0.352216	1.90E-13	postive
DNMT3A	AC084018.	0.348063	3.78E-13	postive
RAD52	AC084018.	0.341748	1.06E-12	postive
MBD1	AC084018.	0.336535	2.43E-12	postive
MECP2	AC084018.	0.390523	2.01E-16	postive
NEIL1	AC084018.	0.373109	5.06E-15	postive
TDG	AC084018.	0.307299	1.95E-10	postive
ZBTB33	AC084018.	0.352481	1.82E-13	postive
TET1	AC084018.	0.364243	2.43E-14	postive
TET2	AC084018.	0.504433	6.47E-28	postive
TET3	AC084018.	0.432111	3.98E-20	postive
NSUN6	AP000697.	0.375627	3.21E-15	postive
NEIL1	AP000697.	0.372289	5.86E-15	postive
TET2	AP000697.	0.388332	3.04E-16	postive
NEIL1	AC090425.	0.425618	1.63E-19	postive
SMUG1	AC090425.	0.344187	7.13E-13	postive
NSUN7	FBXL19-AS	0.318612	3.77E-11	postive
DNMT3A	FBXL19-AS	0.320705	2.77E-11	postive
TET1	FBXL19-AS	0.35449	1.30E-13	postive
TET3	FBXL19-AS	0.460586	5.66E-23	postive
NSUN3	AC131934.	0.512341	6.94E-29	postive
TRDMT1	AC131934.	0.340209	1.35E-12	postive
UHRF2	AC131934.	0.351265	2.22E-13	postive
TET1	AC131934.	0.62435	8.43E-46	postive
TET2	AC131934.	0.585409	3.62E-39	postive
TET3	AC131934.	0.344704	6.55E-13	postive
NSUN3	AC007319.	0.504367	6.59E-28	postive
TRDMT1	AC007319.	0.37365	4.59E-15	postive
TET1	AC007319.	0.493466	1.30E-26	postive
TET2	AC007319.	0.424497	2.07E-19	postive
NSUN6	LINC01376	0.411287	3.30E-18	postive
RAD52	LINC01376	0.398971	3.90E-17	postive
TET1	LINC01376	0.400375	2.96E-17	postive
TET2	LINC01376	0.450275	6.53E-22	postive
TET3	LINC01376	0.446806	1.46E-21	postive
DNMT3A	KCNMB2-/	0.312157	9.70E-11	postive
SMUG1	KCNMB2-/	0.386388	4.39E-16	postive
TET1	ALMS1-IT1	0.546754	2.05E-33	postive
TET3	ALMS1-IT1	0.319354	3.38E-11	postive
NSUN7	AC012184.	0.31842	3.88E-11	postive
TET1	AC012184.	0.425506	1.67E-19	postive
NSUN6	AL691482.	0.35604	9.97E-14	postive
RAD52	AL691482.	0.314887	6.53E-11	postive
NEIL1	AL691482.	0.449158	8.46E-22	postive
SMUG1	STIM2-AS1	0.300691	4.91E-10	postive
MBD3	AC005775.	0.394312	9.68E-17	postive
NTHL1	AC005775.	0.407255	7.49E-18	postive
NSUN3	AC026771.	0.332574	4.52E-12	postive
NSUN6	AC026771.	0.328308	8.75E-12	postive
UHRF2	AC026771.	0.326383	1.17E-11	postive
ZBTB33	AC026771.	0.303547	3.30E-10	postive
TET1	AC026771.	0.548998	9.97E-34	postive
TET2	AC026771.	0.594299	1.33E-40	postive
TET3	AC026771.	0.378452	1.92E-15	postive
NSUN3	AC005479.	0.367222	1.44E-14	postive
UHRF2	AC005479.	0.331849	5.07E-12	postive
ZBTB38	AC005479.	0.364717	2.24E-14	postive

TET1	AC005479.	0.371379	6.90E-15	postive
TET2	AC005479.	0.500375	1.99E-27	postive
NTHL1	AL139246.	0.305822	2.40E-10	postive
NEIL1	AC015849.	0.340756	1.24E-12	postive
NSUN3	AC079313.	0.319077	3.52E-11	postive
ZBTB38	AC079313.	0.304054	3.07E-10	postive
TET1	AC079313.	0.354364	1.32E-13	postive
TET2	AC079313.	0.346109	5.21E-13	postive
NSUN7	AC007292.	0.316105	5.46E-11	postive
NEIL1	AC007292.	0.417109	9.89E-19	postive
TET1	AC007292.	0.426333	1.40E-19	postive
TET2	AC007292.	0.46109	5.01E-23	postive
TET3	AC007292.	0.365605	1.92E-14	postive
NSUN3	ODF2-AS1	0.390694	1.94E-16	postive
NSUN7	ODF2-AS1	0.313153	8.40E-11	postive
NEIL1	ODF2-AS1	0.300215	5.24E-10	postive
UHRF2	ODF2-AS1	0.343644	7.79E-13	postive
ZBTB33	ODF2-AS1	0.32296	1.97E-11	postive
TET1	ODF2-AS1	0.560541	2.22E-35	postive
TET2	ODF2-AS1	0.575281	1.38E-37	postive
TET3	ODF2-AS1	0.380485	1.32E-15	postive
MECP2	CKMT2-AS	0.30653	2.17E-10	postive
NSUN7	AL161729.	0.32627	1.20E-11	postive
DNMT3A	AL161729.	0.336308	2.52E-12	postive
RAD52	AL161729.	0.347524	4.13E-13	postive
NEIL1	AL161729.	0.426345	1.39E-19	postive
NSUN7	AC008543.	0.397558	5.15E-17	postive
DNMT3A	AC008543.	0.364817	2.20E-14	postive
UHRF2	AC008543.	0.326942	1.08E-11	postive
ZBTB33	AC008543.	0.353342	1.57E-13	postive
TET1	AC008543.	0.57388	2.26E-37	postive
TET2	AC008543.	0.485062	1.21E-25	postive
TET3	AC008543.	0.468318	8.55E-24	postive
NSUN6	LINC00174	0.326227	1.20E-11	postive
NEIL1	LINC00174	0.372407	5.74E-15	postive
NEIL1	SNHG12	0.476842	1.01E-24	postive
NSUN5	EXOC3-AS	0.327157	1.04E-11	postive
NSUN6	AC108860.	0.335469	2.88E-12	postive
NSUN7	AC108860.	0.358927	6.10E-14	postive
DNMT3A	AC108860.	0.41288	2.38E-18	postive
TET3	AC108860.	0.388332	3.04E-16	postive
NSUN3	ITFG1-AS1	0.330269	6.47E-12	postive
NSUN6	ITFG1-AS1	0.333678	3.81E-12	postive
NSUN7	ITFG1-AS1	0.313146	8.41E-11	postive
ZBTB33	ITFG1-AS1	0.339289	1.57E-12	postive
TET1	ITFG1-AS1	0.505492	4.82E-28	postive
TET2	ITFG1-AS1	0.618772	8.58E-45	postive
TET3	ITFG1-AS1	0.393141	1.21E-16	postive
NSUN6	AC243830.	0.375724	3.15E-15	postive
RAD52	AC243830.	0.372704	5.44E-15	postive
NEIL1	AC243830.	0.381717	1.05E-15	postive
NSUN7	AC005837.	0.312964	8.63E-11	postive
DNMT1	AC005837.	0.327402	1.01E-11	postive
DNMT3A	AC005837.	0.47117	4.21E-24	postive
ALYREF	AC005837.	0.34499	6.26E-13	postive
UNG	AC005837.	0.33786	1.97E-12	postive
TET1	AC005837.	0.383127	8.09E-16	postive
NSUN3	C2-AS1	0.490167	3.15E-26	postive

TRDMT1	C2-AS1	0.386881	4.00E-16	postive
TET1	C2-AS1	0.303596	3.28E-10	postive
NEIL1	AC015726.	0.425266	1.76E-19	postive
NSUN5	AC004832.	0.317125	4.70E-11	postive
NSUN6	AC010761.	0.405981	9.68E-18	postive
RAD52	AC010761.	0.408766	5.52E-18	postive
NEIL1	AC010761.	0.510425	1.20E-28	postive
NSUN3	AC007365.	0.380293	1.37E-15	postive
UHRF2	AC007365.	0.331515	5.34E-12	postive
ZBTB33	AC007365.	0.332004	4.95E-12	postive
TET1	AC007365.	0.663617	1.64E-53	postive
TET2	AC007365.	0.542352	8.34E-33	postive
TET3	AC007365.	0.351007	2.32E-13	postive
DNMT3A	AC084864.	0.34105	1.18E-12	postive
NSUN6	AC098484.	0.357481	7.81E-14	postive
RAD52	AC098484.	0.36206	3.56E-14	postive
NEIL1	AC098484.	0.410795	3.65E-18	postive
NSUN6	MIR200CH	0.302023	4.08E-10	postive
DNMT1	MIR200CH	-0.32584	1.28E-11	negative
NEIL1	MIR200CH	0.387145	3.81E-16	postive
NTHL1	MIR200CH	0.366155	1.74E-14	postive
SMUG1	MIR200CH	0.422864	2.94E-19	postive
ZBTB38	MIR200CH	-0.31134	1.09E-10	negative
NSUN6	LINC00885	0.328682	8.26E-12	postive
NSUN7	LINC00885	0.398724	4.10E-17	postive
SMUG1	LINC00885	0.391445	1.68E-16	postive
TET3	LINC00885	0.377643	2.22E-15	postive
NSUN6	MCF2L-AS	0.446502	1.56E-21	postive
NSUN7	MCF2L-AS	0.342987	8.66E-13	postive
NEIL1	MCF2L-AS	0.407884	6.60E-18	postive
SMUG1	MCF2L-AS	0.343848	7.53E-13	postive
NSUN6	AL583856.	0.431266	4.79E-20	postive
NSUN7	AL583856.	0.332169	4.82E-12	postive
NEIL1	AL583856.	0.421661	3.79E-19	postive
NSUN6	AC023794.	0.329205	7.62E-12	postive
NSUN7	AC023794.	0.309016	1.52E-10	postive
TET1	AC023794.	0.657813	2.68E-52	postive
TET2	AC023794.	0.460319	6.03E-23	postive
TET3	AC023794.	0.344177	7.14E-13	postive
TET2	DARS1-AS	0.382427	9.21E-16	postive
NSUN7	AL121917.	0.390978	1.84E-16	postive
RAD52	AL121917.	0.353182	1.61E-13	postive
NEIL1	AL121917.	0.315757	5.75E-11	postive
NTHL1	AC015912.	0.366008	1.79E-14	postive
SMUG1	AC015912.	0.330777	5.98E-12	postive
DNMT1	AC004943.	0.42122	4.17E-19	postive
TRDMT1	AC004943.	0.331573	5.29E-12	postive
UHRF1	AC004943.	0.333719	3.78E-12	postive
UHRF2	AC004943.	0.392019	1.51E-16	postive
TET1	AC004943.	0.341827	1.04E-12	postive
NSUN6	AC130462.	0.394291	9.72E-17	postive
RAD52	AC130462.	0.349668	2.90E-13	postive
NEIL1	AC130462.	0.346576	4.83E-13	postive
NSUN3	LINC01203	0.502671	1.06E-27	postive
TRDMT1	LINC01203	0.331351	5.47E-12	postive
NSUN6	AC008121.	0.363711	2.67E-14	postive
NSUN7	AC008121.	0.348801	3.35E-13	postive
TET1	AC008121.	0.583066	8.50E-39	postive

TET2	AC008121.	0.317978	4.14E-11	postive
TET3	AC008121.	0.330688	6.06E-12	postive
NSUN3	AC006460.	0.424197	2.21E-19	postive
TET1	AC006460.	0.382835	8.54E-16	postive
TET2	AC006460.	0.405863	9.92E-18	postive
NSUN3	AC008731.	0.513096	5.59E-29	postive
UHRF2	AC008731.	0.326321	1.19E-11	postive
ZBTB38	AC008731.	0.306353	2.22E-10	postive
TET1	AC008731.	0.496548	5.66E-27	postive
TET2	AC008731.	0.628619	1.38E-46	postive
TET3	AC008731.	0.350084	2.71E-13	postive
NSUN6	AC068338.	0.332133	4.85E-12	postive
NEIL1	AC068338.	0.323063	1.94E-11	postive
SMUG1	AC068338.	0.358149	6.96E-14	postive
DNMT3A	AC117395.	0.303464	3.34E-10	postive
NEIL1	AC117395.	0.414767	1.61E-18	postive
NSUN3	CDC42-IT1	0.379833	1.49E-15	postive
NSUN6	CDC42-IT1	0.312865	8.76E-11	postive
UHRF2	CDC42-IT1	0.310198	1.29E-10	postive
TET1	CDC42-IT1	0.674802	6.31E-56	postive
TET2	CDC42-IT1	0.560546	2.22E-35	postive
TET3	CDC42-IT1	0.367286	1.43E-14	postive
NTHL1	AP004609.	0.381985	1.00E-15	postive
TET2	AP003059.	0.311747	1.03E-10	postive
DNMT3A	FGF12-AS2	0.43685	1.39E-20	postive
ZBTB33	FGF12-AS2	0.309909	1.34E-10	postive
TET1	FGF12-AS2	0.386612	4.21E-16	postive
TET3	FGF12-AS2	0.312685	8.99E-11	postive
NSUN3	AC011442.	0.354236	1.35E-13	postive
NSUN7	AC011442.	0.353418	1.55E-13	postive
TET1	AC011442.	0.551892	3.89E-34	postive
TET2	AC011442.	0.52892	5.30E-31	postive
TET3	AC011442.	0.430099	6.18E-20	postive
NEIL1	AC132192.	0.408559	5.75E-18	postive
DNMT1	CENATAC-	0.428351	9.04E-20	postive
MBD1	CENATAC-	0.314001	7.42E-11	postive
MBD4	CENATAC-	0.379004	1.73E-15	postive
UHRF1	CENATAC-	0.377909	2.12E-15	postive
UHRF2	CENATAC-	0.34056	1.28E-12	postive
ZBTB33	CENATAC-	0.321473	2.47E-11	postive
TET3	CENATAC-	0.337411	2.12E-12	postive
TET2	AL136985.	0.309646	1.39E-10	postive
NSUN3	SDCBP2-A	0.570966	6.28E-37	postive
TRDMT1	SDCBP2-A	0.390742	1.92E-16	postive
UHRF2	SDCBP2-A	0.368417	1.17E-14	postive
ZBTB38	SDCBP2-A	0.369	1.05E-14	postive
TET1	SDCBP2-A	0.629159	1.10E-46	postive
TET2	SDCBP2-A	0.566048	3.43E-36	postive
TET3	SDCBP2-A	0.36462	2.28E-14	postive
NSUN3	AC037198.	0.344305	6.99E-13	postive
NSUN3	PRR34	0.373868	4.41E-15	postive
UHRF2	PRR34	0.307989	1.76E-10	postive
ZBTB38	PRR34	0.323486	1.82E-11	postive
TET1	PRR34	0.350041	2.73E-13	postive
TET2	PRR34	0.369351	9.90E-15	postive
NSUN6	AL139349.	0.444362	2.56E-21	postive
NSUN7	AL139349.	0.300306	5.18E-10	postive
RAD52	AL139349.	0.337457	2.10E-12	postive

MBD1	AL139349.	0.310003	1.32E-10	postive
NEIL1	AL139349.	0.615203	3.69E-44	postive
TET2	AL139349.	0.321173	2.58E-11	postive
NSUN6	AL162258.	0.376799	2.59E-15	postive
NEIL1	AL162258.	0.562033	1.34E-35	postive
NSUN6	LINC02042	0.309998	1.32E-10	postive
TET1	LINC02042	0.641723	4.50E-49	postive
TET2	LINC02042	0.410908	3.56E-18	postive
TET3	LINC02042	0.331088	5.70E-12	postive
DNMT3A	AP001107.	0.303177	3.48E-10	postive
NEIL1	LINC00847	0.334482	3.36E-12	postive
NSUN3	AC007619.	0.386562	4.25E-16	postive
UHRF2	AC007619.	0.340155	1.37E-12	postive
TET1	AC007619.	0.563818	7.34E-36	postive
TET2	AC007619.	0.607024	9.78E-43	postive
TET3	AC007619.	0.357482	7.80E-14	postive
NSUN6	AC027801.	0.362272	3.43E-14	postive
NSUN7	AC027801.	0.316192	5.39E-11	postive
RAD52	AC027801.	0.348039	3.80E-13	postive
NEIL1	AC027801.	0.365192	2.06E-14	postive
TET1	AC027801.	0.343781	7.61E-13	postive
RAD52	AC092111.	0.583722	6.70E-39	postive
DNMT3A	STARD7-A	0.342747	9.00E-13	postive
MECP2	STARD7-A	0.316796	4.93E-11	postive
NEIL1	STARD7-A	0.308002	1.76E-10	postive
NSUN3	CASC2	0.317494	4.45E-11	postive
NSUN7	CASC2	0.304729	2.80E-10	postive
DNMT3A	CASC2	0.302668	3.73E-10	postive
TRDMT1	CASC2	0.32242	2.14E-11	postive
TET2	CASC2	0.331435	5.40E-12	postive
NSUN6	AC025178.	0.307985	1.76E-10	postive
NSUN7	AC025178.	0.366752	1.57E-14	postive
RAD52	AC025178.	0.379648	1.54E-15	postive
TET1	AC025178.	0.472269	3.20E-24	postive
TET3	AC025178.	0.33194	4.99E-12	postive
NSUN3	AC022211.	0.32429	1.61E-11	postive
UHRF2	AC022211.	0.30896	1.54E-10	postive
ZBTB33	AC022211.	0.318274	3.97E-11	postive
TET1	AC022211.	0.450605	6.04E-22	postive
TET2	AC022211.	0.605555	1.74E-42	postive
TET3	AC022211.	0.422629	3.09E-19	postive
NSUN6	AC027601.	0.348738	3.38E-13	postive
DNMT3A	AC027601.	0.334109	3.56E-12	postive
RAD52	AC027601.	0.491401	2.26E-26	postive
MBD1	AC027601.	0.372636	5.51E-15	postive
NEIL1	AC027601.	0.484297	1.48E-25	postive
TET3	AC027601.	0.321587	2.42E-11	postive
NTHL1	AC010247.	0.350856	2.38E-13	postive
NSUN6	AL391684.	0.519047	9.98E-30	postive
NSUN7	AL391684.	0.358631	6.41E-14	postive
RAD52	AL391684.	0.484897	1.26E-25	postive
NEIL1	AL391684.	0.523208	2.93E-30	postive
TET1	AL391684.	0.39221	1.45E-16	postive
TET2	AL391684.	0.337509	2.08E-12	postive
NSUN6	AC013403.	0.339509	1.51E-12	postive
NSUN7	AC013403.	0.30108	4.65E-10	postive
DNMT3A	AC013403.	0.403021	1.75E-17	postive
RAD52	AC013403.	0.39089	1.87E-16	postive

MECP2	AC013403.	0.324458	1.57E-11	postive
NEIL1	AC013403.	0.393767	1.08E-16	postive
TET1	AC013403.	0.317765	4.28E-11	postive
TET3	AC013403.	0.318789	3.68E-11	postive
NSUN6	PARD3-AS	0.315971	5.57E-11	postive
NEIL1	AC138150.	0.363519	2.76E-14	postive
NSUN6	AC106782.	0.303236	3.45E-10	postive
NSUN7	AC106782.	0.33195	4.99E-12	postive
RAD52	AC106782.	0.323631	1.78E-11	postive
NEIL1	AC106782.	0.439124	8.38E-21	postive
NSUN3	AC005954.	0.355304	1.13E-13	postive
RAD52	AC005954.	0.331148	5.65E-12	postive
MECP2	AC005954.	0.318628	3.76E-11	postive
NEIL1	AC005954.	0.339978	1.41E-12	postive
UHRF2	AC005954.	0.339351	1.55E-12	postive
ZBTB33	AC005954.	0.347807	3.94E-13	postive
TET1	AC005954.	0.325982	1.25E-11	postive
TET2	AC005954.	0.529707	4.18E-31	postive
TET3	AC005954.	0.348259	3.66E-13	postive
UNG	DDX11-AS	0.305814	2.40E-10	postive
TET1	ABALON	0.40431	1.35E-17	postive
TET2	ABALON	0.305504	2.51E-10	postive
TET3	AC105219.	0.389685	2.35E-16	postive
NSUN5	AC087741.	0.300078	5.34E-10	postive
MBD3	AC087741.	0.37564	3.20E-15	postive
NTHL1	AC087741.	0.321079	2.61E-11	postive
NSUN3	HMGA2-A	0.689692	2.60E-59	postive
TRDMT1	HMGA2-A	0.449308	8.17E-22	postive
ZBTB38	HMGA2-A	0.325277	1.39E-11	postive
TET1	HMGA2-A	0.314894	6.52E-11	postive
NSUN6	AC024361.	0.354314	1.33E-13	postive
NSUN7	AC024361.	0.400666	2.79E-17	postive
RAD52	AC024361.	0.317625	4.37E-11	postive
TET1	AC024361.	0.587123	1.93E-39	postive
TET2	AC024361.	0.348471	3.53E-13	postive
TET3	AC024361.	0.39926	3.69E-17	postive
NSUN3	AC009137.	0.324025	1.68E-11	postive
TET1	AC009137.	0.393518	1.13E-16	postive
TET2	AC009137.	0.387023	3.90E-16	postive
NSUN3	ADAMTSL4	0.314021	7.40E-11	postive
TET1	ADAMTSL4	0.31241	9.35E-11	postive
TET2	ADAMTSL4	0.378536	1.89E-15	postive
NSUN3	PRC1-AS1	0.526066	1.25E-30	postive
TRDMT1	PRC1-AS1	0.314324	7.08E-11	postive
RAD52	PRC1-AS1	0.309124	1.50E-10	postive
NEIL1	PRC1-AS1	0.341772	1.05E-12	postive
UHRF2	PRC1-AS1	0.34231	9.66E-13	postive
TET1	PRC1-AS1	0.495959	6.65E-27	postive
TET2	PRC1-AS1	0.534145	1.08E-31	postive
TET3	PRC1-AS1	0.340014	1.40E-12	postive
DNMT3A	LINC01977	0.344152	7.17E-13	postive
NSUN3	RUFY1-AS	0.436318	1.57E-20	postive
TRDMT1	RUFY1-AS	0.323242	1.89E-11	postive
MECP2	RUFY1-AS	0.350744	2.43E-13	postive
UHRF2	RUFY1-AS	0.320081	3.03E-11	postive
TET1	RUFY1-AS	0.528158	6.67E-31	postive
TET2	RUFY1-AS	0.48037	4.08E-25	postive
TET3	RUFY1-AS	0.326263	1.20E-11	postive

NSUN6	C9orf163	0.304039	3.08E-10	postive
NEIL1	C9orf163	0.394406	9.51E-17	postive
NSUN6	AC018904.	0.381012	1.20E-15	postive
DNMT1	AC018904.	-0.315	6.42E-11	negative
NEIL1	AC018904.	0.485046	1.21E-25	postive
SMUG1	AC018904.	0.300485	5.05E-10	postive
NSUN6	NFYC-AS1	0.36382	2.62E-14	postive
NSUN7	NFYC-AS1	0.366942	1.52E-14	postive
DNMT3A	NFYC-AS1	0.346458	4.92E-13	postive
RAD52	NFYC-AS1	0.414967	1.54E-18	postive
MECP2	NFYC-AS1	0.330948	5.83E-12	postive
NEIL1	NFYC-AS1	0.361169	4.15E-14	postive
ZBTB33	NFYC-AS1	0.327927	9.28E-12	postive
TET1	NFYC-AS1	0.420201	5.17E-19	postive
TET2	NFYC-AS1	0.387132	3.82E-16	postive
TET3	NFYC-AS1	0.380116	1.41E-15	postive
NSUN6	AC004148.	0.570239	8.08E-37	postive
NSUN7	AC004148.	0.386785	4.08E-16	postive
DNMT3A	AC004148.	0.354914	1.21E-13	postive
RAD52	AC004148.	0.559994	2.67E-35	postive
MBD1	AC004148.	0.353349	1.57E-13	postive
NEIL1	AC004148.	0.650757	7.36E-51	postive
NSUN6	AC009087.	0.390222	2.12E-16	postive
RAD52	AC009087.	0.334103	3.56E-12	postive
NEIL1	AC009087.	0.353316	1.58E-13	postive
ZBTB33	AC009087.	0.30416	3.03E-10	postive
TET1	AC009087.	0.423142	2.77E-19	postive
TET2	AC009087.	0.49149	2.21E-26	postive
TET3	AC009087.	0.362407	3.35E-14	postive
NSUN3	AP001793.	0.360672	4.52E-14	postive
NSUN6	AP001793.	0.307705	1.84E-10	postive
NSUN7	AP001793.	0.319212	3.45E-11	postive
RAD52	AP001793.	0.392825	1.29E-16	postive
NEIL1	AP001793.	0.302613	3.76E-10	postive
UHRF2	AP001793.	0.301027	4.69E-10	postive
ZBTB33	AP001793.	0.332726	4.42E-12	postive
TET1	AP001793.	0.394757	8.88E-17	postive
TET2	AP001793.	0.602661	5.40E-42	postive
TET3	AP001793.	0.41468	1.64E-18	postive
NSUN3	GSTCD-AS	0.426287	1.41E-19	postive
TRDMT1	GSTCD-AS	0.304145	3.03E-10	postive
UHRF2	GSTCD-AS	0.351444	2.16E-13	postive
TET1	GSTCD-AS	0.635588	6.81E-48	postive
TET2	GSTCD-AS	0.59816	3.06E-41	postive
TET3	GSTCD-AS	0.339832	1.44E-12	postive
TET1	AC009955.	0.430051	6.25E-20	postive
TET3	AC009955.	0.334341	3.43E-12	postive
NSUN6	AC016737.	0.421871	3.63E-19	postive
NSUN7	AC016737.	0.348454	3.55E-13	postive
RAD52	AC016737.	0.436665	1.45E-20	postive
NEIL1	AC016737.	0.450722	5.88E-22	postive
SMUG1	AC016737.	0.357307	8.04E-14	postive
NSUN7	ZNRD2-AS	0.373434	4.77E-15	postive
DNMT3A	ZNRD2-AS	0.346312	5.04E-13	postive
TET3	ZNRD2-AS	0.350277	2.62E-13	postive
NSUN7	AC022762.	0.361229	4.10E-14	postive
RAD52	AC022762.	0.361202	4.12E-14	postive
NEIL1	AC022762.	0.453006	3.44E-22	postive

NSUN3	AL031429.	0.477109	9.41E-25	postive
TRDMT1	AL031429.	0.316182	5.40E-11	postive
NSUN3	AL450326.	0.377564	2.26E-15	postive
ZBTB38	AL450326.	0.463151	3.04E-23	postive
ZBTB4	AL450326.	0.312551	9.16E-11	postive
NSUN3	KCNIP2-AS	0.301888	4.16E-10	postive
NSUN7	KCNIP2-AS	0.373518	4.70E-15	postive
TET1	KCNIP2-AS	0.537615	3.68E-32	postive
TET2	KCNIP2-AS	0.417281	9.54E-19	postive
TET1	FIRRE	0.498451	3.37E-27	postive
TET2	FIRRE	0.536666	4.95E-32	postive
TET3	FIRRE	0.387717	3.42E-16	postive
NSUN7	RBM26-AS	0.355433	1.10E-13	postive
TET2	RBM26-AS	0.328745	8.18E-12	postive
NSUN3	AC127024.	0.425491	1.68E-19	postive
NEIL1	AC127024.	0.327223	1.03E-11	postive
UHRF2	AC127024.	0.321914	2.31E-11	postive
TET1	AC127024.	0.484436	1.42E-25	postive
TET2	AC127024.	0.57539	1.33E-37	postive
TET3	AC127024.	0.315778	5.73E-11	postive
NSUN6	AL357140.	0.318332	3.93E-11	postive
NSUN7	AL357140.	0.316828	4.91E-11	postive
TET1	AL357140.	0.301446	4.42E-10	postive
NSUN6	AC084018.	0.410762	3.67E-18	postive
NSUN7	AC084018.	0.313969	7.46E-11	postive
DNMT3A	AC084018.	0.331881	5.04E-12	postive
RAD52	AC084018.	0.34533	5.92E-13	postive
MBD1	AC084018.	0.33485	3.17E-12	postive
MECP2	AC084018.	0.336624	2.40E-12	postive
NEIL1	AC084018.	0.424723	1.98E-19	postive
TET3	AC084018.	0.304513	2.88E-10	postive
NSUN3	SNHG4	0.609573	3.56E-43	postive
ZBTB33	SNHG4	0.324407	1.59E-11	postive
TET1	SNHG4	0.304666	2.82E-10	postive
TET2	SNHG4	0.505067	5.42E-28	postive
TET3	SNHG4	0.408275	6.09E-18	postive
MBD3	AC055822.	0.346936	4.55E-13	postive
NEIL1	AC055822.	0.314081	7.34E-11	postive
NEIL1	NARF-AS1	0.339425	1.54E-12	postive
ZBTB33	NARF-AS1	0.334668	3.26E-12	postive
TET1	NARF-AS1	0.316198	5.39E-11	postive
TET2	NARF-AS1	0.549058	9.78E-34	postive
TET3	NARF-AS1	0.377636	2.23E-15	postive
NSUN3	ENTPD1-A	0.760245	1.25E-78	postive
TRDMT1	ENTPD1-A	0.494656	9.45E-27	postive
UHRF2	ENTPD1-A	0.352353	1.85E-13	postive
ZBTB38	ENTPD1-A	0.415954	1.26E-18	postive
TET1	ENTPD1-A	0.650992	6.60E-51	postive
TET2	ENTPD1-A	0.55665	8.14E-35	postive
TET3	ENTPD1-A	0.357132	8.28E-14	postive
NEIL1	AC009690.	0.465257	1.82E-23	postive
TET2	AC009690.	0.544746	3.90E-33	postive
NSUN6	AC099482.	0.334037	3.60E-12	postive
NSUN7	AC099482.	0.324642	1.53E-11	postive
TET1	AC099482.	0.622355	1.94E-45	postive
TET3	AC099482.	0.336722	2.36E-12	postive
NSUN3	AC026356.	0.491597	2.15E-26	postive
TRDMT1	AC026356.	0.474282	1.93E-24	postive

UHRF2	AC026356.	0.444368	2.55E-21	postive
ZBTB38	AC026356.	0.344201	7.11E-13	postive
TET1	AC026356.	0.358989	6.03E-14	postive
TET2	AC026356.	0.302896	3.61E-10	postive
DNMT3A	DLX6-AS1	0.338356	1.82E-12	postive
NSUN6	MAL2-AS1	0.337492	2.09E-12	postive
NSUN7	MAL2-AS1	0.322399	2.15E-11	postive
TET1	MAL2-AS1	0.652907	2.71E-51	postive
TET2	MAL2-AS1	0.411275	3.31E-18	postive
TET3	MAL2-AS1	0.363613	2.72E-14	postive
NSUN6	AL353622.	0.410439	3.92E-18	postive
RAD52	AL353622.	0.358225	6.88E-14	postive
NEIL1	AL353622.	0.538713	2.62E-32	postive
NSUN3	AC011773.	0.344873	6.38E-13	postive
DNMT3A	AC011773.	0.424482	2.08E-19	postive
TET1	AC011773.	0.613657	6.91E-44	postive
TET2	AC011773.	0.423723	2.45E-19	postive
TET3	AC011773.	0.389666	2.36E-16	postive
NTHL1	AP000251.	0.34206	1.01E-12	postive
SMUG1	AP000251.	0.303167	3.48E-10	postive
NSUN3	AL161891.	0.523915	2.38E-30	postive
DNMT1	AL161891.	0.439325	8.01E-21	postive
TRDMT1	AL161891.	0.405265	1.12E-17	postive
TDG	AL161891.	0.404976	1.19E-17	postive
UHRF1	AL161891.	0.336405	2.48E-12	postive
UHRF2	AL161891.	0.338002	1.93E-12	postive
TET1	AL161891.	0.506437	3.70E-28	postive
TET2	AL161891.	0.35752	7.75E-14	postive
TET3	AL161891.	0.362491	3.30E-14	postive
NSUN6	AC006017.	0.358725	6.31E-14	postive
NSUN7	AC006017.	0.327791	9.47E-12	postive
TET1	AC006017.	0.656527	4.94E-52	postive
TET2	AC006017.	0.383392	7.70E-16	postive
TET3	AC006017.	0.365668	1.90E-14	postive
NSUN3	AL049539.	0.31007	1.31E-10	postive
TET1	AL049539.	0.550656	5.82E-34	postive
TET2	AL049539.	0.500709	1.82E-27	postive
DNMT3A	LINC0171ʻ	0.326103	1.23E-11	postive
ZBTB33	LINC0171ʻ	0.320847	2.71E-11	postive
TET1	LINC0171ʻ	0.343846	7.53E-13	postive
TET2	LINC0171ʻ	0.368849	1.08E-14	postive
NSUN6	AC093249.	0.302454	3.84E-10	postive
NEIL1	AC093249.	0.351086	2.29E-13	postive
NTHL1	AC093249.	0.358586	6.46E-14	postive
YBX1	AL603839.	0.309714	1.38E-10	postive
NSUN3	AL110115.	0.379655	1.54E-15	postive
UHRF2	AL110115.	0.33828	1.84E-12	postive
TET1	AL110115.	0.528502	6.01E-31	postive
TET2	AL110115.	0.575227	1.41E-37	postive
TET3	AL110115.	0.311905	1.01E-10	postive
DNMT1	OXCT1-AS	0.346774	4.67E-13	postive
NSUN6	NFE2L1-D	0.33786	1.97E-12	postive
NSUN7	NFE2L1-D	0.381493	1.10E-15	postive
DNMT3A	NFE2L1-D	0.346546	4.85E-13	postive
RAD52	NFE2L1-D	0.31789	4.20E-11	postive
NEIL1	NFE2L1-D	0.336056	2.62E-12	postive
TET1	NFE2L1-D	0.304676	2.82E-10	postive
NEIL1	HEXA-AS1	0.352356	1.85E-13	postive

NSUN3	AC211476.	0.380043	1.43E-15	postive
NSUN6	AC211476.	0.309918	1.34E-10	postive
NSUN7	AC211476.	0.305799	2.40E-10	postive
UHRF2	AC211476.	0.333134	4.15E-12	postive
TET1	AC211476.	0.621876	2.37E-45	postive
TET2	AC211476.	0.572494	3.68E-37	postive
TET3	AC211476.	0.351559	2.12E-13	postive
NSUN3	AL731563.	0.352571	1.79E-13	postive
RAD52	AL731563.	0.311284	1.10E-10	postive
TET1	AL731563.	0.51871	1.10E-29	postive
TET2	AL731563.	0.318718	3.71E-11	postive
TDG	AL139035.	0.335432	2.89E-12	postive
ZBTB33	AL139035.	0.302146	4.01E-10	postive
TET3	AL139035.	0.392973	1.25E-16	postive
NSUN3	AC037487.	0.692329	6.21E-60	postive
TRDMT1	AC037487.	0.428836	8.14E-20	postive
UHRF2	AC037487.	0.337618	2.05E-12	postive
ZBTB38	AC037487.	0.323236	1.89E-11	postive
TET1	AC037487.	0.629392	9.95E-47	postive
TET2	AC037487.	0.561541	1.59E-35	postive
NSUN6	AL158063.	0.393809	1.07E-16	postive
NSUN7	AL158063.	0.342517	9.34E-13	postive
RAD52	AL158063.	0.356196	9.71E-14	postive
NEIL1	AL158063.	0.494234	1.06E-26	postive
TET1	AC012213.	0.317269	4.60E-11	postive
TET3	AC012464.	0.356666	8.96E-14	postive
NSUN6	ARHGAP27	0.413416	2.13E-18	postive
NSUN7	ARHGAP27	0.324724	1.51E-11	postive
RAD52	ARHGAP27	0.346986	4.51E-13	postive
NEIL1	ARHGAP27	0.55578	1.09E-34	postive
RAD52	LINC00685	0.321785	2.35E-11	postive
NEIL1	LINC00685	0.523563	2.64E-30	postive
NSUN6	AC108681.	0.30174	4.25E-10	postive
TET2	AC108681.	0.376611	2.68E-15	postive
TET3	AC108681.	0.32661	1.13E-11	postive
NSUN6	ZNF32-AS	0.544743	3.91E-33	postive
NSUN7	ZNF32-AS	0.370006	8.81E-15	postive
DNMT3A	ZNF32-AS	0.363843	2.61E-14	postive
RAD52	ZNF32-AS	0.467798	9.73E-24	postive
NEIL1	ZNF32-AS	0.567034	2.45E-36	postive
NSUN6	RNF139-A	0.406132	9.40E-18	postive
NSUN7	RNF139-A	0.380052	1.43E-15	postive
DNMT3A	RNF139-A	0.372313	5.84E-15	postive
RAD52	RNF139-A	0.366321	1.69E-14	postive
NEIL1	RNF139-A	0.346409	4.96E-13	postive
TET1	RNF139-A	0.416657	1.09E-18	postive
TET2	RNF139-A	0.308869	1.56E-10	postive
TET3	RNF139-A	0.413139	2.25E-18	postive
NSUN7	PITPNA-AS	0.302068	4.06E-10	postive
NSUN3	AC091729.	0.330976	5.80E-12	postive
UHRF2	AC091729.	0.331891	5.03E-12	postive
TET1	AC091729.	0.591615	3.64E-40	postive
TET2	AC091729.	0.515884	2.50E-29	postive
TET3	AC091729.	0.353992	1.41E-13	postive
NSUN6	SUGT1-DT	0.403688	1.53E-17	postive
RAD52	SUGT1-DT	0.362753	3.15E-14	postive
NEIL1	SUGT1-DT	0.557772	5.61E-35	postive
NSUN6	AL450344.	0.336745	2.35E-12	postive

NSUN7	AL450344.	0.309451	1.43E-10	postive
TET1	AL450344.	0.662015	3.57E-53	postive
TET2	AL450344.	0.382068	9.85E-16	postive
TET3	AL450344.	0.335992	2.65E-12	postive
NSUN3	AC040904.	0.680112	4.13E-57	postive
TRDMT1	AC040904.	0.441488	4.92E-21	postive
UHRF2	AC040904.	0.31089	1.16E-10	postive
TET1	AC040904.	0.502418	1.13E-27	postive
TET2	AC040904.	0.440093	6.74E-21	postive
NSUN6	FABP6-AS.	0.503233	9.04E-28	postive
SMUG1	FABP6-AS.	0.303182	3.47E-10	postive
TET1	FABP6-AS.	0.383209	7.97E-16	postive
NSUN6	WARS2-AS	0.367795	1.30E-14	postive
NSUN7	WARS2-AS	0.325124	1.42E-11	postive
RAD52	WARS2-AS	0.319271	3.42E-11	postive
TET1	WARS2-AS	0.390265	2.11E-16	postive
TET3	WARS2-AS	0.333156	4.13E-12	postive
ALYREF	AL135744.	0.303745	3.21E-10	postive
ZBTB33	PPIC-AS1	0.310226	1.28E-10	postive
ZBTB38	PPIC-AS1	0.339102	1.62E-12	postive
TET2	PPIC-AS1	0.307468	1.90E-10	postive
NSUN6	KRT7-AS	0.473135	2.57E-24	postive
ALYREF	KRT7-AS	-0.30767	1.85E-10	negative
RAD52	KRT7-AS	0.351195	2.25E-13	postive
NEIL1	KRT7-AS	0.612503	1.10E-43	postive
TET2	KRT7-AS	0.305703	2.44E-10	postive
ZBTB33	AC015922.	0.370563	7.98E-15	postive
ZBTB38	AC015922.	0.478905	5.95E-25	postive
ZBTB4	AC015922.	0.437211	1.29E-20	postive
NSUN3	AC012358.	0.559849	2.80E-35	postive
TET2	AC012358.	0.339665	1.48E-12	postive
NSUN6	AC007686.	0.319719	3.20E-11	postive
NSUN7	AC007686.	0.32901	7.86E-12	postive
RAD52	AC007686.	0.315078	6.35E-11	postive
MECP2	AC007686.	0.33974	1.46E-12	postive
TET1	AC007686.	0.47288	2.74E-24	postive
RAD52	AC005261.	0.396519	6.31E-17	postive
NEIL1	AC005261.	0.420032	5.36E-19	postive
TET2	AC005261.	0.329149	7.69E-12	postive
NSUN3	AC087286.	0.581955	1.27E-38	postive
TRDMT1	AC087286.	0.392723	1.32E-16	postive
UHRF2	AC087286.	0.367368	1.41E-14	postive
ZBTB38	AC087286.	0.356405	9.37E-14	postive
TET1	AC087286.	0.597814	3.50E-41	postive
TET2	AC087286.	0.596061	6.82E-41	postive
NSUN3	MED4-AS1	0.308833	1.56E-10	postive
NSUN6	MED4-AS1	0.333977	3.63E-12	postive
NSUN7	MED4-AS1	0.318365	3.91E-11	postive
TET1	MED4-AS1	0.636377	4.82E-48	postive
TET2	MED4-AS1	0.45763	1.15E-22	postive
TET3	MED4-AS1	0.335783	2.74E-12	postive
NTHL1	AL035420.	0.354214	1.36E-13	postive
NSUN3	AF117829.	0.350375	2.58E-13	postive
NSUN6	AF117829.	0.366828	1.55E-14	postive
NSUN7	AF117829.	0.348727	3.39E-13	postive
RAD52	AF117829.	0.320751	2.75E-11	postive
TET1	AF117829.	0.52092	5.76E-30	postive
TET2	AF117829.	0.494983	8.65E-27	postive

TET3	AF117829.	0.395865	7.16E-17	postive
NSUN6	LINC0090C	0.427148	1.17E-19	postive
NSUN6	LINC02886	0.344381	6.91E-13	postive
NSUN7	LINC02886	0.30024	5.23E-10	postive
TET1	LINC02886	0.606827	1.06E-42	postive
RAD52	LINC01748	0.355945	1.01E-13	postive
TET2	LINC01748	0.358706	6.33E-14	postive
TET3	LINC01748	0.308202	1.71E-10	postive
NTHL1	AC136604.	0.312946	8.65E-11	postive
NSUN3	GAS8-AS1	0.394263	9.77E-17	postive
NSUN7	GAS8-AS1	0.306534	2.17E-10	postive
NEIL1	GAS8-AS1	0.367289	1.43E-14	postive
TET1	GAS8-AS1	0.389527	2.42E-16	postive
TET2	GAS8-AS1	0.575062	1.49E-37	postive
TET3	GAS8-AS1	0.335836	2.71E-12	postive
RAD52	AC112484.	0.35679	8.78E-14	postive
TET3	AC112484.	0.313201	8.34E-11	postive
DNMT3A	AC012360.	0.308819	1.57E-10	postive
TET1	AC012360.	0.451903	4.46E-22	postive
TET3	AC012360.	0.306197	2.27E-10	postive
NEIL1	AC078909.	0.44515	2.13E-21	postive
NSUN5	EMSLR	0.319641	3.24E-11	postive
NSUN6	GEMIN7-A	0.452303	4.06E-22	postive
NSUN7	GEMIN7-A	0.327915	9.30E-12	postive
RAD52	GEMIN7-A	0.401932	2.18E-17	postive
NEIL1	GEMIN7-A	0.615873	2.81E-44	postive
TET2	GEMIN7-A	0.307682	1.84E-10	postive
NSUN6	AP000866.	0.45724	1.26E-22	postive
NSUN7	AP000866.	0.459365	7.59E-23	postive
RAD52	AP000866.	0.36014	4.95E-14	postive
MBD1	AP000866.	0.377518	2.28E-15	postive
MECP2	AP000866.	0.305318	2.57E-10	postive
NEIL1	AP000866.	0.364102	2.49E-14	postive
ZBTB33	AP000866.	0.369664	9.37E-15	postive
ZBTB38	AP000866.	0.305845	2.39E-10	postive
TET1	AP000866.	0.384639	6.10E-16	postive
TET2	AP000866.	0.534505	9.65E-32	postive
TET3	AP000866.	0.443581	3.06E-21	postive
NSUN6	AL354993.	0.394374	9.57E-17	postive
NSUN7	AL354993.	0.477934	7.63E-25	postive
DNMT3A	AL354993.	0.330822	5.94E-12	postive
RAD52	AL354993.	0.321553	2.44E-11	postive
ZBTB33	AL354993.	0.407717	6.82E-18	postive
TET1	AL354993.	0.526452	1.11E-30	postive
TET2	AL354993.	0.5206	6.33E-30	postive
TET3	AL354993.	0.503276	8.93E-28	postive
NEIL1	AC104187.	0.313491	8.00E-11	postive
NTHL1	AC104187.	0.336051	2.62E-12	postive
SMUG1	AC104187.	0.335139	3.03E-12	postive
NSUN5	CAMTA1-I	0.317368	4.53E-11	postive
NTHL1	CAMTA1-I	0.425142	1.81E-19	postive
RAD52	AC007098.	0.345849	5.44E-13	postive
NSUN3	AL353804.	0.614558	4.80E-44	postive
TRDMT1	AL353804.	0.440509	6.13E-21	postive
UHRF2	AL353804.	0.348227	3.68E-13	postive
ZBTB38	AL353804.	0.340292	1.34E-12	postive
TET1	AL353804.	0.646837	4.46E-50	postive
TET2	AL353804.	0.591671	3.57E-40	postive

TET3	AL353804.	0.335675	2.78E-12	postive
NSUN3	AC144548.	0.418296	7.72E-19	postive
NSUN4	AC144548.	0.319051	3.54E-11	postive
NSUN7	AC144548.	0.316736	4.98E-11	postive
RAD52	AC144548.	0.364795	2.21E-14	postive
MBD1	AC144548.	0.310852	1.17E-10	postive
NEIL1	AC144548.	0.359609	5.42E-14	postive
TDG	AC144548.	0.373654	4.59E-15	postive
ZBTB33	AC144548.	0.35254	1.80E-13	postive
TET1	AC144548.	0.33145	5.39E-12	postive
TET2	AC144548.	0.564933	5.02E-36	postive
TET3	AC144548.	0.412547	2.55E-18	postive
NSUN6	AL049780.	0.376316	2.83E-15	postive
RAD52	AL049780.	0.355952	1.01E-13	postive
NEIL1	AL049780.	0.404495	1.30E-17	postive
TET1	AL049780.	0.402572	1.92E-17	postive
TET2	AL049780.	0.513041	5.68E-29	postive
TET3	AL049780.	0.323804	1.74E-11	postive
NSUN6	INTS6-AS1	0.315354	6.09E-11	postive
RAD52	INTS6-AS1	0.371098	7.25E-15	postive
NEIL1	INTS6-AS1	0.456413	1.54E-22	postive
NSUN6	LNCTAM34	0.420903	4.46E-19	postive
NEIL1	LNCTAM34	0.3328	4.37E-12	postive
SMUG1	LNCTAM34	0.306976	2.04E-10	postive
NSUN3	AC008750.	0.600009	1.51E-41	postive
NSUN6	AC008750.	0.305596	2.48E-10	postive
TRDMT1	AC008750.	0.405896	9.85E-18	postive
MECP2	AC008750.	0.3019	4.15E-10	postive
TET1	AC008750.	0.601248	9.34E-42	postive
TET2	AC008750.	0.520883	5.82E-30	postive
TET3	AC008750.	0.351918	1.99E-13	postive
NSUN3	AC009268.	0.362599	3.24E-14	postive
TET1	AC009268.	0.480448	4.00E-25	postive
NSUN6	PSPC1-AS	0.415776	1.31E-18	postive
NSUN7	PSPC1-AS	0.415288	1.45E-18	postive
RAD52	PSPC1-AS	0.362174	3.49E-14	postive
NEIL1	PSPC1-AS	0.45401	2.72E-22	postive
TET1	PSPC1-AS	0.533188	1.45E-31	postive
TET2	PSPC1-AS	0.526917	9.69E-31	postive
TET3	PSPC1-AS	0.356602	9.06E-14	postive
DNMT1	AC145207.	0.380851	1.23E-15	postive
DNMT3A	AC145207.	0.402213	2.06E-17	postive
ALYREF	AC145207.	0.382262	9.50E-16	postive
TET1	AC145207.	0.381913	1.01E-15	postive
TET3	AC145207.	0.390553	1.99E-16	postive
NSUN3	SOCAR	0.452607	3.78E-22	postive
TRDMT1	SOCAR	0.388136	3.16E-16	postive
TET1	SOCAR	0.359705	5.34E-14	postive
NSUN6	KDM4A-AS	0.39219	1.46E-16	postive
NSUN7	KDM4A-AS	0.370941	7.46E-15	postive
DNMT3A	KDM4A-AS	0.386793	4.07E-16	postive
RAD52	KDM4A-AS	0.358817	6.21E-14	postive
TET1	KDM4A-AS	0.541091	1.24E-32	postive
TET2	KDM4A-AS	0.36589	1.82E-14	postive
TET3	KDM4A-AS	0.447914	1.13E-21	postive
NEIL1	AC010999.	0.357453	7.84E-14	postive
TET1	AC010999.	0.379322	1.64E-15	postive
NSUN6	AL008582.	0.420501	4.85E-19	postive

RAD52	AL008582.	0.428163	9.42E-20	postive
NEIL1	AL008582.	0.496476	5.78E-27	postive
TET1	AL078644.	0.324656	1.53E-11	postive
NSUN3	AC011405.	0.441795	4.59E-21	postive
UHRF2	AC011405.	0.321746	2.37E-11	postive
TET1	AC011405.	0.602979	4.77E-42	postive
TET2	AC011405.	0.568552	1.45E-36	postive
TET3	AC011405.	0.357565	7.69E-14	postive
RAD52	LINC01337	0.305027	2.68E-10	postive
NEIL1	LINC01337	0.406937	7.99E-18	postive
DNMT3A	AC092969.	0.314729	6.68E-11	postive
MBD3	AL022341.	0.316947	4.82E-11	postive
NTHL1	AL022341.	0.421491	3.93E-19	postive
NSUN5	AL117332.	0.319293	3.41E-11	postive
ALYREF	AL117332.	0.313469	8.02E-11	postive
MBD3	AL117332.	0.423579	2.52E-19	postive
NSUN3	AC018752.	0.592008	3.15E-40	postive
TRDMT1	AC018752.	0.484634	1.35E-25	postive
UHRF2	AC018752.	0.343223	8.33E-13	postive
ZBTB33	AC018752.	0.349946	2.77E-13	postive
ZBTB38	AC018752.	0.413298	2.18E-18	postive
TET1	AC018752.	0.644879	1.09E-49	postive
TET2	AC018752.	0.608516	5.42E-43	postive
TET3	AC018752.	0.347484	4.16E-13	postive
NSUN3	AC005740.	0.540645	1.43E-32	postive
TRDMT1	AC005740.	0.345219	6.03E-13	postive
NEIL1	AC005740.	0.321308	2.53E-11	postive
TET1	AC005740.	0.375082	3.54E-15	postive
TET2	AC005740.	0.327484	9.93E-12	postive
MBD3	AC011446.	0.354624	1.27E-13	postive
NTHL1	AC011446.	0.412193	2.74E-18	postive
SMUG1	AC011446.	0.349152	3.16E-13	postive
NSUN3	AC092756.	0.35867	6.37E-14	postive
UHRF2	AC092756.	0.309038	1.52E-10	postive
TET1	AC092756.	0.539476	2.06E-32	postive
TET2	AC092756.	0.548911	1.03E-33	postive
DNMT1	AL449423.	0.457832	1.10E-22	postive
UHRF1	AL449423.	0.507982	2.39E-28	postive
UHRF2	AL449423.	0.354045	1.40E-13	postive
NSUN3	STARD13-	0.741951	5.12E-73	postive
TRDMT1	STARD13-	0.479376	5.27E-25	postive
UHRF2	STARD13-	0.312745	8.91E-11	postive
ZBTB38	STARD13-	0.300058	5.36E-10	postive
TET1	STARD13-	0.496266	6.11E-27	postive
TET2	STARD13-	0.457697	1.13E-22	postive
NSUN6	AL513320.	0.324189	1.64E-11	postive
RAD52	AL513320.	0.400365	2.97E-17	postive
NEIL1	AL513320.	0.562615	1.10E-35	postive
NSUN7	LINC00471	0.31244	9.31E-11	postive
DNMT3A	LINC00471	0.360181	4.92E-14	postive
TET1	LINC00471	0.407368	7.32E-18	postive
TET3	LINC00471	0.307824	1.81E-10	postive
NSUN6	AC009148.	0.399455	3.55E-17	postive
NSUN7	AC009148.	0.322301	2.18E-11	postive
RAD52	AC009148.	0.434805	2.20E-20	postive
NEIL1	AC009148.	0.519516	8.70E-30	postive
SMUG1	AC009148.	0.3158	5.71E-11	postive
NSUN3	GNG12-AS	0.77429	2.71E-83	postive

TRDMT1	GNG12-AS	0.483246	1.94E-25	postive
UHRF2	GNG12-AS	0.307188	1.98E-10	postive
ZBTB38	GNG12-AS	0.400281	3.02E-17	postive
TET1	GNG12-AS	0.532599	1.73E-31	postive
TET2	GNG12-AS	0.566758	2.69E-36	postive
DNMT3A	AC073073.	0.340608	1.27E-12	postive
ZBTB33	AC073073.	0.357622	7.62E-14	postive
TET3	AC073073.	0.348833	3.33E-13	postive
NSUN6	RPP38-DT	0.462767	3.34E-23	postive
NSUN7	RPP38-DT	0.433414	2.99E-20	postive
DNMT3A	RPP38-DT	0.381039	1.19E-15	postive
MBD1	RPP38-DT	0.329216	7.61E-12	postive
TET1	RPP38-DT	0.419074	6.55E-19	postive
TET3	RPP38-DT	0.320119	3.02E-11	postive
NSUN6	AC008735.	0.380635	1.28E-15	postive
DNMT3A	AC008735.	0.3956	7.54E-17	postive
RAD52	AC008735.	0.485298	1.14E-25	postive
MBD1	AC008735.	0.37043	8.17E-15	postive
NEIL1	AC008735.	0.393915	1.05E-16	postive
TET3	AC008735.	0.34386	7.52E-13	postive
NEIL1	MIF-AS1	0.342802	8.92E-13	postive
TET2	MIF-AS1	0.468848	7.50E-24	postive
NSUN6	AC253536.	0.351727	2.06E-13	postive
NSUN7	AC253536.	0.30448	2.90E-10	postive
NEIL1	AC253536.	0.401789	2.24E-17	postive
TET1	AC253536.	0.402056	2.12E-17	postive
TET2	AC253536.	0.504598	6.18E-28	postive
TET3	AC253536.	0.311216	1.11E-10	postive
NSUN3	AC093788.	0.308636	1.61E-10	postive
NSUN6	AC093788.	0.430155	6.11E-20	postive
RAD52	AC093788.	0.484804	1.29E-25	postive
NEIL1	AC093788.	0.48237	2.44E-25	postive
TET2	AC093788.	0.321137	2.59E-11	postive
NSUN7	AL008635.	0.353799	1.46E-13	postive
NEIL1	AL008635.	0.371675	6.54E-15	postive
NSUN6	ZNF252P-/	0.528789	5.51E-31	postive
NSUN7	ZNF252P-/	0.415604	1.35E-18	postive
DNMT3A	ZNF252P-/	0.300655	4.93E-10	postive
RAD52	ZNF252P-/	0.402144	2.09E-17	postive
NEIL1	ZNF252P-/	0.405035	1.17E-17	postive
TET1	ZNF252P-/	0.468587	8.00E-24	postive
TET2	ZNF252P-/	0.311278	1.10E-10	postive
TET3	ZNF252P-/	0.31258	9.13E-11	postive
MBD4	AC083799.	0.331604	5.26E-12	postive
NSUN7	AL109614.	0.312919	8.69E-11	postive
ZBTB33	AL109614.	0.306176	2.28E-10	postive
TET1	AL109614.	0.498587	3.25E-27	postive
TET2	AL109614.	0.592391	2.72E-40	postive
TET3	AL109614.	0.373632	4.60E-15	postive
NSUN7	AC130371.	0.323685	1.77E-11	postive
NSUN6	AL159169.	0.475372	1.46E-24	postive
NSUN7	AL159169.	0.305377	2.55E-10	postive
RAD52	AL159169.	0.355515	1.09E-13	postive
NEIL1	AL159169.	0.426914	1.23E-19	postive
NSUN6	AC022167.	0.310037	1.32E-10	postive
NSUN7	AC022167.	0.30931	1.46E-10	postive
DNMT3A	AC022167.	0.321455	2.47E-11	postive
RAD52	AC022167.	0.316031	5.52E-11	postive

TET3	AC022167.	0.37201	6.16E-15	postive
NSUN6	AC002128.	0.323511	1.82E-11	postive
NSUN7	AC002128.	0.337393	2.12E-12	postive
NEIL1	AC002128.	0.310368	1.26E-10	postive
ZBTB33	AC002128.	0.352321	1.86E-13	postive
TET1	AC002128.	0.464097	2.41E-23	postive
TET2	AC002128.	0.569718	9.68E-37	postive
TET3	AC002128.	0.472779	2.81E-24	postive
NEIL1	AL157762.	0.391073	1.81E-16	postive
TET1	AC004083.	0.464278	2.31E-23	postive
TET3	AC004083.	0.334696	3.25E-12	postive
DNMT1	MIR924HG	0.473954	2.09E-24	postive
TRDMT1	MIR924HG	0.306621	2.14E-10	postive
ALYREF	MIR924HG	0.332638	4.48E-12	postive
UHRF1	MIR924HG	0.392796	1.30E-16	postive
ALYREF	AC091153.	0.319156	3.48E-11	postive
NSUN5	ATP2A1-A	0.4175	9.12E-19	postive
ALYREF	ATP2A1-A	0.355208	1.15E-13	postive
MBD3	ATP2A1-A	0.379126	1.69E-15	postive
NTHL1	ATP2A1-A	0.448286	1.04E-21	postive
NTHL1	AL133353.	0.321186	2.57E-11	postive
SMUG1	AL133353.	0.413319	2.17E-18	postive
NSUN3	GTF3C2-A	0.3442	7.11E-13	postive
NSUN6	GTF3C2-A	0.375786	3.12E-15	postive
NSUN7	GTF3C2-A	0.330599	6.15E-12	postive
RAD52	GTF3C2-A	0.319081	3.52E-11	postive
ZBTB33	GTF3C2-A	0.318761	3.69E-11	postive
TET1	GTF3C2-A	0.683783	6.05E-58	postive
TET2	GTF3C2-A	0.552813	2.88E-34	postive
TET3	GTF3C2-A	0.496211	6.21E-27	postive
NEIL1	U91328.3	0.309156	1.49E-10	postive
NSUN7	OLMALINC	0.305643	2.46E-10	postive
MBD1	OLMALINC	0.344408	6.88E-13	postive
NSUN5	HK2-DT	0.315349	6.10E-11	postive
NSUN6	MED8-AS1	0.323241	1.89E-11	postive
DNMT3A	MED8-AS1	0.301074	4.66E-10	postive
RAD52	MED8-AS1	0.357753	7.45E-14	postive
NEIL1	MED8-AS1	0.343447	8.04E-13	postive
TET3	MED8-AS1	0.394031	1.02E-16	postive
NSUN6	CTBP1-AS	0.436577	1.48E-20	postive
RAD52	CTBP1-AS	0.397388	5.32E-17	postive
MBD1	CTBP1-AS	0.42697	1.22E-19	postive
NEIL1	CTBP1-AS	0.418757	7.00E-19	postive
TET2	CTBP1-AS	0.38534	5.35E-16	postive
NSUN3	AC004253.	0.326784	1.11E-11	postive
NSUN6	AC004253.	0.360833	4.39E-14	postive
RAD52	AC004253.	0.457746	1.12E-22	postive
NEIL1	AC004253.	0.494199	1.07E-26	postive
TET1	AC004253.	0.355044	1.18E-13	postive
TET2	AC004253.	0.399234	3.71E-17	postive
TET3	AC004253.	0.305887	2.38E-10	postive
NSUN6	LINC01004	0.45403	2.70E-22	postive
NSUN7	LINC01004	0.38652	4.28E-16	postive
RAD52	LINC01004	0.420762	4.59E-19	postive
NEIL1	LINC01004	0.580791	1.93E-38	postive
TET2	LINC01004	0.414086	1.85E-18	postive
NSUN6	AATBC	0.455536	1.89E-22	postive
MBD1	AATBC	0.314531	6.87E-11	postive

NEIL1	AATBC	0.321448	2.47E-11	postive
TET3	AATBC	0.331087	5.70E-12	postive
NSUN3	AC234772.	0.71352	3.53E-65	postive
TRDMT1	AC234772.	0.436822	1.40E-20	postive
TET1	AC234772.	0.355118	1.17E-13	postive
TET2	AC234772.	0.415632	1.35E-18	postive
NSUN6	AC007128.	0.355106	1.17E-13	postive
NSUN7	AC007128.	0.306691	2.12E-10	postive
DNMT3A	AC007128.	0.33729	2.16E-12	postive
RAD52	AC007128.	0.303864	3.16E-10	postive
TDG	AC007128.	0.332508	4.57E-12	postive
ZBTB33	AC007128.	0.397876	4.84E-17	postive
TET1	AC007128.	0.542751	7.35E-33	postive
TET2	AC007128.	0.440748	5.81E-21	postive
TET3	AC007128.	0.54554	3.03E-33	postive
NSUN5	LINC01816	0.310889	1.16E-10	postive
DNMT3A	LINC01816	0.43054	5.61E-20	postive
NSUN6	LINC00115	0.337768	2.00E-12	postive
RAD52	LINC00115	0.409979	4.31E-18	postive
NEIL1	LINC00115	0.479235	5.47E-25	postive
NSUN6	AC091906.	0.300869	4.79E-10	postive
NSUN7	AC091906.	0.3102	1.29E-10	postive
TET1	AC091906.	0.629897	8.01E-47	postive
TET2	AC091906.	0.503194	9.14E-28	postive
TET3	AC091906.	0.36603	1.78E-14	postive
NSUN6	AC025682.	0.345938	5.36E-13	postive
NSUN7	AC025682.	0.420451	4.90E-19	postive
DNMT3A	AC025682.	0.418555	7.31E-19	postive
RAD52	AC025682.	0.362217	3.46E-14	postive
TET1	AC025682.	0.525105	1.67E-30	postive
TET2	AC025682.	0.412432	2.61E-18	postive
TET3	AC025682.	0.423013	2.85E-19	postive
NSUN6	AC018413.	0.351132	2.27E-13	postive
NEIL1	AC018413.	0.361416	3.97E-14	postive
NSUN5	YTHDF3-A	0.334759	3.22E-12	postive
MBD3	YTHDF3-A	0.360935	4.32E-14	postive
NTHL1	YTHDF3-A	0.467825	9.66E-24	postive
NSUN3	AC019186.	0.301986	4.10E-10	postive
TET1	AC019186.	0.529575	4.35E-31	postive
TET2	AC019186.	0.340463	1.30E-12	postive
NSUN3	AC114980.	0.445961	1.77E-21	postive
TET1	AC114980.	0.502277	1.18E-27	postive
TET2	AC114980.	0.484464	1.41E-25	postive
NSUN3	GSN-AS1	0.642549	3.11E-49	postive
TRDMT1	GSN-AS1	0.425169	1.80E-19	postive
UHRF2	GSN-AS1	0.356646	9.00E-14	postive
ZBTB38	GSN-AS1	0.334833	3.18E-12	postive
TET1	GSN-AS1	0.650001	1.04E-50	postive
TET2	GSN-AS1	0.584356	5.32E-39	postive
TET3	GSN-AS1	0.317288	4.59E-11	postive
NSUN3	AC115102.	0.459154	7.98E-23	postive
TET1	AC115102.	0.530637	3.15E-31	postive
TET2	AC115102.	0.502965	9.74E-28	postive
TET3	AC115102.	0.301043	4.68E-10	postive
ZBTB33	AC010595.	0.308879	1.55E-10	postive
TET1	AC010595.	0.41616	1.21E-18	postive
TET2	AC010595.	0.332152	4.83E-12	postive
TET3	AC010595.	0.301055	4.67E-10	postive

TRDMT1	WAKMAR2	0.332067	4.90E-12	postive
UHRF2	WAKMAR2	0.343038	8.59E-13	postive
TET1	WAKMAR2	0.30586	2.38E-10	postive
NSUN7	ITGA9-AS1	0.331619	5.25E-12	postive
NSUN3	AL590369.	0.314164	7.25E-11	postive
NSUN6	AL590369.	0.317682	4.33E-11	postive
NSUN7	AL590369.	0.305554	2.49E-10	postive
DNMT3A	AL590369.	0.355222	1.14E-13	postive
ZBTB33	AL590369.	0.331203	5.60E-12	postive
TET1	AL590369.	0.406876	8.09E-18	postive
TET2	AL590369.	0.40005	3.16E-17	postive
TET3	AL590369.	0.442675	3.76E-21	postive
MBD3	AC011445.	0.317035	4.76E-11	postive
NTHL1	AC011445.	0.408321	6.04E-18	postive
NSUN3	AC211433.	0.339219	1.59E-12	postive
UHRF2	AC211433.	0.303487	3.33E-10	postive
TET1	AC211433.	0.653705	1.86E-51	postive
TET2	AC211433.	0.541546	1.08E-32	postive
TET3	AC211433.	0.359433	5.59E-14	postive
ZBTB33	SNHG16	0.333694	3.80E-12	postive
TET1	SNHG16	0.461681	4.34E-23	postive
TET2	SNHG16	0.457089	1.31E-22	postive
TET3	SNHG16	0.363795	2.63E-14	postive
NSUN3	AC006064.	0.394124	1.00E-16	postive
TRDMT1	AC006064.	0.300139	5.30E-10	postive
RAD52	AC006064.	0.321973	2.29E-11	postive
UHRF2	AC006064.	0.370408	8.21E-15	postive
ZBTB33	AC006064.	0.344377	6.91E-13	postive
TET1	AC006064.	0.62303	1.47E-45	postive
TET2	AC006064.	0.520824	5.93E-30	postive
TET3	AC006064.	0.398274	4.48E-17	postive
NSUN3	AL008718.	0.380686	1.27E-15	postive
UHRF2	AL008718.	0.315063	6.36E-11	postive
TET1	AL008718.	0.636917	3.80E-48	postive
TET2	AL008718.	0.553288	2.47E-34	postive
TET3	AL008718.	0.331054	5.73E-12	postive
MBD3	AL021707.	0.337171	2.20E-12	postive
NEIL1	AL021707.	0.354347	1.33E-13	postive
ZBTB38	AF111167.	0.305383	2.55E-10	postive
NSUN7	AC009961.	0.312125	9.75E-11	postive
TET1	AC009961.	0.387049	3.88E-16	postive
NSUN3	OPA1-AS1	0.472058	3.37E-24	postive
TRDMT1	OPA1-AS1	0.325232	1.40E-11	postive
UHRF2	OPA1-AS1	0.342039	1.01E-12	postive
ZBTB33	OPA1-AS1	0.302114	4.03E-10	postive
TET1	OPA1-AS1	0.675906	3.60E-56	postive
TET2	OPA1-AS1	0.629211	1.07E-46	postive
TET3	OPA1-AS1	0.376664	2.66E-15	postive
NSUN3	AC040934.	0.33645	2.46E-12	postive
NSUN6	AC040934.	0.343744	7.66E-13	postive
NSUN7	AC040934.	0.363491	2.77E-14	postive
RAD52	AC040934.	0.334904	3.14E-12	postive
MECP2	AC040934.	0.301177	4.59E-10	postive
NEIL1	AC040934.	0.34877	3.36E-13	postive
UHRF2	AC040934.	0.311794	1.02E-10	postive
TET1	AC040934.	0.552674	3.02E-34	postive
TET2	AC040934.	0.593441	1.84E-40	postive
TET3	AC040934.	0.360548	4.62E-14	postive

MECP2	Z97989.1	0.328255	8.82E-12	postive
TET1	Z97989.1	0.501558	1.44E-27	postive
TET1	AL132656.	0.43546	1.90E-20	postive
TET2	AL132656.	0.310299	1.27E-10	postive
NSUN3	AC068790.	0.452044	4.31E-22	postive
NSUN6	AC068790.	0.308633	1.61E-10	postive
RAD52	AC068790.	0.306962	2.04E-10	postive
TET1	AC068790.	0.629123	1.12E-46	postive
TET2	AC068790.	0.585208	3.90E-39	postive
TET3	AC068790.	0.362384	3.36E-14	postive
