# Supplementary material for: 5-Methylcytosine-Related Long Noncoding RNAs Are Potential Biomarkers to Predict Overall Survival and Regulate Tumor-Immune Environment in Patients with Bladder Cancer
Source: Dis Markers. 2022 Mar 4;2022:3117359. doi: 10.1155/2022/3117359 (PMC8966750; doi:10.1155/2022/3117359)
Supplement: Supplementary 2 — Table s2: univariate Cox analysis in the training set. [file 3117359.f2.pdf]

lncRNA	HR	HR.95L	HR.95H	pvalue
TMEM147-	0.651313	0.470739	0.901156	0.009647
AC009065.	0.601319	0.381205	0.94853	0.028728
LINC00106	0.650795	0.484057	0.874967	0.004449
AC011461.	0.401628	0.211031	0.764368	0.005463
PDXDC2P-	0.616028	0.416039	0.91215	0.015559
AP003352.	0.523749	0.354875	0.772986	0.001128
AC105137.	0.317826	0.126157	0.800695	0.015038
AC108134.	0.394499	0.210053	0.740905	0.003821
GLYCTK-A	0.113815	0.015582	0.831351	0.032192
AL031282.	0.490673	0.251524	0.957203	0.036775
AC092119.	0.515543	0.285428	0.931182	0.028068
RPARP-AS	0.541552	0.361446	0.811403	0.002949
AL121583.	0.462831	0.235109	0.911118	0.025793
ZNF32-AS	0.398779	0.236333	0.672885	0.000573
AC010542.	0.606203	0.427472	0.859663	0.004979
AL023284.	0.795394	0.657784	0.961793	0.018181
KMT2E-AS	0.721835	0.524826	0.992796	0.045025
MIOS-DT	0.603239	0.420475	0.865443	0.006056
CERS3-AS	2.307629	1.286277	4.139975	0.005044
AC007785.	0.233025	0.0648	0.837975	0.025703
THUMPD3	0.53253	0.372399	0.761516	0.000555
AL591043.	0.267121	0.10238	0.696951	0.006979
AC011503.	0.601929	0.425148	0.852217	0.004218
BDNF-AS	0.313052	0.136641	0.71722	0.006037
AL390719.	0.738395	0.632819	0.861585	0.000117
LINC02626	0.159299	0.034128	0.743565	0.019444
IQCH-AS1	0.492713	0.277512	0.874796	0.015663
PAXIP1-DT	0.741702	0.571967	0.961809	0.024218
AC011462.	0.620036	0.424476	0.905693	0.013424
C1orf220	0.40687	0.206588	0.801322	0.00931
AL133255.	0.163415	0.043645	0.611854	0.00716
AC126178.	0.621624	0.433981	0.890399	0.00951
AC061975.	0.241237	0.059836	0.972584	0.045604
AC008870.	0.547112	0.325275	0.920241	0.02301
AL358072.	0.43906	0.209692	0.919318	0.02903
AC080112.	0.66731	0.533857	0.834123	0.000381
AL078587.	0.468012	0.252207	0.868475	0.016083
AC113139.	0.349155	0.173352	0.703246	0.003225
AC103706.	0.689454	0.52175	0.911062	0.008923
AL122035.	1.587951	1.005287	2.508327	0.047416
AC003102.	0.633869	0.453307	0.886354	0.007694
RAP2C-AS	6.734163	2.314441	19.59391	0.000465
AL022328.	0.63568	0.424363	0.952225	0.027992
AC011815.	0.143056	0.02109	0.970363	0.046507
AL365330.	0.655166	0.455149	0.943083	0.02289
IGBP1-AS1	0.313982	0.141099	0.698693	0.004532
AC089999.	0.414906	0.20742	0.829945	0.012886
AC099850.	1.263914	1.034905	1.5436	0.021656
AL133355.	0.615302	0.393542	0.962024	0.033192
AL353801.	0.211186	0.071661	0.62237	0.004803
RAB11B-A	0.667506	0.465641	0.956882	0.027819
AP005329.	0.32317	0.127	0.822354	0.017769
TTC23L-AS	0.572936	0.390785	0.839991	0.004329
GHRLOS	0.571237	0.362122	0.901111	0.016054
AC022007.	0.604526	0.408077	0.895546	0.012067
ASB16-AS	0.613853	0.392275	0.960589	0.032683
AC092794.	0.296348	0.135635	0.647491	0.002289

MANEA-D	0.257406	0.085698	0.773158	0.015587
AC009065.	0.400049	0.242506	0.659939	0.000334
AC099778.	0.389577	0.201702	0.752447	0.005003
TGFB2-AS	1.595262	1.093089	2.328139	0.015459
AC084125.	0.437754	0.246953	0.775973	0.004679
AC104564.	0.404307	0.240173	0.680609	0.000655
KCNQ1OT	4.243556	1.783435	10.09724	0.001083
AL031775.	0.53375	0.323597	0.880381	0.013935
LINC0225€	3.804563	1.504907	9.618334	0.004747
AC005840.	0.477782	0.307481	0.742407	0.001022
AC027682.	0.27138	0.09367	0.786237	0.016257
AC010487.	0.673512	0.520699	0.871174	0.002609
Z84485.1	0.36738	0.175412	0.769435	0.007934
MCCC1-A:	0.668972	0.449098	0.996495	0.048015
AC005332.	0.612889	0.397752	0.944388	0.026463
AC005387.	0.482364	0.281097	0.827739	0.008141
AC100860.	0.682773	0.472999	0.985579	0.041598
CEBPA-DT	0.70627	0.550049	0.906861	0.006402
AC125257.	0.57319	0.36318	0.904639	0.016829
DOCK8-A:	0.358822	0.193303	0.66607	0.001164
KLF3-AS1	0.321535	0.131566	0.785799	0.012822
AL022322.	0.664487	0.493061	0.895513	0.007256
AC109449.	0.12171	0.030919	0.479105	0.002591
LINC01341	0.644251	0.482304	0.860577	0.002916
AC073896.	0.692142	0.507927	0.943167	0.019777
LINC0280:	0.476811	0.272519	0.834249	0.009462
AP002807.	0.685903	0.486009	0.968012	0.031959
GATA3-A:	0.781651	0.675754	0.904142	0.000911
AL136304.	0.550551	0.32514	0.932233	0.026343
TONSL-A:	0.639092	0.409263	0.997986	0.048973
AC005785.	0.540217	0.310376	0.940262	0.02942
AC015849.	0.709871	0.516917	0.974851	0.034231
U73166.1	0.473259	0.229772	0.974766	0.042429
PCAT7	0.626864	0.477072	0.823687	0.000802
RBMS3-A:	2.237815	1.201409	4.168285	0.011144
AC073957.	0.58424	0.375128	0.90992	0.017427
AC027243.	0.248264	0.068817	0.895644	0.03331
AC142472.	0.517399	0.287295	0.931802	0.028143
AC023302.	0.488063	0.25605	0.930309	0.029298
HDAC2-A:	0.079089	0.015201	0.411498	0.002568
AC011466.	0.186973	0.039361	0.888167	0.034933
AC024451.	0.257318	0.104273	0.634994	0.003226
AC006001.	0.596072	0.378228	0.939387	0.025788
LINC0260	0.569219	0.411078	0.788197	0.000691
AL021878.	0.484307	0.265525	0.883357	0.018058
AC010326.	0.604711	0.43241	0.845668	0.003286
GAS6-DT	1.433486	1.031557	1.99202	0.03195
MIR4435-:	1.409367	1.126304	1.763571	0.002702
SPINT1-A:	0.75895	0.63254	0.910622	0.003006
AC079160.	0.31052	0.139531	0.691049	0.004165
AC068196.	0.035228	0.004087	0.303616	0.00233
AL139123.	0.414318	0.225655	0.760715	0.004481
CAPN10-D	0.400421	0.180791	0.886866	0.024077
SLBP-DT	0.618445	0.420972	0.90855	0.014339
AC010491.	0.303351	0.112246	0.819819	0.018691
AL513218.	0.563799	0.33823	0.939802	0.027941
AL451085.	0.120346	0.024341	0.595	0.009413
AL512770.	0.267574	0.105876	0.676224	0.005319

C21orf62-.	0.230463	0.071439	0.743474	0.014049
AC034236.	0.449237	0.258225	0.781542	0.00462
AC004803.	2.724769	1.205661	6.157922	0.015972
AC010331.	0.556632	0.355801	0.870822	0.010296
AC245884.	0.521663	0.332638	0.818102	0.00459
AC009061.	0.509205	0.279111	0.928985	0.0278
AL390066.	0.353398	0.14537	0.85912	0.021733
AC016888.	1.295788	1.007676	1.666276	0.043428
AC002059.	0.717897	0.540357	0.953768	0.022223
AL445228.	1.868579	1.031619	3.384573	0.039144
AL731569.	0.325546	0.130028	0.815055	0.016544
AC020978.	0.122964	0.018438	0.820066	0.030398
AC008763.	0.63399	0.42152	0.953557	0.028646
AC104825.	0.619923	0.475946	0.807453	0.000391
AC010998.	0.2263	0.054638	0.937298	0.040436
AC022001.	2.33357	1.044156	5.215267	0.038897
AC010319.	0.365187	0.163124	0.817547	0.01429
AC145285.	0.415559	0.185053	0.933192	0.03338
AC016027.	0.324202	0.146212	0.718864	0.005564
ARHGEF2-	0.377135	0.157181	0.904886	0.028978
LINC02416	0.431983	0.199049	0.937501	0.033736
AC008610.	0.711345	0.512018	0.98827	0.042326
AL139287.	0.697569	0.518343	0.938765	0.017453
AC009283.	0.642318	0.472855	0.872514	0.004617
MAP3K14-	0.521001	0.311212	0.87221	0.013137
AL590652.	0.559906	0.368731	0.8502	0.0065
AC008915.	0.562263	0.357983	0.883112	0.012434
DICER1-AS	0.623441	0.396789	0.979557	0.040408
AC021016.	0.414103	0.190455	0.900381	0.026097
RNF216P1	1.740911	1.06886	2.835516	0.025912
AP003059.	0.164506	0.039109	0.691962	0.013803
FKBP14-AS	7.173571	1.676794	30.68959	0.007885
AC021016.	0.541439	0.349968	0.837665	0.005859
AL138756.	0.477022	0.296365	0.767803	0.002304
AC006435.	0.565918	0.381893	0.838619	0.004554
AL033384.	0.495621	0.313876	0.782605	0.002598
AL160314.	0.233504	0.057612	0.946414	0.04164
AC128687.	0.267397	0.085989	0.831512	0.022684
AL583810.	0.284095	0.087795	0.919298	0.035693
AC084782.	0.287464	0.089129	0.92715	0.036927
SNHG20	0.506172	0.324748	0.788949	0.00264
AC021321.	0.24024	0.112273	0.51406	0.000238
AC005332.	0.273055	0.076105	0.979683	0.046431
LINC01018	2.275727	1.283819	4.034005	0.004872
AC010359.	0.513046	0.271251	0.970376	0.040127
STAM-AS1	0.210041	0.053869	0.818972	0.024601
LINC02615	0.501802	0.32458	0.775789	0.001922
LINC01089	0.651435	0.479072	0.885814	0.006272
AC010300.	0.434963	0.212699	0.889485	0.022559
AC010618.	0.36229	0.182169	0.720508	0.003798
AP006621.	0.706634	0.503429	0.991861	0.044728
AC104785.	0.368026	0.198064	0.683834	0.001566
MIR29B2C	0.443271	0.258496	0.760124	0.003109
AC120053.	0.510965	0.351888	0.741956	0.000418
HMGA1P4	0.734442	0.540186	0.998555	0.048931
MSC-AS1	1.51212	1.122373	2.037208	0.006546
LINC01311	0.533464	0.312735	0.909984	0.021102
AC008870.	0.155351	0.030185	0.79954	0.025907

MYCL-AS1	0.647614	0.440184	0.952793	0.027423
C1orf147	0.108744	0.015274	0.774219	0.026727
AC114730.	0.481204	0.279422	0.8287	0.008353
AC010168.	0.433758	0.261768	0.718753	0.001189
AP000864.	0.453318	0.208732	0.984502	0.045561
CYTOR	1.253103	1.04689	1.499935	0.013914
PTPRK-AS	3.058778	1.079772	8.664907	0.035341
AC006557.	0.271904	0.076704	0.963859	0.043698
AC019131.	0.558223	0.358773	0.868552	0.009744
AC020911.	0.237452	0.066623	0.846312	0.026603
LINC0064S	0.533119	0.335488	0.847173	0.007773
AC010503.	0.784139	0.677613	0.907411	0.001098
AC022150.	0.71278	0.522136	0.973032	0.032997
AP001628.	0.669958	0.468035	0.958997	0.028615
AC013356.	0.316972	0.103815	0.967789	0.043648
Z98200.1	0.110722	0.039519	0.310213	2.83E-05
AC092171.	0.532626	0.335115	0.846546	0.007707
CSRP3-AS	0.195485	0.039141	0.976337	0.046685
LENG8-AS	0.576228	0.382453	0.868183	0.008393
AC020663.	0.412529	0.235025	0.724095	0.002038
AC108868.	88.37743	3.804545	2052.958	0.005229
AC097641.	0.505718	0.273735	0.9343	0.029484
AC104532.	0.344092	0.198956	0.595103	0.000135
DANCR	0.764987	0.619278	0.944979	0.012959
FLJ12825	0.075343	0.014481	0.392013	0.00212
AC245060.	0.426086	0.20124	0.902155	0.025814
ADGRD1-/	1.52888	1.038672	2.250446	0.031372
PTOV1-AS	0.587304	0.42157	0.818195	0.001655
AL662884.	0.128933	0.024097	0.689864	0.016674
AC007038.	0.563433	0.358932	0.884448	0.012642
SLC25A25-	0.545216	0.375748	0.791117	0.001405
AC002128.	0.501003	0.293678	0.85469	0.011209
AP006621.	0.534915	0.335534	0.852774	0.008557
HDAC4-AS	0.463997	0.269469	0.798955	0.005614
AC092171.	0.737967	0.571581	0.952788	0.019755
EXTL3-AS1	0.283484	0.082274	0.97677	0.045803
AL161452.	0.392934	0.18008	0.85738	0.018951
SGMS1-AS	0.232158	0.058113	0.927461	0.038779
AL049795.	0.14526	0.023681	0.891035	0.037103
AL355472.	0.473551	0.274045	0.818298	0.007395
AC008124.	0.545575	0.331291	0.898461	0.017282
AC107308.	1.48291	1.033256	2.128246	0.032562
AC020765.	0.609011	0.424524	0.873671	0.007071
AC011330.	0.394475	0.197234	0.788965	0.008534
AL355353.	0.701523	0.571027	0.861842	0.000736
FAM225A	2.014116	1.049488	3.865374	0.035275
AL353622.	0.11028	0.022631	0.53739	0.006361
AC013731.	0.395224	0.181269	0.861712	0.019585
AP001630.	0.413021	0.172697	0.987777	0.046855
SEC24B-AS	0.171289	0.05159	0.568716	0.003955
AC116914.	0.483994	0.318153	0.736282	0.000699
U47924.1	0.427756	0.230085	0.795249	0.007273
AC008735.	0.37151	0.158841	0.868919	0.022367
AC034236.	0.17405	0.036021	0.840996	0.029598
ASMTL-AS	0.655461	0.500486	0.858423	0.002147
SPAG5-AS	0.253861	0.096281	0.669347	0.005579
AF131215.	0.628483	0.42303	0.933718	0.021476
AC008543.	0.271565	0.105098	0.701704	0.007117

THAP9-AS	0.586144	0.410382	0.837184	0.003314
AP002840.	0.459407	0.269261	0.783829	0.004324
AC006042.	0.666258	0.529131	0.838921	0.000553
AC087741.	0.565307	0.384044	0.832124	0.003833
MIR302CH	0.128374	0.02254	0.731129	0.020734
AC011921.	0.362683	0.160324	0.820456	0.014888
AC244034.	0.3381	0.117929	0.969324	0.043597
MIR34AHG	0.438585	0.206398	0.931971	0.0321
AC023908.	0.307631	0.107518	0.880195	0.027957
ZFHX2-AS	0.182794	0.052596	0.635283	0.0075
AC090515.	0.574581	0.352749	0.935916	0.026012
AC068620.	0.46081	0.238322	0.891002	0.021278
LINC01011	0.499582	0.25476	0.979674	0.043412
AL353708.	0.473761	0.252007	0.890646	0.020366
AC108471.	0.107136	0.021525	0.533246	0.006375
TRPM2-AS	0.799666	0.64867	0.985812	0.036278
ZNF213-A	0.642641	0.431907	0.956196	0.029191
AL033527.	0.456073	0.243339	0.854787	0.014306
AC074044.	0.19084	0.06486	0.561514	0.002629
AC080129.	0.563169	0.35532	0.892601	0.014547
SNHG14	0.608323	0.382111	0.968454	0.036164
WASIR2	0.512456	0.336641	0.780092	0.001819
AC073288.	0.209248	0.06242	0.70145	0.01126
HOXB-AS3	0.709645	0.511291	0.984949	0.040303
AC017083.	0.493613	0.244006	0.998555	0.049532
AL731566.	0.290827	0.087825	0.963053	0.043219
LINC01176	0.646498	0.492011	0.849492	0.001744
VIM-AS1	1.533685	1.07844	2.181103	0.017301
AL354892.	0.392458	0.159093	0.968134	0.04233
AC100821.	0.437549	0.198613	0.963928	0.040254
AC124016.	0.600072	0.402366	0.894924	0.012267
AC018695.	0.749415	0.599926	0.936154	0.011048
AL513477.	0.47336	0.27612	0.811496	0.006539
AL021707.	0.494904	0.3483	0.703215	8.70E-05
AC005841.	0.421916	0.192277	0.925814	0.031381
AC004839.	0.144146	0.025467	0.815892	0.028522
AL391834.	0.517874	0.369854	0.725132	0.000127
AL390728.	0.557623	0.418178	0.743566	6.95E-05
AC079174.	0.42378	0.179612	0.999873	0.049966
H1-10-AS	0.556787	0.351423	0.882161	0.012633
GRASLND	2.079833	1.25028	3.459791	0.004799
ZNF436-A	0.60552	0.435627	0.841673	0.002828
MAPKAPK5	0.603738	0.401909	0.906919	0.015075
AC084876.	0.238526	0.073637	0.772637	0.016843
NBR2	0.643623	0.477858	0.86689	0.003731
ANKRD10-	0.795838	0.638707	0.991626	0.041861
AC010761.	0.433263	0.253808	0.739602	0.002173
AL355488.	0.653983	0.45679	0.936302	0.020371
AL133410.	0.405683	0.248106	0.663341	0.000323
AP001412.	0.334875	0.146451	0.765724	0.009527
AL138921.	0.175099	0.044029	0.696342	0.013369
AL358075.	0.642777	0.44538	0.927664	0.018221
AC092296.	0.284428	0.085973	0.940981	0.039435
AC008735.	0.455166	0.250388	0.827422	0.009845
AL662797.	0.304635	0.126144	0.735688	0.008234
AC024267.	0.284114	0.133243	0.605815	0.001125
AC011477.	0.661496	0.502832	0.870225	0.003143
AC107068.	0.551782	0.305878	0.995375	0.048227

AC012186.	0.551335	0.317288	0.958026	0.03468
UBE2Q1-A	0.325426	0.156458	0.676873	0.002661
AP000866.	0.649701	0.434952	0.970479	0.035176
AC011468.	0.545791	0.369026	0.807229	0.002426
PPP1R26-/	0.386864	0.211246	0.708482	0.002096
AL592211.	0.540378	0.33442	0.873178	0.011942
EHMT2-AS	0.1891	0.070242	0.509076	0.00098
AC073575.	0.447789	0.238244	0.841638	0.01258
AL390294.	0.704454	0.571382	0.868518	0.001039
FRMD6-AS	2.325945	1.284524	4.211694	0.005328
AC114488.	0.682024	0.519646	0.895143	0.005809
AC063948.	0.587693	0.363971	0.948929	0.029675
AC253576.	0.488483	0.291323	0.819075	0.006592
AC018809.	0.532342	0.327249	0.865971	0.011097
VIPR1-AS1	0.222963	0.077603	0.6406	0.00532
AC092802.	0.169562	0.038644	0.744011	0.018679
AC021491.	0.346922	0.122378	0.983468	0.046446
AL158196.	0.332682	0.136397	0.811436	0.015551
AP001625.	0.624072	0.395045	0.985879	0.043288
AC132872.	0.583512	0.380248	0.895434	0.013684
AC012615.	0.623071	0.420851	0.922457	0.018121
AP003419.	0.472416	0.293714	0.759845	0.001984
AC105001.	4.302714	1.584532	11.6838	0.004196
AL133551.	0.168748	0.034322	0.829676	0.028542
AC005229.	2.019953	1.342151	3.040054	0.000749
AC073534.	0.330708	0.186685	0.585839	0.000149
AC005387.	0.354327	0.156456	0.802446	0.012858
AL645940.	0.487442	0.274987	0.86404	0.013882
LINC00893	0.523326	0.300069	0.912691	0.022495
TRAPPC12	0.637279	0.424597	0.956494	0.029656
MORF4L2-	0.320989	0.107786	0.955908	0.041255
AL139041.	0.308787	0.138154	0.690169	0.004188
LINC01767	0.517223	0.348101	0.768511	0.001102
AC109347.	0.543847	0.326318	0.906384	0.019433
AC005306.	0.281127	0.122321	0.646105	0.002801
LINC01355	0.571741	0.396906	0.823589	0.00268
AC074212.	0.53219	0.298395	0.949166	0.032623
ZKSCAN2-	0.419646	0.24657	0.71421	0.001372
AC066613.	0.477298	0.242976	0.937594	0.031792
AC078820.	2.629349	1.443442	4.789575	0.00158
C1RL-AS1	0.554943	0.316495	0.973038	0.039844
AC021491.	0.505811	0.263272	0.971787	0.04077
AC024060.	0.531772	0.401712	0.703943	1.02E-05
AC005332.	0.640186	0.471577	0.869079	0.004241
AC104938.	14.16401	2.885454	69.52775	0.001093
AP005482.	0.575796	0.355331	0.933046	0.025003
RUSC1-AS	0.610371	0.407771	0.913631	0.016445
AC015802.	0.249831	0.076893	0.811724	0.02106
AC010422.	0.211902	0.069347	0.647502	0.006477
AL133415.	3.621644	1.605201	8.171129	0.001936
MRPL20-A	0.567878	0.359067	0.898121	0.015547
RGMB-AS1	0.470033	0.232839	0.948858	0.035166
LINC02481	0.494774	0.336368	0.727777	0.000352
AC009902.	0.226531	0.063418	0.809171	0.022258
KIF1C-AS1	0.32573	0.143138	0.741241	0.007502
AC020931.	0.085561	0.011323	0.646523	0.017189
GATA2-AS	0.718831	0.577599	0.894596	0.003097
AC010201.	0.431541	0.221329	0.841406	0.013631

AC090589.	0.574264	0.363191	0.908005	0.017654
AC004596.	0.522002	0.294669	0.924719	0.025866
HCG25	0.219097	0.078608	0.61067	0.003696
AC074117.	0.45167	0.291674	0.699433	0.000368
AC069307.	0.494765	0.290448	0.842809	0.009619
AL133297.	4.043453	1.163632	14.05041	0.027918
AL031716.	0.512147	0.296975	0.88322	0.016103
GARS1-DT	0.444604	0.211618	0.934099	0.032359
STAG3L5P	0.364743	0.199184	0.667914	0.001085
AL031289.	0.298393	0.093915	0.948069	0.040328
LINC00628	0.288775	0.096554	0.863672	0.026272
AC003070.	0.70166	0.555222	0.886721	0.003011
AL136295.	0.38652	0.198672	0.751983	0.00512
AC011481.	0.56587	0.335788	0.953603	0.032486
AC026704.	0.373422	0.178158	0.782698	0.009084
FARP1-AS	0.643537	0.429321	0.964641	0.03282
HNRNPD-	0.352165	0.153151	0.809794	0.014028
AL731567.	0.612287	0.488983	0.766684	1.91E-05
AL137244.	0.320401	0.125736	0.816444	0.017084
AC011498.	0.270744	0.073637	0.995456	0.049204
SCAT2	0.535616	0.338126	0.848452	0.00781
AC012615.	0.36528	0.191376	0.697213	0.002262
AC025280.	4.0585	1.891683	8.707284	0.000322
AL096870.	0.300888	0.094434	0.958694	0.042224
AC084018.	0.255663	0.069816	0.936225	0.039449
AP000697.	0.399058	0.194306	0.819569	0.012354
AC007319.	1.467028	1.016955	2.11629	0.040375
ALMS1-IT1	2.18144	1.282218	3.711289	0.004017
AL691482.	0.806832	0.699949	0.930038	0.003073
AL161729.	0.495639	0.27666	0.887943	0.018301
SNHG12	0.622568	0.431924	0.897358	0.011068
AC108860.	0.659489	0.481428	0.903408	0.009525
AC243830.	0.373575	0.194333	0.718141	0.003148
C2-AS1	0.167114	0.031384	0.889841	0.036016
AC010761.	0.701391	0.494949	0.993938	0.046135
AC098484.	0.329248	0.143518	0.755334	0.008734
MIR200CH	0.826789	0.720044	0.949359	0.007001
LINC00885	0.833242	0.697467	0.995448	0.044407
MCF2L-AS	0.843489	0.716596	0.992851	0.040735
AL583856.	0.299643	0.125007	0.718247	0.006894
AC015912.	0.737067	0.573297	0.947618	0.01733
AC130462.	0.196533	0.046938	0.8229	0.025964
AC008121.	0.199896	0.053542	0.746293	0.016604
AC068338.	0.56295	0.326148	0.971683	0.039102
AP003059.	0.203766	0.048093	0.863339	0.030816
AC132192.	0.604646	0.378112	0.966902	0.035686
AL139349.	0.498682	0.322637	0.770785	0.001737
AL162258.	0.386846	0.206529	0.724597	0.003017
STARD7-A	0.464491	0.232188	0.929211	0.030197
AC025178.	0.368172	0.169059	0.801793	0.011861
AC027601.	0.315737	0.111893	0.890942	0.029396
AL391684.	0.345197	0.153615	0.775712	0.010031
AC013403.	0.439357	0.207229	0.931507	0.031952
AC005954.	0.10617	0.023407	0.481563	0.003647
LINC01977	0.584896	0.352512	0.970471	0.037895
C9orf163	0.326385	0.117652	0.905442	0.031494
AC018904.	0.710769	0.585092	0.863442	0.000584
NFYC-AS1	0.506373	0.289349	0.886174	0.017164

AC004148.	0.547903	0.393368	0.763146	0.000372
AC016737.	0.494558	0.268395	0.911297	0.023957
AC022762.	0.443588	0.220932	0.890636	0.022276
AL031429.	4.536346	1.654998	12.43412	0.00329
AL357140.	0.428606	0.187572	0.979373	0.044495
AC084018.	0.703423	0.537405	0.920729	0.010429
AC055822.	0.568917	0.391598	0.826527	0.003079
NARF-AS1	0.219375	0.052906	0.909636	0.036575
AL353622.	0.480084	0.316227	0.728847	0.000572
AL158063.	0.351656	0.167451	0.738494	0.005767
ARHGAP2	0.650796	0.465487	0.909875	0.011993
ZNF32-AS	0.347652	0.190386	0.634826	0.000584
RNF139-A	0.303163	0.105936	0.867581	0.026098
SUGT1-DT	0.455275	0.279334	0.742034	0.001594
FABP6-AS	0.080233	0.013703	0.469766	0.005144
WARS2-AS	0.53732	0.290409	0.99416	0.047859
KRT7-AS	0.823703	0.725879	0.934709	0.002641
AC007686.	0.161723	0.031083	0.841432	0.030377
AC087286.	1.845373	1.059404	3.21445	0.030483
LINC0174	0.628693	0.444617	0.888977	0.008645
GEMIN7-A	0.420514	0.223795	0.790149	0.007106
AP000866.	0.164878	0.032649	0.832628	0.029135
AC104187.	0.115631	0.029511	0.453064	0.00196
PSPC1-AS	0.601231	0.381174	0.948329	0.02866
AL008582.	0.422072	0.23528	0.757159	0.003817
AL513320.	0.538642	0.330409	0.878109	0.013092
AC008735.	0.710658	0.544842	0.926939	0.011748
AC253536.	0.574785	0.365321	0.904349	0.016632
AC093788.	0.400707	0.22895	0.701315	0.001363
ZNF252P-	0.127214	0.031029	0.521561	0.004181
AL159169.	0.414863	0.175192	0.982413	0.045467
AC002128.	0.460554	0.234826	0.903268	0.024071
U91328.3	0.25563	0.093439	0.699352	0.007899
CTBP1-AS	0.297619	0.133618	0.662916	0.003016
AC004253.	0.470032	0.285881	0.772805	0.002921
LINC0100	0.669347	0.464838	0.96383	0.030928
AATBC	0.757323	0.640098	0.896016	0.001197
LINC0011	0.417269	0.196825	0.88461	0.022621
