# Supplementary material for: 5-Methylcytosine-Related Long Noncoding RNAs Are Potential Biomarkers to Predict Overall Survival and Regulate Tumor-Immune Environment in Patients with Bladder Cancer
Source: Dis Markers. 2022 Mar 4;2022:3117359. doi: 10.1155/2022/3117359 (PMC8966750; doi:10.1155/2022/3117359)
Supplement: Supplementary 3 — Table s3: enrichment pathways with significant differences. [file 3117359.f3.pdf]

Pathway	logFC
KEGG_MELANOMA	-0.22939
KEGG_REGULATION_OF_ACTIN_CYTOSKELETON	-0.22059
KEGG_RENAL_CELL_CARCINOMA	-0.20934
KEGG_GLIOMA	-0.20908
KEGG_GAP_JUNCTION	-0.1992
KEGG_MAPK_SIGNALING_PATHWAY	-0.16078
KEGG_PROSTATE_CANCER	-0.19387
KEGG_MELANOGENESIS	-0.16939
KEGG_AXON_GUIDANCE	-0.17895
KEGG_FOCAL_ADHESION	-0.2476
KEGG_PATHWAYS_IN_CANCER	-0.16986
KEGG_COLORECTAL_CANCER	-0.19354
KEGG_LONG_TERM_DEPRESSION	-0.14743
KEGG_NEUROTROPHIN_SIGNALING_PATHWAY	-0.17537
KEGG_LONG_TERM_POTENTIATION	-0.1702
KEGG_ARRHYTHMOGENIC_RIGHT_VENTRICULAR_CARDIOMYOPATHY_ARVC	-0.23233
KEGG_PANCREATIC_CANCER	-0.18989
KEGG_WNT_SIGNALING_PATHWAY	-0.15452
KEGG_SMALL_CELL_LUNG_CANCER	-0.18939
KEGG_ADHERENS_JUNCTION	-0.19199
KEGG_ENDOMETRIAL_CANCER	-0.19189
KEGG_TYPE_II_DIABETES_MELLITUS	-0.14037
KEGG_EPITHELIAL_CELL_SIGNALING_IN_HELICOBACTER_PYLORI_INFECTION	-0.16456
KEGG_DORSO_VENTRAL_AXIS_FORMATION	-0.20495
KEGG_ACUTE_MYELOID_LEUKEMIA	-0.17652
KEGG_TGF_BETA_SIGNALING_PATHWAY	-0.16483
KEGG_ECM_RECEPTOR_INTERACTION	-0.27122
KEGG_CALCIUM_SIGNALING_PATHWAY	-0.14876
KEGG_VASCULAR_SMOOTH_MUSCLE_CONTRACTION	-0.16331
KEGG_ALDOSTERONE_REGULATED_SODIUM_REABSORPTION	-0.15502
KEGG_LEUKOCYTE_TRANSENDOTHELIAL_MIGRATION	-0.19296
KEGG_MTOR_SIGNALING_PATHWAY	-0.13979
KEGG_DILATED_CARDIOMYOPATHY	-0.20783
KEGG_FC_GAMMA_R_MEDIATED_PHAGOCYTOSIS	-0.16091
KEGG_HYPERTROPHIC_CARDIOMYOPATHY_HCM	-0.20807
KEGG_B_CELL_RECEPTOR_SIGNALING_PATHWAY	-0.1828
KEGG_RENIN_ANGIOTENSIN_SYSTEM	-0.21658
KEGG_PRION_DISEASES	-0.20336
KEGG_CHRONIC_MYELOID_LEUKEMIA	-0.16405
KEGG_PROGESTERONE_MEDIATED_OOCYTE_MATURATION	-0.1619
KEGG_NON_SMALL_CELL_LUNG_CANCER	-0.14999
KEGG_ERBB_SIGNALING_PATHWAY	-0.14841
KEGG_OOCYTE_MEIOSIS	-0.17454
KEGG_GLYCOSAMINOGLYCAN_BIOSYNTHESIS_CHONDROITIN_SULFATE	-0.2453
KEGG_CHEMOKINE_SIGNALING_PATHWAY	-0.18547
KEGG_ADIPOCYTOKINE_SIGNALING_PATHWAY	-0.1326
KEGG_GNRH_SIGNALING_PATHWAY	-0.11183
KEGG_TIGHT_JUNCTION	-0.11812
KEGG_NEUROACTIVE_LIGAND_RECEPTOR_INTERACTION	-0.13151
KEGG_PATHOGENIC_ESCHERICHIA_COLI_INFECTION	-0.17428
KEGG_VIBRIO_CHOLERAE_INFECTION	-0.14071
KEGG_COMPLEMENT_AND_COAGULATION_CASCADES	-0.20614
KEGG_JAK_STAT_SIGNALING_PATHWAY	-0.16184
KEGG_T_CELL_RECEPTOR_SIGNALING_PATHWAY	-0.17075
KEGG_FC_EPSILON_RI_SIGNALING_PATHWAY	-0.11187
KEGG_BLADDER_CANCER	-0.1424
KEGG_INSULIN_SIGNALING_PATHWAY	-0.10293

KEGG_TOLL_LIKE_RECEPTOR_SIGNALING_PATHWAY	-0.16278
KEGG_VEGF_SIGNALING_PATHWAY	-0.10116
KEGG_CELL_ADHESION_MOLECULES_CAMS	-0.20364
KEGG_NOD_LIKE_RECEPTOR_SIGNALING_PATHWAY	-0.17938
KEGG_GLYCOSAMINOGLYCAN_DEGRADATION	-0.18825
KEGG_GLYCOSAMINOGLYCAN_BIOSYNTHESIS_KERATAN_SULFATE	-0.17834
KEGG_LYSOSOME	-0.15328
KEGG_PRIMARY_BILE_ACID_BIOSYNTHESIS	-0.15917
KEGG_CYTOKINE_CYTOKINE_RECEPTOR_INTERACTION	-0.18509
KEGG_PURINE_METABOLISM	-0.10577
KEGG_LEISHMANIA_INFECTION	-0.22089
KEGG_VIRAL_MYOCARDITIS	-0.20083
KEGG_APOPTOSIS	-0.13337
KEGG_HEMATOPOIETIC_CELL_LINEAGE	-0.20667
KEGG_NATURAL_KILLER_CELL_MEDIATED_CYTOTOXICITY	-0.15436
KEGG_AMYOTROPHIC_LATERAL_SCLEROSIS_ALS	-0.10189
KEGG_SNARE_INTERACTIONS_IN_VESICULAR_TRANSPORT	-0.11627
KEGG_GLYCOSPHINGOLIPID_BIOSYNTHESIS_LACTO_AND_NEOLACTO_SERIES	-0.12368
KEGG_VASOPRESSIN_REGULATED_WATER_REABSORPTION	-0.10911
KEGG_O_GLYCAN_BIOSYNTHESIS	-0.12836
KEGG_GALACTOSE_METABOLISM	-0.12892
KEGG_GLYCOSPHINGOLIPID_BIOSYNTHESIS_GANGLIO_SERIES	-0.15731
KEGG_SPHINGOLIPID_METABOLISM	-0.10383
KEGG_PYRUVATE_METABOLISM	-0.11547
KEGG_NICOTINATE_AND_NICOTINAMIDE_METABOLISM	-0.10386
KEGG_LINOLEIC_ACID_METABOLISM	0.137342
KEGG_BIOSYNTHESIS_OF_UNSATURATED_FATTY_ACIDS	-0.13347
KEGG_AMINO_SUGAR_AND_NUCLEOTIDE_SUGAR_METABOLISM	-0.11823
KEGG_HEDGEHOG_SIGNALING_PATHWAY	-0.10168
KEGG_BASAL_CELL_CARCINOMA	-0.10472
KEGG_PANTOTHENATE_AND_COA_BIOSYNTHESIS	-0.10394
KEGG_SYSTEMIC_LUPUS_ERYTHEMATOSUS	-0.12437
KEGG_CIRCADIAN_RHYTHM_MAMMAL	-0.13619
KEGG_ALPHA_LINOLENIC_ACID_METABOLISM	0.115412
KEGG_ASTHMA	-0.19297
KEGG_RIBOSOME	0.215835
KEGG_INTESTINAL_IMMUNE_NETWORK_FOR_IGA_PRODUCTION	-0.17407
KEGG_CITRATE_CYCLE_TCA_CYCLE	-0.13707
KEGG_MISMATCH_REPAIR	-0.16899
KEGG_CELL_CYCLE	-0.12112
KEGG_PROPANOATE_METABOLISM	-0.10378
KEGG_LIMONENE_AND_PINENE_DEGRADATION	-0.10968
KEGG_AUTOIMMUNE_THYROID_DISEASE	-0.14776
KEGG_STEROID_BIOSYNTHESIS	-0.11807
KEGG_NUCLEOTIDE_EXCISION_REPAIR	-0.1112
KEGG_OXIDATIVE_PHOSPHORYLATION	0.106504
KEGG_ALLOGRAFT_REJECTION	-0.16648
KEGG_ANTIGEN_PROCESSING_AND_PRESENTATION	-0.12353
KEGG_DNA_REPLICATION	-0.15253
KEGG_GRAFT_VERSUS_HOST_DISEASE	-0.15835
KEGG_TYPE_I_DIABETES_MELLITUS	-0.14135
KEGG_PROTEASOME	-0.12017
KEGG_PRIMARY_IMMUNODEFICIENCY	-0.11595

AveExpr	t	P.Value	adj.P.Val	B
-0.02538	-10.7719	5.45E-24	1.01E-21	43.7201
-0.03472	-9.76224	2.23E-20	2.08E-18	35.56857
-0.04286	-9.49078	1.92E-19	1.03E-17	33.4602
-0.03599	-9.47328	2.21E-19	1.03E-17	33.32557
-0.03737	-9.36856	5.01E-19	1.86E-17	32.52322
-0.0305	-9.22363	1.54E-18	4.78E-17	31.42229
-0.04048	-9.20086	1.84E-18	4.89E-17	31.2504
-0.03327	-9.08887	4.35E-18	9.04E-17	30.4087
-0.0417	-9.08803	4.37E-18	9.04E-17	30.40242
-0.04129	-8.86616	2.36E-17	4.38E-16	28.75529
-0.03477	-8.84302	2.81E-17	4.74E-16	28.58508
-0.04226	-8.69112	8.73E-17	1.35E-15	27.47518
-0.01535	-8.61473	1.54E-16	2.20E-15	26.922
-0.03669	-8.54568	2.56E-16	3.40E-15	26.42489
-0.02263	-8.49265	3.77E-16	4.68E-15	26.04505
-0.03203	-8.47665	4.24E-16	4.93E-15	25.93075
-0.04763	-8.28799	1.67E-15	1.82E-14	24.5944
-0.03624	-8.25443	2.12E-15	2.19E-14	24.35897
-0.04681	-8.10872	6.00E-15	5.87E-14	23.34445
-0.03984	-8.03235	1.03E-14	9.57E-14	22.81794
-0.03086	-8.02029	1.12E-14	9.92E-14	22.73511
-0.02155	-7.89957	2.61E-14	2.20E-13	21.91112
-0.02743	-7.89097	2.77E-14	2.24E-13	21.85278
-0.02841	-7.84321	3.85E-14	2.99E-13	21.52952
-0.04489	-7.81171	4.79E-14	3.57E-13	21.31716
-0.04924	-7.78275	5.85E-14	4.19E-13	21.12248
-0.04499	-7.77375	6.23E-14	4.29E-13	21.06203
-0.03364	-7.76348	6.68E-14	4.44E-13	20.99321
-0.03838	-7.72733	8.56E-14	5.49E-13	20.75136
-0.03311	-7.69076	1.10E-13	6.82E-13	20.50753
-0.02867	-7.58664	2.23E-13	1.34E-12	19.81817
-0.04902	-7.5302	3.27E-13	1.90E-12	19.44738
-0.03454	-7.52548	3.37E-13	1.90E-12	19.41648
-0.0467	-7.47236	4.81E-13	2.63E-12	19.0696
-0.03589	-7.42065	6.80E-13	3.61E-12	18.73372
-0.03873	-7.37082	9.46E-13	4.89E-12	18.41175
-0.02738	-7.26002	1.96E-12	9.87E-12	17.70169
-0.02498	-7.21434	2.65E-12	1.30E-11	17.41139
-0.04376	-7.20481	2.82E-12	1.34E-11	17.35104
-0.04082	-7.18867	3.13E-12	1.45E-11	17.24889
-0.04073	-7.18085	3.29E-12	1.49E-11	17.19948
-0.03175	-7.12387	4.76E-12	2.11E-11	16.84064
-0.03465	-7.10819	5.27E-12	2.28E-11	16.74227
-0.05069	-6.90602	1.91E-11	8.08E-11	15.48924
-0.03996	-6.86403	2.49E-11	1.03E-10	15.23257
-0.02284	-6.7797	4.22E-11	1.71E-10	14.72084
-0.03451	-6.69455	7.15E-11	2.83E-10	14.20927
-0.02313	-6.58299	1.42E-10	5.48E-10	13.54681
-0.0121	-6.56971	1.53E-10	5.82E-10	13.46854
-0.03803	-6.53903	1.85E-10	6.88E-10	13.28823
-0.01364	-6.49559	2.40E-10	8.76E-10	13.0341
-0.01691	-6.41624	3.87E-10	1.38E-09	12.57349
-0.03386	-6.34913	5.76E-10	2.02E-09	12.18749
-0.04755	-6.30141	7.63E-10	2.63E-09	11.91505
-0.04151	-6.29681	7.84E-10	2.65E-09	11.88886
-0.04764	-6.27615	8.85E-10	2.94E-09	11.77149
-0.03414	-6.26571	9.41E-10	3.07E-09	11.71233

-0.03487	-6.23533	1.12E-09	3.60E-09	11.54055
-0.03907	-6.14673	1.88E-09	5.92E-09	11.04355
-0.02994	-6.13587	2.00E-09	6.20E-09	10.98307
-0.04229	-6.09326	2.56E-09	7.79E-09	10.74647
-0.03101	-6.04892	3.29E-09	9.88E-09	10.50179
-0.04812	-6.01068	4.09E-09	1.21E-08	10.29191
-0.0329	-5.929	6.48E-09	1.87E-08	9.847444
-0.05236	-5.92737	6.54E-09	1.87E-08	9.838602
-0.03742	-5.81988	1.19E-08	3.35E-08	9.261589
-0.04104	-5.80921	1.26E-08	3.50E-08	9.204816
-0.04449	-5.79082	1.40E-08	3.82E-08	9.107129
-0.03132	-5.58744	4.21E-08	1.14E-07	8.044511
-0.03844	-5.56395	4.77E-08	1.27E-07	7.923856
-0.02637	-5.51198	6.29E-08	1.65E-07	7.658509
-0.03281	-5.3658	1.35E-07	3.49E-07	6.92363
-0.01344	-5.34258	1.52E-07	3.88E-07	6.808474
-0.04086	-5.3051	1.85E-07	4.65E-07	6.623508
-0.04679	-5.23564	2.63E-07	6.53E-07	6.28371
-0.02969	-5.0584	6.39E-07	1.54E-06	5.434497
-0.01744	-4.99223	8.85E-07	2.11E-06	5.12403
-0.01748	-4.94251	1.13E-06	2.65E-06	4.893156
-0.04207	-4.92535	1.22E-06	2.85E-06	4.813932
-0.0476	-4.89982	1.38E-06	3.18E-06	4.696523
-0.03773	-4.87984	1.52E-06	3.46E-06	4.605059
-0.05713	-4.87049	1.59E-06	3.57E-06	4.562339
-0.01749	4.867139	1.62E-06	3.59E-06	4.547055
-0.06183	-4.7937	2.30E-06	5.02E-06	4.214387
-0.03832	-4.7885	2.35E-06	5.09E-06	4.190997
-0.04117	-4.76261	2.66E-06	5.68E-06	4.074912
-0.03903	-4.63078	4.90E-06	1.04E-05	3.492501
-0.02719	-4.55758	6.84E-06	1.43E-05	3.175481
-0.00777	-4.38947	1.45E-05	2.96E-05	2.46474
-0.04839	-4.22384	2.96E-05	5.92E-05	1.788224
-0.02573	4.138126	4.25E-05	8.28E-05	1.447483
-0.03383	-4.1227	4.54E-05	8.70E-05	1.386859
-0.0172	4.018505	6.97E-05	0.000131	0.982704
-0.03889	-3.97347	8.37E-05	0.000156	0.810993
-0.03579	-3.95533	9.01E-05	0.000166	0.7423
-0.02732	-3.90596	0.00011	0.000198	0.556914
-0.03863	-3.66535	0.00028	0.000477	-0.31563
-0.04916	-3.60745	0.000347	0.000582	-0.51785
-0.05942	-3.55646	0.00042	0.000697	-0.69344
-0.0138	-3.44345	0.000634	0.001016	-1.07424
-0.0445	-3.36705	0.000832	0.0013	-1.32509
-0.04327	-3.31267	0.001006	0.001547	-1.50038
-0.02256	3.201664	0.001473	0.002227	-1.84985
-0.04158	-3.19725	0.001495	0.002232	-1.86353
-0.02223	-3.1963	0.0015	0.002232	-1.86645
-0.0168	-3.15873	0.001702	0.002493	-1.98197
-0.03002	-3.11111	0.001994	0.002876	-2.12654
-0.0377	-3.00416	0.002827	0.004005	-2.44358
-0.01255	-2.80269	0.005309	0.007207	-3.01192
-0.03484	-2.75058	0.006213	0.008314	-3.15273
